# Air pollution disproportionately impairs beneficial invertebrates: a meta-analysis

**DOI:** 10.1038/s41467-024-49729-5

**Published:** 2024-07-11

**Authors:** James M. W. Ryalls, Jacob Bishop, Adedayo O. Mofikoya, Lisa M. Bromfield, Shinichi Nakagawa, Robbie D. Girling

**Affiliations:** 1 School of Agriculture, Policy and Development, University of Reading, Reading, Berkshire RG6 6EU UK; 2 Evolution and Ecology Research Centre, School of Biological and Environmental Science, University of New South Wales, Sydney, NSW 2052 Australia; 3 Department of Biological Sciences, University of Alberta, CW 405, Biological Sciences Building, Edmonton, AB T6G 2E9 Canada; 4 Centre for Sustainable Agricultural Systems, Institute for Life Sciences and the Environment, University of Southern Queensland, Toowoomba, QLD 4350 Australia

**Keywords:** Ecosystem services, Entomology, Behavioural ecology, Atmospheric chemistry, Ecosystem ecology

## Abstract

Air pollution has the potential to disrupt ecologically- and economically-beneficial services provided by invertebrates, including pollination and natural pest regulation. To effectively predict and mitigate this disruption requires an understanding of how the impacts of air pollution vary between invertebrate groups. Here we conduct a global meta-analysis of 120 publications comparing the performance of different invertebrate functional groups in unpolluted and polluted atmospheres. We focus on the pollutants ozone, nitrogen oxides, sulfur dioxide and particulate matter. We show that beneficial invertebrate performance is reduced by air pollution, whereas the performance of plant pest invertebrates is not significantly affected. Ozone pollution has the most detrimental impacts, and these occur at concentrations below national and international air quality standards. Changes in invertebrate performance are not dependent on air pollutant concentrations, indicating that even low levels of pollution are damaging. Predicted increases in tropospheric ozone could result in unintended consequences to global invertebrate populations and their valuable ecological services.

## Abstract (web summary)

Air pollution has the potential to harm invertebrates. This meta-analysis compares invertebrate performance in unpolluted and polluted atmospheres, finding negative impacts on beneficial invertebrates and detrimental impacts from low levels of ozone.

## Introduction

Many of the essential ecosystem services that nature provides, including nutrient cycling, pest control, pollination, and the maintenance of soil structure and fertility, are reliant on the actions of invertebrate species^1^. However, globally, invertebrate populations are fundamentally threatened by a range of human activities including land use change, the introduction of alien invasive species, and air pollution^2^. Common air pollutants, derived from anthropogenic origins, can cause significant reductions in invertebrate fitness^3–7^. Air pollutants can have direct impacts by inducing changes at physiological and molecular levels^8–11^. They can also have indirect impacts by inducing changes to the nutritional status of host plants, or by disrupting odor-mediated navigation and communication^7,12–15^ through chemical reactions that modify odor cues and signaling compounds (volatile organic compounds [VOCs]). To-date, research on the impacts of air pollution on invertebrates has focused on either individual species or the interactions between two species in controlled laboratory or field studies, with little understanding of wider landscape-scale impacts. While previous meta-analyses have synthesized the effects of a number of air pollutants on invertebrates^3,16–21^, no studies have identified how we could effectively predict the broad impacts of air pollution across invertebrate communities. Relative to other factors contributing to the decline of invertebrate populations, this knowledge gap highlights a significant lack of understanding about the extent and consistency of air pollution impacts across all invertebrate groups. This means that it is not yet possible to estimate the potential impacts of air pollution on insect-provisioned ecosystem services and disservices.

Air pollutants that are elevated as a result of anthropogenic activity, including ozone (O_3_), sulfur dioxide (SO_2_), nitrogen oxides (NO_*x*_, comprising nitric oxide [NO] and nitrogen dioxide [NO_2_]) and respirable suspended particulate matter (PM), can all alter the abundance, health, and distribution of invertebrates^3–7,21,22^. Concentrations of tropospheric O_3_, produced in photochemical reactions between NO_*x*_ and VOCs^23^, have more than doubled from pre-industrial times and the frequency of high-O_3_ episodes is projected to increase in the coming decades^24,25^. Nitrogen oxides are emitted predominantly by combustion engine vehicles and, despite legislation and a transition towards electric vehicles, will continue to be an important air pollutant due to the long average lifespans of combustion engine vehicles (>20 years)^26^. SO_2_ is primarily released from the burning of fossil fuels, especially coal, for energy generation and domestic heating^27^. Complex reactions of chemicals, including SO_2_ and NO_*x*_, form fine particles respirable by humans, or PM, which are most prevalent in industrial, urban, and high-traffic areas^28^. Elevated levels of PM have also been linked to an increase in wildfires as a result of human-induced climate change, with significant repercussions for rural areas^29^. Studies have investigated the effects of each of these pollutants on the performance of a range of invertebrate species (Supplementary Data 1), but without comparisons of impacts between groups, it is difficult to predict which are most at risk.

**Fig. 1:**
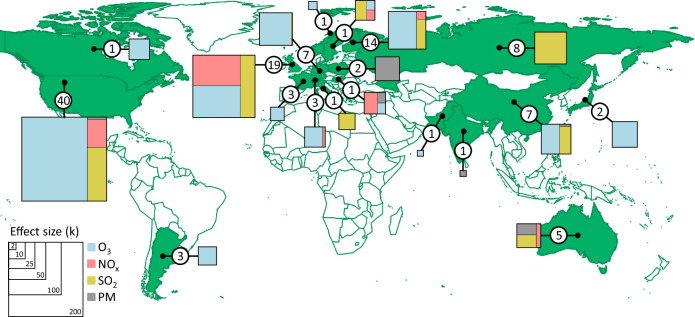
Geographical distribution of studies included in the meta-analysis. For each included country (highlighted green), the total number of publications (circled) is shown. Map and centroids (points) for each country used the Natural Earth data set with the R package ‘maps’. The treemaps (produced by the R package ‘treemap’) shown for each country are scaled by the total number of effect sizes and indicate the proportion of effect sizes per pollutant.

Here, we conduct a multi-level meta-analysis to identify simple and generalizable ways to understand the variation in how invertebrate performance responds to air pollution. Understanding what can be generalized is essential because knowledge of the diversity of responses of invertebrate groups to air pollutants globally remains limited. Functional trait-based approaches (i.e. identifying characteristics shared between species) facilitate holistic predictions into how invertebrate communities respond to environmental change, but they are underutilized in general, especially in the context of air pollution^30–32^. We quantify the effects of air pollution on invertebrate performance (with respect to individual species, populations, and communities of invertebrates), defined broadly as invertebrate abundance, feeding efficiency, growth/development, survival, searching efficiency, diversity, and reproduction, by calculating the ratio of invertebrate performance in control conditions and in elevated pollutant conditions. This allows us to combine the results of experimental studies across 120 publications (Supplementary Data 1, Supplementary Fig. 1), four air pollutants, over forty invertebrate families, and a total of 877 effect sizes across 19 countries (Fig. 1). We compare the extent to which different predictors explain variation in the effects of air pollution on invertebrate performance; these predictors include different levels of invertebrate taxonomic classification, different ways of measuring their functional characteristics, and the plant species that the invertebrates were interacting with in each experimental study (Tables 1 and 2, Supplementary Note 3). For example, the category ‘pest status’ comprises of beneficial invertebrates (i.e. those providing ecological and economic benefits to humans in the form of decomposition, pollination, and pest control services), significant pest invertebrates (i.e. those appearing in at least one of three global databases of economically important plant pests) and other herbivores (i.e. those not included in these databases and not considered economically beneficial). See the “Methods” section for full details on classifications. We focus on invertebrate responses to individual air pollutants because interactions between pollutants are complex, and only a handful of empirical studies^13,15,32–36^ exist on invertebrate responses to mixtures of pollutants. We also explore whether the effects of air pollutants on invertebrate performance vary with pollutant concentration, to understand the potential role of mitigation strategies. This study offers insights into the complex interactions between air pollution and different invertebrate groups, the results of which can inform future policies to regulate air pollution and the development of management plans to mitigate the effects on those most vulnerable invertebrate groups. We show that beneficial invertebrates, such as pollinators and natural pest regulators, which are essential for food security, are adversely affected by air pollution. Conversely, air pollution has no impact on herbivore pest invertebrates. Of the four pollutants, tropospheric ozone has the most detrimental effect on beneficial invertebrates, impairing their performance even at low concentrations.

**Table 1:** Summary of predictors (moderators) of responses of invertebrate performance to elevated concentrations of air pollution (i.e. overall effects of all four air pollutants: Ozone, nitrogen oxides, sulfur dioxide and particulate matter)

Moderator	Categories	df	LRT	*P*	*R*^2^
Pest status	Beneficial, Significant pest. Other herbivore	3, 860	25.60	<0.0001*	0.103
Feeding guild	Borer/miner, Cell-feeder (includes phloem-feeders), Chewer, Detritivore, Pollinator, Parasitoid, Predator	7, 814	39.11	<0.0001*	0.146
Invertebrate Order	Acari, Astigmata, Chilopoda, Coleoptera, Collembola, Diplopoda, Diptera, Haplotaxida, Hemiptera, Homoptera, Hymenoptera, Lepidoptera, Mesostigmata, Orbatida, Orthoptera, Prostigmata, Psocoptera, Thysanoptera, Trombidiformes	19, 795	40.71	0.0017*	0.167
Invertebrate Family	See Supplementary Information for full list	37, 721	59.35	0.0085*	0.176
Lifestage	Adult, Egg, Larva, Multiple, Nymph, Pupa	6, 746	2.04	0.844	0.007
Winged	No, Yes, Both	3, 711	3.81	0.149	0.015
Diet specialization	Generalist, Specialist	2, 693	1.66	0.198	0.011
Plant Order	Asterales, Brassicales, Caryophyllales, Cucurbitales, Dipsacales, Fabales, Fagales, Gentianales, Lamiales, Magnoliales, Malpighiales, Malvales, Pinales, Poales, Polypodiales, Rosales, Sapindales, Solanales	19, 701	21.96	0.234	0.104
Plant Family	See Supplementary Information for full list	20, 700	23.57	0.213	0.102
Annuality	Annual, Biennial, Perennial	3, 689	1.80	0.408	0.011
Plant type	Monocot (Angiosperm), Dicot (Angiosperm), Gymnosperm	3, 718	2.15	0.342	0.014

Statistics presented are likelihood ratio test comparisons between a uni-moderator model and a nested null model containing only random effects and marginal *R*^2^ of the uni-moderator model. Significance indicated by *P < 0.05.

**Table 2:** Summary of predictors (moderators) of responses of invertebrate performance to elevated concentrations of four individual air pollutants: Ozone (O_3_), nitrogen oxides (NO_*x*_), sulfur dioxide (SO_2_), and particulate matter (PM)

Moderator	O_3_			NO_*x*_			SO_2_			PM		
	df	*P*	*R*^2^	df	*P*	*R*^2^	df	*P*	*R*^2^	df	*P*	*R*^2^
Pest status	3,468	<0.0001*	0.113	3,86	0.016*	0.140	3,242	0.041	0.079	3,55	0.091	0.162
Feeding guild	7,446	<0.0001*	0.171	4,65	0.0009*	0.374	5,217	0.176	0.087	2,50	0.620	0.012
Invertebrate Order	11,441	0.032*	0.119	5,60	0.071	0.341	15,224	0.083	0.233	5,53	0.216	0.154
Invertebrate Family	25,399	0.005*	0.225	5,57	0.012*	0.363	18,200	0.456	0.141	5,49	0.199	0.069
Lifestage	4,422	0.912	0.004	4,85	0.475	0.051	6,177	0.116	0.106	4,50	0.807	0.008
Winged	3,379	0.364	0.018	3,83	0.497	0.031	3,185	0.540	0.028	3,55	0.734	0.052
Diet specialization	2,388	0.685	0.002	2,48	0.309	0.042	2,198	0.150	0.028	–	–	–
Plant Order	17,423	0.212	0.138	8,63	0.082	0.441	7,157	0.422	0.100	3,42	0.011*	0.491
Plant Family	18,422	0.080	0.172	8,63	0.082	0.441	6,158	0.308	0.100	3,42	0.011*	0.491
Annuality	3,403	0.634	0.008	2,69	0.140	0.143	2,168	0.752	0.002	2,43	0.125	0.011
Plant type	3,432	0.972	0.001	3,68	0.891	0.002	3,167	0.125	0.074	2,43	0.184	0.106

Statistics presented are likelihood ratio test comparisons between a uni-moderator model and a nested null model containing only random effects and marginal *R*^2^ of the uni-moderator model. Significance indicated by *P < 0.05.

## Results

### Effects of air pollution on invertebrates

**Fig. 2:**
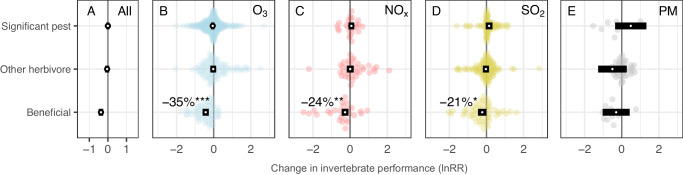
The effects of air pollution on pest and beneficial invertebrate performance. Orchard plots of meta-analytic mean effect sizes (ln RR; log response ratio) for each of three levels of invertebrate pest status. White points represent meta-analytic means and black rectangles represent the 95% confidence intervals from a model across all pollutants (**A**) or individual pollutants (**B**–**E**; ozone (O_3_), nitrogen oxides (NO_*x*_), sulfur dioxide (SO_2_) and particulate matter (PM), respectively). Points to the left of zero indicate negative impacts and points to the right indicate positive impacts. 95% confidence intervals overlapping the zero line indicate the mean estimate is not significantly different from zero (*P* < 0.05). Significant effects of O_3_, NO_*x*_, and SO_2_ are indicated by ***P < 0.0001, **P = 0.004 and *P = 0.046, respectively. Number of effect sizes for significant pests, other herbivores, and beneficial invertebrates (top to bottom): All (415, 282, 166), O_3_ (302, 95, 74), NO_*x*_ (26, 36, 27), SO_2_ (80, 111, 54), PM (7, 40, 11). Source data are provided as a Source Data file.

Elevated concentrations of air pollution (i.e. all pollutants considered together) reduced the performance of beneficial invertebrates by 31.3% compared with control conditions (confidence interval (CI) = 22.2–39.3%, *P* < 0.001) while, in contrast, the performance of significant pest invertebrates (CI = −8.3% to 8.4%, *P* = 0.924) and other herbivores (CI = −6.7% to 14.0%, *P* = 0.435) was unaffected by air pollution (Fig. 2A).

**Fig. 3:**
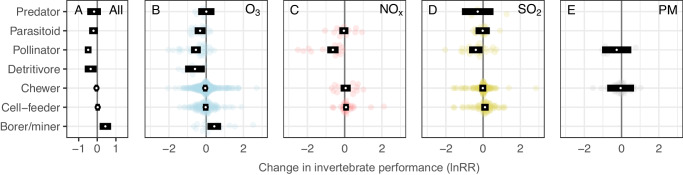
The effects of air pollution on the performance of invertebrates from different feeding guilds. Orchard plots of meta-analytic mean effect sizes (lnRR; log response ratio) for up to seven invertebrate feeding guilds. Model estimates for each feeding guild calculated from less than three studies were not included. White points represent meta-analytic means and black rectangles represent the 95% confidence intervals from a model across all pollutants (**A**) or individual pollutants (**B**–**E**; ozone (O_3_), nitrogen oxides (NO_*x*_), sulfur dioxide (SO_2_) and particulate matter (PM), respectively). Points to the left of zero indicate negative impacts and points to the right indicate positive impacts. 95% confidence intervals overlapping the zero line indicate the mean estimate is not significantly different from zero (*P* < 0.05). Number of effect sizes from top to bottom: All (10, 47, 80, 24, 428, 210, 22), O_3_ (5, 18, 34, 7, 263, 111, 15), NO_*x*_ (12, 14, 12, 31), SO_2_ (4, 17, 25, 108, 68), PM (7, 45). Source data are provided as a Source Data file.

Detritivores, pollinators, and parasitoids were all negatively affected (21–39% reductions in performance) while all herbivorous guilds were either unaffected or responded positively to air pollution (Fig. 3A). These relatively simple predictors (i.e. pest status and feeding guild; see methods and Supplementary Note 3 for detailed classifications) explained 10% and 15% of variation in the response of invertebrates to elevated concentrations of air pollution across the different pollutants tested (marginal *R*^2^ see ref. ^37^; Table 1). Searching efficiency was the most negatively affected aspect of invertebrate performance across all pollutants and was reduced by a third on average when invertebrates were exposed to elevated air pollution treatments (CI = 20–44% reduction, *P* < 0.001; Supplementary Fig. 2A). The diversity of invertebrates was also significantly reduced across all pollutants (*P* = 0.024).

### Effects of individual pollutants

The effects of air pollution on invertebrate performance varied between pollutants, with O_3_ and NO_*x*_ having the greatest negative impacts; reducing the performance of all invertebrates by an average of 10.4% and 11.1%, respectively, in comparison to 1.6% and 18.6% reductions following exposure to SO_2_ and PM. These differences between pollutants increased when considering their divergent impacts on pests and beneficial invertebrates, with O_3_ having the most detrimental impacts on those invertebrates that provide ecological and economic benefits to humans (Fig. 2B–E).

Over half of the available effect sizes measured the response of invertebrates to O_3_ (number of effect sizes: O_3_ = 478, SO_2_ = 245, NO_*x*_ = 96, and PM = 58). Pest status and feeding guild (*R*^2^ = 11% and 17%, respectively; Table 2) were important predictors of invertebrate responses to elevated concentrations of O_3_ (Figs. 2B and 3B). Ozone reduced the performance of beneficial invertebrates by 35% (*P* < 0.001) but had no impact overall on significant pests (*P* = 0.292) or other herbivores (*P* = 0.740). Breaking down the responses by feeding guild, O_3_ reduced the performance of pollinators (*P* < 0.001), parasitoids (*P* = 0.027), and detritivores (*P* = 0.025) by 42%, 28%, and 45%, respectively, while having no significant effect on predators, chewers or cell-feeders. In contrast, the performance of invertebrate borers/miners increased by 55% (Fig. 3B). Less generalizable predictors, including invertebrate families (*R*^2^ = 23%), and the plant species they were associated with during exposure (*R*^2^ = 17%), explained more variation in response to O_3_, but we note that these predictors have 25 and 18 levels, respectively (Table 2). O_3_ pollution affected searching efficiency most negatively, reducing it by 34% compared to control treatments (*P* < 0.001). Ozone pollution also had significant negative effects on invertebrate reproduction (17%, *P* = 0.013) and abundance (14%, *P* = 0.034) (Supplementary Fig. 2B).

The performance of beneficial invertebrates was reduced by an average of 24% (*P* = 0.004) following exposure to NO_*x*_, while significant pests and other herbivores were unaffected (*P* = 0.375 and 0.945, respectively; Fig. 2C). More than 40% of the variation in the response of invertebrates to NO_*x*_ was explained by the plant Order or plant Family they were associated with at the time of exposure (Table 2). Moreover, the experimental method by which invertebrates were exposed to NO_*x*_ also changed the response (*P* = 0.037, df = 4,92; *R*^2^ = 0.27), with the controlled field free-air enrichment (FAE) method tending to show a more negative impact compared with field, laboratory (lab) and open top chamber (OTC) methods (Supplementary Note 4). NO_*x*_ pollution had greater negative effects on invertebrate survival than other aspects of invertebrate performance, reducing survival by 90%, although there was large uncertainty in this estimate (CI = 35–98%, *P* = 0.016). NO_*x*_ pollution also negatively affected searching efficiency, reducing this by 44% on average (*P* = 0.002; Supplementary Fig. 2C).

Pest status had a weak significant effect (*P* = 0.041) on the performance of invertebrates exposed to SO_2_ (Table 2), whereby beneficial invertebrate performance decreased by 21% (*P* = 0.046) compared with significant pests (*P* = 0.130) and other herbivores (*P* = 0.714). In general, responses to SO_2_ were highly variable, making it difficult to explain variation in these responses with generalizable predictors; the predictors with the most explanatory power for SO_2_ impacts were invertebrate Order and Family (*R*^2^ = 23% and 14%, respectively) but these factors have 15 and 18 levels and did not significantly improve model explanatory power (Table 2). SO_2_ had a marginally significant negative effect on the diversity of invertebrates, reducing this by 26% (*P* = 0.043), but did not cause significant changes in other aspects of invertebrate performance (Supplementary Fig. 2D).

We found no evidence that beneficial invertebrates were disproportionately affected by PM (Fig. 2E) or any differences in response between feeding guilds (Fig. 3E; Table 2) but it is important to note the comparatively small number of effect sizes available for PM. Our results indicate that the effects of PM are driven by the plant Order or Family that the invertebrates are associated with (Table 2; values for plant Order and plant Family are identical for PM because these have the same levels).

### Effects of pollutant concentration

**Fig. 4:**
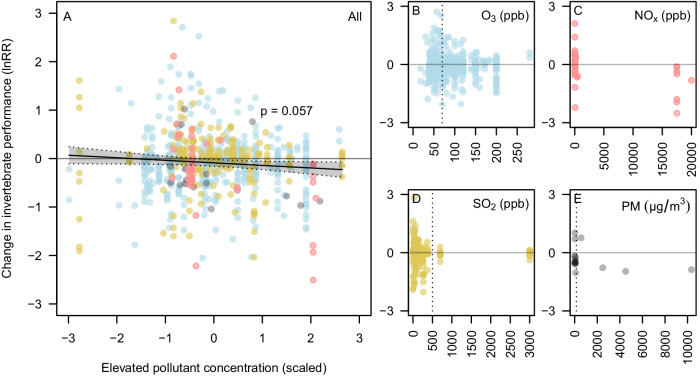
The relationship between air pollutant concentration and changes in invertebrate performance. Panel **A** line shows the relationship between the change in invertebrate performance in control and elevated treatments (log response ratio, ln RR) and the scaled concentration of pollution in the elevated treatment (z-transformed within each pollutant type) from meta-regression model. Shaded area shows a 95% confidence interval and *P*-value is for the slope. **B**–**E** Scatter plots showing the relationship between ln RR and the (unscaled) concentration of elevated pollutant treatment for each pollutant. NO_*x*_ panel combines NO, NO_2_, and NO_*x*_. Dashed vertical lines illustrate the highest National Ambient Air Quality Standards (NAAQS) per pollutant set by the United States Environmental Protection Agency. Levels for NO_*x*_ (NO + NO_2_) are not stipulated, but the NAAQS hourly daily maximum concentrations for NO_2_ is 100 ppb. Source data are provided as a Source Data file.

Air pollution exerted detrimental impacts of a similar magnitude regardless of the concentration to which the pollutants were elevated; we found only a weak and marginally significant relationship between the concentration of elevated pollutant treatments and change in invertebrate performance relative to control conditions (Fig. 4; overall slope across pollutants *P* = 0.057, or 0.045 if imputed ambient concentrations were excluded from 9 studies; Supplementary Note 6). This was maintained when considering the relationships between concentration and effect size separately for significant pests, other herbivores, and beneficial invertebrates (e.g. an interaction term between elevated concentration and pest status, *P* = 0.882, or 0.537 when excluding imputed studies) and for individual pollutants. Likewise, we found no difference in the effect of elevated pollutant concentrations or in the overall effect size between studies that used a filtered air (zero pollution) control and those that used an ambient control (Supplementary Note 5). Many publications did not report information about the concentrations of air pollution tested, with 85 and 107 effect sizes missing information for elevated and control conditions, respectively.

## Discussion

### Oxidizing air pollutants impair beneficial invertebrates

Our study identified disproportionate impacts of O_3_, NO_*x*_, and SO_2_ on those beneficial invertebrates that provide the essential ecosystem services of pollination, pest control and nutrient cycling to human society. The potential threats to food security from these impacts are accentuated because, in contrast, we found no evidence that invertebrate pest species are negatively impacted by these or other air pollutants. Pollination services account for 5–8% (US$235–577 billion in 2015) of the total global value of agricultural food production^38^, and more than 70% of all crop species benefit from pollination by invertebrates^39^. The economic importance of natural pest control (i.e. trophic regulation of pest populations) and nutrient cycling services is more poorly understood, but the former was valued in 2006 at ~$6 billion annually in the US alone^40^. Air pollution has not previously been considered an important driver of declines in beneficial invertebrates, which face a range of environmental pressures (e.g. agricultural intensification, climate change, and introductions of invasive species^41,42^). However, our synthesis of previously disparate evidence implicates air pollution as a significant and overlooked contributor to these declines.

### Air pollution impacts on invertebrate performance are not concentration-dependent

Of significant concern is that even moderate levels of air pollution impaired the performance of beneficial invertebrates; we found only a weak (and marginally significant) relationship between the change in invertebrate performance and the concentration of pollution that was applied in the elevated treatment. We did not identify an effect of concentration when we modeled responses separately for significant pests, other herbivores, and beneficial invertebrates for the different pollutants individually, or when we accounted for the level of pollution in the control (baseline) treatment. This corroborates our previous findings in a field experiment with NO_*x*_ and O_3_, in which we demonstrated significant reductions in flower visitation even with relatively minor increases in pollutant concentration^13^. Beneficial invertebrate populations and the services they provide are, therefore, likely to continue to decline if the current trends of air pollution persist^43^. Any future reductions in NO_*x*_ in urban environments and polluted rural areas (e.g. those next to major roads), as a result of policy changes and shifts away from combustion engine vehicles, may result in increased O_3_ concentrations due to a reduction in O_3_ quenching by NO_*x*_^23,44^. This interaction between pollutants is of concern because our analysis suggests that O_3_ is particularly detrimental to the performance of beneficial invertebrates, and consequently, this is likely to affect the services they provide. While our results provide clear conclusions as to the impacts of individual pollutants applied in experimental settings, there are currently few studies into the effects of co-occurring air pollutants^13,15,32–36^, and how these pollutants interact at the different mixing ratios that could result from current and future emissions scenarios. Regardless of whether the decrease of some pollutants (e.g. NO_*x*_) may exacerbate others (e.g. O_3_) in the short term (see ref. ^45^), all three oxidizing air pollutants impaired the performance of beneficial invertebrates, demonstrating the need to reduce air pollutant concentrations and fossil fuel dependence.

### Mechanistic insights into air pollution-mediated changes in invertebrate groups

We deliberately defined invertebrate performance broadly within our study so as to incorporate different aspects of invertebrate services or disservices, including invertebrate abundance, reproductive rate, feeding efficiency, and searching efficiency. While we found largely consistent impacts across these measures of performance, there were greater negative effects on searching efficiency (the rate at which an invertebrate can locate food and/or host resources), particularly by O_3_ and NO_*x*_ pollution. Similarly, we found that beneficial invertebrates and those feeding guilds with a high dependence upon VOCs for food and host location were particularly impacted by air pollution. Air pollutants can chemically alter VOCs, resulting in reductions in foraging success, as demonstrated in previous modeling-, lab- and field-based studies^13,15,46–48^. They can also modify the biosynthetic pathways of plant secondary metabolites, resulting in changes to VOC emissions by plants^49^. There is also emerging evidence that they can cause physiological changes to invertebrate antennae that reduce their sensitivity to VOC cues^10,11^. The majority of parasitoids and pollinators in our study are aerial invertebrate foragers, which, whilst using a combination of senses to locate food resources, often rely on VOC cues for host and patch location, particularly at greater distances^50^. Parasitoids, particularly hymenopteran wasps that forage for an invertebrate host for offspring to develop in, commonly have significant plasticity in their capacity to learn, memorize, and use VOC cues to locate their herbivore hosts^51^. At the same time, predators, which forage for food, tend to be less sensitive to VOCs than parasitoids^21^, which may explain the differences in impact that we measured between parasitoids and predatory invertebrates.

Disproportionate impacts of air pollution on the performance of different feeding guilds have the potential to alter community structure, with larger scale impacts upon ecosystem service provision than is captured in studies measuring individual species responses. This is particularly the case where species can have multiple roles in a community; we categorized species into feeding guilds, but this can vary with the composition of the community and over time; the larvae of some pollinators are predators (e.g. some hoverfly species) or pests (e.g. some moths and butterflies). Species-level differences, in combination with a scarcity of data, may explain why we found no significant impacts of NO_*x*_ and SO_2_ on natural enemies. A recent field study^36^ demonstrated that NO_*x*_-mediated effects on parasitoid attraction to plant-released volatiles can be Family- or even species-specific. Some species may be better able to offset air pollution-mediated disruption to navigation than others; for example, the nocturnal pollinator *Manduca sexta* can learn to associate air pollution-altered VOCs with their floral nectar resource^52^. Previous reviews and meta-analysis studies have demonstrated no effects^16–19^ or positive effects of air pollution on the performance of herbivores, especially aphids^3,20,21^. In our study, the performance of boring/mining herbivores increased under air pollution (O_3_ in particular), likely because they are able to exploit stress-induced increases in plant metabolites or decreases in plant resistance^32,53,54^, but we found no overall effect, and significant variation in the performance of the two most abundant feeding guilds: cell-feeding and tissue-chewing herbivores. These classifications incorporate a broad range of species and a non-significant impact at feeding guild scale could be masking species-scale impacts and corresponding changes in community structure due to air pollution. Responses of herbivores to air pollution are known to be complex and highly variable, for example, air pollution can result in stress-related increases of plant secondary metabolites, which herbivores may take advantage of^55,56^ or be impaired by^3^.

### Invertebrate responses to PM are uncertain

Literature on the effects of airborne PM on invertebrates was particularly scarce compared with studies using O_3_, NO_*x*_, and SO_2_. Airborne PM is often associated with other pollutants (e.g. NO_*x*_), and its chemical components, size, and spatial distribution depend on the source of the particles, making it challenging to quantify^57^. Airborne PM can result in direct negative consequences on pollinator learning, memory and survival^8,10^ and recent studies have indicated that PM deposition on the surface of plants negatively affects the feeding efficiency of tissue-chewing herbivores, which may be dependent on the type and quantity of PM accumulation on the plant surface^58,59^. Geographically, studies with PM are focused in Australia but are generally lacking, and there is a scarcity of studies on all pollutants across Africa, SE Asia, and South America (as indicated in Fig. 1). We, therefore, advocate for air pollution studies in these regions to gain a more comprehensive global understanding of how air pollutants, especially PM, affect insect populations.

### Summary

Our findings indicate that air pollutants disproportionately impair the performance of beneficial invertebrates. These negative impacts appear to stem from the disruption of VOC-mediated food or host location by these species. The impacts of air pollution do not appear to vary with pollutant concentration; even moderate levels of O_3_ and NO_*x*_ adversely affect beneficial invertebrates, which indicates that the threats posed by air pollution are likely to remain or worsen without particularly severe and draconian changes to policy. Ozone pollution, in particular, appears to be a significant concern. Therefore, while the results of this analysis provide further evidence that reducing emissions of all air pollutants should be a priority, they indicate that an increased focus on reducing, or at least restricting, increases in ozone could be particularly advantageous for beneficial invertebrate species. Likewise, our evidence suggests that air pollution detrimentally impacts pollinators. Air pollution-mediated reductions in flower visitation by pollinators are likely to result in a higher proportion of economic losses than is currently predicted, especially if O_3_ levels continue to increase unabated^13,60^. As such, our results demonstrate that air pollution needs to be carefully considered alongside other threats in management plans and policies aiming to safeguard these beneficial invertebrates.

## Methods

### Study selection and classification of predictors

We searched Web of Science (all databases) following the approach by Bishop and Nakagawa^61^ to identify relevant publications for our meta-analysis, using the terms ‘air pollution’ ‘terrestrial invertebrate/insect/arthropod’ in combination with terms indicative of the four individual air pollutants (NO_*x*_, O_3_, SO_2_, and PM). See Supplementary Note 1 for a full list of search terms used. Our search includes articles published on or before 10 November 2022. We screened the 1446 unduplicated records by title and abstract and identified 231 publications of potential relevance (1215 studies did not include a measure of invertebrate performance and/or the target pollutants). Our criteria for inclusion in the analysis was that the publication must present a measure of invertebrate performance in either ambient air pollution conditions or a control air pollution treatment, and at elevated air pollution conditions or in an elevated air pollution treatment. A total of 120 publications (Supplementary Data 1) met our inclusion criteria. Further details are provided in a PRISMA diagram (Supplementary Fig. 1).

Where possible, we extracted pollutant concentrations for the control and elevated air pollution treatments. Several semi-field (i.e. open-top chamber or free-air enrichment) and field studies reported an elevated concentration but no control pollutant concentration (*N* = 5 publications and 34 effect sizes for O_3_, 4 publications, and 21 effect sizes for SO_2_). We imputed these values using the mean control concentration across all other field studies (30 ppb O_3_ and 10 ppb SO_2_). We extracted numerical data from graphical figures using WebPlotDigitizer.

The 120 publications that satisfied our inclusion criteria for the meta-analysis included a total of 877 effect sizes; some studies tested responses to more than one air pollutant or measured several aspects of invertebrate performance. While some data here are available for combined pollutants (25 effect sizes from 5 studies for interactions between O_3_ and other pollutants, 10 effect sizes from 1 study for interactions not involving O_3_), there were too few to include them in the analysis, so we included only effect sizes where pollutants were applied individually. We extracted the mean values, standard deviations (SD), and sample sizes (*N*) for each effect size comparing invertebrate performance between the two air pollution treatments. We extracted data for both direct and indirect (e.g. plant-mediated) invertebrate performance responses, which are often challenging to disentangle from one another^62^. Performance metrics included abundance, feeding efficiency, growth/development, reproduction, searching efficiency, survival, and diversity (see Supplementary Note 6 for testing suitability as a proxy for invertebrate population performance). Where studies did not report SDs but presented data for more than one comparison (e.g. multiple genotypes or multiple years), a single value was obtained by aggregating raw data at the largest scale to avoid nonindependence, as in ref. ^63^. Where SDs were missing and not able to be calculated by combining multiple data points (*N* = 1 publication, 4 effect sizes), they were imputed by averaging those from other effect sizes from the same performance metric, invertebrate Family and air pollutant^64^.

#### Defining categories

Significant pests were defined as invertebrate species that were listed in the Centre for Agriculture and Bioscience International (CABI) Distribution Maps of Plant Pests (DMPP) and/or the European and Mediterranean Plant Protection Organization (EPPO) Alert (A1) list or Pest Risk Analyses (PRA) databases. Other herbivores include non-beneficial invertebrates that were not defined in these CABI or EPPO databases but include plant pests that are non-commercially important to food and commodity crops, as well as minor pests and non-pest herbivores. The most common Families of both ‘significant pests’ and ‘other herbivores’ were Aphididae and Chrysomelidae. Invertebrate feeding guilds were defined based on the predominant mode of feeding for the specific life stage recorded. The majority of beneficial invertebrates were nectar or pollen feeders (classified as pollinators for brevity) but also included the feeding guilds ‘predators’, ‘parasitic wasps (i.e. parasitoids)’ and ‘detritivores’ (see Supplementary Note 3 for details). The most common Families of beneficial invertebrates were Apidae and Braconidae. Any species that undergoes outbreaks that are known to result in significant economic or ecological damage at some stage in their life cycle (according to CABI’s DMPP and/or EPPO’s A1/RDA databases) were considered a significant pest for the purposes of this study. As such, in two studies, adult moths (*Plutella xylostella* and *Manduca sexta*) that were nectar- and/or pollen-feeders but were listed in the CABI and/or EPPO databases were defined as significant pests.

### Calculating effect sizes

To quantify the effect of air pollution on invertebrate performance (the effect size), we used the natural log of the response ratio (ln RR), which is the log proportional change in performance between invertebrates exposed to elevated pollution and ambient or control conditions. This converts to the percentage loss or increase in invertebrate performance in elevated air pollution using the formula 1−exp(ln RR)*100 for negative values and exp(ln RR)−1*100 for positive values, respectively.

### Multi-level meta-analysis models

#### Random effects

Effect sizes and sampling variances from the same publication and country are likely to be correlated (clustered), which invalidates model assumptions of independence^65^. We used multi-level meta-analytic models with random effects and variance–covariance (VCV) matrices to account for the dependence of effect sizes and sampling variances, respectively, the latter specifically resulting from effect sizes that shared a common control treatment^61,63^. We identified the optimal random effects structure by comparing the Akaike Information Criteria (AIC) of different candidate models. These candidate models included all 877 effect sizes across all air pollutant treatments. The random effects tested were an individual effect size identifier (infoID; unique per effect size, necessary to estimate residual heterogeneity), a publication identifier (studyID; year nested within the publication), country (i.e. country in which experiments were reported in a publication were conducted), extractor (the person who initially extracted each datapoint, each of which was checked by an alternative extractor) and a multiple outcome cluster identifier (indicating where more than one performance metric is reported for the same individual or group of invertebrates for each study). The optimal random effects structure contained identifiers for individual effect sizes, multiple outcome clusters, and publications; for further details and R code, refer to Supplementary Note 2.

#### Fixed effects (moderators)

We compared uni-moderator models to the optimal random effect model to determine which variables or functional groups explained the most variation in air pollution-mediated changes in invertebrate performance. We first did this on the whole dataset, including all pollutants (Table 1), and then on separate datasets for each pollutant type (O_3_, NO_*x*_, SO_2_, and PM; Table 2) to explore whether impacts varied between pollutants. We conducted these analyses separately rather than modeling interaction terms between moderators and pollutant types in the overall model because there were many missing levels for different moderators within individual pollutant types (e.g. Fig. 3). We assessed the explanatory power of each moderator by conducting a likelihood ratio test (LRT) comparing a candidate model containing a single moderator to a (null) model containing only random effects. The number of effect sizes included in these models varied between moderators, because several moderators contained missing values for some effect sizes where we were unable to categorize them when extracting the data from publications. We ranked the moderators by the LRT *P*-value to determine which had the greatest explanatory power. If *P* > 0.05, we concluded that the moderator did not explain variation in the air-pollution-mediated changes in invertebrate performance^66^. The 13 moderators we tested (based on previous classifications^30,32^) are reported in Table 1 and Supplementary Note 3. We conducted all model comparisons using models fit with maximum likelihood (ML), while we report model estimates in the manuscript from models fitted with restricted maximum likelihood (REML).

#### Associations of pollutant concentration and invertebrate performance

Multi-level meta-analysis models with the same error structure were used to determine how the effect of air pollution on invertebrate performance varies with the concentration of the pollutants. This was determined for studies reporting concentrations of NO_*x*_ (i.e. NO + NO_2_, reported if the paper did not differentiate between NO and NO_2_; *N* = 10), NO_2_ only (reported if the paper did not also report NO; *N* = 10), O_3_ (*N* = 74) and SO_2_ (*N* = 28). We converted the concentration of elevated pollution treatments to a common scale, first by log-transforming them, and then by scaling them within each pollutant type using z-scores. We tested this scaled concentration of elevated pollution treatment as a moderator across all pollutants. We tested whether this relationship varied between pollutant types (interaction between scaled concentration and pollutant type) and between significant pests, other herbivores, and beneficial invertebrates (interaction between scaled concentration and pest status). We also tested whether the air pollutant concentration in the control treatment, which was typically either filtered air (0 ppb for NO_*x*_, O_3_, and SO_2_, and 0 µg/m^3^ for PM) or ambient concentration, explained variation in the effect size. Please see the ‘defining categories’ section above for a full definition of the three pest status levels.

### Publication bias and sensitivity analysis

Publication bias was assessed using a funnel plot from a model including the two most significant moderators^67^ and funnel asymmetry was tested using Egger’s regression^68^. A significant slope for standard error would indicate statistically significant funnel asymmetry after controlling for all other variables in the model. In the absence of bias, the funnel plot forms a symmetrical inverted funnel centered on the mean effect. Using these methods, we did not identify evidence of publication bias (*z* = −1.49, *P* = 0.135; Supplementary Fig. 3).

Comparisons between effect sizes and year of publication were made to determine the presence of any time-lag bias (i.e. a change in the magnitude of the effect over time) by including ‘year’ as a moderator in multi-level models^69^. The negative effects of air pollution have become more pronounced over time across all pollutants, (Supplementary Fig. 4), apparently driven by significant reductions in performance over time with O_3_ and NO_*x*_ pollution (Supplementary Fig. 5A and B; Supplementary Note 6).

Leave-one-out analysis was also used to determine whether our mean effect size estimates were robust to the exclusion of individual publications. Our sensitivity analyses indicate that our results were not unduly influenced by findings of individual experiments; the effects of air pollutants on significant pests, other herbivores, and beneficial invertebrates were stable when we excluded the results of each study individually in leave-one-out analyses (Supplementary Figs. 6–8). Alluvial plots were used to visualize the degree of overlap between categories (i.e. within-moderator levels) for different pairs of moderators (Supplementary Fig. 9).

### Reporting summary

Further information on research design is available in the Nature Portfolio Reporting Summary linked to this article.

### Supplementary information

Supplementary Information

Peer Review File

Description of Additional Supplementary Files

Supplementary Data 1

Reporting Summary

### Source data

Source Data

## Data Availability

The data that support the findings of this study are available in Supplementary Data 1. Source data for figures generated in this study can be found in the Source Data file. Source data are provided with this paper.

## Code availability

The code for data analysis that supports the findings of this study is integrated into the Supplementary Information file and is available from Open Science Framework^70^ (10.17605/OSF.IO/UEQP7).
