# Supplementary material for: Air pollution disproportionately impairs beneficial invertebrates: a meta-analysis
Source: Nat Commun. 2024 Jul 11;15:5447. doi: 10.1038/s41467-024-49729-5 (PMC11239652; doi:10.1038/s41467-024-49729-5)
Supplement: Supplementary file 1 — Supplementary Information [file 41467_2024_49729_MOESM1_ESM.pdf]

Supplementary Information - Air pollution disproportionately impairs beneficial invertebrates: a meta-analysis

James M.W. Ryalls, Jacob Bishop, Adedayo O. Mofikoya, Lisa M. Bromfield, Shinichi Nakagawa and Robbie D. Girling

June 2024

Contents

Supplementary Note 1: Introduction and literature search **2**

Identifying literature 2

Supplementary Fig. 1 3

Preparation 3

Figure 1 5

Supplementary Note 2: Preparing data and model structure for analysis **5**

Imputed standard deviation 5

Calculating effect sizes for metadat dataframe 6

Testing random effects 6

Supplementary Note 3: Multilevel models - all pollutants **7**

How does response to pollution vary between our different measures of performance? 8

What are the simplest groupings or functional characteristics that explain variation in the impact of air pollution on invertebrate performance? 9

Null models for individual pollutants 31

Supplementary Note 4: Multilevel models - individual pollutants **33**

Method 33

Invertebrate Order 38

Invertebrate Family 45

Lifestage 52

Winged 57

Diet specialisation 62

Response Measure 66

Supplementary Fig. 2 72

Plant Order 72
Plant Family 78
Annuality 84
Plant type 89
4.1. **Pest status: are pests or beneficial invertebrates more affected by air pollution?** . 94
Status - O3 94
Status - NOx 96
Status - SO2 98
Status - PM 99
Figure 2 100
4.2. **Feeding guilds: which feeding guilds are most affected by air pollution?** 100
Feeding guild - O3 102
Feeding guild - NOx 104
Feeding guild - SO2 106
Feeding guild - PM 107
Figure 3 109

Supplementary Note 5: Air pollutant concentrations **109**
Figure 4 114

Supplementary Note 6: Publication bias and sensitivity analysis **114**
Supplementary Fig. 3 114
Supplementary Fig. 4 117
Supplementary Fig. 5 120
Supplementary Fig. 6 124
Supplementary Fig. 7 126
Supplementary Fig. 8 127
Supplementary Fig. 9 137

R Session Information **140**

Supplementary References **141**

Supplementary Note 1: Introduction and literature search

Identifying literature

Three people (JMWR, AM and LMB) conducted the literature search and made all decisions regarding which studies to include or exclude. Two people (JMWR and AM) extracted the data and all studies were checked by each extractor.

Supplementary Fig. 1

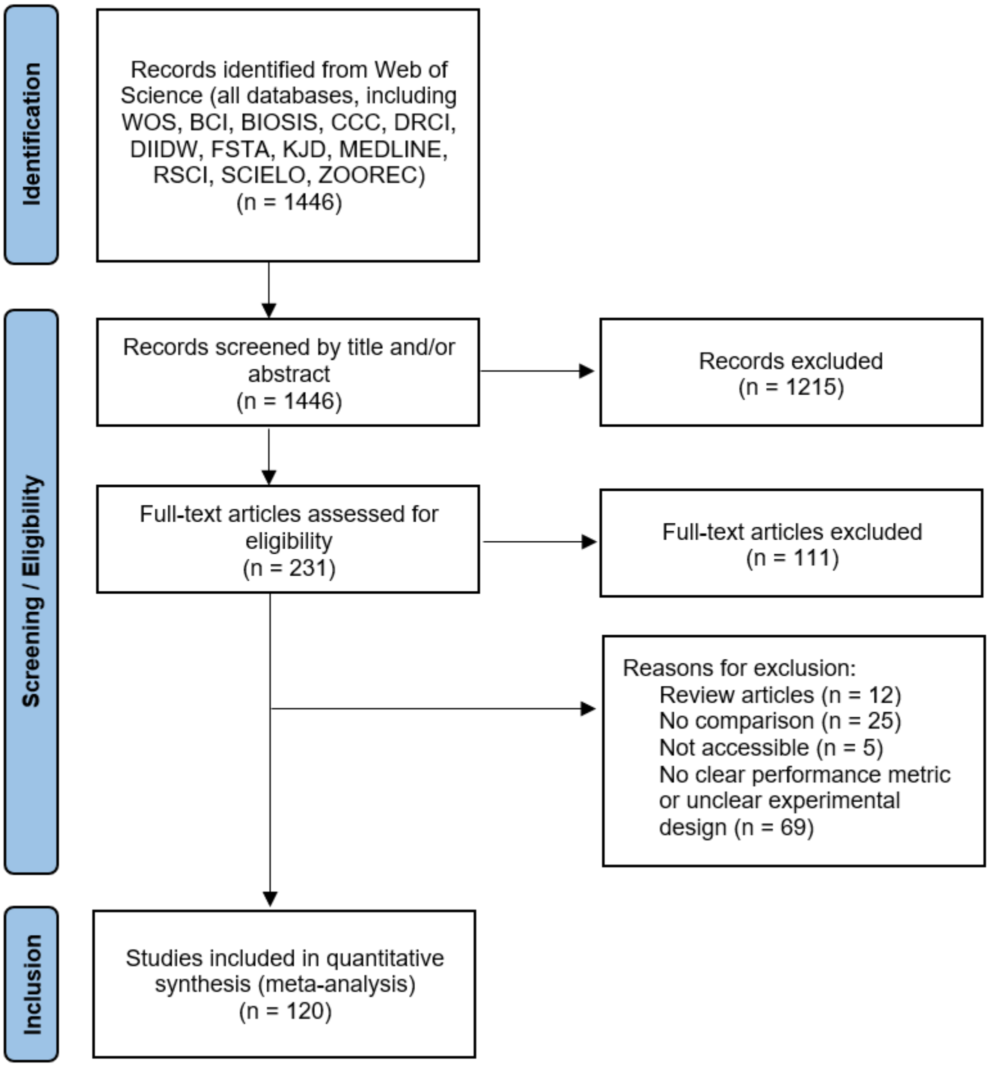

Supplementary Fig. 1. PRISMA (Preferred Reporting Items for Systematic Reviews and Meta-Analyses) diagram illustrating our study selection process

Search terms: Topic = air pollution AND (terrestrial (invertebrate OR insect OR arthropod)) AND (sulphur dioxide OR SO2 OR diesel OR nitrogen oxide OR atmospheric nitrogen OR NO2 OR ozone OR O3 OR particulate matter OR PM)

Preparation

First we install and/or load necessary packages

```
library(metaAidR)
library(orchaRd)
library(metafor)
library(devtools)
library(tidyverse)
```

```r
library(patchwork)
library(R.rsp)
library(tibble)
library(cowplot)
library(metafor)
library(corpcor)
library(here)
library(MCMCglmm)
library(rworldmap)
library(ggplot2)
library(patchwork)
library(formatR)
library(ggalluvial)
library(ggpubr)
```

Then we load some additional functions that we prepared

```r
source(here("R/functions.R"), chdir = TRUE)
```

Load the data

```r
metadat <- read.csv(here("data/Metatable_v2.csv"), header = T,
    na.strings = c("", "NA"))
```

Change the relevant variables to factors and remove the small number of effect sizes measuring responses to combined pollutants

```r
metadat$studyid <- as.factor(metadat$studyid)
metadat$Pollutant <- as.factor(metadat$Pollutant)
metadat$Pollutant2 <- as.factor(metadat$Pollutant2)
metadat$Pollutant3 <- as.factor(metadat$Pollutant3)
metadat$Performance <- as.factor(metadat$Performance)
metadat$Performance2 <- as.factor(metadat$Performance2)
metadat$Location <- as.factor(metadat$Location)
metadat$Invert.Order <- as.factor(metadat$Invert.Order)
metadat$Invert.Family <- as.factor(metadat$Invert.Family)
metadat$Invert.species.or.subfamily <- as.factor(metadat$Invert.species.or.subfamily)
metadat$feeding.guild <- as.factor(metadat$feeding.guild)
metadat$Lifestage <- as.factor(metadat$Lifestage)
metadat$Winged <- as.factor(metadat$Winged)
metadat$Diet.specialisation <- as.factor(metadat$Diet.specialisation)
metadat$Plant.Order <- as.factor(metadat$Plant.Order)
metadat$Plant.Family <- as.factor(metadat$Plant.Family)
metadat$Plant.Species <- as.factor(metadat$Plant.Species)
metadat$Annuality <- as.factor(metadat$Annuality)
metadat$Monocot_Dicot <- as.factor(metadat$Monocot_Dicot)
metadat$Status <- as.factor(metadat$Status)
str(metadat)
summary(metadat$Pollutant3)
# subset to remove O3xCO2 interaction
metadat <- droplevels(subset(metadat, Pollutant3 != "O3*CO2"))
```

We chose to remove the effect sizes where pollutants were applied in combination (a total of 35 effect sizes from 6 studies) due to the small number of replicates for these treatments which included various combinations of pollutants. Only effect sizes where one of our 4 focal pollutants were applied are included in subsequent models.

```
# 4P subset
metadat_4P <- droplevels(subset(metadat, Pollutant2 == "O3" |
    Pollutant2 == "NOx" | Pollutant2 == "PM" | Pollutant2 ==
    "SO2"))
# Rerun VCV matrix
metadat_4P$cV.lnRR <- with(metadat_4P, cV.lnRR(CMean = Xc, CSD = Dev_c,
    CN = Nc))
```

How many publications do we have? For reporting in MS

```
length(levels(factor(metadat_4P$studyid)))
```

```
## [1] 120
```

How many datapoints do we have? For reporting in MS

```
length(levels(factor(metadat_4P$infoid)))
```

```
## [1] 877
```

How many countries do our effect sizes come from and which countries are represented?

```
levels(factor(metadat_4P$Country))
```

```
##  [1] "Argentina"   "Australia"   "Canada"      "China"       "Finland"
##  [6] "France"      "Germany"     "Hungary"     "India"       "Italy"
## [11] "Japan"       "Norway"      "Pakistan"    "Poland"      "Russia"
## [16] "Sweden"      "Switzerland" "UK"          "USA"
```

Figure 1

See main text for Figure 1

Supplementary Note 2: Preparing data and model structure for analysis

Imputed standard deviation

One of the 120 publications ([1]Dohmen et al. 1984; Table S1) our main analyses are based upon did not report standard deviation (SD) or a convertible alternative (e.g. standard error). Rather than completely removing the effect sizes for this publication and losing important information, we imputed the standard deviation by taking a mean of the SD from all responses that use the same performance metric under the same pollution scenario for the same invertebrate family.

Calculating effect sizes for metadat dataframe

For our analyses we use lnRR (log response ratio), which can be readily back-transformed to percentage change in invertebrate performance, using (1-exp(lnRR))100 for negative values and (exp(lnRR)-1)100 for positive values. We use new estimators of this effect size to account for differences in sample size between the two groups ([2]Senior et al. 2020; https://doi.org/10.1002/jrsm.1423). While calculating our effect size measure we also calculate a variance co-variance matrix which allows us to account for dependence (correlation of sampling errors) that results from treatment effects being compared to a shared control (e.g. where two different air pollution treatments are applied, and compared to the same control group).

```
# lnRR2 and cV.lnRR are functions we make in the functions.R script
metadat_4P$lnrr.yi <- with(metadat_4P,lnRR2(CMean=Xc,
                                  CSD=Dev_c,
                                  CN=Nc,
                                  EMean=Xe,
                                  ESD=Dev_e,
                                  EN=Ne))[,1]
metadat_4P$lnrr.vi <- with(metadat_4P,lnRR2(CMean=Xc,
                                  CSD=Dev_c,
                                  CN=Nc,
                                  EMean=Xe,
                                  ESD=Dev_e,
                                  EN=Ne))[,2]
# we remove data rows that are NaN's
metadat_4P <- droplevels(subset(metadat_4P,!(is.na(lnrr.yi))))
# adding control variance for shared control VCV
metadat_4P$cV.lnRR <- with(metadat_4P,cV.lnRR(CMean=Xc,
                                    CSD=Dev_c,
                                    CN=Nc))

# shared control = true
V.d2.shared.lnRR_4P <- VCV.shared(data=metadat_4P,
                               cV="cV.lnRR", #covariance
                               V = "lnrr.vi", #variance
                               cluster="sharedcontrolcluster", #clustering factor
                               obs="infoid") # each data row has a unique infoid
#is.positive.definite(V.d2.shared.lnRR_4P) # check the VCV is made correctly
```

Testing random effects

Due to the nature of the experiments from which our effect sizes are taken, there are several different ways in which the effect sizes could be clustered; for example, means could be more similar from experiments conducted in the same country, due to similarity in methodology. To explore this and to then model and account for these different levels of clustering, we test different candidate random effects structures in multi-level models. We always include the random effects ‘infoid’ and ‘studyid’ to estimate the residual heterogeneity (e.g. unexplained variation) and to account for dependence between effect sizes from the same publication, respectively. We test other random effects to determine whether they are important sources of heterogeneity using AIC.

Random effects: Variables marked with asterisk were used in the final models and are reported in the paper.

- infoid* - Unique identifier per effect size (row in dataset) to model the residual heterogeneity.

- studyid* - Unique identifier per publication.
- Country - Unique identifier per country in which the experiments reported in a publication were conducted.
- multipleoutcomecluster* - Unique identifier per experimental unit Where more than one effect size is reported for the same individual or group (e.g. one publication reports pupal mass and development time from the same experimental unit). Note that including this random effect controls for correlation of the effect sizes, but not of their sampling errors which may also also be correlated. We are already using a variance covariance matrix to account for correlation of sampling errors due to shared control values.

```
# Null model with vcv matrix:
RE_vcv <- rma.mv(yi = lnrr.yi, V = V.d2.shared.lnRR_4P, random = list(~1 |
    studyid, ~1 | infoid), data = metadat_4P, method = "ML")
summary(RE_vcv)  # AIC 1146.4065

# Test whether country should be a random effect:
RE1.vcv <- rma.mv(yi = lnrr.yi, V = V.d2.shared.lnRR_4P, random = list(~1 |
    studyid, ~1 | infoid, ~1 | Country), data = metadat_4P, method = "ML")
summary(RE1.vcv)  #AIC higher 1148.1136 therefore not included

# Test whether extractor should be a random effect:
RE2.vcv <- rma.mv(yi = lnrr.yi, V = V.d2.shared.lnRR_4P, random = list(~1 |
    studyid, ~1 | infoid, ~1 | Extractor), data = metadat_4P,
    method = "ML")
summary(RE2.vcv)  #AIC higher 1148.4065 therefore not included

# Test whether multiple outcomes cluster should be a random
# effect:
RE3.vcv <- rma.mv(yi = lnrr.yi, V = V.d2.shared.lnRR_4P, random = list(~1 |
    studyid, ~1 | infoid, ~1 | multipleoutcomecluster), data = metadat_4P,
    method = "ML")
summary(RE3.vcv)  #AIC lower 1125.0844 therefore included
# this is our null model for all pollutants

# refit with REML for reporting in MS
RE3.vcv.reml <- rma.mv(yi = lnrr.yi, V = V.d2.shared.lnRR_4P,
    random = list(~1 | studyid, ~1 | infoid, ~1 | multipleoutcomecluster),
    data = metadat_4P, method = "REML")
summary(RE3.vcv.reml)

# quantify the heterogeneity in effect size
i2_ml(RE3.vcv.reml)
```

Supplementary Note 3: Multilevel models - all pollutants

Our first step to explain variation in responses of invertebrates to air pollution was to use the full dataset (e.g. across all pollutant types) and use moderators to try to explain variation in effect sizes.

How does response to pollution vary between our different measures of performance?

We compared effect sizes between our different measures of performance. In our model including all pollutant types, lnRR was significantly different from zero for searching efficiency (p < 0.001) and diversity (p = 0.02) performance metrics.

```
perf <- rma.mv(yi = lnrr.yi, V = V.d2.shared.lnRR_4P, mods = ~Performance2 -
    1, random = list(~1 | studyid, ~1 | infoid, ~1 | multipleoutcomecluster),
    data = metadat_4P, method = "ML")
# summary(perf)

# likelihood ratio test to compare to null model without
# performance moderator
anova(perf, RE3.vcv)
```

```
##
##          df       AIC       BIC      AICc    logLik     LRT   pval        QE
## Full     10 1116.3348 1164.0999 1116.5889 -548.1674                7991.8339
## Reduced   4 1125.0844 1144.1904 1125.1302 -558.5422 20.7495 0.0020 8758.6680
```

```
perf.reml <- rma.mv(yi = lnrr.yi, V = V.d2.shared.lnRR_4P, mods = ~Performance2 -
    1, random = list(~1 | studyid, ~1 | infoid, ~1 | multipleoutcomecluster),
    data = metadat_4P, method = "REML")
summary(perf.reml)
```

```
##
## Multivariate Meta-Analysis Model (k = 877; method: REML)
##
##    logLik   Deviance        AIC        BIC       AICc
## -545.6681  1091.3362  1111.3362  1159.0211  1111.5923
##
## Variance Components:
##
##             estim    sqrt  nlvls  fixed                  factor
## sigma^2.1  0.0863  0.2938    120     no                 studyid
## sigma^2.2  0.0348  0.1866    875     no                  infoid
## sigma^2.3  0.0665  0.2578    562     no  multipleoutcomecluster
##
## Test for Residual Heterogeneity:
## QE(df = 870) = 7991.8339, p-val < .0001
##
## Test of Moderators (coefficients 1:7):
## QM(df = 7) = 28.9016, p-val = 0.0002
##
## Model Results:
##
##                                    estimate      se     zval    pval    ci.lb
## Performance2abundance               -0.0550  0.0555  -0.9898  0.3223  -0.1638
## Performance2diversity               -0.2319  0.1026  -2.2591  0.0239  -0.4330
## Performance2feeding efficiency       0.0044  0.0485   0.0909  0.9276  -0.0907
## Performance2growth/development      -0.0587  0.0451  -1.3011  0.1932  -0.1472
## Performance2reproduction            -0.0973  0.0575  -1.6941  0.0903  -0.2100
```

```
## Performance2searching efficiency   -0.4015  0.0915  -4.3880  <.0001  -0.5808
## Performance2survival              -0.0526  0.0596  -0.8817  0.3780  -0.1695
##                                     ci.ub
## Performance2abundance              0.0539
## Performance2diversity             -0.0307    *
## Performance2feeding efficiency     0.0996
## Performance2growth/development     0.0297
## Performance2reproduction           0.0153    .
## Performance2searching efficiency  -0.2221  ***
## Performance2survival               0.0643
##
## ---
## Signif. codes:  0 '***' 0.001 '**' 0.01 '*' 0.05 '.' 0.1 ' ' 1
```

```r
# to what extent is searching efficiency confounded with
# other categories? Such as whether the invertebrate is
# classed as a pest or a beneficial (see below)
with(metadat_4P, aggregate(lnrr.yi, by = list(Performance2, Status),
    length))
```

```
##                 Group.1          Group.2   x
## 1             abundance       Beneficial  76
## 2             diversity       Beneficial   9
## 3    feeding efficiency       Beneficial   9
## 4    growth/development       Beneficial   4
## 5          reproduction       Beneficial   4
## 6  searching efficiency       Beneficial  52
## 7              survival       Beneficial  12
## 8             abundance  Other herbivore  62
## 9             diversity  Other herbivore  14
## 10   feeding efficiency  Other herbivore  51
## 11   growth/development  Other herbivore  88
## 12         reproduction  Other herbivore  29
## 13 searching efficiency  Other herbivore   6
## 14             survival  Other herbivore  32
## 15            abundance Significant pest  59
## 16   feeding efficiency Significant pest 104
## 17   growth/development Significant pest 147
## 18         reproduction Significant pest  64
## 19 searching efficiency Significant pest  11
## 20             survival Significant pest  30
```

```r
# it is not confounded, there are 11 effect sizes for
# searching efficiency for significant pests (of 415), 6
# effect sizes for Other herbivores (of 282) and there are
# 52 for beneficials (of 166).
```

What are the simplest groupings or functional characteristics that explain variation in the impact of air pollution on invertebrate performance?

We used individual models containing single moderators (uni-moderator models) to compare their explanatory power. In each case we compared these uni-moderator models to a null model containing no moderators

using a likelihood ratio test. We often needed to run these models on different datasets, each time excluding NA values where we had been unable to attribute an effect size to a particular level of the moderator; we made corresponding nested null models in each case for the likelihood ratio test. We found that the moderators with the greatest explanatory power were pest status ($P < 0.0001$) and feeding guild ($P < 0.0001$). See Table 1 in the main manuscript for a summary of model outputs.

The 13 moderators tested were as follows:

- **Performance** - summarized in the model above - this included metrics applied to individual species, populations and communities of invertebrates and included measures of abundance (e.g. population density; N = 39 studies, 207 effect sizes), diversity (e.g. Shannon diversity; N = 6 studies, 27 effect sizes), feeding efficiency (e.g. consumption rate; N = 35 studies, 164 effect sizes), growth/development (e.g. relative growth rate; N = 41 studies, 239 effect sizes), reproduction (e.g. number of offspring; N = 28 studies, 97 effect sizes), searching efficiency (e.g. foraging time; N = 16 studies, 69 effect sizes) and survival (e.g. emergence success; N = 19 studies, 74 effect sizes).
- **Pest status** - this includes significant pests (N = 62 studies, 415 effect sizes, classified using CABI/EPPO databases, as defined in the text; common families include Aphididae, Chrysomelidae and Erebidae), other herbivores (N = 39 studies, 282 effect sizes; this non-pest herbivores and minor pests; common families include Aphididae and Chrysomelidae) and beneficial invertebrates (N = 31 studies, 166 effect sizes; common families include Apidae and Braconidae).
- **Feeding guild** - this refers to the feeding strategies that invertebrates employ and included 7 categories, as follows: Boring or mining herbivores (N = 7 studies, 22 effect sizes; the most common family is Curculionidae), cell-feeding herbivores (N = 34 studies, 210 effect sizes, includes fluid-feeders or phloem-feeders, such as aphids; the most common family is Aphididae), leaf-chewing herbivores (N = 51 studies, 428 effect sizes; the most common family is Chrysomelidae), detritivorous herbivores (i.e. decomposers, N = 5 studies, 24 effect sizes; the most common family is Drosophilidae), nectar or pollen-feeding insects (classified as pollinators for brevity, N = 17 studies, 80 effect sizes; the most common family is Apidae), parasitic wasps (i.e. parasitoids, N = 9 studies, 47 effect sizes; the most common family is Braconidae) and predatory insects (N = 4 studies, 10 effect sizes; Families are undefined because there were multiple predators recorded per study. These were all classified as beneficial invertebrates for the purpose of this meta-analysis based on their dominant prey).
- **Invertebrate Order** - see Table 1 or model summary below for a full list.
- **Invertebrate Family** - see model summary below for a full list.
- **Lifestage** - whether the invertebrate lifecycle stage recorded was adult, egg, larva, nymph, pupae or multiple lifestages. The majority of studies recorded adult invertebrates (N = 49), followed by larvae (N = 37).
- **Winged** - whether the invertebrates recorded were winged (yes, N = 47 studies) or not winged (no, N = 42 studies). Many invertebrates (e.g. many aphid population performance measures) were recorded as both winged and not winged (both, N = 19 studies).
- **Diet specialisation** - where available, species that were generalists (i.e. tend to feed on a variety of orders or families; N = 51 studies) and specialists (tend to feed on one or two families; N = 48 studies).
- **Plant Order** - where available, the plant associated with the invertebrate was recorded - see Table 1 or model summary below for a full list of plant orders.
- **Plant Family** - see model summary below for a full list.
- **Annuality** - whether the plant species associated with the invertebrate was an annual (completes its life cycle within a single growing season or year; N = 37 studies), biennial (life cycle spans two growing seasons or years; N = 3 studies) or perennial (persist for many years; N = 58 studies).
- **Plant Type** - whether the plant is a monocot (flowering plant that has seeds with only one cotyledon in their embryo; N = 10 studies) , dicot (flowering plant with seeds that have two cotyledons in their embryo; N = 73 studies) or gymnosperm (seed-producing plants that do not produce flowers; N = 16 studies).
- **Method** - the experimental method used in the study, includes controlled free-air enrichment (FAE; N = 22 studies), field (N = 23 studies), laboratory (lab; N = 59 studies) and controlled open top chamber (OTC) experiments (N = 18 studies).

METHOD

```r
# str(metadat_4P)
metadat_Location <- droplevels(subset(metadat_4P, !(is.na(Location))))
# rerun VCV matrix
V.d2.shared.lnRR_Location <- VCV.shared(data = metadat_Location,
    cV = "cV.lnRR", V = "lnrr.vi", cluster = "sharedcontrolcluster",
    obs = "infoid")
## is.positive.definite(V.d2.shared.lnRR_Location) # check
## the VCV is made correctly

Q1B.null.Location <- rma.mv(yi = lnrr.yi, V = V.d2.shared.lnRR_Location,
    random = list(~1 | studyid, ~1 | infoid, ~1 | multipleoutcomecluster),
    data = metadat_Location, method = "ML")
Q1B.Location <- rma.mv(yi = lnrr.yi, V = V.d2.shared.lnRR_Location,
    mods = ~Location - 1, random = list(~1 | studyid, ~1 | infoid,
        ~1 | multipleoutcomecluster), data = metadat_Location,
    method = "ML")
anova(Q1B.null.Location, Q1B.Location)
```

```
##
##          df       AIC       BIC      AICc    logLik    LRT   pval        QE
## Full      7 1130.3090 1163.7446 1130.4379 -558.1545               8379.1220
## Reduced   4 1125.0844 1144.1904 1125.1302 -558.5422 0.7753 0.8554 8758.6680
```

```r
# summary(Q1B.Location)

Q1B.Location.reml <- rma.mv(yi = lnrr.yi, V = V.d2.shared.lnRR_Location,
    mods = ~Location - 1, random = list(~1 | studyid, ~1 | infoid,
        ~1 | multipleoutcomecluster), data = metadat_Location,
    method = "REML")
summary(Q1B.Location.reml)
```

```
##
## Multivariate Meta-Analysis Model (k = 877; method: REML)
##
##    logLik   Deviance        AIC        BIC       AICc
## -554.5118  1109.0236  1123.0236  1156.4272  1123.1531
##
## Variance Components:
##
##              estim    sqrt  nlvls  fixed                  factor
## sigma^2.1   0.0990  0.3146    120     no                 studyid
## sigma^2.2   0.0372  0.1929    875     no                  infoid
## sigma^2.3   0.0613  0.2475    562     no  multipleoutcomecluster
##
## Test for Residual Heterogeneity:
## QE(df = 873) = 8379.1220, p-val < .0001
##
## Test of Moderators (coefficients 1:4):
## QM(df = 4) = 8.0560, p-val = 0.0895
##
```

```
## Model Results:
##
##                  estimate      se     zval    pval    ci.lb    ci.ub
## LocationFAE      -0.1273  0.0805  -1.5810  0.1139  -0.2852   0.0305
## Locationfield    -0.0580  0.0854  -0.6800  0.4965  -0.2253   0.1092
## Locationlab      -0.1099  0.0488  -2.2527  0.0243  -0.2056  -0.0143  *
## LocationOTC      -0.0489  0.0842  -0.5803  0.5617  -0.2139   0.1162
##
## ---
## Signif. codes:  0 '***' 0.001 '**' 0.01 '*' 0.05 '.' 0.1 ' ' 1
```

```r
# get R2 for location
r2_ml(Q1B.Location.reml)
```

```
##    R2_marginal R2_conditional
##    0.004215699    0.812273434
```

PEST STATUS

```r
# metadat_4P <- readRDS(file = here('R/metadat_4P'))
# summary(metadat_4P$Status) metadat_4P_status <-
# droplevels(subset(metadat_4P,Status=='Beneficial'|Status=='Other
# herbivore'|Status=='Significant pest'))
metadat_Status <- droplevels(subset(metadat_4P, !(is.na(Status))))
# rerun VCV matrix
V.d2.shared.lnRR_Status <- VCV.shared(data = metadat_Status,
    cV = "cV.lnRR", V = "lnrr.vi", cluster = "sharedcontrolcluster",
    obs = "infoid")
## is.positive.definite(V.d2.shared.lnRR_Status) # check
## the VCV is made correctly

Q1B.null.Status <- rma.mv(yi = lnrr.yi, V = V.d2.shared.lnRR_Status,
    random = list(~1 | studyid, ~1 | infoid, ~1 | multipleoutcomecluster),
    data = metadat_Status, method = "ML")
Q1B.Status <- rma.mv(yi = lnrr.yi, V = V.d2.shared.lnRR_Status,
    mods = ~Status - 1, random = list(~1 | studyid, ~1 | infoid,
        ~1 | multipleoutcomecluster), data = metadat_Status,
    method = "ML")
anova(Q1B.null.Status, Q1B.Status)  #P<0.0001
```

```
##
##         df       AIC       BIC      AICc    logLik     LRT   pval        QE
## Full     6 1105.0915 1133.6539 1105.1896 -546.5457                8028.0053
## Reduced  4 1126.6916 1145.7333 1126.7382 -559.3458 25.6002 <.0001 8658.7218
```

```r
# summary(Q1B.Status)


# re-run model with REML for reporting in manscript
Q1B.Status.reml <- rma.mv(yi = lnrr.yi, V = V.d2.shared.lnRR_Status,
    mods = ~Status - 1, random = list(~1 | studyid, ~1 | infoid,
```

```
        ~1 | multipleoutcomecluster), data = metadat_Status,
    method = "REML")
summary(Q1B.Status.reml)
```

```
##
## Multivariate Meta-Analysis Model (k = 863; method: REML)
##
##    logLik   Deviance        AIC        BIC       AICc
## -544.2349  1088.4699  1100.4699  1129.0115  1100.5683
##
## Variance Components:
##
##              estim    sqrt  nlvls  fixed                 factor
## sigma^2.1  0.0723  0.2689    120     no                studyid
## sigma^2.2  0.0378  0.1944    861     no                 infoid
## sigma^2.3  0.0631  0.2511    550     no  multipleoutcomecluster
##
## Test for Residual Heterogeneity:
## QE(df = 860) = 8028.0053, p-val < .0001
##
## Test of Moderators (coefficients 1:3):
## QM(df = 3) = 35.4352, p-val < .0001
##
## Model Results:
##
##                           estimate      se     zval    pval    ci.lb    ci.ub
## StatusBeneficial           -0.3749  0.0634  -5.9146  <.0001  -0.4991  -0.2507
## StatusOther herbivore      -0.0431  0.0552  -0.7806  0.4350  -0.1512   0.0651
## StatusSignificant pest     -0.0041  0.0428  -0.0951  0.9242  -0.0879   0.0798
##
## StatusBeneficial          ***
## StatusOther herbivore
## StatusSignificant pest
##
## ---
## Signif. codes:  0 '***' 0.001 '**' 0.01 '*' 0.05 '.' 0.1 ' ' 1
```

```
# get R2 for model
r2_ml(Q1B.Status.reml)
```

```
##    R2_marginal R2_conditional
##      0.1030722      0.8042375
```

```
# summary(Q1B.Status.reml)$b
(1 - exp(-0.374885929)) * 100  # 31.26% reduction
```

```
## [1] 31.26323
```

FEEDING GUILD

```r
# summary(metadat_4P$feeding.guild)
metadat_4P_feeding.guild <- droplevels(subset(metadat_4P, feeding.guild ==
    "borer/miner" | feeding.guild == "cell-feeder" | feeding.guild ==
    "chewer" | feeding.guild == "detritivore" | feeding.guild ==
    "nectar/pollen-feeder" | feeding.guild == "parasitoid" |
    feeding.guild == "predator"))
metadat_feeding.guild <- droplevels(subset(metadat_4P_feeding.guild,
    !(is.na(feeding.guild))))
# rerun VCV matrix
V.d2.shared.lnRR_feeding.guild <- VCV.shared(data = metadat_feeding.guild,
    cV = "cV.lnRR", V = "lnrr.vi", cluster = "sharedcontrolcluster",
    obs = "infoid")
# is.positive.definite(V.d2.shared.lnRR_feeding.guild) #
# check the VCV is made correctly

Q1B.null.Guild <- rma.mv(yi = lnrr.yi, V = V.d2.shared.lnRR_feeding.guild,
    random = list(~1 | studyid, ~1 | infoid, ~1 | multipleoutcomecluster),
    data = metadat_feeding.guild, method = "ML")
Q1B.Guild <- rma.mv(yi = lnrr.yi, V = V.d2.shared.lnRR_feeding.guild,
    mods = ~feeding.guild - 1, random = list(~1 | studyid, ~1 |
        infoid, ~1 | multipleoutcomecluster), data = metadat_feeding.guild,
    method = "ML")
anova(Q1B.null.Guild, Q1B.Guild)  #P<0.0001
```

```
##
##         df       AIC       BIC      AICc    logLik     LRT   pval        QE
## Full    10 1034.1226 1081.2278 1034.3942 -507.0613                7736.5603
## Reduced  4 1061.2371 1080.0792 1061.2861 -526.6185 39.1145 <.0001 8411.9944
```

```r
# summary(Q1B.Guild)


# re-run model with REML for manuscript
Q1B.Guild.reml <- rma.mv(yi = lnrr.yi, V = V.d2.shared.lnRR_feeding.guild,
    mods = ~feeding.guild - 1, random = list(~1 | studyid, ~1 |
        infoid, ~1 | multipleoutcomecluster), data = metadat_feeding.guild,
    method = "REML")
summary(Q1B.Guild.reml)
```

```
##
## Multivariate Meta-Analysis Model (k = 821; method: REML)
##
##    logLik   Deviance        AIC        BIC       AICc
## -502.4177  1004.8354  1024.8354  1071.8550  1025.1094
##
## Variance Components:
##
##             estim    sqrt  nlvls  fixed                  factor
## sigma^2.1  0.0742  0.2724    116     no                 studyid
## sigma^2.2  0.0381  0.1951    819     no                  infoid
## sigma^2.3  0.0638  0.2526    522     no  multipleoutcomecluster
##
```

```
## Test for Residual Heterogeneity:
## QE(df = 814) = 7736.5603, p-val < .0001
##
## Test of Moderators (coefficients 1:7):
## QM(df = 7) = 47.9460, p-val < .0001
##
## Model Results:
##
##                                      estimate      se     zval    pval    ci.lb
## feeding.guildborer/miner               0.4345  0.1566   2.7743  0.0055   0.1276
## feeding.guildcell-feeder               0.0217  0.0587   0.3694  0.7118  -0.0933
## feeding.guildchewer                   -0.0441  0.0481  -0.9162  0.3595  -0.1383
## feeding.guilddetritivore              -0.3556  0.1628  -2.1848  0.0289  -0.6746
## feeding.guildnectar/pollen-feeder     -0.4896  0.0909  -5.3863  <.0001  -0.6677
## feeding.guildparasitoid               -0.2044  0.1192  -1.7154  0.0863  -0.4380
## feeding.guildpredator                 -0.1725  0.1864  -0.9253  0.3548  -0.5379
##                                        ci.ub
## feeding.guildborer/miner              0.7415   **
## feeding.guildcell-feeder              0.1367
## feeding.guildchewer                   0.0502
## feeding.guilddetritivore             -0.0366    *
## feeding.guildnectar/pollen-feeder    -0.3114  ***
## feeding.guildparasitoid               0.0291    .
## feeding.guildpredator                 0.1929
##
## ---
## Signif. codes:  0 '***' 0.001 '**' 0.01 '*' 0.05 '.' 0.1 ' ' 1
```

```
# get R2
r2_ml(Q1B.Guild.reml)
```

```
##    R2_marginal R2_conditional
##      0.1455682      0.8152239
```

INVERTEBRATE ORDER

```
metadat_4P_Invert.Order <- droplevels(subset(metadat_4P, Invert.Order !=
    "Multiple"))
# summary(metadat_4P_Invert.Order$Invert.Order)
metadat_Invert.Order <- droplevels(subset(metadat_4P_Invert.Order,
    !(is.na(Invert.Order))))
# rerun VCV matrix
V.d2.shared.lnRR_Invert.Order <- VCV.shared(data = metadat_Invert.Order,
    cV = "cV.lnRR", V = "lnrr.vi", cluster = "sharedcontrolcluster",
    obs = "infoid")
# is.positive.definite(V.d2.shared.lnRR_Invert.Order) #
# check the VCV is made correctly

Q1B.null.Order <- rma.mv(yi = lnrr.yi, V = V.d2.shared.lnRR_Invert.Order,
    random = list(~1 | studyid, ~1 | infoid, ~1 | multipleoutcomecluster),
    data = metadat_Invert.Order, method = "ML")
Q1B.Order <- rma.mv(yi = lnrr.yi, V = V.d2.shared.lnRR_Invert.Order,
```

```r
    mods = ~Invert.Order - 1, random = list(~1 | studyid, ~1 |
        infoid, ~1 | multipleoutcomecluster), data = metadat_Invert.Order,
    method = "ML")
anova(Q1B.null.Order, Q1B.Order)
```

```
##
##         df       AIC       BIC      AICc    logLik     LRT   pval        QE
## Full    22 1032.8400 1136.2831 1034.1194 -494.4200                7969.5938
## Reduced  4 1037.5511 1056.3590 1037.6006 -514.7756 40.7111 0.0017 8361.0113
```

```r
summary(Q1B.Order)
```

```
##
## Multivariate Meta-Analysis Model (k = 814; method: ML)
##
##    logLik   Deviance        AIC        BIC       AICc
## -494.4200  2444.5808  1032.8400  1136.2831  1034.1194
##
## Variance Components:
##
##              estim    sqrt  nlvls  fixed                  factor
## sigma^2.1  0.0629  0.2508    114     no                 studyid
## sigma^2.2  0.0386  0.1964    812     no                  infoid
## sigma^2.3  0.0612  0.2474    522     no  multipleoutcomecluster
##
## Test for Residual Heterogeneity:
## QE(df = 795) = 7969.5938, p-val < .0001
##
## Test of Moderators (coefficients 1:19):
## QM(df = 19) = 47.8358, p-val = 0.0003
##
## Model Results:
##
##                             estimate      se     zval    pval    ci.lb    ci.ub
## Invert.OrderAcari            -0.7049  0.2817  -2.5021  0.0123  -1.2571  -0.1527
## Invert.OrderAstigmata        -0.2451  0.6151  -0.3984  0.6903  -1.4506   0.9605
## Invert.OrderChilopoda         1.3180  1.0161   1.2971  0.1946  -0.6736   3.3096
## Invert.OrderColeoptera        0.0147  0.0707   0.2076  0.8356  -0.1239   0.1532
## Invert.OrderCollembola       -0.4156  0.1956  -2.1251  0.0336  -0.7989  -0.0323
## Invert.OrderDiplopoda        -1.7711  1.4069  -1.2589  0.2081  -4.5285   0.9864
## Invert.OrderDiptera          -0.3151  0.1805  -1.7455  0.0809  -0.6689   0.0387
## Invert.OrderHaplotaxida      -0.9678  0.3078  -3.1439  0.0017  -1.5711  -0.3644
## Invert.OrderHemiptera         0.0418  0.0588   0.7101  0.4776  -0.0735   0.1570
## Invert.OrderHomoptera        -0.2484  0.1946  -1.2762  0.2019  -0.6298   0.1331
## Invert.OrderHymenoptera      -0.2664  0.0763  -3.4918  0.0005  -0.4160  -0.1169
## Invert.OrderLepidoptera      -0.0626  0.0570  -1.0980  0.2722  -0.1744   0.0492
## Invert.OrderMesostigmata     -0.0293  0.2898  -0.1011  0.9195  -0.5972   0.5386
## Invert.OrderOrbatida          1.1018  0.5525   1.9943  0.0461   0.0190   2.1846
## Invert.OrderOrthoptera       -0.2716  0.2380  -1.1414  0.2537  -0.7381   0.1948
## Invert.OrderProstigmata      -0.4181  0.5302  -0.7886  0.4304  -1.4573   0.6211
## Invert.OrderPsocoptera       -1.8525  0.8591  -2.1562  0.0311  -3.5363  -0.1686
## Invert.OrderThysanoptera      1.6650  1.0650   1.5634  0.1180  -0.4224   3.7524
```

```
## Invert.OrderTrombidiformes   -0.0127  0.3162  -0.0403  0.9679  -0.6324   0.6069
##
## Invert.OrderAcari             *
## Invert.OrderAstigmata
## Invert.OrderChilopoda
## Invert.OrderColeoptera
## Invert.OrderCollembola        *
## Invert.OrderDiplopoda
## Invert.OrderDiptera           .
## Invert.OrderHaplotaxida      **
## Invert.OrderHemiptera
## Invert.OrderHomoptera
## Invert.OrderHymenoptera     ***
## Invert.OrderLepidoptera
## Invert.OrderMesostigmata
## Invert.OrderOrbatida          *
## Invert.OrderOrthoptera
## Invert.OrderProstigmata
## Invert.OrderPsocoptera        *
## Invert.OrderThysanoptera
## Invert.OrderTrombidiformes
##
## ---
## Signif. codes:  0 '***' 0.001 '**' 0.01 '*' 0.05 '.' 0.1 ' ' 1
```

```
Q1B.Order.reml <- rma.mv(yi = lnrr.yi, V = V.d2.shared.lnRR_Invert.Order,
    mods = ~Invert.Order - 1, random = list(~1 | studyid, ~1 |
        infoid, ~1 | multipleoutcomecluster), data = metadat_Invert.Order,
    method = "REML")
# get R2
r2_ml(Q1B.Order.reml)
```

```
##    R2_marginal R2_conditional
##      0.1672841      0.8219108
```

INVERTEBRATE FAMILY

```
summary(metadat_4P$Invert.Family)
```

```
## \tMegachilidae      Acrididae      Agaonidae    Aleyrodidae      Aphididae
##              2              6              4              8            189
##         Apidae     Braconidae  Chrysomelidae  Coccinellidae  Curculionidae
##             39             28            152             24             13
##    Diprionidae Drosophilidae  Enchytraeidae       Erebidae  Eriocraniidae
##              6             10              4             58              4
##    Gelechiidae    Geometridae Gracillariidae Gryllacrididae      Gryllidae
##              2             14              3              2              4
##     Halictidae  Lasiocampidae     Lycaenidae   Megachilidae       Multiple
##              3             36              2              1             44
##      Noctuidae    Nymphalidae Phalangopsidae       Phoridae       Pieridae
##             26              4              1              1             28
```

```
##    Plutellidae    Saturniidae   Scarabaeidae     Sphingidae      Syrphidae
##             29              8             15             20              2
##     Tachinidae  Tetranychidae    Tortricidae           NA's
##              3              6              1             75
```

```
metadat_4P_Invert.Family <- droplevels(subset(metadat_4P, Invert.Family !=
    "Multiple"))
# summary(metadat_4P_Invert.Family$Invert.Family)
metadat_Invert.Family <- droplevels(subset(metadat_4P_Invert.Family,
    !(is.na(Invert.Family))))
# rerun VCV matrix
V.d2.shared.lnRR_Invert.Family <- VCV.shared(data = metadat_Invert.Family,
    cV = "cV.lnRR", V = "lnrr.vi", cluster = "sharedcontrolcluster",
    obs = "infoid")
# is.positive.definite(V.d2.shared.lnRR_Invert.Family) #
# check the VCV is made correctly

Q1B.null.Invert.Family <- rma.mv(yi = lnrr.yi, V = V.d2.shared.lnRR_Invert.Family,
    random = list(~1 | studyid, ~1 | infoid, ~1 | multipleoutcomecluster),
    data = metadat_Invert.Family, method = "ML")
Q1B.Invert.Family <- rma.mv(yi = lnrr.yi, V = V.d2.shared.lnRR_Invert.Family,
    mods = ~Invert.Family - 1, random = list(~1 | studyid, ~1 |
        infoid, ~1 | multipleoutcomecluster), data = metadat_Invert.Family,
    method = "ML")
anova(Q1B.null.Invert.Family, Q1B.Invert.Family)
```

```
##
##         df      AIC       BIC      AICc    logLik     LRT   pval        QE
## Full    40 951.9061 1137.1335 956.4807 -435.9531                5843.3010
## Reduced  4 939.2549  957.7776 939.3080 -465.6275 59.3488 0.0085 7108.2758
```

```
summary(Q1B.Invert.Family)
```

```
##
## Multivariate Meta-Analysis Model (k = 758; method: ML)
##
##    logLik   Deviance        AIC        BIC       AICc
## -435.9531  2270.4824   951.9061  1137.1335   956.4807
##
## Variance Components:
##
##               estim    sqrt  nlvls  fixed                  factor
## sigma^2.1   0.0192  0.1387    107     no                 studyid
## sigma^2.2   0.0385  0.1963    756     no                  infoid
## sigma^2.3   0.0650  0.2549    478     no  multipleoutcomecluster
##
## Test for Residual Heterogeneity:
## QE(df = 721) = 5843.3010, p-val < .0001
##
## Test of Moderators (coefficients 1:37):
## QM(df = 37) = 82.7743, p-val < .0001
##
## Model Results:
```

```
##
##                              estimate      se     zval    pval    ci.lb
## Invert.Family\tMegachilidae   -0.3198  0.3470  -0.9217  0.3567  -1.0000
## Invert.FamilyAcrididae        -0.3265  0.2713  -1.2038  0.2287  -0.8582
## Invert.FamilyAgaonidae        -0.5873  0.2636  -2.2277  0.0259  -1.1040
## Invert.FamilyAleyrodidae      -0.5230  0.2018  -2.5909  0.0096  -0.9186
## Invert.FamilyAphididae         0.0354  0.0434   0.8149  0.4151  -0.0497
## Invert.FamilyApidae           -0.3113  0.0953  -3.2669  0.0011  -0.4981
## Invert.FamilyBraconidae       -0.0281  0.1141  -0.2465  0.8053  -0.2518
## Invert.FamilyChrysomelidae    -0.0730  0.0629  -1.1608  0.2457  -0.1962
## Invert.FamilyCoccinellidae     0.2043  0.1164   1.7555  0.0792  -0.0238
## Invert.FamilyCurculionidae     0.2414  0.1897   1.2722  0.2033  -0.1305
## Invert.FamilyDiprionidae       0.0237  0.2071   0.1146  0.9087  -0.3822
## Invert.FamilyDrosophilidae    -0.1366  0.1837  -0.7439  0.4569  -0.4967
## Invert.FamilyEnchytraeidae    -0.9873  0.2752  -3.5879  0.0003  -1.5267
## Invert.FamilyErebidae         -0.0909  0.0944  -0.9626  0.3357  -0.2759
## Invert.FamilyEriocraniidae     0.0091  0.2173   0.0417  0.9667  -0.4168
## Invert.FamilyGelechiidae       0.5046  0.8991   0.5613  0.5746  -1.2576
## Invert.FamilyGeometridae       0.1385  0.1912   0.7246  0.4687  -0.2362
## Invert.FamilyGracillariidae    0.8080  0.3361   2.4041  0.0162   0.1493
## Invert.FamilyGryllacrididae   -0.2712  0.4484  -0.6047  0.5454  -1.1500
## Invert.FamilyGryllidae        -0.2755  0.3093  -0.8906  0.3731  -0.8817
## Invert.FamilyHalictidae       -0.0884  0.3563  -0.2480  0.8041  -0.7867
## Invert.FamilyLasiocampidae    -0.0307  0.0997  -0.3079  0.7582  -0.2262
## Invert.FamilyLycaenidae       -0.1720  0.4895  -0.3513  0.7253  -1.1314
## Invert.FamilyMegachilidae     -1.1519  0.4287  -2.6871  0.0072  -1.9921
## Invert.FamilyNoctuidae        -0.1721  0.1675  -1.0273  0.3043  -0.5004
## Invert.FamilyNymphalidae      -0.6329  0.2515  -2.5166  0.0118  -1.1258
## Invert.FamilyPhalangopsidae    0.2151  0.6784   0.3171  0.7512  -1.1145
## Invert.FamilyPhoridae         -0.3522  1.4700  -0.2396  0.8107  -3.2332
## Invert.FamilyPieridae         -0.1584  0.1270  -1.2468  0.2125  -0.4074
## Invert.FamilyPlutellidae      -0.2361  0.0958  -2.4647  0.0137  -0.4239
## Invert.FamilySaturniidae      -0.2574  0.2411  -1.0678  0.2856  -0.7299
## Invert.FamilyScarabaeidae      0.0524  0.1750   0.2996  0.7645  -0.2905
## Invert.FamilySphingidae        0.1088  0.1446   0.7524  0.4518  -0.1746
## Invert.FamilySyrphidae        -0.3343  0.3544  -0.9434  0.3455  -1.0289
## Invert.FamilyTachinidae       -0.6001  0.3824  -1.5692  0.1166  -1.3497
## Invert.FamilyTetranychidae    -0.0127  0.2412  -0.0528  0.9579  -0.4855
## Invert.FamilyTortricidae      -1.0320  0.5484  -1.8819  0.0599  -2.1068
##                                ci.ub
## Invert.Family\tMegachilidae   0.3603
## Invert.FamilyAcrididae        0.2051
## Invert.FamilyAgaonidae       -0.0706    *
## Invert.FamilyAleyrodidae     -0.1273   **
## Invert.FamilyAphididae        0.1204
## Invert.FamilyApidae          -0.1246   **
## Invert.FamilyBraconidae       0.1956
## Invert.FamilyChrysomelidae    0.0502
## Invert.FamilyCoccinellidae    0.4325    .
## Invert.FamilyCurculionidae    0.6132
## Invert.FamilyDiprionidae      0.4297
## Invert.FamilyDrosophilidae    0.2234
## Invert.FamilyEnchytraeidae   -0.4480  ***
## Invert.FamilyErebidae         0.0941
```

```
## Invert.FamilyEriocraniidae    0.4350
## Invert.FamilyGelechiidae      2.2668
## Invert.FamilyGeometridae      0.5133
## Invert.FamilyGracillariidae   1.4667    *
## Invert.FamilyGryllacrididae   0.6077
## Invert.FamilyGryllidae        0.3308
## Invert.FamilyHalictidae       0.6100
## Invert.FamilyLasiocampidae    0.1648
## Invert.FamilyLycaenidae       0.7874
## Invert.FamilyMegachilidae    -0.3117   **
## Invert.FamilyNoctuidae        0.1562
## Invert.FamilyNymphalidae     -0.1400    *
## Invert.FamilyPhalangopsidae   1.5447
## Invert.FamilyPhoridae         2.5289
## Invert.FamilyPieridae         0.0906
## Invert.FamilyPlutellidae     -0.0484    *
## Invert.FamilySaturniidae      0.2151
## Invert.FamilyScarabaeidae     0.3953
## Invert.FamilySphingidae       0.3921
## Invert.FamilySyrphidae        0.3603
## Invert.FamilyTachinidae       0.1494
## Invert.FamilyTetranychidae    0.4600
## Invert.FamilyTortricidae      0.0428    .
##
## ---
## Signif. codes:  0 '***' 0.001 '**' 0.01 '*' 0.05 '.' 0.1 ' ' 1
```

```
Q1B.Invert.Family.reml <- rma.mv(yi = lnrr.yi, V = V.d2.shared.lnRR_Invert.Family,
    mods = ~Invert.Family - 1, random = list(~1 | studyid, ~1 |
        infoid, ~1 | multipleoutcomecluster), data = metadat_Invert.Family,
    method = "REML")
# get R2
r2_ml(Q1B.Invert.Family.reml)
```

```
##    R2_marginal R2_conditional
##      0.1768613      0.8084186
```

LIFESTAGE

```
# summary(metadat_4P$Lifestage)
metadat_Lifestage <- droplevels(subset(metadat_4P, !(is.na(Lifestage))))
# rerun VCV matrix
V.d2.shared.lnRR_Lifestage <- VCV.shared(data = metadat_Lifestage,
    cV = "cV.lnRR", V = "lnrr.vi", cluster = "sharedcontrolcluster",
    obs = "infoid")
# is.positive.definite(V.d2.shared.lnRR_Lifestage) # check
# the VCV is made correctly

Q1B.null.Lifestage <- rma.mv(yi = lnrr.yi, V = V.d2.shared.lnRR_Lifestage,
    random = list(~1 | studyid, ~1 | infoid, ~1 | multipleoutcomecluster),
    data = metadat_Lifestage, method = "ML")
```

```r
Q1B.Lifestage <- rma.mv(yi = lnrr.yi, V = V.d2.shared.lnRR_Lifestage,
    mods = ~Lifestage - 1, random = list(~1 | studyid, ~1 | infoid,
        ~1 | multipleoutcomecluster), data = metadat_Lifestage,
    method = "ML")
anova(Q1B.null.Lifestage, Q1B.Lifestage)
```

```
##
##         df      AIC      BIC     AICc    logLik    LRT   pval        QE
## Full     9 839.2478 880.8524 839.4903 -410.6239               6214.3541
## Reduced  4 831.2865 849.7775 831.3401 -411.6433 2.0388 0.8438 6313.1437
```

```r
summary(Q1B.Lifestage)
```

```
##
## Multivariate Meta-Analysis Model (k = 752; method: ML)
##
##    logLik   Deviance        AIC        BIC       AICc
## -410.6239  2180.6877   839.2478   880.8524   839.4903
##
## Variance Components:
##
##              estim    sqrt  nlvls  fixed                  factor
## sigma^2.1  0.0924  0.3040    100     no                 studyid
## sigma^2.2  0.0346  0.1860    750     no                  infoid
## sigma^2.3  0.0351  0.1873    467     no  multipleoutcomecluster
##
## Test for Residual Heterogeneity:
## QE(df = 746) = 6214.3541, p-val < .0001
##
## Test of Moderators (coefficients 1:6):
## QM(df = 6) = 10.0150, p-val = 0.1240
##
## Model Results:
##
##                     estimate      se     zval    pval    ci.lb    ci.ub
## Lifestageadult       -0.1103  0.0450  -2.4527  0.0142  -0.1984  -0.0222  *
## Lifestageeggs         0.1516  0.4222   0.3590  0.7196  -0.6759   0.9790
## Lifestagelarva       -0.0998  0.0481  -2.0747  0.0380  -0.1941  -0.0055  *
## Lifestagemultiple    -0.1273  0.0642  -1.9844  0.0472  -0.2531  -0.0016  *
## Lifestagenymph        0.0181  0.1576   0.1152  0.9083  -0.2907   0.3270
## Lifestagepupa        -0.1674  0.0712  -2.3513  0.0187  -0.3070  -0.0279  *
##
## ---
## Signif. codes:  0 '***' 0.001 '**' 0.01 '*' 0.05 '.' 0.1 ' ' 1
```

```r
Q1B.Lifestage.reml <- rma.mv(yi = lnrr.yi, V = V.d2.shared.lnRR_Lifestage,
    mods = ~Lifestage - 1, random = list(~1 | studyid, ~1 | infoid,
        ~1 | multipleoutcomecluster), data = metadat_Lifestage,
    method = "REML")
# get R2
r2_ml(Q1B.Lifestage.reml)
```

```
##    R2_marginal R2_conditional
##    0.006963507    0.792996750
```

WINGED

```
# summary(metadat_4P$Winged)
metadat_Winged <- droplevels(subset(metadat_4P, !(is.na(Winged))))
# rerun VCV matrix
V.d2.shared.lnRR_Winged <- VCV.shared(data = metadat_Winged,
    cV = "cV.lnRR", V = "lnrr.vi", cluster = "sharedcontrolcluster",
    obs = "infoid")
# is.positive.definite(V.d2.shared.lnRR_Winged) # check the
# VCV is made correctly

Q1B.null.Winged <- rma.mv(yi = lnrr.yi, V = V.d2.shared.lnRR_Winged,
    random = list(~1 | studyid, ~1 | infoid, ~1 | multipleoutcomecluster),
    data = metadat_Winged, method = "ML")
Q1B.Winged <- rma.mv(yi = lnrr.yi, V = V.d2.shared.lnRR_Winged,
    mods = ~Winged - 1, random = list(~1 | studyid, ~1 | infoid,
        ~1 | multipleoutcomecluster), data = metadat_Winged,
    method = "ML")
anova(Q1B.null.Winged, Q1B.Winged)
```

```
##
##         df      AIC      BIC     AICc    logLik    LRT   pval        QE
## Full     6 813.2245 840.6498 813.3433 -400.6122              6946.9926
## Reduced  4 813.0378 831.3213 813.0942 -402.5189 3.8133 0.1486 6973.9295
```

```
summary(Q1B.Winged)
```

```
##
## Multivariate Meta-Analysis Model (k = 714; method: ML)
##
##    logLik   Deviance        AIC        BIC       AICc
## -400.6122  2085.6481   813.2245   840.6498   813.3433
##
## Variance Components:
##
##              estim    sqrt  nlvls  fixed                  factor
## sigma^2.1  0.0943  0.3072     97     no                 studyid
## sigma^2.2  0.0325  0.1802    712     no                  infoid
## sigma^2.3  0.0495  0.2224    445     no  multipleoutcomecluster
##
## Test for Residual Heterogeneity:
## QE(df = 711) = 6946.9926, p-val < .0001
##
## Test of Moderators (coefficients 1:3):
## QM(df = 3) = 11.8442, p-val = 0.0079
##
## Model Results:
##
```

```
##              estimate      se     zval    pval    ci.lb    ci.ub
## Wingedboth    -0.0563  0.0742  -0.7589  0.4479  -0.2016   0.0891
## Wingedno      -0.0626  0.0507  -1.2330  0.2176  -0.1620   0.0369
## Wingedyes     -0.1720  0.0502  -3.4222  0.0006  -0.2704  -0.0735  ***
##
## ---
## Signif. codes:  0 '***' 0.001 '**' 0.01 '*' 0.05 '.' 0.1 ' ' 1
```

```
Q1B.Winged.reml <- rma.mv(yi = lnrr.yi, V = V.d2.shared.lnRR_Winged,
    mods = ~Winged - 1, random = list(~1 | studyid, ~1 | infoid,
        ~1 | multipleoutcomecluster), data = metadat_Winged,
    method = "REML")
# get R2
r2_ml(Q1B.Winged.reml)
```

```
##    R2_marginal R2_conditional
##     0.01545974     0.82315538
```

DIET SPECIALISATION

```
# summary(metadat_4P$Diet.specialisation)
metadat_Diet <- droplevels(subset(metadat_4P, !(is.na(Diet.specialisation))))
# rerun VCV matrix
V.d2.shared.lnRR_Diet <- VCV.shared(data = metadat_Diet, cV = "cV.lnRR",
    V = "lnrr.vi", cluster = "sharedcontrolcluster", obs = "infoid")
# is.positive.definite(V.d2.shared.lnRR_Diet) # check the
# VCV is made correctly

Q1B.null.Diet <- rma.mv(yi = lnrr.yi, V = V.d2.shared.lnRR_Diet,
    random = list(~1 | studyid, ~1 | infoid, ~1 | multipleoutcomecluster),
    data = metadat_Diet, method = "ML")
Q1B.Diet <- rma.mv(yi = lnrr.yi, V = V.d2.shared.lnRR_Diet, mods = ~Diet.specialisation -
    1, random = list(~1 | studyid, ~1 | infoid, ~1 | multipleoutcomecluster),
    data = metadat_Diet, method = "ML")
anova(Q1B.null.Diet, Q1B.Diet)
```

```
##
##         df      AIC      BIC     AICc    logLik    LRT   pval        QE
## Full     5 824.3500 847.0695 824.4371 -407.1750              7873.6884
## Reduced  4 824.0109 842.1866 824.0689 -408.0055 1.6610 0.1975 7873.7252
```

```
summary(Q1B.Diet)
```

```
##
## Multivariate Meta-Analysis Model (k = 695; method: ML)
##
##    logLik   Deviance        AIC        BIC       AICc
## -407.1750  2204.3601   824.3500   847.0695   824.4371
##
## Variance Components:
```

```
##
##              estim    sqrt  nlvls  fixed                 factor
## sigma^2.1  0.0901  0.3002     96     no                studyid
## sigma^2.2  0.0333  0.1825    693     no                 infoid
## sigma^2.3  0.0667  0.2582    429     no  multipleoutcomecluster
##
## Test for Residual Heterogeneity:
## QE(df = 693) = 7873.6884, p-val < .0001
##
## Test of Moderators (coefficients 1:2):
## QM(df = 2) = 8.5313, p-val = 0.0140
##
## Model Results:
##
##                                estimate      se     zval    pval    ci.lb
## Diet.specialisationgeneralist   -0.1424  0.0507  -2.8104  0.0049  -0.2417
## Diet.specialisationspecialist   -0.0471  0.0542  -0.8693  0.3847  -0.1534
##                                  ci.ub
## Diet.specialisationgeneralist  -0.0431  **
## Diet.specialisationspecialist   0.0591
##
## ---
## Signif. codes:  0 '***' 0.001 '**' 0.01 '*' 0.05 '.' 0.1 ' ' 1
```

```
Q1B.Diet.reml <- rma.mv(yi = lnrr.yi, V = V.d2.shared.lnRR_Diet,
    mods = ~Diet.specialisation - 1, random = list(~1 | studyid,
        ~1 | infoid, ~1 | multipleoutcomecluster), data = metadat_Diet,
    method = "REML")
# get R2
r2_ml(Q1B.Diet.reml)
```

```
##    R2_marginal R2_conditional
##     0.01112189     0.83061469
```

PLANT ORDER

```
# summary(metadat_4P$Plant.Order)
metadat_4P_Plant.Order <- droplevels(subset(metadat_4P, Plant.Order !=
    "multiple"))
metadat_Plant.Order <- droplevels(subset(metadat_4P_Plant.Order,
    !(is.na(Plant.Order))))
# rerun VCV matrix
V.d2.shared.lnRR_Plant.Order <- VCV.shared(data = metadat_Plant.Order,
    cV = "cV.lnRR", V = "lnrr.vi", cluster = "sharedcontrolcluster",
    obs = "infoid")
# is.positive.definite(V.d2.shared.lnRR_Plant.Order) #
# check the VCV is made correctly

Q1B.null.Plant.Order <- rma.mv(yi = lnrr.yi, V = V.d2.shared.lnRR_Plant.Order,
    random = list(~1 | studyid, ~1 | infoid, ~1 | multipleoutcomecluster),
    data = metadat_Plant.Order, method = "ML")
```

```r
Q1B.Plant.Order <- rma.mv(yi = lnrr.yi, V = V.d2.shared.lnRR_Plant.Order,
    mods = ~Plant.Order - 1, random = list(~1 | studyid, ~1 |
        infoid, ~1 | multipleoutcomecluster), data = metadat_Plant.Order,
    method = "ML")
anova(Q1B.null.Plant.Order, Q1B.Plant.Order)
```

```
##
##         df      AIC      BIC     AICc    logLik     LRT   pval        QE
## Full    22 886.9487 987.6922 888.4006 -421.4743                5991.0988
## Reduced  4 872.9039 891.2209 872.9598 -432.4519 21.9552 0.2340 7030.0964
```

```r
summary(Q1B.Plant.Order)
```

```
##
## Multivariate Meta-Analysis Model (k = 720; method: ML)
##
##    logLik   Deviance        AIC        BIC       AICc
## -421.4743  2197.5661   886.9487   987.6922   888.4006
##
## Variance Components:
##
##             estim    sqrt  nlvls  fixed                  factor
## sigma^2.1  0.0808  0.2842     98     no                 studyid
## sigma^2.2  0.0375  0.1936    719     no                  infoid
## sigma^2.3  0.0434  0.2082    439     no  multipleoutcomecluster
##
## Test for Residual Heterogeneity:
## QE(df = 701) = 5991.0988, p-val < .0001
##
## Test of Moderators (coefficients 1:19):
## QM(df = 19) = 27.2482, p-val = 0.0990
##
## Model Results:
##
##                              estimate      se     zval    pval    ci.lb    ci.ub
## Plant.Order\tPinales           0.2195  0.1754   1.2514  0.2108  -0.1243   0.5634
## Plant.OrderAsterales           0.0284  0.3708   0.0767  0.9389  -0.6983   0.7551
## Plant.OrderBrassicales        -0.2547  0.0854  -2.9823  0.0029  -0.4221  -0.0873
## Plant.OrderCaryophyllales     -0.2840  0.2698  -1.0527  0.2925  -0.8128   0.2448
## Plant.OrderCucurbitales       -0.6826  0.3524  -1.9370  0.0527  -1.3732   0.0081
## Plant.OrderDipsacales         -0.4779  0.5482  -0.8718  0.3833  -1.5524   0.5965
## Plant.OrderFabales             0.0201  0.0926   0.2168  0.8284  -0.1614   0.2015
## Plant.OrderFagales             0.0839  0.0894   0.9380  0.3483  -0.0914   0.2592
## Plant.OrderGentianales         0.1958  0.4293   0.4561  0.6483  -0.6456   1.0372
## Plant.OrderLamiales           -0.8555  0.4525  -1.8906  0.0587  -1.7424   0.0314
## Plant.OrderMagnoliales         0.0570  0.3343   0.1704  0.8647  -0.5982   0.7121
## Plant.OrderMalpighiales       -0.0536  0.0814  -0.6587  0.5101  -0.2132   0.1059
## Plant.OrderMalvales            0.0906  0.4345   0.2085  0.8348  -0.7609   0.9421
## Plant.OrderPinales             0.0608  0.0946   0.6426  0.5205  -0.1246   0.2462
## Plant.OrderPoales             -0.0838  0.1162  -0.7211  0.4709  -0.3116   0.1440
## Plant.OrderPolypodiales        0.4058  0.1965   2.0650  0.0389   0.0206   0.7910
## Plant.OrderRosales            -0.0467  0.2356  -0.1981  0.8430  -0.5084   0.4151
```

```
## Plant.OrderSapindales        0.0362  0.1881   0.1923  0.8475  -0.3326   0.4049
## Plant.OrderSolanales        -0.1569  0.1344  -1.1674  0.2431  -0.4203   0.1065
##
## Plant.Order\tPinales
## Plant.OrderAsterales
## Plant.OrderBrassicales     **
## Plant.OrderCaryophyllales
## Plant.OrderCucurbitales     .
## Plant.OrderDipsacales
## Plant.OrderFabales
## Plant.OrderFagales
## Plant.OrderGentianales
## Plant.OrderLamiales         .
## Plant.OrderMagnoliales
## Plant.OrderMalpighiales
## Plant.OrderMalvales
## Plant.OrderPinales
## Plant.OrderPoales
## Plant.OrderPolypodiales     *
## Plant.OrderRosales
## Plant.OrderSapindales
## Plant.OrderSolanales
##
## ---
## Signif. codes:  0 '***' 0.001 '**' 0.01 '*' 0.05 '.' 0.1 ' ' 1
```

```
Q1B.Plant.Order.reml <- rma.mv(yi = lnrr.yi, V = V.d2.shared.lnRR_Plant.Order,
    mods = ~Plant.Order - 1, random = list(~1 | studyid, ~1 |
        infoid, ~1 | multipleoutcomecluster), data = metadat_Plant.Order,
    method = "REML")
# get R2
r2_ml(Q1B.Plant.Order.reml)
```

```
##    R2_marginal R2_conditional
##      0.1037354      0.8292699
```

PLANT FAMILY

```
summary(metadat_4P$Plant.Family)
```

```
##        Adoxaceae    Amaranthaceae      Apocynaceae       Asteraceae
##                1                2                3                3
##       Betulaceae     Brassicaceae   Cucurbitaceae Dennstaedtiaceae
##               46              122                2               12
##         Fabaceae         Fagaceae        Lamiaceae     Magnoliaceae
##               74               29                3                4
##        Malvaceae         multiple        Pinaceae          Poaceae
##                3                6               93               74
##     Polygonaceae         Rosaceae       Salicaceae      Sapindaceae
##                2               41              119               24
##       Solanaceae             NA's
##               63              151
```

```r
metadat_4P_Plant.Family <- droplevels(subset(metadat_4P, Plant.Family !=
    "multiple"))
metadat_Plant.Family <- droplevels(subset(metadat_4P_Plant.Family,
    !(is.na(Plant.Family))))
# rerun VCV matrix
V.d2.shared.lnRR_Plant.Family <- VCV.shared(data = metadat_Plant.Family,
    cV = "cV.lnRR", V = "lnrr.vi", cluster = "sharedcontrolcluster",
    obs = "infoid")
# is.positive.definite(V.d2.shared.lnRR_Plant.Family) #
# check the VCV is made correctly

Q1B.null.Plant.Family <- rma.mv(yi = lnrr.yi, V = V.d2.shared.lnRR_Plant.Family,
    random = list(~1 | studyid, ~1 | infoid, ~1 | multipleoutcomecluster),
    data = metadat_Plant.Family, method = "ML")
Q1B.Plant.Family <- rma.mv(yi = lnrr.yi, V = V.d2.shared.lnRR_Plant.Family,
    mods = ~Plant.Family - 1, random = list(~1 | studyid, ~1 |
        infoid, ~1 | multipleoutcomecluster), data = metadat_Plant.Family,
    method = "ML")
anova(Q1B.null.Plant.Family, Q1B.Plant.Family)
```

```
##
##         df      AIC      BIC     AICc    logLik     LRT   pval        QE
## Full    23 887.3366 992.6594 888.9228 -420.6683                5953.4942
## Reduced  4 872.9039 891.2209 872.9598 -432.4519 23.5672 0.2133 7030.0964
```

```r
summary(Q1B.Plant.Family)
```

```
##
## Multivariate Meta-Analysis Model (k = 720; method: ML)
##
##    logLik   Deviance        AIC        BIC       AICc
## -420.6683  2195.9540   887.3366   992.6594   888.9228
##
## Variance Components:
##
##              estim    sqrt  nlvls  fixed                  factor
## sigma^2.1  0.0797  0.2823     98     no                 studyid
## sigma^2.2  0.0372  0.1929    719     no                  infoid
## sigma^2.3  0.0434  0.2083    439     no  multipleoutcomecluster
##
## Test for Residual Heterogeneity:
## QE(df = 700) = 5953.4942, p-val < .0001
##
## Test of Moderators (coefficients 1:20):
## QM(df = 20) = 29.0878, p-val = 0.0860
##
## Model Results:
##
##                               estimate      se     zval    pval    ci.lb
## Plant.FamilyAdoxaceae          -0.4809  0.5473  -0.8787  0.3795  -1.5536
## Plant.FamilyAmaranthaceae      -0.4990  0.3631  -1.3743  0.1694  -1.2105
## Plant.FamilyApocynaceae         0.1958  0.4279   0.4576  0.6472  -0.6428
```

```
## Plant.FamilyAsteraceae          0.0284  0.3691   0.0770  0.9387  -0.6951
## Plant.FamilyBetulaceae          0.1693  0.1099   1.5401  0.1235  -0.0462
## Plant.FamilyBrassicaceae       -0.2543  0.0850  -2.9930  0.0028  -0.4208
## Plant.FamilyCucurbitaceae      -0.6826  0.3506  -1.9468  0.0516  -1.3698
## Plant.FamilyDennstaedtiaceae    0.3661  0.1992   1.8381  0.0661  -0.0243
## Plant.FamilyFabaceae            0.0103  0.0924   0.1119  0.9109  -0.1708
## Plant.FamilyFagaceae           -0.0761  0.1522  -0.5000  0.6171  -0.3743
## Plant.FamilyLamiaceae          -0.8555  0.4512  -1.8961  0.0579  -1.7398
## Plant.FamilyMagnoliaceae        0.0567  0.3333   0.1701  0.8649  -0.5966
## Plant.FamilyMalvaceae           0.0908  0.4330   0.2098  0.8338  -0.7578
## Plant.FamilyPinaceae            0.0691  0.0921   0.7500  0.4532  -0.1115
## Plant.FamilyPoaceae            -0.0839  0.1156  -0.7257  0.4680  -0.3104
## Plant.FamilyPolygonaceae       -0.0240  0.3992  -0.0602  0.9520  -0.8065
## Plant.FamilyRosaceae           -0.0466  0.2344  -0.1988  0.8424  -0.5059
## Plant.FamilySalicaceae         -0.0393  0.0817  -0.4809  0.6306  -0.1995
## Plant.FamilySapindaceae         0.0430  0.1876   0.2293  0.8187  -0.3246
## Plant.FamilySolanaceae         -0.1567  0.1337  -1.1719  0.2413  -0.4188
##                                 ci.ub
## Plant.FamilyAdoxaceae          0.5917
## Plant.FamilyAmaranthaceae      0.2126
## Plant.FamilyApocynaceae        1.0344
## Plant.FamilyAsteraceae         0.7519
## Plant.FamilyBetulaceae         0.3848
## Plant.FamilyBrassicaceae      -0.0878  **
## Plant.FamilyCucurbitaceae      0.0046   .
## Plant.FamilyDennstaedtiaceae   0.7565   .
## Plant.FamilyFabaceae           0.1914
## Plant.FamilyFagaceae           0.2221
## Plant.FamilyLamiaceae          0.0288   .
## Plant.FamilyMagnoliaceae       0.7099
## Plant.FamilyMalvaceae          0.9395
## Plant.FamilyPinaceae           0.2497
## Plant.FamilyPoaceae            0.1427
## Plant.FamilyPolygonaceae       0.7584
## Plant.FamilyRosaceae           0.4128
## Plant.FamilySalicaceae         0.1209
## Plant.FamilySapindaceae        0.4107
## Plant.FamilySolanaceae         0.1054
##
## ---
## Signif. codes:  0 '***' 0.001 '**' 0.01 '*' 0.05 '.' 0.1 ' ' 1
```

```
Q1B.Plant.Family.reml <- rma.mv(yi = lnrr.yi, V = V.d2.shared.lnRR_Plant.Family,
    mods = ~Plant.Family - 1, random = list(~1 | studyid, ~1 |
        infoid, ~1 | multipleoutcomecluster), data = metadat_Plant.Family,
    method = "REML")
# get R2
r2_ml(Q1B.Plant.Family.reml)
```

```
##    R2_marginal R2_conditional
##      0.1020066      0.8317745
```

ANNUALITY

```r
# summary(metadat_4P$Annuality)
metadat_Annuality <- droplevels(subset(metadat_4P, !(is.na(Annuality))))
# rerun VCV matrix
V.d2.shared.lnRR_Annuality <- VCV.shared(data = metadat_Annuality,
    cV = "cV.lnRR", V = "lnrr.vi", cluster = "sharedcontrolcluster",
    obs = "infoid")
# is.positive.definite(V.d2.shared.lnRR_Annuality) # check
# the VCV is made correctly

Q1B.null.Annuality <- rma.mv(yi = lnrr.yi, V = V.d2.shared.lnRR_Annuality,
    random = list(~1 | studyid, ~1 | infoid, ~1 | multipleoutcomecluster),
    data = metadat_Annuality, method = "ML")
Q1B.Annuality <- rma.mv(yi = lnrr.yi, V = V.d2.shared.lnRR_Annuality,
    mods = ~Annuality - 1, random = list(~1 | studyid, ~1 | infoid,
        ~1 | multipleoutcomecluster), data = metadat_Annuality,
    method = "ML")
anova(Q1B.null.Annuality, Q1B.Annuality)
```

```
##
##         df      AIC      BIC     AICc    logLik    LRT   pval        QE
## Full     6 809.2703 836.5078 809.3929 -398.6351              6339.9701
## Reduced  4 807.0654 825.2238 807.1236 -399.5327 1.7951 0.4076 6603.7618
```

```r
summary(Q1B.Annuality)
```

```
##
## Multivariate Meta-Analysis Model (k = 692; method: ML)
##
##    logLik   Deviance        AIC        BIC       AICc
## -398.6351  2112.3348   809.2703   836.5078   809.3929
##
## Variance Components:
##
##              estim    sqrt  nlvls  fixed                  factor
## sigma^2.1   0.1031  0.3212     95     no                 studyid
## sigma^2.2   0.0379  0.1947    691     no                  infoid
## sigma^2.3   0.0366  0.1913    423     no  multipleoutcomecluster
##
## Test for Residual Heterogeneity:
## QE(df = 689) = 6339.9701, p-val < .0001
##
## Test of Moderators (coefficients 1:3):
## QM(df = 3) = 5.5302, p-val = 0.1368
##
## Model Results:
##
##                      estimate      se     zval    pval    ci.lb   ci.ub
## Annualityannual       -0.1106  0.0619  -1.7875  0.0739  -0.2318  0.0107  .
## Annualitybiennial     -0.2950  0.2187  -1.3484  0.1775  -0.7237  0.1338
## Annualityperennial    -0.0393  0.0516  -0.7622  0.4459  -0.1404  0.0618
```

```
##
## ---
## Signif. codes:  0 '***' 0.001 '**' 0.01 '*' 0.05 '.' 0.1 ' ' 1
```

```
Q1B.Annuality.reml <- rma.mv(yi = lnrr.yi, V = V.d2.shared.lnRR_Annuality,
    mods = ~Annuality - 1, random = list(~1 | studyid, ~1 | infoid,
        ~1 | multipleoutcomecluster), data = metadat_Annuality,
    method = "REML")
# get R2
r2_ml(Q1B.Annuality.reml)
```

```
##    R2_marginal R2_conditional
##     0.01120706     0.79705276
```

MONOCOT_DICOT

```
# summary(metadat_4P$Monocot_Dicot)
metadat_Monocot_Dicot <- droplevels(subset(metadat_4P, !(is.na(Monocot_Dicot))))
# rerun VCV matrix
V.d2.shared.lnRR_Monocot_Dicot <- VCV.shared(data = metadat_Monocot_Dicot,
    cV = "cV.lnRR", V = "lnrr.vi", cluster = "sharedcontrolcluster",
    obs = "infoid")
# is.positive.definite(V.d2.shared.lnRR_Monocot_Dicot) #
# check the VCV is made correctly

Q1B.null.Plant.type <- rma.mv(yi = lnrr.yi, V = V.d2.shared.lnRR_Monocot_Dicot,
    random = list(~1 | studyid, ~1 | infoid, ~1 | multipleoutcomecluster),
    data = metadat_Monocot_Dicot, method = "ML")
Q1B.Plant.type <- rma.mv(yi = lnrr.yi, V = V.d2.shared.lnRR_Monocot_Dicot,
    mods = ~Monocot_Dicot - 1, random = list(~1 | studyid, ~1 |
        infoid, ~1 | multipleoutcomecluster), data = metadat_Monocot_Dicot,
    method = "ML")
anova(Q1B.null.Plant.type, Q1B.Plant.type)
```

```
##
##         df      AIC      BIC     AICc    logLik    LRT   pval        QE
## Full     6 857.6779 885.1617 857.7955 -422.8389                6862.3863
## Reduced  4 855.8231 874.1457 855.8790 -423.9116 2.1453 0.3421 6978.9902
```

```
summary(Q1B.Plant.type)
```

```
##
## Multivariate Meta-Analysis Model (k = 721; method: ML)
##
##    logLik   Deviance        AIC        BIC       AICc
## -422.8389  2204.0358   857.6779   885.1617   857.7955
##
## Variance Components:
##
##              estim    sqrt  nlvls  fixed                 factor
```

```
## sigma^2.1  0.0928  0.3046     98     no                 studyid
## sigma^2.2  0.0371  0.1927    720     no                  infoid
## sigma^2.3  0.0434  0.2083    440     no  multipleoutcomecluster
##
## Test for Residual Heterogeneity:
## QE(df = 718) = 6862.3863, p-val < .0001
##
## Test of Moderators (coefficients 1:3):
## QM(df = 3) = 6.5337, p-val = 0.0883
##
## Model Results:
##
##                           estimate      se     zval    pval    ci.lb    ci.ub
## Monocot_Dicotdicot         -0.0950  0.0428  -2.2204  0.0264  -0.1788  -0.0111  *
## Monocot_Dicotgymnosperm     0.0485  0.0937   0.5178  0.6046  -0.1351   0.2321
## Monocot_Dicotmonocot       -0.1243  0.1141  -1.0889  0.2762  -0.3479   0.0994
##
## ---
## Signif. codes:  0 '***' 0.001 '**' 0.01 '*' 0.05 '.' 0.1 ' ' 1
```

```
Q1B.Plant.type.reml <- rma.mv(yi = lnrr.yi, V = V.d2.shared.lnRR_Monocot_Dicot,
    mods = ~Monocot_Dicot - 1, random = list(~1 | studyid, ~1 |
        infoid, ~1 | multipleoutcomecluster), data = metadat_Monocot_Dicot,
    method = "REML")
# get R2
r2_ml(Q1B.Plant.type.reml)
```

```
##    R2_marginal R2_conditional
##     0.01424875     0.79641169
```

Null models for individual pollutants

The above analyses were conducted across all pollutant types. We conducted the next section of work on individual pollutants to understand which factors explain variation in response to the individual pollutants.

O3

```
metadat_O3<-droplevels(subset(metadat_4P,Pollutant2=="O3"))
# rerun VCV matrix:
V.d2.shared.lnRR_O3 <- VCV.shared(data=metadat_O3,
                                  cV="cV.lnRR", #covariance
                                  V = "lnrr.vi", #variance
                                  cluster="sharedcontrolcluster", #clustering factor
                                  obs="infoid") # each data row has a unique infoid
#is.positive.definite(V.d2.shared.lnRR_O3) # check the VCV is made correctly
```

Run null (overall) model for O3 effects

```r
O3.null.reml <- rma.mv(yi = lnrr.yi, V = V.d2.shared.lnRR_O3,
    random = list(~1 | studyid, ~1 | infoid, ~1 | multipleoutcomecluster),
    data = metadat_O3, method = "REML")
i2_ml(O3.null.reml)
```

```
##                   I2_Total                I2_studyid                 I2_infoid
##                   97.57655                  56.49411                  29.34413
## I2_multipleoutcomecluster
##                   11.73832
```

NOx

```r
metadat_NOx<-subset(metadat_4P,Pollutant2=="NOx")

# rerun VCV matrix:
V.d2.shared.lnRR_NOx <- VCV.shared(data=metadat_NOx,
                                  cV="cV.lnRR", #covariance
                                  V = "lnrr.vi", #variance
                                  cluster="sharedcontrolcluster", #clustering factor
                                  obs="infoid") # each data row has a unique infoid
#is.positive.definite(V.d2.shared.lnRR_NOx) # check the VCV is made correctly
```

Run null (overall) model for NOx effects

```r
NOx.null.reml <- rma.mv(yi = lnrr.yi, V = V.d2.shared.lnRR_NOx,
    random = list(~1 | studyid, ~1 | infoid, ~1 | multipleoutcomecluster),
    data = metadat_NOx, method = "REML")
i2_ml(NOx.null.reml)
```

```
##                   I2_Total                I2_studyid                 I2_infoid
##                  96.189942                 42.547393                  2.484249
## I2_multipleoutcomecluster
##                  51.158300
```

PM

```r
metadat_PM<-droplevels(subset(metadat_4P,Pollutant2=="PM"))
# rerun VCV matrix:
V.d2.shared.lnRR_PM <- VCV.shared(data=metadat_PM,
                                  cV="cV.lnRR", #covariance
                                  V = "lnrr.vi", #variance
                                  cluster="sharedcontrolcluster", #clustering factor
                                  obs="infoid") # each data row has a unique infoid
#is.positive.definite(V.d2.shared.lnRR_PM) # check the VCV is made correctly
```

```r
PM.null.reml <- rma.mv(yi = lnrr.yi, V = V.d2.shared.lnRR_PM,
    random = list(~1 | studyid, ~1 | infoid, ~1 | multipleoutcomecluster),
    data = metadat_PM, method = "REML")
i2_ml(PM.null.reml)
```

```
##                    I2_Total                  I2_studyid                   I2_infoid
##                9.649123e+01                7.209799e+01                2.439324e+01
## I2_multipleoutcomecluster
##                1.241288e-08
```

SO2

```
metadat_SO2<-droplevels(subset(metadat_4P,Pollutant2=="SO2"))
# rerun VCV matrix:
V.d2.shared.lnRR_SO2 <- VCV.shared(data=metadat_SO2,
                                   cV="cV.lnRR", #covariance
                                   V = "lnrr.vi", #variance
                                   cluster="sharedcontrolcluster", #clustering factor
                                   obs="infoid") # each data row has a unique infoid
#is.positive.definite(V.d2.shared.lnRR_SO2) # check the VCV is made correctly
```

```
SO2.null.reml <- rma.mv(yi = lnrr.yi, V = V.d2.shared.lnRR_SO2,
    random = list(~1 | studyid, ~1 | infoid, ~1 | multipleoutcomecluster),
    data = metadat_SO2, method = "REML")
i2_ml(SO2.null.reml)
```

```
##                    I2_Total                  I2_studyid                   I2_infoid
##                   99.754083                   20.339884                    8.188646
## I2_multipleoutcomecluster
##                   71.225553
```

Supplementary Note 4: Multilevel models - individual pollutants

Method

METHOD - O3

```
metadat_Location_O3 <- droplevels(subset(metadat_O3, !(is.na(Location))))
# rerun VCV matrix
V.d2.shared.lnRR_Location_O3 <- VCV.shared(data = metadat_Location_O3,
    cV = "cV.lnRR", V = "lnrr.vi", cluster = "sharedcontrolcluster",
    obs = "infoid")
# is.positive.definite(V.d2.shared.lnRR_Location_O3) #
# check the VCV is made correctly

Q1B.null.Location_O3 <- rma.mv(yi = lnrr.yi, V = V.d2.shared.lnRR_Location_O3,
    random = list(~1 | studyid, ~1 | infoid, ~1 | multipleoutcomecluster),
    data = metadat_Location_O3, method = "ML")
Q1B.Location_O3 <- rma.mv(yi = lnrr.yi, V = V.d2.shared.lnRR_Location_O3,
    mods = ~Location - 1, random = list(~1 | studyid, ~1 | infoid,
        ~1 | multipleoutcomecluster), data = metadat_Location_O3,
    method = "ML")
anova(Q1B.null.Location_O3, Q1B.Location_O3)
```

```
##
##         df      AIC      BIC     AICc    logLik    LRT   pval        QE
## Full     7 623.1995 652.3867 623.4377 -304.5997               3812.6601
## Reduced  4 619.3467 636.0252 619.4313 -305.6734 2.1473 0.5424 4123.7388
```

```
Q1B.Location_O3.reml <- rma.mv(yi = lnrr.yi, V = V.d2.shared.lnRR_Location_O3,
    mods = ~Location - 1, random = list(~1 | studyid, ~1 | infoid,
        ~1 | multipleoutcomecluster), data = metadat_Location_O3,
    method = "REML")
summary(Q1B.Location_O3.reml)
```

```
##
## Multivariate Meta-Analysis Model (k = 478; method: REML)
##
##    logLik   Deviance        AIC        BIC       AICc
## -301.3060   602.6120   616.6120   645.7404   616.8523
##
## Variance Components:
##
##              estim    sqrt  nlvls  fixed                  factor
## sigma^2.1   0.1023  0.3198     75     no                 studyid
## sigma^2.2   0.0508  0.2253    477     no                  infoid
## sigma^2.3   0.0208  0.1444    309     no  multipleoutcomecluster
##
## Test for Residual Heterogeneity:
## QE(df = 474) = 3812.6601, p-val < .0001
##
## Test of Moderators (coefficients 1:4):
## QM(df = 4) = 8.4085, p-val = 0.0777
##
## Model Results:
##
##                  estimate      se     zval    pval    ci.lb    ci.ub
## LocationFAE       -0.1151  0.0825  -1.3945  0.1632  -0.2768   0.0467
## Locationfield      0.1470  0.1908   0.7705  0.4410  -0.2270   0.5210
## Locationlab       -0.1378  0.0586  -2.3527  0.0186  -0.2525  -0.0230  *
## LocationOTC       -0.0999  0.0924  -1.0811  0.2797  -0.2809   0.0812
##
## ---
## Signif. codes:  0 '***' 0.001 '**' 0.01 '*' 0.05 '.' 0.1 ' ' 1
```

```
# get R2 for location
r2_ml(Q1B.Location_O3.reml)
```

```
##    R2_marginal R2_conditional
##     0.02555991     0.71550458
```

METHOD - NOx

```
metadat_Location_NOx <- droplevels(subset(metadat_NOx, !(is.na(Location))))
# rerun VCV matrix
```

```r
V.d2.shared.lnRR_Location_NOx <- VCV.shared(data = metadat_Location_NOx,
    cV = "cV.lnRR", V = "lnrr.vi", cluster = "sharedcontrolcluster",
    obs = "infoid")
# is.positive.definite(V.d2.shared.lnRR_Location_NOx) #
# check the VCV is made correctly

Q1B.null.Location_NOx <- rma.mv(yi = lnrr.yi, V = V.d2.shared.lnRR_Location_NOx,
    random = list(~1 | studyid, ~1 | infoid, ~1 | multipleoutcomecluster),
    data = metadat_Location_NOx, method = "ML")
Q1B.Location_NOx <- rma.mv(yi = lnrr.yi, V = V.d2.shared.lnRR_Location_NOx,
    mods = ~Location - 1, random = list(~1 | studyid, ~1 | infoid,
        ~1 | multipleoutcomecluster), data = metadat_Location_NOx,
    method = "ML")
anova(Q1B.null.Location_NOx, Q1B.Location_NOx)
```

```
##
##         df      AIC      BIC     AICc   logLik    LRT   pval       QE
## Full     7 132.7800 150.7304 134.0527 -59.3900               745.5351
## Reduced  4 135.2745 145.5319 135.7141 -63.6373 8.4945 0.0368 861.6905
```

```r
Q1B.Location_NOx.reml <- rma.mv(yi = lnrr.yi, V = V.d2.shared.lnRR_Location_NOx,
    mods = ~Location - 1, random = list(~1 | studyid, ~1 | infoid,
        ~1 | multipleoutcomecluster), data = metadat_Location_NOx,
    method = "REML")
summary(Q1B.Location_NOx.reml)
```

```
##
## Multivariate Meta-Analysis Model (k = 96; method: REML)
##
##   logLik  Deviance       AIC       BIC      AICc
## -57.3780  114.7560  128.7560  146.4085  130.0893
##
## Variance Components:
##
##              estim    sqrt  nlvls  fixed                  factor
## sigma^2.1   0.0747  0.2734     19     no                 studyid
## sigma^2.2   0.0048  0.0690     96     no                  infoid
## sigma^2.3   0.0829  0.2880     73     no  multipleoutcomecluster
##
## Test for Residual Heterogeneity:
## QE(df = 92) = 745.5351, p-val < .0001
##
## Test of Moderators (coefficients 1:4):
## QM(df = 4) = 9.0110, p-val = 0.0608
##
## Model Results:
##
##                   estimate      se     zval    pval    ci.lb    ci.ub
## LocationFAE        -0.4798  0.1897  -2.5299  0.0114  -0.8515  -0.1081  *
## Locationfield       0.1506  0.1957   0.7698  0.4414  -0.2329   0.5341
## Locationlab        -0.0586  0.1074  -0.5461  0.5850  -0.2691   0.1518
## LocationOTC        -0.6183  0.4715  -1.3114  0.1897  -1.5425   0.3058
```

```
##
## ---
## Signif. codes:  0 '***' 0.001 '**' 0.01 '*' 0.05 '.' 0.1 ' ' 1
```

```r
# get R2 for location
r2_ml(Q1B.Location_NOx.reml)
```

```
##    R2_marginal R2_conditional
##      0.2677197      0.9785373
```

METHOD - SO2

```r
metadat_Location_SO2 <- droplevels(subset(metadat_SO2, !(is.na(Location))))
# rerun VCV matrix
V.d2.shared.lnRR_Location_SO2 <- VCV.shared(data = metadat_Location_SO2,
    cV = "cV.lnRR", V = "lnrr.vi", cluster = "sharedcontrolcluster",
    obs = "infoid")
# is.positive.definite(V.d2.shared.lnRR_Location_SO2) #
# check the VCV is made correctly

Q1B.null.Location_SO2 <- rma.mv(yi = lnrr.yi, V = V.d2.shared.lnRR_Location_SO2,
    random = list(~1 | studyid, ~1 | infoid, ~1 | multipleoutcomecluster),
    data = metadat_Location_SO2, method = "ML")
Q1B.Location_SO2 <- rma.mv(yi = lnrr.yi, V = V.d2.shared.lnRR_Location_SO2,
    mods = ~Location - 1, random = list(~1 | studyid, ~1 | infoid,
        ~1 | multipleoutcomecluster), data = metadat_Location_SO2,
    method = "ML")
anova(Q1B.null.Location_SO2, Q1B.Location_SO2)
```

```
##
##         df      AIC      BIC     AICc    logLik    LRT   pval        QE
## Full     7 299.2594 323.7682 299.7320 -142.6297               2671.1052
## Reduced  4 299.3097 313.3147 299.4764 -145.6549 6.0503 0.1092 3118.9168
```

```r
Q1B.Location_SO2.reml <- rma.mv(yi = lnrr.yi, V = V.d2.shared.lnRR_Location_SO2,
    mods = ~Location - 1, random = list(~1 | studyid, ~1 | infoid,
        ~1 | multipleoutcomecluster), data = metadat_Location_SO2,
    method = "REML")
summary(Q1B.Location_SO2.reml)
```

```
##
## Multivariate Meta-Analysis Model (k = 245; method: REML)
##
##    logLik   Deviance        AIC        BIC       AICc
## -139.6585   279.3170   293.3170   317.7105   293.7977
##
## Variance Components:
##
##              estim    sqrt  nlvls  fixed                  factor
## sigma^2.1   0.0386  0.1965     32     no                 studyid
```

```
## sigma^2.2  0.0183  0.1354    245     no                  infoid
## sigma^2.3  0.1600  0.4000    160     no  multipleoutcomecluster
##
## Test for Residual Heterogeneity:
## QE(df = 241) = 2671.1052, p-val < .0001
##
## Test of Moderators (coefficients 1:4):
## QM(df = 4) = 5.8957, p-val = 0.2071
##
## Model Results:
##
##                  estimate      se     zval    pval    ci.lb   ci.ub
## LocationFAE       -0.0963  0.2219  -0.4340  0.6643  -0.5311  0.3386
## Locationfield     -0.1833  0.0946  -1.9387  0.0525  -0.3686  0.0020  .
## Locationlab        0.0651  0.0800   0.8139  0.4157  -0.0917  0.2219
## LocationOTC        0.1825  0.1609   1.1343  0.2567  -0.1328  0.4977
##
## ---
## Signif. codes:  0 '***' 0.001 '**' 0.01 '*' 0.05 '.' 0.1 ' ' 1
```

```r
# get R2 for location
r2_ml(Q1B.Location_SO2.reml)
```

```
##    R2_marginal R2_conditional
##     0.07625819     0.92188889
```

METHOD - PM

```r
metadat_Location_PM <- droplevels(subset(metadat_PM, !(is.na(Location))))
# rerun VCV matrix
V.d2.shared.lnRR_Location_PM <- VCV.shared(data = metadat_Location_PM,
    cV = "cV.lnRR", V = "lnrr.vi", cluster = "sharedcontrolcluster",
    obs = "infoid")
# is.positive.definite(V.d2.shared.lnRR_Location_PM) #
# check the VCV is made correctly

Q1B.null.Location_PM <- rma.mv(yi = lnrr.yi, V = V.d2.shared.lnRR_Location_PM,
    random = list(~1 | studyid, ~1 | infoid, ~1 | multipleoutcomecluster),
    data = metadat_Location_PM, method = "ML")
Q1B.Location_PM <- rma.mv(yi = lnrr.yi, V = V.d2.shared.lnRR_Location_PM,
    mods = ~Location - 1, random = list(~1 | studyid, ~1 | infoid,
        ~1 | multipleoutcomecluster), data = metadat_Location_PM,
    method = "ML")
anova(Q1B.null.Location_PM, Q1B.Location_PM)
```

```
##
##         df     AIC     BIC    AICc   logLik    LRT   pval       QE
## Full     5 68.9951 79.2973 70.1489 -29.4975               438.5236
## Reduced  4 68.2091 76.4509 68.9638 -30.1046 1.2140 0.2705 501.0757
```

```r
Q1B.Location_PM.reml <- rma.mv(yi = lnrr.yi, V = V.d2.shared.lnRR_Location_PM,
    mods = ~Location - 1, random = list(~1 | studyid, ~1 | infoid,
        ~1 | multipleoutcomecluster), data = metadat_Location_PM,
    method = "REML")
summary(Q1B.Location_PM.reml)
```

```
##
## Multivariate Meta-Analysis Model (k = 58; method: REML)
##
##   logLik  Deviance       AIC       BIC      AICc
## -27.3520   54.7041   64.7041   74.8308   65.9041
##
## Variance Components:
##
##              estim    sqrt  nlvls  fixed                  factor
## sigma^2.1  0.2199  0.4689      8     no                 studyid
## sigma^2.2  0.0680  0.2608     57     no                  infoid
## sigma^2.3  0.0000  0.0000     20     no  multipleoutcomecluster
##
## Test for Residual Heterogeneity:
## QE(df = 56) = 438.5236, p-val < .0001
##
## Test of Moderators (coefficients 1:2):
## QM(df = 2) = 2.2503, p-val = 0.3246
##
## Model Results:
##
##                estimate      se     zval    pval    ci.lb   ci.ub
## Locationfield   -0.0136  0.2656  -0.0512  0.9591  -0.5341  0.5069
## Locationlab     -0.3982  0.2656  -1.4992  0.1338  -0.9189  0.1224
##
## ---
## Signif. codes:  0 '***' 0.001 '**' 0.01 '*' 0.05 '.' 0.1 ' ' 1
```

```r
# get R2 for location
r2_ml(Q1B.Location_PM.reml)
```

```
##    R2_marginal R2_conditional
##      0.0743835      0.7813123
```

Invertebrate Order

INVERTEBRATE ORDER - O3

```r
# summary(metadat_O3$Invert.Order)
metadat_O3_Invert.Order <- droplevels(subset(metadat_O3, Invert.Order !=
    "Multiple"))
metadat_Invert.Order_O3 <- droplevels(subset(metadat_O3_Invert.Order,
    !(is.na(Invert.Order))))
# rerun VCV matrix
```

```r
V.d2.shared.lnRR_Invert.Order_03 <- VCV.shared(data = metadat_Invert.Order_03,
    cV = "cV.lnRR", V = "lnrr.vi", cluster = "sharedcontrolcluster",
    obs = "infoid")
# is.positive.definite(V.d2.shared.lnRR_Invert.Order_03) #
# check the VCV is made correctly

Q1B.null.Order_03 <- rma.mv(yi = lnrr.yi, V = V.d2.shared.lnRR_Invert.Order_03,
    random = list(~1 | studyid, ~1 | infoid, ~1 | multipleoutcomecluster),
    data = metadat_Invert.Order_03, method = "ML")
Q1B.Order_03 <- rma.mv(yi = lnrr.yi, V = V.d2.shared.lnRR_Invert.Order_03,
    mods = ~Invert.Order - 1, random = list(~1 | studyid, ~1 |
        infoid, ~1 | multipleoutcomecluster), data = metadat_Invert.Order_03,
    method = "ML")
anova(Q1B.null.Order_03, Q1B.Order_03)
```

```
##
##         df      AIC      BIC     AICc    logLik     LRT   pval        QE
## Full    14 578.7407 636.3322 579.7018 -275.3703               3586.3849
## Reduced  4 578.4932 594.9480 578.5827 -285.2466 19.7525 0.0317 4008.7727
```

```r
Q1B.Order_03.reml <- rma.mv(yi = lnrr.yi, V = V.d2.shared.lnRR_Invert.Order_03,
    mods = ~Invert.Order - 1, random = list(~1 | studyid, ~1 |
        infoid, ~1 | multipleoutcomecluster), data = metadat_Invert.Order_03,
    method = "REML")
summary(Q1B.Order_03.reml)
```

```
##
## Multivariate Meta-Analysis Model (k = 452; method: REML)
##
##    logLik   Deviance        AIC        BIC       AICc
## -269.1973   538.3946   566.3946   623.6412   567.3805
##
## Variance Components:
##
##              estim    sqrt  nlvls  fixed                  factor
## sigma^2.1   0.1104  0.3323     72     no                 studyid
## sigma^2.2   0.0517  0.2275    451     no                  infoid
## sigma^2.3   0.0161  0.1270    289     no  multipleoutcomecluster
##
## Test for Residual Heterogeneity:
## QE(df = 441) = 3586.3849, p-val < .0001
##
## Test of Moderators (coefficients 1:11):
## QM(df = 11) = 23.2426, p-val = 0.0163
##
## Model Results:
##
##                             estimate      se     zval    pval    ci.lb    ci.ub
## Invert.OrderAcari            -0.5460  0.3283  -1.6634  0.0962  -1.1894   0.0974
## Invert.OrderColeoptera        0.0120  0.1112   0.1076  0.9143  -0.2059   0.2298
## Invert.OrderCollembola       -0.2194  0.2938  -0.7469  0.4551  -0.7953   0.3564
## Invert.OrderDiptera          -0.6418  0.2494  -2.5736  0.0101  -1.1306  -0.1530
```

```
## Invert.OrderHaplotaxida      -0.8002  0.3495  -2.2892  0.0221  -1.4853  -0.1151
## Invert.OrderHemiptera         0.0200  0.0869   0.2301  0.8180  -0.1504   0.1904
## Invert.OrderHomoptera        -0.3463  0.2297  -1.5079  0.1316  -0.7964   0.1038
## Invert.OrderHymenoptera      -0.3701  0.1155  -3.2051  0.0013  -0.5965  -0.1438
## Invert.OrderLepidoptera      -0.0548  0.0744  -0.7358  0.4618  -0.2006   0.0911
## Invert.OrderMesostigmata     -0.1977  0.4112  -0.4809  0.6306  -1.0036   0.6081
## Invert.OrderTrombidiformes   -0.0127  0.3565  -0.0357  0.9715  -0.7115   0.6861
##
## Invert.OrderAcari             .
## Invert.OrderColeoptera
## Invert.OrderCollembola
## Invert.OrderDiptera           *
## Invert.OrderHaplotaxida       *
## Invert.OrderHemiptera
## Invert.OrderHomoptera
## Invert.OrderHymenoptera      **
## Invert.OrderLepidoptera
## Invert.OrderMesostigmata
## Invert.OrderTrombidiformes
##
## ---
## Signif. codes:  0 '***' 0.001 '**' 0.01 '*' 0.05 '.' 0.1 ' ' 1
```

```
r2_ml(Q1B.Order_O3.reml)
```

```
##    R2_marginal R2_conditional
##      0.1190369      0.7442835
```

INVERTEBRATE ORDER - NOx

```
metadat_NOx_Invert.Order <- droplevels(subset(metadat_NOx, Invert.Order !=
    "Multiple"))
metadat_Invert.Order_NOx <- droplevels(subset(metadat_NOx_Invert.Order,
    !(is.na(Invert.Order))))
# rerun VCV matrix
V.d2.shared.lnRR_Invert.Order_NOx <- VCV.shared(data = metadat_Invert.Order_NOx,
    cV = "cV.lnRR", V = "lnrr.vi", cluster = "sharedcontrolcluster",
    obs = "infoid")
# is.positive.definite(V.d2.shared.lnRR_Invert.Order_NOx) #
# check the VCV is made correctly

Q1B.null.Order_NOx <- rma.mv(yi = lnrr.yi, V = V.d2.shared.lnRR_Invert.Order_NOx,
    random = list(~1 | studyid, ~1 | infoid, ~1 | multipleoutcomecluster),
    data = metadat_Invert.Order_NOx, method = "ML")
Q1B.Order_NOx <- rma.mv(yi = lnrr.yi, V = V.d2.shared.lnRR_Invert.Order_NOx,
    mods = ~Invert.Order - 1, random = list(~1 | studyid, ~1 |
        infoid, ~1 | multipleoutcomecluster), data = metadat_Invert.Order_NOx,
    method = "ML")
anova(Q1B.null.Order_NOx, Q1B.Order_NOx)
```

```
##
```

```
##         df     AIC     BIC    AICc   logLik    LRT   pval       QE
## Full     8 80.8700 98.2651 83.4414 -32.4350               509.1963
## Reduced  4 81.4937 90.1913 82.1604 -36.7469 8.6237 0.0712 538.2573
```

```
Q1B.Order_NOx.reml <- rma.mv(yi = lnrr.yi, V = V.d2.shared.lnRR_Invert.Order_NOx,
    mods = ~Invert.Order - 1, random = list(~1 | studyid, ~1 |
        infoid, ~1 | multipleoutcomecluster), data = metadat_Invert.Order_NOx,
    method = "REML")
summary(Q1B.Order_NOx.reml)
```

```
##
## Multivariate Meta-Analysis Model (k = 65; method: REML)
##
##   logLik  Deviance       AIC       BIC      AICc
## -31.1169   62.2337   78.2337   94.9885   81.0572
##
## Variance Components:
##
##             estim    sqrt  nlvls  fixed                  factor
## sigma^2.1  0.0000  0.0000     15     no                 studyid
## sigma^2.2  0.0000  0.0000     65     no                  infoid
## sigma^2.3  0.0995  0.3155     56     no  multipleoutcomecluster
##
## Test for Residual Heterogeneity:
## QE(df = 60) = 509.1963, p-val < .0001
##
## Test of Moderators (coefficients 1:5):
## QM(df = 5) = 8.4836, p-val = 0.1315
##
## Model Results:
##
##                          estimate      se     zval    pval    ci.lb   ci.ub
## Invert.OrderColeoptera     0.0955  0.1348   0.7084  0.4787  -0.1687  0.3597
## Invert.OrderHemiptera      0.0668  0.0683   0.9791  0.3275  -0.0670  0.2007
## Invert.OrderHomoptera      0.6372  0.5519   1.1546  0.2483  -0.4445  1.7188
## Invert.OrderHymenoptera   -0.1648  0.0950  -1.7345  0.0828  -0.3511  0.0214  .
## Invert.OrderLepidoptera   -0.6522  0.3983  -1.6375  0.1015  -1.4329  0.1284
##
## ---
## Signif. codes:  0 '***' 0.001 '**' 0.01 '*' 0.05 '.' 0.1 ' ' 1
```

```
r2_ml(Q1B.Order_NOx.reml)
```

```
##    R2_marginal R2_conditional
##      0.3414101      1.0000000
```

INVERTEBRATE ORDER - SO2

```
metadat_SO2_Invert.Order <- droplevels(subset(metadat_SO2, Invert.Order !=
    "Multiple"))
metadat_Invert.Order_SO2 <- droplevels(subset(metadat_SO2_Invert.Order,
```

```r
    !(is.na(Invert.Order))))
# rerun VCV matrix
V.d2.shared.lnRR_Invert.Order_SO2 <- VCV.shared(data = metadat_Invert.Order_SO2,
    cV = "cV.lnRR", V = "lnrr.vi", cluster = "sharedcontrolcluster",
    obs = "infoid")
# is.positive.definite(V.d2.shared.lnRR_Invert.Order_SO2) #
# check the VCV is made correctly

Q1B.null.Order_SO2 <- rma.mv(yi = lnrr.yi, V = V.d2.shared.lnRR_Invert.Order_SO2,
    random = list(~1 | studyid, ~1 | infoid, ~1 | multipleoutcomecluster),
    data = metadat_Invert.Order_SO2, method = "ML")
Q1B.Order_SO2 <- rma.mv(yi = lnrr.yi, V = V.d2.shared.lnRR_Invert.Order_SO2,
    mods = ~Invert.Order - 1, random = list(~1 | studyid, ~1 |
        infoid, ~1 | multipleoutcomecluster), data = metadat_Invert.Order_SO2,
    method = "ML")
anova(Q1B.null.Order_SO2, Q1B.Order_SO2)
```

```
##
##         df      AIC      BIC     AICc    logLik     LRT   pval        QE
## Full    18 296.4783 359.0547 299.5874 -130.2392                2599.2907
## Reduced  4 290.2923 304.1981 290.4632 -141.1461 21.8139 0.0825 3057.2724
```

```r
Q1B.Order_SO2.reml <- rma.mv(yi = lnrr.yi, V = V.d2.shared.lnRR_Invert.Order_SO2,
    mods = ~Invert.Order - 1, random = list(~1 | studyid, ~1 |
        infoid, ~1 | multipleoutcomecluster), data = metadat_Invert.Order_SO2,
    method = "REML")
summary(Q1B.Order_SO2.reml)
```

```
##
## Multivariate Meta-Analysis Model (k = 239; method: REML)
##
##    logLik   Deviance        AIC        BIC       AICc
## -119.4237   238.8474   274.8474   336.2570   278.1840
##
## Variance Components:
##
##             estim    sqrt  nlvls  fixed                  factor
## sigma^2.1  0.0608  0.2465     31     no                 studyid
## sigma^2.2  0.0185  0.1359    239     no                  infoid
## sigma^2.3  0.1499  0.3872    157     no  multipleoutcomecluster
##
## Test for Residual Heterogeneity:
## QE(df = 224) = 2599.2907, p-val < .0001
##
## Test of Moderators (coefficients 1:15):
## QM(df = 15) = 21.1456, p-val = 0.1322
##
## Model Results:
##
##                             estimate      se     zval    pval    ci.lb    ci.ub
## Invert.OrderAstigmata        -0.2980  0.6794  -0.4387  0.6609  -1.6297   1.0336
## Invert.OrderChilopoda         1.2650  1.0563   1.1976  0.2311  -0.8053   3.3353
```

```
## Invert.OrderColeoptera     -0.0099  0.1253  -0.0787  0.9373  -0.2553   0.2356
## Invert.OrderCollembola     -1.2505  0.6977  -1.7922  0.0731  -2.6180   0.1170
## Invert.OrderDiplopoda      -1.8241  1.4362  -1.2701  0.2040  -4.6389   0.9908
## Invert.OrderDiptera        -0.0502  0.2953  -0.1700  0.8650  -0.6291   0.5287
## Invert.OrderHemiptera       0.1012  0.1087   0.9312  0.3518  -0.1118   0.3142
## Invert.OrderHymenoptera    -0.1480  0.1680  -0.8807  0.3785  -0.4773   0.1813
## Invert.OrderLepidoptera     0.0399  0.1545   0.2584  0.7961  -0.2628   0.3427
## Invert.OrderMesostigmata    0.1465  0.5473   0.2677  0.7889  -0.9261   1.2191
## Invert.OrderOrbatida        1.0488  0.6233   1.6827  0.0924  -0.1728   2.2704
## Invert.OrderOrthoptera     -0.2801  0.2481  -1.1290  0.2589  -0.7665   0.2062
## Invert.OrderProstigmata    -0.4711  0.6036  -0.7805  0.4351  -1.6542   0.7120
## Invert.OrderPsocoptera     -1.9055  0.9063  -2.1025  0.0355  -3.6818  -0.1292
## Invert.OrderThysanoptera    1.6120  1.1034   1.4609  0.1440  -0.5506   3.7747
##
## Invert.OrderAstigmata
## Invert.OrderChilopoda
## Invert.OrderColeoptera
## Invert.OrderCollembola    .
## Invert.OrderDiplopoda
## Invert.OrderDiptera
## Invert.OrderHemiptera
## Invert.OrderHymenoptera
## Invert.OrderLepidoptera
## Invert.OrderMesostigmata
## Invert.OrderOrbatida      .
## Invert.OrderOrthoptera
## Invert.OrderProstigmata
## Invert.OrderPsocoptera    *
## Invert.OrderThysanoptera
##
## ---
## Signif. codes:  0 '***' 0.001 '**' 0.01 '*' 0.05 '.' 0.1 ' ' 1
```

```r
r2_ml(Q1B.Order_SO2.reml)
```

```
##    R2_marginal R2_conditional
##      0.2328362      0.9381801
```

INVERTEBRATE ORDER - PM

```r
metadat_Invert.Order_PM <- droplevels(subset(metadat_PM, !(is.na(Invert.Order))))
# rerun VCV matrix
V.d2.shared.lnRR_Invert.Order_PM <- VCV.shared(data = metadat_Invert.Order_PM,
    cV = "cV.lnRR", V = "lnrr.vi", cluster = "sharedcontrolcluster",
    obs = "infoid")
# is.positive.definite(V.d2.shared.lnRR_Invert.Order_PM) #
# check the VCV is made correctly

Q1B.null.Order_PM <- rma.mv(yi = lnrr.yi, V = V.d2.shared.lnRR_Invert.Order_PM,
    random = list(~1 | studyid, ~1 | infoid, ~1 | multipleoutcomecluster),
    data = metadat_Invert.Order_PM, method = "ML")
```

```r
Q1B.Order_PM <- rma.mv(yi = lnrr.yi, V = V.d2.shared.lnRR_Invert.Order_PM,
    mods = ~Invert.Order - 1, random = list(~1 | studyid, ~1 |
        infoid, ~1 | multipleoutcomecluster), data = metadat_Invert.Order_PM,
    method = "ML")
anova(Q1B.null.Order_PM, Q1B.Order_PM)
```

```
##
##          df     AIC     BIC    AICc   logLik    LRT   pval       QE
## Full      8 70.4273 86.9108 73.3660 -27.2136               381.3114
## Reduced   4 68.2091 76.4509 68.9638 -30.1046 5.7819 0.2160 501.0757
```

```r
Q1B.Order_PM.reml <- rma.mv(yi = lnrr.yi, V = V.d2.shared.lnRR_Invert.Order_PM,
    mods = ~Invert.Order - 1, random = list(~1 | studyid, ~1 |
        infoid, ~1 | multipleoutcomecluster), data = metadat_Invert.Order_PM,
    method = "REML")
summary(Q1B.Order_PM.reml)
```

```
##
## Multivariate Meta-Analysis Model (k = 58; method: REML)
##
##   logLik  Deviance       AIC       BIC      AICc
## -23.7010   47.4021   63.4021   79.1644   66.6748
##
## Variance Components:
##
##             estim    sqrt  nlvls  fixed                  factor
## sigma^2.1  0.3999  0.6324      8     no                 studyid
## sigma^2.2  0.0677  0.2601     57     no                  infoid
## sigma^2.3  0.0000  0.0000     20     no  multipleoutcomecluster
##
## Test for Residual Heterogeneity:
## QE(df = 53) = 381.3114, p-val < .0001
##
## Test of Moderators (coefficients 1:5):
## QM(df = 5) = 1.9752, p-val = 0.8526
##
## Model Results:
##
##                           estimate      se     zval    pval    ci.lb   ci.ub
## Invert.OrderColeoptera      0.2023  0.4608   0.4389  0.6607  -0.7009  1.1055
## Invert.OrderCollembola     -0.4151  0.6523  -0.6363  0.5246  -1.6935  0.8634
## Invert.OrderDiptera        -0.3844  0.7451  -0.5159  0.6059  -1.8448  1.0760
## Invert.OrderHymenoptera    -0.2593  0.4087  -0.6344  0.5258  -1.0604  0.5418
## Invert.OrderLepidoptera    -0.5495  0.6526  -0.8420  0.3998  -1.8287  0.7296
##
## ---
## Signif. codes:  0 '***' 0.001 '**' 0.01 '*' 0.05 '.' 0.1 ' ' 1
```

```r
r2_ml(Q1B.Order_PM.reml)
```

```
##    R2_marginal R2_conditional
##      0.1542639      0.8776032
```

Invertebrate Family

INVERTEBRATE FAMILY - O3

```
# summary(metadat_O3$Invert.Family)
metadat_O3_Invert.Family <- droplevels(subset(metadat_O3, Invert.Family !=
    "Multiple"))
metadat_Invert.Family_O3 <- droplevels(subset(metadat_O3_Invert.Family,
    !(is.na(Invert.Family))))
# rerun VCV matrix
V.d2.shared.lnRR_Invert.Family_O3 <- VCV.shared(data = metadat_Invert.Family_O3,
    cV = "cV.lnRR", V = "lnrr.vi", cluster = "sharedcontrolcluster",
    obs = "infoid")
# is.positive.definite(V.d2.shared.lnRR_Invert.Family_O3) #
# check the VCV is made correctly

Q1B.null.Invert.Family_O3 <- rma.mv(yi = lnrr.yi, V = V.d2.shared.lnRR_Invert.Family_O3,
    random = list(~1 | studyid, ~1 | infoid, ~1 | multipleoutcomecluster),
    data = metadat_Invert.Family_O3, method = "ML")
Q1B.Invert.Family_O3 <- rma.mv(yi = lnrr.yi, V = V.d2.shared.lnRR_Invert.Family_O3,
    mods = ~Invert.Family - 1, random = list(~1 | studyid, ~1 |
        infoid, ~1 | multipleoutcomecluster), data = metadat_Invert.Family_O3,
    method = "ML")
anova(Q1B.null.Invert.Family_O3, Q1B.Invert.Family_O3)
```

```
##
##         df      AIC      BIC     AICc    logLik     LRT   pval        QE
## Full    28 525.3508 638.7434 529.4622 -234.6754                2671.3416
## Reduced  4 523.1208 539.3198 523.2163 -257.5604 45.7700 0.0047 3680.6213
```

```
Q1B.Invert.Family_O3.reml <- rma.mv(yi = lnrr.yi, V = V.d2.shared.lnRR_Invert.Family_O3,
    mods = ~Invert.Family - 1, random = list(~1 | studyid, ~1 |
        infoid, ~1 | multipleoutcomecluster), data = metadat_Invert.Family_O3,
    method = "REML")
summary(Q1B.Invert.Family_O3.reml)
```

```
##
## Multivariate Meta-Analysis Model (k = 424; method: REML)
##
##    logLik   Deviance        AIC        BIC       AICc
## -225.4171   450.8341   506.8341   618.5250   511.2233
##
## Variance Components:
##
##             estim    sqrt  nlvls  fixed                  factor
## sigma^2.1  0.0637  0.2524     68     no                 studyid
## sigma^2.2  0.0516  0.2272    423     no                  infoid
## sigma^2.3  0.0184  0.1356    265     no  multipleoutcomecluster
##
## Test for Residual Heterogeneity:
## QE(df = 399) = 2671.3416, p-val < .0001
##
```

```
## Test of Moderators (coefficients 1:25):
## QM(df = 25) = 46.3786, p-val = 0.0058
##
## Model Results:
##
##                               estimate      se     zval    pval    ci.lb
## Invert.Family\tMegachilidae    -0.4202  0.3225  -1.3030  0.1926  -1.0524
## Invert.FamilyAgaonidae         -0.5388  0.3228  -1.6689  0.0951  -1.1715
## Invert.FamilyAleyrodidae       -0.5236  0.2742  -1.9098  0.0562  -1.0611
## Invert.FamilyAphididae         -0.0148  0.0721  -0.2059  0.8369  -0.1562
## Invert.FamilyApidae            -0.2180  0.1667  -1.3080  0.1909  -0.5446
## Invert.FamilyBraconidae        -0.2803  0.2095  -1.3379  0.1809  -0.6909
## Invert.FamilyChrysomelidae     -0.0989  0.1120  -0.8833  0.3771  -0.3184
## Invert.FamilyCoccinellidae      0.5521  0.3454   1.5983  0.1100  -0.1249
## Invert.FamilyCurculionidae      0.2746  0.2126   1.2915  0.1965  -0.1421
## Invert.FamilyDiprionidae        0.0221  0.2787   0.0794  0.9367  -0.5240
## Invert.FamilyEnchytraeidae     -0.9720  0.3322  -2.9264  0.0034  -1.6231
## Invert.FamilyErebidae          -0.0812  0.1126  -0.7211  0.4708  -0.3018
## Invert.FamilyGelechiidae        0.5080  0.9139   0.5559  0.5783  -1.2831
## Invert.FamilyLasiocampidae     -0.0480  0.1208  -0.3974  0.6911  -0.2849
## Invert.FamilyMegachilidae      -0.9876  0.3993  -2.4731  0.0134  -1.7703
## Invert.FamilyNymphalidae        0.2011  0.3887   0.5172  0.6050  -0.5608
## Invert.FamilyPieridae          -0.1609  0.1606  -1.0015  0.3166  -0.4757
## Invert.FamilyPlutellidae       -0.2537  0.1192  -2.1291  0.0332  -0.4872
## Invert.FamilySaturniidae       -0.2652  0.2806  -0.9453  0.3445  -0.8152
## Invert.FamilyScarabaeidae      -0.3434  0.3199  -1.0733  0.2831  -0.9703
## Invert.FamilySphingidae         0.1695  0.2019   0.8394  0.4012  -0.2263
## Invert.FamilySyrphidae         -0.4345  0.3298  -1.3175  0.1877  -1.0808
## Invert.FamilyTachinidae        -0.8496  0.4087  -2.0790  0.0376  -1.6506
## Invert.FamilyTetranychidae     -0.0127  0.2855  -0.0446  0.9644  -0.5724
## Invert.FamilyTortricidae       -1.0320  0.5583  -1.8486  0.0645  -2.1261
##                                 ci.ub
## Invert.Family\tMegachilidae    0.2119
## Invert.FamilyAgaonidae         0.0940   .
## Invert.FamilyAleyrodidae       0.0138   .
## Invert.FamilyAphididae         0.1265
## Invert.FamilyApidae            0.1087
## Invert.FamilyBraconidae        0.1303
## Invert.FamilyChrysomelidae     0.1206
## Invert.FamilyCoccinellidae     1.2291
## Invert.FamilyCurculionidae     0.6913
## Invert.FamilyDiprionidae       0.5683
## Invert.FamilyEnchytraeidae    -0.3210  **
## Invert.FamilyErebidae          0.1395
## Invert.FamilyGelechiidae       2.2991
## Invert.FamilyLasiocampidae     0.1888
## Invert.FamilyMegachilidae     -0.2049   *
## Invert.FamilyNymphalidae       0.9630
## Invert.FamilyPieridae          0.1539
## Invert.FamilyPlutellidae      -0.0202   *
## Invert.FamilySaturniidae       0.2847
## Invert.FamilyScarabaeidae      0.2836
## Invert.FamilySphingidae        0.5653
## Invert.FamilySyrphidae         0.2119
```

```
## Invert.FamilyTachinidae       -0.0487   *
## Invert.FamilyTetranychidae     0.5469
## Invert.FamilyTortricidae       0.0622   .
##
## ---
## Signif. codes:  0 '***' 0.001 '**' 0.01 '*' 0.05 '.' 0.1 ' ' 1
```

```
r2_ml(Q1B.Invert.Family_O3.reml)
```

```
##    R2_marginal R2_conditional
##      0.2249564      0.7008223
```

INVERTEBRATE FAMILY - NOx

```
# summary(metadat_NOx$Invert.Family)
metadat_NOx_Invert.Family <- droplevels(subset(metadat_NOx, Invert.Family !=
    "Multiple"))
metadat_Invert.Family_NOx <- droplevels(subset(metadat_NOx_Invert.Family,
    !(is.na(Invert.Family))))
# rerun VCV matrix
V.d2.shared.lnRR_Invert.Family_NOx <- VCV.shared(data = metadat_Invert.Family_NOx,
    cV = "cV.lnRR", V = "lnrr.vi", cluster = "sharedcontrolcluster",
    obs = "infoid")
# is.positive.definite(V.d2.shared.lnRR_Invert.Family_NOx)
# # check the VCV is made correctly

Q1B.null.Invert.Family_NOx <- rma.mv(yi = lnrr.yi, V = V.d2.shared.lnRR_Invert.Family_NOx,
    random = list(~1 | studyid, ~1 | infoid, ~1 | multipleoutcomecluster),
    data = metadat_Invert.Family_NOx, method = "ML")
Q1B.Invert.Family_NOx <- rma.mv(yi = lnrr.yi, V = V.d2.shared.lnRR_Invert.Family_NOx,
    mods = ~Invert.Family - 1, random = list(~1 | studyid, ~1 |
        infoid, ~1 | multipleoutcomecluster), data = metadat_Invert.Family_NOx,
    method = "ML")
anova(Q1B.null.Invert.Family_NOx, Q1B.Invert.Family_NOx)
```

```
##
##         df     AIC     BIC    AICc   logLik     LRT   pval       QE
## Full     8 73.1564 90.1735 75.8734 -28.5782                482.4424
## Reduced  4 77.9124 86.4209 78.6141 -34.9562 12.7559 0.0125 524.2354
```

```
Q1B.Invert.Family_NOx.reml <- rma.mv(yi = lnrr.yi, V = V.d2.shared.lnRR_Invert.Family_NOx,
    mods = ~Invert.Family - 1, random = list(~1 | studyid, ~1 |
        infoid, ~1 | multipleoutcomecluster), data = metadat_Invert.Family_NOx,
    method = "REML")
summary(Q1B.Invert.Family_NOx.reml)
```

```
##
## Multivariate Meta-Analysis Model (k = 62; method: REML)
##
##   logLik  Deviance       AIC       BIC      AICc
```

```
## -28.2236   56.4472   72.4472   88.7916   75.4472
##
## Variance Components:
##
##               estim    sqrt  nlvls  fixed                 factor
## sigma^2.1  0.0000  0.0000     15     no                studyid
## sigma^2.2  0.0000  0.0000     62     no                 infoid
## sigma^2.3  0.0912  0.3019     53     no  multipleoutcomecluster
##
## Test for Residual Heterogeneity:
## QE(df = 57) = 482.4424, p-val < .0001
##
## Test of Moderators (coefficients 1:5):
## QM(df = 5) = 12.7991, p-val = 0.0253
##
## Model Results:
##
##                           estimate      se     zval    pval    ci.lb    ci.ub
## Invert.FamilyAphididae       0.0758  0.0652   1.1631  0.2448  -0.0519   0.2036
## Invert.FamilyApidae         -0.4067  0.1509  -2.6948  0.0070  -0.7026  -0.1109
## Invert.FamilyBraconidae      0.1333  0.1374   0.9699  0.3321  -0.1361   0.4027
## Invert.FamilyScarabaeidae    0.0909  0.1306   0.6961  0.4864  -0.1650   0.3468
## Invert.FamilySphingidae     -0.6525  0.3928  -1.6610  0.0967  -1.4223   0.1174
##
## Invert.FamilyAphididae
## Invert.FamilyApidae         **
## Invert.FamilyBraconidae
## Invert.FamilyScarabaeidae
## Invert.FamilySphingidae     .
##
## ---
## Signif. codes:  0 '***' 0.001 '**' 0.01 '*' 0.05 '.' 0.1 ' ' 1
```

```
r2_ml(Q1B.Invert.Family_NOx.reml)
```

```
##    R2_marginal R2_conditional
##      0.3630829      1.0000000
```

INVERTEBRATE FAMILY - SO2

```
# summary(metadat_SO2$Invert.Family)
metadat_SO2_Invert.Family <- droplevels(subset(metadat_SO2, Invert.Family !=
    "Multiple"))
metadat_Invert.Family_SO2 <- droplevels(subset(metadat_SO2_Invert.Family,
    !(is.na(Invert.Family))))
# rerun VCV matrix
V.d2.shared.lnRR_Invert.Family_SO2 <- VCV.shared(data = metadat_Invert.Family_SO2,
    cV = "cV.lnRR", V = "lnrr.vi", cluster = "sharedcontrolcluster",
    obs = "infoid")
# is.positive.definite(V.d2.shared.lnRR_Invert.Family_SO2)
# # check the VCV is made correctly
```

```r
Q1B.null.Invert.Family_SO2 <- rma.mv(yi = lnrr.yi, V = V.d2.shared.lnRR_Invert.Family_SO2,
    random = list(~1 | studyid, ~1 | infoid, ~1 | multipleoutcomecluster),
    data = metadat_Invert.Family_SO2, method = "ML")
Q1B.Invert.Family_SO2 <- rma.mv(yi = lnrr.yi, V = V.d2.shared.lnRR_Invert.Family_SO2,
    mods = ~Invert.Family - 1, random = list(~1 | studyid, ~1 |
        infoid, ~1 | multipleoutcomecluster), data = metadat_Invert.Family_SO2,
    method = "ML")
anova(Q1B.null.Invert.Family_SO2, Q1B.Invert.Family_SO2)
```

```
##
##         df      AIC      BIC     AICc    logLik     LRT   pval        QE
## Full    21 265.7746 336.8490 270.4889 -111.8873                2073.7454
## Reduced  4 248.7559 262.2939 248.9437 -120.3779 16.9813 0.4556 2329.8625
```

```r
Q1B.Invert.Family_SO2.reml <- rma.mv(yi = lnrr.yi, V = V.d2.shared.lnRR_Invert.Family_SO2,
    mods = ~Invert.Family - 1, random = list(~1 | studyid, ~1 |
        infoid, ~1 | multipleoutcomecluster), data = metadat_Invert.Family_SO2,
    method = "REML")
summary(Q1B.Invert.Family_SO2.reml)
```

```
##
## Multivariate Meta-Analysis Model (k = 218; method: REML)
##
##   logLik  Deviance       AIC       BIC      AICc
## -99.4663  198.9327  240.9327  310.1973  246.1237
##
## Variance Components:
##
##             estim    sqrt  nlvls  fixed                  factor
## sigma^2.1  0.0661  0.2570     29     no                 studyid
## sigma^2.2  0.0171  0.1306    218     no                  infoid
## sigma^2.3  0.1399  0.3740    142     no  multipleoutcomecluster
##
## Test for Residual Heterogeneity:
## QE(df = 200) = 2073.7454, p-val < .0001
##
## Test of Moderators (coefficients 1:18):
## QM(df = 18) = 14.6855, p-val = 0.6834
##
## Model Results:
##
##                              estimate      se     zval    pval    ci.lb
## Invert.FamilyAcrididae        -0.3284  0.3599  -0.9124  0.3615  -1.0337
## Invert.FamilyAphididae         0.1027  0.1101   0.9325  0.3511  -0.1131
## Invert.FamilyBraconidae       -0.0263  0.2317  -0.1133  0.9098  -0.4805
## Invert.FamilyChrysomelidae    -0.1205  0.1660  -0.7260  0.4679  -0.4460
## Invert.FamilyCoccinellidae     0.1474  0.1982   0.7436  0.4571  -0.2411
## Invert.FamilyDrosophilidae    -0.0849  0.3220  -0.2636  0.7921  -0.7160
## Invert.FamilyEriocraniidae     0.0084  0.3277   0.0255  0.9797  -0.6340
## Invert.FamilyGeometridae       0.0968  0.2508   0.3861  0.6994  -0.3948
## Invert.FamilyGracillariidae    0.8032  0.4755   1.6892  0.0912  -0.1288
## Invert.FamilyGryllacrididae   -0.2829  0.5270  -0.5368  0.5914  -1.3158
```

```
## Invert.FamilyGryllidae        -0.2947  0.4002  -0.7363  0.4615  -1.0790
## Invert.FamilyHalictidae       -0.0904  0.4381  -0.2064  0.8365  -0.9490
## Invert.FamilyLycaenidae       -0.2125  0.5474  -0.3882  0.6979  -1.2853
## Invert.FamilyNoctuidae         0.0923  0.3120   0.2959  0.7673  -0.5192
## Invert.FamilyNymphalidae      -1.0889  0.4146  -2.6263  0.0086  -1.9015
## Invert.FamilyPhalangopsidae    0.2151  0.7486   0.2874  0.7738  -1.2522
## Invert.FamilyPhoridae         -0.3035  1.4975  -0.2027  0.8394  -3.2385
## Invert.FamilyTachinidae        0.2926  0.9230   0.3170  0.7512  -1.5164
##                                 ci.ub
## Invert.FamilyAcrididae         0.3770
## Invert.FamilyAphididae         0.3185
## Invert.FamilyBraconidae        0.4280
## Invert.FamilyChrysomelidae     0.2049
## Invert.FamilyCoccinellidae     0.5359
## Invert.FamilyDrosophilidae     0.5462
## Invert.FamilyEriocraniidae     0.6507
## Invert.FamilyGeometridae       0.5884
## Invert.FamilyGracillariidae    1.7351   .
## Invert.FamilyGryllacrididae    0.7501
## Invert.FamilyGryllidae         0.4897
## Invert.FamilyHalictidae        0.7682
## Invert.FamilyLycaenidae        0.8603
## Invert.FamilyNoctuidae         0.7038
## Invert.FamilyNymphalidae      -0.2763  **
## Invert.FamilyPhalangopsidae    1.6824
## Invert.FamilyPhoridae          2.6315
## Invert.FamilyTachinidae        2.1016
##
## ---
## Signif. codes:  0 '***' 0.001 '**' 0.01 '*' 0.05 '.' 0.1 ' ' 1
```

```
r2_ml(Q1B.Invert.Family_SO2.reml)
```

```
##    R2_marginal R2_conditional
##      0.1414544      0.9343072
```

INVERTEBRATE FAMILY - PM

```
# summary(metadat_PM$Invert.Family)
metadat_Invert.Family_PM <- droplevels(subset(metadat_PM, !(is.na(Invert.Family))))
# rerun VCV matrix
V.d2.shared.lnRR_Invert.Family_PM <- VCV.shared(data = metadat_Invert.Family_PM,
    cV = "cV.lnRR", V = "lnrr.vi", cluster = "sharedcontrolcluster",
    obs = "infoid")
# is.positive.definite(V.d2.shared.lnRR_Invert.Family_PM) #
# check the VCV is made correctly

Q1B.null.Invert.Family_PM <- rma.mv(yi = lnrr.yi, V = V.d2.shared.lnRR_Invert.Family_PM,
    random = list(~1 | studyid, ~1 | infoid, ~1 | multipleoutcomecluster),
    data = metadat_Invert.Family_PM, method = "ML")
Q1B.Invert.Family_PM <- rma.mv(yi = lnrr.yi, V = V.d2.shared.lnRR_Invert.Family_PM,
```

```r
    mods = ~Invert.Family - 1, random = list(~1 | studyid, ~1 |
        infoid, ~1 | multipleoutcomecluster), data = metadat_Invert.Family_PM,
    method = "ML")
anova(Q1B.null.Invert.Family_PM, Q1B.Invert.Family_PM)
```

```
##
##         df     AIC     BIC    AICc   logLik    LRT   pval       QE
## Full     8 68.7103 84.6222 71.9103 -26.3552               364.3208
## Reduced  4 66.7173 74.6733 67.5337 -29.3587 6.0070 0.1986 399.7649
```

```r
Q1B.Invert.Family_PM.reml <- rma.mv(yi = lnrr.yi, V = V.d2.shared.lnRR_Invert.Family_PM,
    mods = ~Invert.Family - 1, random = list(~1 | studyid, ~1 |
        infoid, ~1 | multipleoutcomecluster), data = metadat_Invert.Family_PM,
    method = "REML")
summary(Q1B.Invert.Family_PM.reml)
```

```
##
## Multivariate Meta-Analysis Model (k = 54; method: REML)
##
##   logLik  Deviance       AIC       BIC      AICc
## -22.3854   44.7708   60.7708   75.9053   64.3708
##
## Variance Components:
##
##              estim    sqrt  nlvls  fixed                  factor
## sigma^2.1   0.6538  0.8086      7     no                 studyid
## sigma^2.2   0.0723  0.2690     53     no                  infoid
## sigma^2.3   0.0000  0.0000     18     no  multipleoutcomecluster
##
## Test for Residual Heterogeneity:
## QE(df = 49) = 364.3208, p-val < .0001
##
## Test of Moderators (coefficients 1:5):
## QM(df = 5) = 1.1119, p-val = 0.9530
##
## Model Results:
##
##                               estimate      se     zval    pval    ci.lb   ci.ub
## Invert.FamilyApidae            -0.2566  0.5028  -0.5104  0.6098  -1.2420  0.7288
## Invert.FamilyChrysomelidae      0.0308  0.8102   0.0381  0.9696  -1.5572  1.6189
## Invert.FamilyDrosophilidae     -0.3848  0.9009  -0.4271  0.6693  -2.1506  1.3810
## Invert.FamilyNoctuidae         -0.5534  0.8251  -0.6707  0.5024  -2.1706  1.0638
## Invert.FamilyScarabaeidae       0.3920  0.8400   0.4667  0.6407  -1.2543  2.0383
##
## Invert.FamilyApidae
## Invert.FamilyChrysomelidae
## Invert.FamilyDrosophilidae
## Invert.FamilyNoctuidae
## Invert.FamilyScarabaeidae
##
## ---
## Signif. codes:  0 '***' 0.001 '**' 0.01 '*' 0.05 '.' 0.1 ' ' 1
```

```r
r2_ml(Q1B.Invert.Family_PM.reml)
```

```
##    R2_marginal R2_conditional
##     0.06893938     0.90725092
```

Lifestage

LIFESTAGE - O3

```r
metadat_Lifestage_O3 <- droplevels(subset(metadat_O3, !(is.na(Lifestage))))
# rerun VCV matrix
V.d2.shared.lnRR_Lifestage_O3 <- VCV.shared(data = metadat_Lifestage_O3,
    cV = "cV.lnRR", V = "lnrr.vi", cluster = "sharedcontrolcluster",
    obs = "infoid")
# is.positive.definite(V.d2.shared.lnRR_Lifestage_O3) #
# check the VCV is made correctly

Q1B.null.Lifestage_O3 <- rma.mv(yi = lnrr.yi, V = V.d2.shared.lnRR_Lifestage_O3,
    random = list(~1 | studyid, ~1 | infoid, ~1 | multipleoutcomecluster),
    data = metadat_Lifestage_O3, method = "ML")
Q1B.Lifestage_O3 <- rma.mv(yi = lnrr.yi, V = V.d2.shared.lnRR_Lifestage_O3,
    mods = ~Lifestage - 1, random = list(~1 | studyid, ~1 | infoid,
        ~1 | multipleoutcomecluster), data = metadat_Lifestage_O3,
    method = "ML")
anova(Q1B.null.Lifestage_O3, Q1B.Lifestage_O3)
```

```
##
##         df      AIC      BIC     AICc    logLik    LRT   pval        QE
## Full     7 503.1654 531.5465 503.4333 -244.5827               2858.3382
## Reduced  4 497.6980 513.9158 497.7930 -244.8490 0.5326 0.9117 3477.2180
```

```r
Q1B.Lifestage_O3.reml <- rma.mv(yi = lnrr.yi, V = V.d2.shared.lnRR_Lifestage_O3,
    mods = ~Lifestage - 1, random = list(~1 | studyid, ~1 | infoid,
        ~1 | multipleoutcomecluster), data = metadat_Lifestage_O3,
    method = "REML")
summary(Q1B.Lifestage_O3.reml)
```

```
##
## Multivariate Meta-Analysis Model (k = 426; method: REML)
##
##    logLik   Deviance        AIC        BIC       AICc
## -241.9417   483.8834   497.8834   526.1985   498.1540
##
## Variance Components:
##
##              estim    sqrt  nlvls  fixed                  factor
## sigma^2.1   0.0953  0.3087     64     no                 studyid
## sigma^2.2   0.0463  0.2151    425     no                  infoid
## sigma^2.3   0.0164  0.1279    270     no  multipleoutcomecluster
##
```

```
## Test for Residual Heterogeneity:
## QE(df = 422) = 2858.3382, p-val < .0001
##
## Test of Moderators (coefficients 1:4):
## QM(df = 4) = 5.9403, p-val = 0.2037
##
## Model Results:
##
##                      estimate      se     zval    pval    ci.lb    ci.ub
## Lifestageadult        -0.1274  0.0619  -2.0566  0.0397  -0.2488  -0.0060  *
## Lifestagelarva        -0.0766  0.0632  -1.2117  0.2256  -0.2005   0.0473
## Lifestagemultiple     -0.1309  0.1111  -1.1784  0.2386  -0.3486   0.0868
## Lifestagenymph        -0.0968  0.1826  -0.5299  0.5962  -0.4546   0.2611
##
## ---
## Signif. codes:  0 '***' 0.001 '**' 0.01 '*' 0.05 '.' 0.1 ' ' 1
```

```
r2_ml(Q1B.Lifestage_O3.reml)
```

```
##    R2_marginal R2_conditional
##    0.003982893    0.708116722
```

LIFESTAGE - NOx

```
metadat_Lifestage_NOx <- droplevels(subset(metadat_NOx, !(is.na(Lifestage))))
# rerun VCV matrix
V.d2.shared.lnRR_Lifestage_NOx <- VCV.shared(data = metadat_Lifestage_NOx,
    cV = "cV.lnRR", V = "lnrr.vi", cluster = "sharedcontrolcluster",
    obs = "infoid")
# is.positive.definite(V.d2.shared.lnRR_Lifestage_NOx) #
# check the VCV is made correctly

Q1B.null.Lifestage_NOx <- rma.mv(yi = lnrr.yi, V = V.d2.shared.lnRR_Lifestage_NOx,
    random = list(~1 | studyid, ~1 | infoid, ~1 | multipleoutcomecluster),
    data = metadat_Lifestage_NOx, method = "ML")
Q1B.Lifestage_NOx <- rma.mv(yi = lnrr.yi, V = V.d2.shared.lnRR_Lifestage_NOx,
    mods = ~Lifestage - 1, random = list(~1 | studyid, ~1 | infoid,
        ~1 | multipleoutcomecluster), data = metadat_Lifestage_NOx,
    method = "ML")
anova(Q1B.null.Lifestage_NOx, Q1B.Lifestage_NOx)
```

```
##
##         df      AIC      BIC     AICc    logLik    LRT   pval       QE
## Full     7 133.3838 150.8043 134.7666 -59.6919               751.2541
## Reduced  4 129.8862 139.8407 130.3624 -60.9431 2.5024 0.4749 843.5594
```

```
Q1B.Lifestage_NOx.reml <- rma.mv(yi = lnrr.yi, V = V.d2.shared.lnRR_Lifestage_NOx,
    mods = ~Lifestage - 1, random = list(~1 | studyid, ~1 | infoid,
        ~1 | multipleoutcomecluster), data = metadat_Lifestage_NOx,
    method = "REML")
summary(Q1B.Lifestage_NOx.reml)
```

```
##
## Multivariate Meta-Analysis Model (k = 89; method: REML)
##
##   logLik  Deviance       AIC       BIC      AICc
## -57.4756  114.9512  128.9512  146.0498  130.4058
##
## Variance Components:
##
##              estim    sqrt  nlvls  fixed                  factor
## sigma^2.1  0.0922  0.3037     17     no                 studyid
## sigma^2.2  0.0046  0.0676     89     no                  infoid
## sigma^2.3  0.0989  0.3145     66     no  multipleoutcomecluster
##
## Test for Residual Heterogeneity:
## QE(df = 85) = 751.2541, p-val < .0001
##
## Test of Moderators (coefficients 1:4):
## QM(df = 4) = 4.6790, p-val = 0.3218
##
## Model Results:
##
##                    estimate      se     zval    pval    ci.lb   ci.ub
## Lifestageadult      -0.1653  0.1144  -1.4441  0.1487  -0.3896  0.0590
## Lifestagelarva      -0.6521  0.5029  -1.2967  0.1947  -1.6378  0.3335
## Lifestagemultiple   -0.1863  0.1655  -1.1257  0.2603  -0.5107  0.1381
## Lifestagenymph       0.1304  0.2895   0.4502  0.6525  -0.4371  0.6978
##
## ---
## Signif. codes:  0 '***' 0.001 '**' 0.01 '*' 0.05 '.' 0.1 ' ' 1
```

```
r2_ml(Q1B.Lifestage_NOx.reml)
```

```
##    R2_marginal R2_conditional
##     0.05092692     0.97784022
```

LIFESTAGE - SO2

```
metadat_Lifestage_SO2 <- droplevels(subset(metadat_SO2, !(is.na(Lifestage))))
# rerun VCV matrix
V.d2.shared.lnRR_Lifestage_SO2 <- VCV.shared(data = metadat_Lifestage_SO2,
    cV = "cV.lnRR", V = "lnrr.vi", cluster = "sharedcontrolcluster",
    obs = "infoid")
# is.positive.definite(V.d2.shared.lnRR_Lifestage_SO2) #
# check the VCV is made correctly

Q1B.null.Lifestage_SO2 <- rma.mv(yi = lnrr.yi, V = V.d2.shared.lnRR_Lifestage_SO2,
    random = list(~1 | studyid, ~1 | infoid, ~1 | multipleoutcomecluster),
    data = metadat_Lifestage_SO2, method = "ML")
Q1B.Lifestage_SO2 <- rma.mv(yi = lnrr.yi, V = V.d2.shared.lnRR_Lifestage_SO2,
    mods = ~Lifestage - 1, random = list(~1 | studyid, ~1 | infoid,
        ~1 | multipleoutcomecluster), data = metadat_Lifestage_SO2,
```

```r
    method = "ML")
anova(Q1B.null.Lifestage_S02, Q1B.Lifestage_S02)
```

```
##
##         df      AIC      BIC     AICc   logLik    LRT   pval        QE
## Full     9 140.0502 168.9356 141.0907 -61.0251               1372.6091
## Reduced  4 138.8835 151.7214 139.1082 -65.4417 8.8333 0.1159 1468.2555
```

```r
Q1B.Lifestage_S02.reml <- rma.mv(yi = lnrr.yi, V = V.d2.shared.lnRR_Lifestage_S02,
    mods = ~Lifestage - 1, random = list(~1 | studyid, ~1 | infoid,
        ~1 | multipleoutcomecluster), data = metadat_Lifestage_S02,
    method = "REML")
summary(Q1B.Lifestage_S02.reml)
```

```
##
## Multivariate Meta-Analysis Model (k = 183; method: REML)
##
##   logLik  Deviance       AIC       BIC      AICc
## -59.2239  118.4478  136.4478  165.0332  137.5257
##
## Variance Components:
##
##             estim    sqrt  nlvls  fixed                  factor
## sigma^2.1  0.0885  0.2974     24     no                 studyid
## sigma^2.2  0.0131  0.1143    183     no                  infoid
## sigma^2.3  0.0527  0.2295    113     no  multipleoutcomecluster
##
## Test for Residual Heterogeneity:
## QE(df = 177) = 1372.6091, p-val < .0001
##
## Test of Moderators (coefficients 1:6):
## QM(df = 6) = 8.9052, p-val = 0.1790
##
## Model Results:
##
##                     estimate      se     zval    pval    ci.lb    ci.ub
## Lifestageadult       -0.1159  0.0883  -1.3118  0.1896  -0.2890   0.0572
## Lifestageeggs         0.1516  0.4128   0.3672  0.7135  -0.6574   0.9605
## Lifestagelarva       -0.0748  0.0949  -0.7878  0.4308  -0.2608   0.1113
## Lifestagemultiple     0.1076  0.1668   0.6447  0.5191  -0.2194   0.4346
## Lifestagenymph        0.2639  0.3202   0.8243  0.4097  -0.3636   0.8914
## Lifestagepupa        -0.2300  0.0996  -2.3095  0.0209  -0.4252  -0.0348  *
##
## ---
## Signif. codes:  0 '***' 0.001 '**' 0.01 '*' 0.05 '.' 0.1 ' ' 1
```

```r
r2_ml(Q1B.Lifestage_S02.reml)
```

```
##    R2_marginal R2_conditional
##      0.1062869      0.9242779
```

LIFESTAGE - PM

```
metadat_Lifestage_PM <- droplevels(subset(metadat_PM, !(is.na(Lifestage))))
# rerun VCV matrix
V.d2.shared.lnRR_Lifestage_PM <- VCV.shared(data = metadat_Lifestage_PM,
    cV = "cV.lnRR", V = "lnrr.vi", cluster = "sharedcontrolcluster",
    obs = "infoid")
# is.positive.definite(V.d2.shared.lnRR_Lifestage_PM) #
# check the VCV is made correctly

Q1B.null.Lifestage_PM <- rma.mv(yi = lnrr.yi, V = V.d2.shared.lnRR_Lifestage_PM,
    random = list(~1 | studyid, ~1 | infoid, ~1 | multipleoutcomecluster),
    data = metadat_Lifestage_PM, method = "ML")
Q1B.Lifestage_PM <- rma.mv(yi = lnrr.yi, V = V.d2.shared.lnRR_Lifestage_PM,
    mods = ~Lifestage - 1, random = list(~1 | studyid, ~1 | infoid,
        ~1 | multipleoutcomecluster), data = metadat_Lifestage_PM,
    method = "ML")
anova(Q1B.null.Lifestage_PM, Q1B.Lifestage_PM)
```

```
##
##         df     AIC     BIC    AICc   logLik    LRT   pval       QE
## Full     7 71.7419 85.6648 74.1767 -28.8710              345.9690
## Reduced  4 66.7173 74.6733 67.5337 -29.3587 0.9754 0.8072 399.7649
```

```
Q1B.Lifestage_PM.reml <- rma.mv(yi = lnrr.yi, V = V.d2.shared.lnRR_Lifestage_PM,
    mods = ~Lifestage - 1, random = list(~1 | studyid, ~1 | infoid,
        ~1 | multipleoutcomecluster), data = metadat_Lifestage_PM,
    method = "REML")
summary(Q1B.Lifestage_PM.reml)
```

```
##
## Multivariate Meta-Analysis Model (k = 54; method: REML)
##
##   logLik  Deviance       AIC       BIC      AICc
## -27.8157   55.6314   69.6314   83.0155   72.2980
##
## Variance Components:
##
##             estim    sqrt  nlvls  fixed                  factor
## sigma^2.1  0.2369  0.4867      7     no                 studyid
## sigma^2.2  0.0793  0.2816     53     no                  infoid
## sigma^2.3  0.0000  0.0000     18     no  multipleoutcomecluster
##
## Test for Residual Heterogeneity:
## QE(df = 50) = 345.9690, p-val < .0001
##
## Test of Moderators (coefficients 1:4):
## QM(df = 4) = 1.5611, p-val = 0.8158
##
## Model Results:
##
##                     estimate      se     zval    pval    ci.lb   ci.ub
```

```
## Lifestageadult      -0.1221  0.2219  -0.5504  0.5821  -0.5571  0.3128
## Lifestagelarva      -0.2574  0.2324  -1.1080  0.2679  -0.7128  0.1980
## Lifestagemultiple   -0.1920  0.2284  -0.8409  0.4004  -0.6396  0.2555
## Lifestagepupa       -0.1804  0.2420  -0.7455  0.4560  -0.6548  0.2939
##
## ---
## Signif. codes:  0 '***' 0.001 '**' 0.01 '*' 0.05 '.' 0.1 ' ' 1
```

```
r2_ml(Q1B.Lifestage_PM.reml)
```

```
##    R2_marginal R2_conditional
##    0.008139754    0.751208723
```

Winged

WINGED - O3

```
metadat_Winged_O3 <- droplevels(subset(metadat_O3, !(is.na(Winged))))
# rerun VCV matrix
V.d2.shared.lnRR_Winged_O3 <- VCV.shared(data = metadat_Winged_O3,
    cV = "cV.lnRR", V = "lnrr.vi", cluster = "sharedcontrolcluster",
    obs = "infoid")
# is.positive.definite(V.d2.shared.lnRR_Winged_O3) # check
# the VCV is made correctly

Q1B.null.Winged_O3 <- rma.mv(yi = lnrr.yi, V = V.d2.shared.lnRR_Winged_O3,
    random = list(~1 | studyid, ~1 | infoid, ~1 | multipleoutcomecluster),
    data = metadat_Winged_O3, method = "ML")
Q1B.Winged_O3 <- rma.mv(yi = lnrr.yi, V = V.d2.shared.lnRR_Winged_O3,
    mods = ~Winged - 1, random = list(~1 | studyid, ~1 | infoid,
        ~1 | multipleoutcomecluster), data = metadat_Winged_O3,
    method = "ML")
anova(Q1B.null.Winged_O3, Q1B.Winged_O3)
```

```
##
##         df      AIC      BIC     AICc    logLik    LRT   pval        QE
## Full     6 490.3886 514.0611 490.6126 -239.1943              2960.0755
## Reduced  4 488.4090 504.1907 488.5151 -240.2045 2.0204 0.3642 3445.9960
```

```
Q1B.Winged_O3.reml <- rma.mv(yi = lnrr.yi, V = V.d2.shared.lnRR_Winged_O3,
    mods = ~Winged - 1, random = list(~1 | studyid, ~1 | infoid,
        ~1 | multipleoutcomecluster), data = metadat_Winged_O3,
    method = "REML")
summary(Q1B.Winged_O3.reml)
```

```
##
## Multivariate Meta-Analysis Model (k = 382; method: REML)
##
##    logLik   Deviance        AIC        BIC       AICc
## -237.2013   474.4026   486.4026   510.0278   486.6284
```

```
##
## Variance Components:
##
##             estim    sqrt  nlvls  fixed                  factor
## sigma^2.1  0.0873  0.2954     61     no                 studyid
## sigma^2.2  0.0356  0.1887    381     no                  infoid
## sigma^2.3  0.0503  0.2244    246     no  multipleoutcomecluster
##
## Test for Residual Heterogeneity:
## QE(df = 379) = 2960.0755, p-val < .0001
##
## Test of Moderators (coefficients 1:3):
## QM(df = 3) = 10.3962, p-val = 0.0155
##
## Model Results:
##
##             estimate      se     zval    pval    ci.lb    ci.ub
## Wingedboth   -0.1339  0.1058  -1.2660  0.2055  -0.3412   0.0734
## Wingedno     -0.0805  0.0634  -1.2687  0.2045  -0.2048   0.0439
## Wingedyes    -0.2016  0.0671  -3.0051  0.0027  -0.3331  -0.0701  **
##
## ---
## Signif. codes:  0 '***' 0.001 '**' 0.01 '*' 0.05 '.' 0.1 ' ' 1
```

```r
r2_ml(Q1B.Winged_O3.reml)
```

```
##    R2_marginal R2_conditional
##     0.01794766     0.79812652
```

WINGED - NOx

```r
metadat_Winged_NOx <- droplevels(subset(metadat_NOx, !(is.na(Winged))))
# rerun VCV matrix
V.d2.shared.lnRR_Winged_NOx <- VCV.shared(data = metadat_Winged_NOx,
    cV = "cV.lnRR", V = "lnrr.vi", cluster = "sharedcontrolcluster",
    obs = "infoid")
# is.positive.definite(V.d2.shared.lnRR_Winged_NOx) # check
# the VCV is made correctly

Q1B.null.Winged_NOx <- rma.mv(yi = lnrr.yi, V = V.d2.shared.lnRR_Winged_NOx,
    random = list(~1 | studyid, ~1 | infoid, ~1 | multipleoutcomecluster),
    data = metadat_Winged_NOx, method = "ML")
Q1B.Winged_NOx <- rma.mv(yi = lnrr.yi, V = V.d2.shared.lnRR_Winged_NOx,
    mods = ~Winged - 1, random = list(~1 | studyid, ~1 | infoid,
        ~1 | multipleoutcomecluster), data = metadat_Winged_NOx,
    method = "ML")
anova(Q1B.null.Winged_NOx, Q1B.Winged_NOx)
```

```
##
##         df      AIC      BIC     AICc   logLik    LRT   pval       QE
## Full     6 124.5636 139.2897 125.6269 -56.2818               760.1094
## Reduced  4 121.9622 131.7795 122.4560 -56.9811 1.3985 0.4969 814.3682
```

```r
Q1B.Winged_NOx.reml <- rma.mv(yi = lnrr.yi, V = V.d2.shared.lnRR_Winged_NOx,
    mods = ~Winged - 1, random = list(~1 | studyid, ~1 | infoid,
        ~1 | multipleoutcomecluster), data = metadat_Winged_NOx,
    method = "REML")
summary(Q1B.Winged_NOx.reml)
```

```
##
## Multivariate Meta-Analysis Model (k = 86; method: REML)
##
##   logLik  Deviance       AIC       BIC      AICc
## -54.7535  109.5069  121.5069  136.0200  122.6122
##
## Variance Components:
##
##             estim    sqrt  nlvls  fixed                  factor
## sigma^2.1  0.0808  0.2843     16     no                 studyid
## sigma^2.2  0.0043  0.0657     86     no                  infoid
## sigma^2.3  0.1062  0.3259     65     no  multipleoutcomecluster
##
## Test for Residual Heterogeneity:
## QE(df = 83) = 760.1094, p-val < .0001
##
## Test of Moderators (coefficients 1:3):
## QM(df = 3) = 4.4131, p-val = 0.2202
##
## Model Results:
##
##             estimate      se     zval    pval    ci.lb    ci.ub
## Wingedboth   -0.1118  0.1422  -0.7863  0.4317  -0.3904   0.1669
## Wingedno     -0.0126  0.1955  -0.0643  0.9488  -0.3958   0.3707
## Wingedyes    -0.2394  0.1152  -2.0780  0.0377  -0.4652  -0.0136  *
##
## ---
## Signif. codes:  0 '***' 0.001 '**' 0.01 '*' 0.05 '.' 0.1 ' ' 1
```

```r
r2_ml(Q1B.Winged_NOx.reml)
```

```
##    R2_marginal R2_conditional
##     0.03082652     0.97813365
```

WINGED - SO2

```r
metadat_Winged_SO2 <- droplevels(subset(metadat_SO2, !(is.na(Winged))))
# rerun VCV matrix
V.d2.shared.lnRR_Winged_SO2 <- VCV.shared(data = metadat_Winged_SO2,
    cV = "cV.lnRR", V = "lnrr.vi", cluster = "sharedcontrolcluster",
    obs = "infoid")
# is.positive.definite(V.d2.shared.lnRR_Winged_SO2) # check
# the VCV is made correctly
```

```r
Q1B.null.Winged_SO2 <- rma.mv(yi = lnrr.yi, V = V.d2.shared.lnRR_Winged_SO2,
    random = list(~1 | studyid, ~1 | infoid, ~1 | multipleoutcomecluster),
    data = metadat_Winged_SO2, method = "ML")
Q1B.Winged_SO2 <- rma.mv(yi = lnrr.yi, V = V.d2.shared.lnRR_Winged_SO2,
    mods = ~Winged - 1, random = list(~1 | studyid, ~1 | infoid,
        ~1 | multipleoutcomecluster), data = metadat_Winged_SO2,
    method = "ML")
anova(Q1B.null.Winged_SO2, Q1B.Winged_SO2)
```

```
##
##         df      AIC      BIC     AICc   logLik    LRT   pval        QE
## Full     6 152.5301 171.9488 152.9942 -70.2651               2099.7817
## Reduced  4 149.7613 162.7071 149.9799 -70.8806 1.2311 0.5403 2124.9131
```

```r
Q1B.Winged_SO2.reml <- rma.mv(yi = lnrr.yi, V = V.d2.shared.lnRR_Winged_SO2,
    mods = ~Winged - 1, random = list(~1 | studyid, ~1 | infoid,
        ~1 | multipleoutcomecluster), data = metadat_Winged_SO2,
    method = "REML")
summary(Q1B.Winged_SO2.reml)
```

```
##
## Multivariate Meta-Analysis Model (k = 188; method: REML)
##
##   logLik  Deviance       AIC       BIC      AICc
## -68.2908  136.5816  148.5816  167.9037  149.0535
##
## Variance Components:
##
##              estim    sqrt  nlvls  fixed                  factor
## sigma^2.1  0.0914  0.3023     23     no                 studyid
## sigma^2.2  0.0189  0.1375    188     no                  infoid
## sigma^2.3  0.0451  0.2124    114     no  multipleoutcomecluster
##
## Test for Residual Heterogeneity:
## QE(df = 185) = 2099.7817, p-val < .0001
##
## Test of Moderators (coefficients 1:3):
## QM(df = 3) = 1.6157, p-val = 0.6558
##
## Model Results:
##
##              estimate      se     zval    pval    ci.lb   ci.ub
## Wingedboth     0.1492  0.1416   1.0534  0.2922  -0.1284  0.4268
## Wingedno       0.0408  0.0907   0.4494  0.6532  -0.1371  0.2186
## Wingedyes     -0.0183  0.0965  -0.1898  0.8495  -0.2075  0.1709
##
## ---
## Signif. codes:  0 '***' 0.001 '**' 0.01 '*' 0.05 '.' 0.1 ' ' 1
```

```r
r2_ml(Q1B.Winged_SO2.reml)
```

```
##    R2_marginal R2_conditional
##     0.02756291     0.88164517
```

WINGED - PM

```r
metadat_Winged_PM <- droplevels(subset(metadat_PM, !(is.na(Winged))))
# rerun VCV matrix
V.d2.shared.lnRR_Winged_PM <- VCV.shared(data = metadat_Winged_PM,
    cV = "cV.lnRR", V = "lnrr.vi", cluster = "sharedcontrolcluster",
    obs = "infoid")
# is.positive.definite(V.d2.shared.lnRR_Winged_PM) # check
# the VCV is made correctly

Q1B.null.Winged_PM <- rma.mv(yi = lnrr.yi, V = V.d2.shared.lnRR_Winged_PM,
    random = list(~1 | studyid, ~1 | infoid, ~1 | multipleoutcomecluster),
    data = metadat_Winged_PM, method = "ML")
Q1B.Winged_PM <- rma.mv(yi = lnrr.yi, V = V.d2.shared.lnRR_Winged_PM,
    mods = ~Winged - 1, random = list(~1 | studyid, ~1 | infoid,
        ~1 | multipleoutcomecluster), data = metadat_Winged_PM,
    method = "ML")
anova(Q1B.null.Winged_PM, Q1B.Winged_PM)
```

```
##
##         df     AIC     BIC    AICc   logLik    LRT   pval       QE
## Full     6 71.5894 83.9521 73.2365 -29.7947               398.1620
## Reduced  4 68.2091 76.4509 68.9638 -30.1046 0.6197 0.7336 501.0757
```

```r
Q1B.Winged_PM.reml <- rma.mv(yi = lnrr.yi, V = V.d2.shared.lnRR_Winged_PM,
    mods = ~Winged - 1, random = list(~1 | studyid, ~1 | infoid,
        ~1 | multipleoutcomecluster), data = metadat_Winged_PM,
    method = "REML")
summary(Q1B.Winged_PM.reml)
```

```
##
## Multivariate Meta-Analysis Model (k = 58; method: REML)
##
##   logLik  Deviance       AIC       BIC      AICc
## -26.1289   52.2578   64.2578   76.3018   66.0078
##
## Variance Components:
##
##             estim    sqrt  nlvls  fixed                  factor
## sigma^2.1  0.3168  0.5628      8     no                 studyid
## sigma^2.2  0.0676  0.2600     57     no                  infoid
## sigma^2.3  0.0000  0.0000     20     no  multipleoutcomecluster
##
## Test for Residual Heterogeneity:
## QE(df = 55) = 398.1620, p-val < .0001
##
## Test of Moderators (coefficients 1:3):
## QM(df = 3) = 1.1989, p-val = 0.7533
##
## Model Results:
##
##             estimate      se     zval     pval    ci.lb   ci.ub
```

```
## Wingedboth    0.0300  0.5651   0.0531  0.9577  -1.0775  1.1375
## Wingedno     -0.4151  0.5851  -0.7094  0.4781  -1.5618  0.7317
## Wingedyes    -0.2153  0.2587  -0.8324  0.4052  -0.7224  0.2917
##
## ---
## Signif. codes:  0 '***' 0.001 '**' 0.01 '*' 0.05 '.' 0.1 ' ' 1
```

```
r2_ml(Q1B.Winged_PM.reml)
```

```
##    R2_marginal R2_conditional
##     0.05248973     0.83334785
```

Diet specialisation

DIET SPECIALISATION - O3

```
metadat_Diet_O3 <- droplevels(subset(metadat_O3, !(is.na(Diet.specialisation))))
# rerun VCV matrix
V.d2.shared.lnRR_Diet_O3 <- VCV.shared(data = metadat_Diet_O3,
    cV = "cV.lnRR", V = "lnrr.vi", cluster = "sharedcontrolcluster",
    obs = "infoid")
# is.positive.definite(V.d2.shared.lnRR_Diet_O3) # check
# the VCV is made correctly

Q1B.null.Diet_O3 <- rma.mv(yi = lnrr.yi, V = V.d2.shared.lnRR_Diet_O3,
    random = list(~1 | studyid, ~1 | infoid, ~1 | multipleoutcomecluster),
    data = metadat_Diet_O3, method = "ML")
Q1B.Diet_O3 <- rma.mv(yi = lnrr.yi, V = V.d2.shared.lnRR_Diet_O3,
    mods = ~Diet.specialisation - 1, random = list(~1 | studyid,
        ~1 | infoid, ~1 | multipleoutcomecluster), data = metadat_Diet_O3,
    method = "ML")
anova(Q1B.null.Diet_O3, Q1B.Diet_O3)
```

```
##
##         df      AIC      BIC     AICc    logLik    LRT   pval        QE
## Full     5 471.1668 490.9975 471.3230 -230.5834                3661.3835
## Reduced  4 469.3310 485.1955 469.4348 -230.6655 0.1642 0.6854 3663.1456
```

```
Q1B.Diet_O3.reml <- rma.mv(yi = lnrr.yi, V = V.d2.shared.lnRR_Diet_O3,
    mods = ~Diet.specialisation - 1, random = list(~1 | studyid,
        ~1 | infoid, ~1 | multipleoutcomecluster), data = metadat_Diet_O3,
    method = "REML")
summary(Q1B.Diet_O3.reml)
```

```
##
## Multivariate Meta-Analysis Model (k = 390; method: REML)
##
##    logLik   Deviance        AIC        BIC       AICc
## -228.9496   457.8992   467.8992   487.7042   468.0563
##
```

```
## Variance Components:
##
##              estim    sqrt  nlvls  fixed                 factor
## sigma^2.1  0.0947  0.3078     59     no                studyid
## sigma^2.2  0.0358  0.1892    389     no                 infoid
## sigma^2.3  0.0362  0.1901    240     no  multipleoutcomecluster
##
## Test for Residual Heterogeneity:
## QE(df = 388) = 3661.3835, p-val < .0001
##
## Test of Moderators (coefficients 1:2):
## QM(df = 2) = 4.5864, p-val = 0.1009
##
## Model Results:
##
##                                 estimate      se     zval    pval    ci.lb
## Diet.specialisationgeneralist    -0.1199  0.0704  -1.7038  0.0884  -0.2579
## Diet.specialisationspecialist    -0.0839  0.0617  -1.3597  0.1739  -0.2049
##                                   ci.ub
## Diet.specialisationgeneralist  0.0180  .
## Diet.specialisationspecialist  0.0370
##
## ---
## Signif. codes:  0 '***' 0.001 '**' 0.01 '*' 0.05 '.' 0.1 ' ' 1
```

```r
r2_ml(Q1B.Diet_O3.reml)
```

```
##    R2_marginal R2_conditional
##     0.00193799     0.78567746
```

DIET SPECIALISATION - NOx

```r
metadat_Diet_NOx <- droplevels(subset(metadat_NOx, !(is.na(Diet.specialisation))))
# rerun VCV matrix
V.d2.shared.lnRR_Diet_NOx <- VCV.shared(data = metadat_Diet_NOx,
    cV = "cV.lnRR", V = "lnrr.vi", cluster = "sharedcontrolcluster",
    obs = "infoid")
# is.positive.definite(V.d2.shared.lnRR_Diet_NOx) # check
# the VCV is made correctly

Q1B.null.Diet_NOx <- rma.mv(yi = lnrr.yi, V = V.d2.shared.lnRR_Diet_NOx,
    random = list(~1 | studyid, ~1 | infoid, ~1 | multipleoutcomecluster),
    data = metadat_Diet_NOx, method = "ML")
Q1B.Diet_NOx <- rma.mv(yi = lnrr.yi, V = V.d2.shared.lnRR_Diet_NOx,
    mods = ~Diet.specialisation - 1, random = list(~1 | studyid,
        ~1 | infoid, ~1 | multipleoutcomecluster), data = metadat_Diet_NOx,
    method = "ML")
anova(Q1B.null.Diet_NOx, Q1B.Diet_NOx)
```

```
##
##          df      AIC      BIC     AICc    logLik     LRT    pval        QE
```

```
## Full     5 81.1680 90.7281 82.5317 -35.5840               604.2263
## Reduced  4 80.2014 87.8495 81.0903 -36.1007 1.0333 0.3094 604.2342
```

```
Q1B.Diet_NOx.reml <- rma.mv(yi = lnrr.yi, V = V.d2.shared.lnRR_Diet_NOx,
    mods = ~Diet.specialisation - 1, random = list(~1 | studyid,
        ~1 | infoid, ~1 | multipleoutcomecluster), data = metadat_Diet_NOx,
    method = "REML")
summary(Q1B.Diet_NOx.reml)
```

```
##
## Multivariate Meta-Analysis Model (k = 50; method: REML)
##
##   logLik  Deviance       AIC       BIC      AICc
## -34.2029   68.4059   78.4059   87.7619   79.8344
##
## Variance Components:
##
##             estim    sqrt  nlvls  fixed                  factor
## sigma^2.1  0.1523  0.3902     14     no                 studyid
## sigma^2.2  0.0583  0.2414     50     no                  infoid
## sigma^2.3  0.0441  0.2099     44     no  multipleoutcomecluster
##
## Test for Residual Heterogeneity:
## QE(df = 48) = 604.2263, p-val < .0001
##
## Test of Moderators (coefficients 1:2):
## QM(df = 2) = 3.8550, p-val = 0.1455
##
## Model Results:
##
##                                estimate      se     zval    pval    ci.lb
## Diet.specialisationgeneralist   -0.3140  0.1644  -1.9100  0.0561  -0.6362
## Diet.specialisationspecialist   -0.1047  0.1736  -0.6031  0.5465  -0.4449
##                                 ci.ub
## Diet.specialisationgeneralist  0.0082  .
## Diet.specialisationspecialist  0.2355
##
## ---
## Signif. codes:  0 '***' 0.001 '**' 0.01 '*' 0.05 '.' 0.1 ' ' 1
```

```
r2_ml(Q1B.Diet_NOx.reml)
```

```
##    R2_marginal R2_conditional
##     0.04179364     0.78069758
```

DIET SPECIALISATION - SO2

```
metadat_Diet_SO2 <- droplevels(subset(metadat_SO2, !(is.na(Diet.specialisation))))
# rerun VCV matrix
V.d2.shared.lnRR_Diet_SO2 <- VCV.shared(data = metadat_Diet_SO2,
    cV = "cV.lnRR", V = "lnrr.vi", cluster = "sharedcontrolcluster",
```

```r
    obs = "infoid")
# is.positive.definite(V.d2.shared.lnRR_Diet_S02) # check
# the VCV is made correctly

Q1B.null.Diet_S02 <- rma.mv(yi = lnrr.yi, V = V.d2.shared.lnRR_Diet_S02,
    random = list(~1 | studyid, ~1 | infoid, ~1 | multipleoutcomecluster),
    data = metadat_Diet_S02, method = "ML")
Q1B.Diet_S02 <- rma.mv(yi = lnrr.yi, V = V.d2.shared.lnRR_Diet_S02,
    mods = ~Diet.specialisation - 1, random = list(~1 | studyid,
        ~1 | infoid, ~1 | multipleoutcomecluster), data = metadat_Diet_S02,
    method = "ML")
anova(Q1B.null.Diet_S02, Q1B.Diet_S02)
```

```
##
##         df      AIC      BIC     AICc    logLik    LRT   pval        QE
## Full     5 225.1069 241.5985 225.4162 -107.5534              2952.3171
## Reduced  4 225.1835 238.3768 225.3886 -108.5917 2.0766 0.1496 2972.9320
```

```r
Q1B.Diet_S02.reml <- rma.mv(yi = lnrr.yi, V = V.d2.shared.lnRR_Diet_S02,
    mods = ~Diet.specialisation - 1, random = list(~1 | studyid,
        ~1 | infoid, ~1 | multipleoutcomecluster), data = metadat_Diet_S02,
    method = "REML")
summary(Q1B.Diet_S02.reml)
```

```
##
## Multivariate Meta-Analysis Model (k = 200; method: REML)
##
##    logLik   Deviance        AIC        BIC       AICc
## -106.0399   212.0797   222.0797   238.5211   222.3922
##
## Variance Components:
##
##              estim    sqrt  nlvls  fixed                  factor
## sigma^2.1   0.0486  0.2205     25     no                 studyid
## sigma^2.2   0.0190  0.1380    200     no                  infoid
## sigma^2.3   0.1710  0.4135    128     no  multipleoutcomecluster
##
## Test for Residual Heterogeneity:
## QE(df = 198) = 2952.3171, p-val < .0001
##
## Test of Moderators (coefficients 1:2):
## QM(df = 2) = 2.0438, p-val = 0.3599
##
## Model Results:
##
##                                estimate      se     zval    pval    ci.lb
## Diet.specialisationgeneralist   -0.0809  0.0795  -1.0174  0.3090  -0.2367
## Diet.specialisationspecialist    0.1109  0.1104   1.0043  0.3152  -0.1055
##                                  ci.ub
## Diet.specialisationgeneralist  0.0749
## Diet.specialisationspecialist  0.3273
##
```

```
## ---
## Signif. codes:  0 '***' 0.001 '**' 0.01 '*' 0.05 '.' 0.1 ' ' 1
```

```
r2_ml(Q1B.Diet_SO2.reml)
```

```
##    R2_marginal R2_conditional
##     0.02822119     0.92243518
```

###DIET SPECIALISATION - PM {.unlisted .unnumbered} We cannot run the models for diet specialisation because there are not sufficient levels of this moderator within PM

Response Measure

RESPONSE MEASURE - O3

```
# summary(metadat_O3$Performance2)
metadat_Perf_O3 <- droplevels(subset(metadat_O3, !(is.na(Performance2))))
# rerun VCV matrix
V.d2.shared.lnRR_Perf_O3 <- VCV.shared(data = metadat_Perf_O3,
    cV = "cV.lnRR", V = "lnrr.vi", cluster = "sharedcontrolcluster",
    obs = "infoid")
# is.positive.definite(V.d2.shared.lnRR_Perf_O3) # check
# the VCV is made correctly

Q1B.null.Perf_O3 <- rma.mv(yi = lnrr.yi, V = V.d2.shared.lnRR_Perf_O3,
    random = list(~1 | studyid, ~1 | infoid, ~1 | multipleoutcomecluster),
    data = metadat_Perf_O3, method = "ML")
# summary(Q1B.null.Perf_O3)
Q1B.Perf_O3 <- rma.mv(yi = lnrr.yi, V = V.d2.shared.lnRR_Perf_O3,
    mods = ~Performance2 - 1, random = list(~1 | studyid, ~1 |
        infoid, ~1 | multipleoutcomecluster), data = metadat_Perf_O3,
    method = "ML")
anova(Q1B.null.Perf_O3, Q1B.Perf_O3)
```

```
##
##          df      AIC      BIC     AICc    logLik     LRT   pval        QE
## Full     10 610.0941 651.7902 610.5652 -295.0471               3364.5487
## Reduced   4 619.3467 636.0252 619.4313 -305.6734 21.2526 0.0017 4123.7388
```

```
Q1B.Perf_O3.reml <- rma.mv(yi = lnrr.yi, V = V.d2.shared.lnRR_Perf_O3,
    mods = ~Performance2 - 1, random = list(~1 | studyid, ~1 |
        infoid, ~1 | multipleoutcomecluster), data = metadat_Perf_O3,
    method = "REML")
summary(Q1B.Perf_O3.reml)
```

```
##
## Multivariate Meta-Analysis Model (k = 478; method: REML)
##
##    logLik   Deviance        AIC        BIC       AICc
## -292.8935   585.7870   605.7870   647.3356   606.2653
```

```
##
## Variance Components:
##
##             estim    sqrt  nlvls  fixed                  factor
## sigma^2.1  0.0888  0.2981     75     no                 studyid
## sigma^2.2  0.0459  0.2141    477     no                  infoid
## sigma^2.3  0.0248  0.1573    309     no  multipleoutcomecluster
##
## Test for Residual Heterogeneity:
## QE(df = 471) = 3364.5487, p-val < .0001
##
## Test of Moderators (coefficients 1:7):
## QM(df = 7) = 28.4010, p-val = 0.0002
##
## Model Results:
##
##                                   estimate      se     zval    pval    ci.lb
## Performance2abundance              -0.1557  0.0733  -2.1246  0.0336  -0.2994
## Performance2diversity               0.0096  0.1928   0.0497  0.9603  -0.3684
## Performance2feeding efficiency      0.0516  0.0594   0.8684  0.3852  -0.0649
## Performance2growth/development     -0.0312  0.0561  -0.5564  0.5779  -0.1413
## Performance2reproduction           -0.1848  0.0744  -2.4843  0.0130  -0.3307
## Performance2searching efficiency   -0.4130  0.1053  -3.9208  <.0001  -0.6194
## Performance2survival               -0.0879  0.0767  -1.1458  0.2519  -0.2383
##                                     ci.ub
## Performance2abundance             -0.0121    *
## Performance2diversity              0.3876
## Performance2feeding efficiency     0.1681
## Performance2growth/development     0.0788
## Performance2reproduction          -0.0390    *
## Performance2searching efficiency  -0.2065  ***
## Performance2survival               0.0625
##
## ---
## Signif. codes:  0 '***' 0.001 '**' 0.01 '*' 0.05 '.' 0.1 ' ' 1
```

```
r2_ml(Q1B.Perf_03.reml)
```

```
##    R2_marginal R2_conditional
##      0.1025507      0.7419256
```

RESPONSE MEASURE - NOx

```
metadat_Perf_NOx <- droplevels(subset(metadat_NOx, !(is.na(Performance2))))
# rerun VCV matrix
V.d2.shared.lnRR_Perf_NOx <- VCV.shared(data = metadat_Perf_NOx,
    cV = "cV.lnRR", V = "lnrr.vi", cluster = "sharedcontrolcluster",
    obs = "infoid")
# is.positive.definite(V.d2.shared.lnRR_Perf_NOx) # check
# the VCV is made correctly
```

```r
Q1B.null.Perf_NOx <- rma.mv(yi = lnrr.yi, V = V.d2.shared.lnRR_Perf_NOx,
    random = list(~1 | studyid, ~1 | infoid, ~1 | multipleoutcomecluster),
    data = metadat_Perf_NOx, method = "ML")
Q1B.Perf_NOx <- rma.mv(yi = lnrr.yi, V = V.d2.shared.lnRR_Perf_NOx,
    mods = ~Performance2 - 1, random = list(~1 | studyid, ~1 |
        infoid, ~1 | multipleoutcomecluster), data = metadat_Perf_NOx,
    method = "ML")
anova(Q1B.null.Perf_NOx, Q1B.Perf_NOx)
```

```
##
##         df      AIC      BIC     AICc   logLik     LRT   pval       QE
## Full     9 131.4037 154.4828 133.4967 -56.7018                677.9765
## Reduced  4 135.2745 145.5319 135.7141 -63.6373 13.8708 0.0165 861.6905
```

```r
Q1B.Perf_NOx.reml <- rma.mv(yi = lnrr.yi, V = V.d2.shared.lnRR_Perf_NOx,
    mods = ~Performance2 - 1, random = list(~1 | studyid, ~1 |
        infoid, ~1 | multipleoutcomecluster), data = metadat_Perf_NOx,
    method = "REML")
summary(Q1B.Perf_NOx.reml)
```

```
##
## Multivariate Meta-Analysis Model (k = 96; method: REML)
##
##   logLik  Deviance       AIC       BIC      AICc
## -52.8404  105.6807  123.6807  146.1790  125.9307
##
## Variance Components:
##
##             estim    sqrt  nlvls  fixed                  factor
## sigma^2.1  0.1127  0.3357     19     no                 studyid
## sigma^2.2  0.0030  0.0545     96     no                  infoid
## sigma^2.3  0.0901  0.3002     73     no  multipleoutcomecluster
##
## Test for Residual Heterogeneity:
## QE(df = 90) = 677.9765, p-val < .0001
##
## Test of Moderators (coefficients 1:6):
## QM(df = 6) = 15.3809, p-val = 0.0175
##
## Model Results:
##
##                                     estimate      se     zval    pval    ci.lb
## Performance2abundance                -0.0335  0.1533  -0.2185  0.8270  -0.3340
## Performance2diversity                -0.1456  0.2103  -0.6926  0.4886  -0.5578
## Performance2growth/development        0.0540  0.1562   0.3459  0.7294  -0.2521
## Performance2reproduction              0.1365  0.2620   0.5212  0.6022  -0.3769
## Performance2searching efficiency     -0.5726  0.1816  -3.1529  0.0016  -0.9285
## Performance2survival                 -2.3030  0.9529  -2.4169  0.0157  -4.1706
##                                      ci.ub
## Performance2abundance               0.2670
## Performance2diversity               0.2665
## Performance2growth/development      0.3601
```

```
## Performance2reproduction           0.6500
## Performance2searching efficiency  -0.2167  **
## Performance2survival              -0.4354   *
##
## ---
## Signif. codes:  0 '***' 0.001 '**' 0.01 '*' 0.05 '.' 0.1 ' ' 1
```

```
r2_ml(Q1B.Perf_NOx.reml)
```

```
##    R2_marginal R2_conditional
##      0.4837034      0.9925380
```

RESPONSE MEASURE - SO2

```
metadat_Perf_SO2 <- droplevels(subset(metadat_SO2, !(is.na(Performance2))))
# rerun VCV matrix
V.d2.shared.lnRR_Perf_SO2 <- VCV.shared(data = metadat_Perf_SO2,
    cV = "cV.lnRR", V = "lnrr.vi", cluster = "sharedcontrolcluster",
    obs = "infoid")
# is.positive.definite(V.d2.shared.lnRR_Perf_SO2) # check
# the VCV is made correctly

Q1B.null.Perf_SO2 <- rma.mv(yi = lnrr.yi, V = V.d2.shared.lnRR_Perf_SO2,
    random = list(~1 | studyid, ~1 | infoid, ~1 | multipleoutcomecluster),
    data = metadat_Perf_SO2, method = "ML")
Q1B.Perf_SO2 <- rma.mv(yi = lnrr.yi, V = V.d2.shared.lnRR_Perf_SO2,
    mods = ~Performance2 - 1, random = list(~1 | studyid, ~1 |
        infoid, ~1 | multipleoutcomecluster), data = metadat_Perf_SO2,
    method = "ML")
anova(Q1B.null.Perf_SO2, Q1B.Perf_SO2)
```

```
##
##         df      AIC      BIC     AICc    logLik     LRT   pval        QE
## Full    10 300.1887 335.2013 301.1289 -140.0944                2327.3299
## Reduced  4 299.3097 313.3147 299.4764 -145.6549 11.1210 0.0847 3118.9168
```

```
Q1B.Perf_SO2.reml <- rma.mv(yi = lnrr.yi, V = V.d2.shared.lnRR_Perf_SO2,
    mods = ~Performance2 - 1, random = list(~1 | studyid, ~1 |
        infoid, ~1 | multipleoutcomecluster), data = metadat_Perf_SO2,
    method = "REML")
summary(Q1B.Perf_SO2.reml)
```

```
##
## Multivariate Meta-Analysis Model (k = 245; method: REML)
##
##    logLik   Deviance        AIC        BIC       AICc
## -138.8409   277.6818   297.6818   332.4045   298.6509
##
## Variance Components:
##
```

```
##             estim    sqrt  nlvls  fixed                  factor
## sigma^2.1  0.0575  0.2398     32     no                 studyid
## sigma^2.2  0.0172  0.1310    245     no                  infoid
## sigma^2.3  0.1481  0.3848    160     no  multipleoutcomecluster
##
## Test for Residual Heterogeneity:
## QE(df = 238) = 2327.3299, p-val < .0001
##
## Test of Moderators (coefficients 1:7):
## QM(df = 7) = 11.3561, p-val = 0.1238
##
## Model Results:
##
##                                     estimate      se     zval    pval    ci.lb
## Performance2abundance                 0.0442  0.0952   0.4639  0.6427  -0.1424
## Performance2diversity                -0.3016  0.1492  -2.0218  0.0432  -0.5939
## Performance2feeding efficiency       -0.1481  0.1004  -1.4743  0.1404  -0.3449
## Performance2growth/development       -0.0041  0.0828  -0.0490  0.9609  -0.1664
## Performance2reproduction              0.0482  0.0939   0.5131  0.6079  -0.1359
## Performance2searching efficiency     -0.0152  0.3179  -0.0477  0.9619  -0.6383
## Performance2survival                 -0.0775  0.1003  -0.7723  0.4399  -0.2741
##                                       ci.ub
## Performance2abundance                0.2308
## Performance2diversity               -0.0092  *
## Performance2feeding efficiency       0.0488
## Performance2growth/development       0.1583
## Performance2reproduction             0.2322
## Performance2searching efficiency     0.6080
## Performance2survival                 0.1191
##
## ---
## Signif. codes:  0 '***' 0.001 '**' 0.01 '*' 0.05 '.' 0.1 ' ' 1
```

```
r2_ml(Q1B.Perf_SO2.reml)
```

```
##    R2_marginal R2_conditional
##     0.02737455     0.92505897
```

RESPONSE MEASURE - PM

```
metadat_Perf_PM <- droplevels(subset(metadat_PM, !(is.na(Performance2))))
# rerun VCV matrix
V.d2.shared.lnRR_Perf_PM <- VCV.shared(data = metadat_Perf_PM,
    cV = "cV.lnRR", V = "lnrr.vi", cluster = "sharedcontrolcluster",
    obs = "infoid")
# is.positive.definite(V.d2.shared.lnRR_Perf_PM) # check
# the VCV is made correctly

Q1B.null.Perf_PM <- rma.mv(yi = lnrr.yi, V = V.d2.shared.lnRR_Perf_PM,
    random = list(~1 | studyid, ~1 | infoid, ~1 | multipleoutcomecluster),
    data = metadat_Perf_PM, method = "ML")
```

```r
Q1B.Perf_PM <- rma.mv(yi = lnrr.yi, V = V.d2.shared.lnRR_Perf_PM,
    mods = ~Performance2 - 1, random = list(~1 | studyid, ~1 |
        infoid, ~1 | multipleoutcomecluster), data = metadat_Perf_PM,
    method = "ML")
anova(Q1B.null.Perf_PM, Q1B.Perf_PM)
```

```
##
##         df     AIC     BIC    AICc   logLik    LRT   pval       QE
## Full     9 73.1769 91.7209 76.9269 -27.5885               390.7760
## Reduced  4 68.2091 76.4509 68.9638 -30.1046 5.0322 0.4120 501.0757
```

```r
Q1B.Perf_PM.reml <- rma.mv(yi = lnrr.yi, V = V.d2.shared.lnRR_Perf_PM,
    mods = ~Performance2 - 1, random = list(~1 | studyid, ~1 |
        infoid, ~1 | multipleoutcomecluster), data = metadat_Perf_PM,
    method = "REML")
summary(Q1B.Perf_PM.reml)
```

```
##
## Multivariate Meta-Analysis Model (k = 58; method: REML)
##
##   logLik  Deviance       AIC       BIC      AICc
## -23.4566   46.9131   64.9131   82.4743   69.1988
##
## Variance Components:
##
##              estim    sqrt  nlvls  fixed                  factor
## sigma^2.1   0.3850  0.6205      8     no                 studyid
## sigma^2.2   0.0654  0.2557     57     no                  infoid
## sigma^2.3   0.0000  0.0000     20     no  multipleoutcomecluster
##
## Test for Residual Heterogeneity:
## QE(df = 52) = 390.7760, p-val < .0001
##
## Test of Moderators (coefficients 1:6):
## QM(df = 6) = 5.2482, p-val = 0.5124
##
## Model Results:
##
##                                    estimate      se     zval    pval    ci.lb
## Performance2abundance               -0.2496  0.3323  -0.7510  0.4527  -0.9010
## Performance2diversity               -0.5570  0.4235  -1.3154  0.1884  -1.3870
## Performance2feeding efficiency       0.1479  0.6259   0.2364  0.8131  -1.0788
## Performance2growth/development      -0.0372  0.6235  -0.0597  0.9524  -1.2594
## Performance2searching efficiency    -0.0410  0.5004  -0.0819  0.9347  -1.0217
## Performance2survival                -0.3842  0.7342  -0.5233  0.6008  -1.8232
##                                     ci.ub
## Performance2abundance              0.4018
## Performance2diversity              0.2729
## Performance2feeding efficiency     1.3747
## Performance2growth/development     1.1849
## Performance2searching efficiency   0.9397
## Performance2survival               1.0548
```

```
##
## ---
## Signif. codes:  0 '***' 0.001 '**' 0.01 '*' 0.05 '.' 0.1 ' ' 1
```

```r
r2_ml(Q1B.Perf_PM.reml)
```

```
##    R2_marginal R2_conditional
##     0.06266586     0.86388750
```

Supplementary Fig. 2

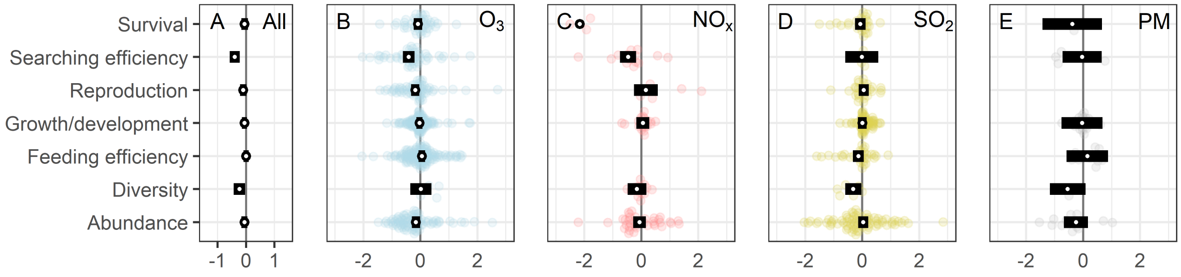

Supplementary Fig. 2. The effects of air pollution on the performance of invertebrates for the seven different performance metrics. Model estimates from an overall model across all pollutants (A) or individual pollutants (B-E). Air pollutants include ozone (O3) nitrogen oxides (NOx), sulfur dioxide (SO2) and particulate matter (PM). Points to the left of zero indicate negative impacts and points to the right indicate positive impacts. 95% confidence intervals (bold lines) overlapping the zero line indicate the mean estimate is not significantly different from zero (P < 0.05).

Plant Order

PLANT ORDER - O3

```r
# summary(metadat_O3$Plant.Order)
metadat_Plant.Order_O3 <- droplevels(subset(metadat_O3, !(is.na(Plant.Order))))
# rerun VCV matrix
V.d2.shared.lnRR_Plant.Order_O3 <- VCV.shared(data = metadat_Plant.Order_O3,
    cV = "cV.lnRR", V = "lnrr.vi", cluster = "sharedcontrolcluster",
    obs = "infoid")
# is.positive.definite(V.d2.shared.lnRR_Plant.Order_O3) #
# check the VCV is made correctly

Q1B.null.Plant.Order_O3 <- rma.mv(yi = lnrr.yi, V = V.d2.shared.lnRR_Plant.Order_O3,
    random = list(~1 | studyid, ~1 | infoid, ~1 | multipleoutcomecluster),
    data = metadat_Plant.Order_O3, method = "ML")
Q1B.Plant.Order_O3 <- rma.mv(yi = lnrr.yi, V = V.d2.shared.lnRR_Plant.Order_O3,
    mods = ~Plant.Order - 1, random = list(~1 | studyid, ~1 |
        infoid, ~1 | multipleoutcomecluster), data = metadat_Plant.Order_O3,
    method = "ML")
anova(Q1B.null.Plant.Order_O3, Q1B.Plant.Order_O3)
```

```
##
##         df      AIC      BIC     AICc    logLik     LRT   pval        QE
## Full    20 586.0884 667.8239 588.0931 -273.0442               3124.2650
## Reduced  4 574.2755 590.6226 574.3674 -283.1377 20.1871 0.2119 3949.0614
```

```
Q1B.Plant.Order_03.reml <- rma.mv(yi = lnrr.yi, V = V.d2.shared.lnRR_Plant.Order_03,
    mods = ~Plant.Order - 1, random = list(~1 | studyid, ~1 |
        infoid, ~1 | multipleoutcomecluster), data = metadat_Plant.Order_03,
    method = "REML")
summary(Q1B.Plant.Order_03.reml)
```

```
##
## Multivariate Meta-Analysis Model (k = 440; method: REML)
##
##    logLik   Deviance        AIC        BIC       AICc
## -262.6862   525.3723   565.3723   646.3198   567.4619
##
## Variance Components:
##
##              estim    sqrt  nlvls  fixed                  factor
## sigma^2.1  0.1096  0.3311     69     no                 studyid
## sigma^2.2  0.0526  0.2293    439     no                  infoid
## sigma^2.3  0.0185  0.1361    281     no  multipleoutcomecluster
##
## Test for Residual Heterogeneity:
## QE(df = 423) = 3124.2650, p-val < .0001
##
## Test of Moderators (coefficients 1:17):
## QM(df = 17) = 20.7608, p-val = 0.2372
##
## Model Results:
##
##                             estimate      se     zval    pval    ci.lb    ci.ub
## Plant.Order\tPinales          0.1000  0.5833   0.1714  0.8639  -1.0433   1.2433
## Plant.OrderAsterales          0.0279  0.4032   0.0692  0.9448  -0.7623   0.8181
## Plant.OrderBrassicales       -0.2936  0.1049  -2.7985  0.0051  -0.4992  -0.0880
## Plant.OrderCaryophyllales    -0.2725  0.2899  -0.9400  0.3472  -0.8406   0.2957
## Plant.OrderCucurbitales      -0.6847  0.3849  -1.7789  0.0753  -1.4390   0.0697
## Plant.OrderFabales            0.0713  0.1310   0.5445  0.5861  -0.1854   0.3281
## Plant.OrderFagales           -0.0180  0.1131  -0.1588  0.8738  -0.2397   0.2038
## Plant.OrderGentianales        0.1966  0.4437   0.4432  0.6577  -0.6731   1.0664
## Plant.OrderMagnoliales       -0.0020  0.3994  -0.0050  0.9960  -0.7848   0.7808
## Plant.OrderMalpighiales      -0.0703  0.1005  -0.6998  0.4840  -0.2673   0.1267
## Plant.OrderMalvales           0.1009  0.4593   0.2198  0.8260  -0.7993   1.0012
## Plant.OrderPinales           -0.0951  0.1491  -0.6379  0.5236  -0.3875   0.1972
## Plant.OrderPoales            -0.1204  0.1400  -0.8601  0.3897  -0.3947   0.1539
## Plant.OrderPolypodiales       0.9390  0.3761   2.4966  0.0125   0.2018   1.6761
## Plant.OrderRosales           -0.1470  0.3963  -0.3708  0.7108  -0.9238   0.6298
## Plant.OrderSapindales         0.0117  0.1821   0.0640  0.9490  -0.3452   0.3685
## Plant.OrderSolanales         -0.0298  0.1694  -0.1760  0.8603  -0.3619   0.3023
##
## Plant.Order\tPinales
## Plant.OrderAsterales
## Plant.OrderBrassicales     **
```

```
## Plant.OrderCaryophyllales
## Plant.OrderCucurbitales     .
## Plant.OrderFabales
## Plant.OrderFagales
## Plant.OrderGentianales
## Plant.OrderMagnoliales
## Plant.OrderMalpighiales
## Plant.OrderMalvales
## Plant.OrderPinales
## Plant.OrderPoales
## Plant.OrderPolypodiales     *
## Plant.OrderRosales
## Plant.OrderSapindales
## Plant.OrderSolanales
##
## ---
## Signif. codes:  0 '***' 0.001 '**' 0.01 '*' 0.05 '.' 0.1 ' ' 1
```

```r
r2_ml(Q1B.Plant.Order_O3.reml)
```

```
##    R2_marginal R2_conditional
##      0.1383572      0.7494059
```

PLANT ORDER - NOx

```r
# summary(metadat_NOx$Plant.Order)
metadat_NOx_Plant.Order <- droplevels(subset(metadat_NOx, Plant.Order !=
    "multiple"))
metadat_Plant.Order_NOx <- droplevels(subset(metadat_NOx_Plant.Order,
    !(is.na(Plant.Order))))
# rerun VCV matrix
V.d2.shared.lnRR_Plant.Order_NOx <- VCV.shared(data = metadat_Plant.Order_NOx,
    cV = "cV.lnRR", V = "lnrr.vi", cluster = "sharedcontrolcluster",
    obs = "infoid")
# is.positive.definite(V.d2.shared.lnRR_Plant.Order_NOx) #
# check the VCV is made correctly

Q1B.null.Plant.Order_NOx <- rma.mv(yi = lnrr.yi, V = V.d2.shared.lnRR_Plant.Order_NOx,
    random = list(~1 | studyid, ~1 | infoid, ~1 | multipleoutcomecluster),
    data = metadat_Plant.Order_NOx, method = "ML")
Q1B.Plant.Order_NOx <- rma.mv(yi = lnrr.yi, V = V.d2.shared.lnRR_Plant.Order_NOx,
    mods = ~Plant.Order - 1, random = list(~1 | studyid, ~1 |
        infoid, ~1 | multipleoutcomecluster), data = metadat_Plant.Order_NOx,
    method = "ML")
anova(Q1B.null.Plant.Order_NOx, Q1B.Plant.Order_NOx)
```

```
##
##         df     AIC     BIC    AICc   logLik     LRT   pval       QE
## Full    11 70.9012 95.7907 75.3758 -24.4506                515.9221
## Reduced  4 69.5089 78.5597 70.1150 -30.7545 12.6077 0.0823 670.6418
```

```r
Q1B.Plant.Order_NOx.reml <- rma.mv(yi = lnrr.yi, V = V.d2.shared.lnRR_Plant.Order_NOx,
    mods = ~Plant.Order - 1, random = list(~1 | studyid, ~1 |
        infoid, ~1 | multipleoutcomecluster), data = metadat_Plant.Order_NOx,
    method = "REML")
summary(Q1B.Plant.Order_NOx.reml)
```

```
##
## Multivariate Meta-Analysis Model (k = 71; method: REML)
##
##    logLik  Deviance       AIC       BIC      AICc
## -20.7938   41.5875   63.5875   87.1620   68.7640
##
## Variance Components:
##
##             estim    sqrt  nlvls  fixed                  factor
## sigma^2.1  0.1638  0.4048     15     no                 studyid
## sigma^2.2  0.0042  0.0649     71     no                  infoid
## sigma^2.3  0.0179  0.1338     53     no  multipleoutcomecluster
##
## Test for Residual Heterogeneity:
## QE(df = 63) = 515.9221, p-val < .0001
##
## Test of Moderators (coefficients 1:8):
## QM(df = 8) = 13.1753, p-val = 0.1060
##
## Model Results:
##
##                          estimate      se     zval    pval    ci.lb    ci.ub
## Plant.OrderBrassicales    -0.7941  0.2663  -2.9822  0.0029  -1.3159  -0.2722
## Plant.OrderDipsacales     -0.3685  0.5377  -0.6853  0.4931  -1.4224   0.6854
## Plant.OrderFabales         0.0175  0.2303   0.0762  0.9393  -0.4337   0.4688
## Plant.OrderFagales         0.3284  0.2641   1.2435  0.2137  -0.1892   0.8461
## Plant.OrderPinales         0.1694  0.2238   0.7571  0.4490  -0.2692   0.6081
## Plant.OrderPoales         -0.1383  0.2894  -0.4778  0.6328  -0.7055   0.4290
## Plant.OrderPolypodiales    0.3481  0.2672   1.3028  0.1926  -0.1756   0.8717
## Plant.OrderSolanales      -0.6549  0.5310  -1.2335  0.2174  -1.6956   0.3857
##
## Plant.OrderBrassicales   **
## Plant.OrderDipsacales
## Plant.OrderFabales
## Plant.OrderFagales
## Plant.OrderPinales
## Plant.OrderPoales
## Plant.OrderPolypodiales
## Plant.OrderSolanales
##
## ---
## Signif. codes:  0 '***' 0.001 '**' 0.01 '*' 0.05 '.' 0.1 ' ' 1
```

```r
r2_ml(Q1B.Plant.Order_NOx.reml)
```

```
##    R2_marginal R2_conditional
##      0.4406448      0.9873164
```

PLANT ORDER - SO2

```r
# summary(metadat_SO2$Plant.Order)
metadat_SO2_Plant.Order <- droplevels(subset(metadat_SO2, Plant.Order !=
    "multiple"))
metadat_Plant.Order_SO2 <- droplevels(subset(metadat_SO2_Plant.Order,
    !(is.na(Plant.Order))))
# rerun VCV matrix
V.d2.shared.lnRR_Plant.Order_SO2 <- VCV.shared(data = metadat_Plant.Order_SO2,
    cV = "cV.lnRR", V = "lnrr.vi", cluster = "sharedcontrolcluster",
    obs = "infoid")
# is.positive.definite(V.d2.shared.lnRR_Plant.Order_SO2) #
# check the VCV is made correctly

Q1B.null.Plant.Order_SO2 <- rma.mv(yi = lnrr.yi, V = V.d2.shared.lnRR_Plant.Order_SO2,
    random = list(~1 | studyid, ~1 | infoid, ~1 | multipleoutcomecluster),
    data = metadat_Plant.Order_SO2, method = "ML")
Q1B.Plant.Order_SO2 <- rma.mv(yi = lnrr.yi, V = V.d2.shared.lnRR_Plant.Order_SO2,
    mods = ~Plant.Order - 1, random = list(~1 | studyid, ~1 |
        infoid, ~1 | multipleoutcomecluster), data = metadat_Plant.Order_SO2,
    method = "ML")
anova(Q1B.null.Plant.Order_SO2, Q1B.Plant.Order_SO2)
```

```
##
##          df      AIC      BIC     AICc   logLik    LRT   pval        QE
## Full     10 167.6803 198.6790 169.1182 -73.8402               1791.3019
## Reduced   4 161.6876 174.0871 161.9392 -76.8438 6.0073 0.4224 1936.1633
```

```r
Q1B.Plant.Order_SO2.reml <- rma.mv(yi = lnrr.yi, V = V.d2.shared.lnRR_Plant.Order_SO2,
    mods = ~Plant.Order - 1, random = list(~1 | studyid, ~1 |
        infoid, ~1 | multipleoutcomecluster), data = metadat_Plant.Order_SO2,
    method = "REML")
summary(Q1B.Plant.Order_SO2.reml)
```

```
##
## Multivariate Meta-Analysis Model (k = 164; method: REML)
##
##   logLik  Deviance       AIC       BIC      AICc
## -68.4874  136.9748  156.9748  187.5372  158.4816
##
## Variance Components:
##
##              estim    sqrt  nlvls  fixed                  factor
## sigma^2.1   0.0607  0.2465     21     no                 studyid
## sigma^2.2   0.0100  0.1002    164     no                  infoid
## sigma^2.3   0.1767  0.4203     96     no  multipleoutcomecluster
##
## Test for Residual Heterogeneity:
## QE(df = 157) = 1791.3019, p-val < .0001
##
## Test of Moderators (coefficients 1:7):
## QM(df = 7) = 5.3965, p-val = 0.6117
```

```
##
## Model Results:
##
##                            estimate      se     zval    pval    ci.lb   ci.ub
## Plant.Order\tPinales        0.2641  0.2375   1.1120  0.2662  -0.2014  0.7296
## Plant.OrderBrassicales      0.1091  0.2517   0.4333  0.6648  -0.3842  0.6023
## Plant.OrderFabales         -0.0763  0.1544  -0.4943  0.6211  -0.3790  0.2263
## Plant.OrderFagales          0.2199  0.2192   1.0032  0.3158  -0.2097  0.6495
## Plant.OrderMalpighiales    -0.1160  0.1669  -0.6951  0.4870  -0.4431  0.2111
## Plant.OrderPinales          0.2023  0.1766   1.1457  0.2519  -0.1438  0.5484
## Plant.OrderPoales           0.4568  0.3798   1.2027  0.2291  -0.2876  1.2013
##
## ---
## Signif. codes:  0 '***' 0.001 '**' 0.01 '*' 0.05 '.' 0.1 ' ' 1
```

```
r2_ml(Q1B.Plant.Order_SO2.reml)
```

```
##    R2_marginal R2_conditional
##      0.1000869      0.9634534
```

PLANT ORDER - PM

```
# summary(metadat_PM$Plant.Order)
metadat_Plant.Order_PM <- droplevels(subset(metadat_PM, !(is.na(Plant.Order))))
# rerun VCV matrix
V.d2.shared.lnRR_Plant.Order_PM <- VCV.shared(data = metadat_Plant.Order_PM,
    cV = "cV.lnRR", V = "lnrr.vi", cluster = "sharedcontrolcluster",
    obs = "infoid")
# is.positive.definite(V.d2.shared.lnRR_Plant.Order_PM) #
# check the VCV is made correctly

Q1B.null.Plant.Order_PM <- rma.mv(yi = lnrr.yi, V = V.d2.shared.lnRR_Plant.Order_PM,
    random = list(~1 | studyid, ~1 | infoid, ~1 | multipleoutcomecluster),
    data = metadat_Plant.Order_PM, method = "ML")
Q1B.Plant.Order_PM <- rma.mv(yi = lnrr.yi, V = V.d2.shared.lnRR_Plant.Order_PM,
    mods = ~Plant.Order - 1, random = list(~1 | studyid, ~1 |
        infoid, ~1 | multipleoutcomecluster), data = metadat_Plant.Order_PM,
    method = "ML")
anova(Q1B.null.Plant.Order_PM, Q1B.Plant.Order_PM)
```

```
##
##         df     AIC     BIC    AICc   logLik    LRT   pval       QE
## Full     6 36.9057 47.7457 39.1163 -12.4529               303.8234
## Reduced  4 41.9213 49.1480 42.9213 -16.9607 9.0156 0.0110 327.6182
```

```
Q1B.Plant.Order_PM.reml <- rma.mv(yi = lnrr.yi, V = V.d2.shared.lnRR_Plant.Order_PM,
    mods = ~Plant.Order - 1, random = list(~1 | studyid, ~1 |
        infoid, ~1 | multipleoutcomecluster), data = metadat_Plant.Order_PM,
    method = "REML")
summary(Q1B.Plant.Order_PM.reml)
```

```
##
## Multivariate Meta-Analysis Model (k = 45; method: REML)
##
##   logLik  Deviance       AIC       BIC      AICc
## -12.4859   24.9718   36.9718   47.3979   39.3718
##
## Variance Components:
##
##              estim    sqrt  nlvls  fixed                  factor
## sigma^2.1  0.0236  0.1535      3     no                 studyid
## sigma^2.2  0.0578  0.2404     45     no                  infoid
## sigma^2.3  0.0000  0.0000      9     no  multipleoutcomecluster
##
## Test for Residual Heterogeneity:
## QE(df = 42) = 303.8234, p-val < .0001
##
## Test of Moderators (coefficients 1:3):
## QM(df = 3) = 11.4155, p-val = 0.0097
##
## Model Results:
##
##                       estimate      se     zval    pval    ci.lb    ci.ub
## Plant.OrderLamiales    -0.8544  0.3731  -2.2900  0.0220  -1.5856  -0.1231  *
## Plant.OrderRosales      0.0280  0.1606   0.1741  0.8618  -0.2868   0.3427
## Plant.OrderSolanales   -0.5406  0.2181  -2.4782  0.0132  -0.9681  -0.1130  *
##
## ---
## Signif. codes:  0 '***' 0.001 '**' 0.01 '*' 0.05 '.' 0.1 ' ' 1
```

```
r2_ml(Q1B.Plant.Order_PM.reml)
```

```
##    R2_marginal R2_conditional
##      0.4914578      0.6388314
```

Plant Family

PLANT FAMILY - O3

```
# summary(metadat_O3$Plant.Family)
metadat_Plant.Family_O3 <- droplevels(subset(metadat_O3, !(is.na(Plant.Family))))
# rerun VCV matrix
V.d2.shared.lnRR_Plant.Family_O3 <- VCV.shared(data = metadat_Plant.Family_O3,
    cV = "cV.lnRR", V = "lnrr.vi", cluster = "sharedcontrolcluster",
    obs = "infoid")
# is.positive.definite(V.d2.shared.lnRR_Plant.Family_O3) #
# check the VCV is made correctly

Q1B.null.Plant.Family_O3 <- rma.mv(yi = lnrr.yi, V = V.d2.shared.lnRR_Plant.Family_O3,
    random = list(~1 | studyid, ~1 | infoid, ~1 | multipleoutcomecluster),
    data = metadat_Plant.Family_O3, method = "ML")
Q1B.Plant.Family_O3 <- rma.mv(yi = lnrr.yi, V = V.d2.shared.lnRR_Plant.Family_O3,
```

```r
    mods = ~Plant.Family - 1, random = list(~1 | studyid, ~1 |
        infoid, ~1 | multipleoutcomecluster), data = metadat_Plant.Family_03,
    method = "ML")
anova(Q1B.null.Plant.Family_03, Q1B.Plant.Family_03)
```

```
##
##         df      AIC      BIC     AICc    logLik     LRT   pval        QE
## Full    21 582.5890 668.4113 584.7996 -270.2945                3079.8803
## Reduced  4 574.2755 590.6226 574.3674 -283.1377 25.6865 0.0804 3949.0614
```

```r
Q1B.Plant.Family_03.reml <- rma.mv(yi = lnrr.yi, V = V.d2.shared.lnRR_Plant.Family_03,
    mods = ~Plant.Family - 1, random = list(~1 | studyid, ~1 |
        infoid, ~1 | multipleoutcomecluster), data = metadat_Plant.Family_03,
    method = "REML")
summary(Q1B.Plant.Family_03.reml)
```

```
##
## Multivariate Meta-Analysis Model (k = 440; method: REML)
##
##    logLik   Deviance        AIC        BIC       AICc
## -259.3217   518.6433   560.6433   645.5884   562.9533
##
## Variance Components:
##
##              estim    sqrt  nlvls  fixed                  factor
## sigma^2.1   0.1207  0.3474     69     no                 studyid
## sigma^2.2   0.0522  0.2284    439     no                  infoid
## sigma^2.3   0.0162  0.1272    281     no  multipleoutcomecluster
##
## Test for Residual Heterogeneity:
## QE(df = 422) = 3079.8803, p-val < .0001
##
## Test of Moderators (coefficients 1:18):
## QM(df = 18) = 25.6007, p-val = 0.1092
##
## Model Results:
##
##                                 estimate      se     zval    pval    ci.lb
## Plant.FamilyAmaranthaceae        -0.4990  0.4083  -1.2222  0.2216  -1.2992
## Plant.FamilyApocynaceae           0.1964  0.4531   0.4335  0.6646  -0.6917
## Plant.FamilyAsteraceae            0.0278  0.4154   0.0669  0.9466  -0.7863
## Plant.FamilyBetulaceae            0.1527  0.1371   1.1138  0.2654  -0.1160
## Plant.FamilyBrassicaceae         -0.2964  0.1087  -2.7255  0.0064  -0.5095
## Plant.FamilyCucurbitaceae        -0.6854  0.3973  -1.7252  0.0845  -1.4640
## Plant.FamilyDennstaedtiaceae      0.9364  0.3899   2.4016  0.0163   0.1722
## Plant.FamilyFabaceae              0.0215  0.1371   0.1570  0.8752  -0.2472
## Plant.FamilyFagaceae             -0.4304  0.2119  -2.0312  0.0422  -0.8456
## Plant.FamilyMagnoliaceae         -0.0020  0.4113  -0.0050  0.9960  -0.8082
## Plant.FamilyMalvaceae             0.1039  0.4692   0.2215  0.8247  -0.8157
## Plant.FamilyPinaceae             -0.0830  0.1491  -0.5568  0.5776  -0.3753
## Plant.FamilyPoaceae              -0.1193  0.1453  -0.8214  0.4114  -0.4041
## Plant.FamilyPolygonaceae         -0.0081  0.4352  -0.0185  0.9852  -0.8610
```

```
## Plant.FamilyRosaceae          -0.1470  0.4083  -0.3601  0.7188  -0.9472
## Plant.FamilySalicaceae        -0.0151  0.1064  -0.1419  0.8872  -0.2235
## Plant.FamilySapindaceae        0.0471  0.1834   0.2569  0.7973  -0.3123
## Plant.FamilySolanaceae        -0.0317  0.1756  -0.1804  0.8569  -0.3759
##                                ci.ub
## Plant.FamilyAmaranthaceae      0.3012
## Plant.FamilyApocynaceae        1.0845
## Plant.FamilyAsteraceae         0.8419
## Plant.FamilyBetulaceae         0.4214
## Plant.FamilyBrassicaceae      -0.0832  **
## Plant.FamilyCucurbitaceae      0.0933   .
## Plant.FamilyDennstaedtiaceae   1.7005   *
## Plant.FamilyFabaceae           0.2903
## Plant.FamilyFagaceae          -0.0151   *
## Plant.FamilyMagnoliaceae       0.8041
## Plant.FamilyMalvaceae          1.0235
## Plant.FamilyPinaceae           0.2093
## Plant.FamilyPoaceae            0.1654
## Plant.FamilyPolygonaceae       0.8449
## Plant.FamilyRosaceae           0.6532
## Plant.FamilySalicaceae         0.1934
## Plant.FamilySapindaceae        0.4065
## Plant.FamilySolanaceae         0.3126
##
## ---
## Signif. codes:  0 '***' 0.001 '**' 0.01 '*' 0.05 '.' 0.1 ' ' 1
```

```
r2_ml(Q1B.Plant.Family_O3.reml)
```

```
##    R2_marginal R2_conditional
##      0.1718387      0.7714335
```

PLANT FAMILY - NOx

```
# summary(metadat_NOx$Plant.Family)
metadat_Plant.Family_NOx <- droplevels(subset(metadat_NOx, !(is.na(Plant.Family))))
# rerun VCV matrix
V.d2.shared.lnRR_Plant.Family_NOx <- VCV.shared(data = metadat_Plant.Family_NOx,
    cV = "cV.lnRR", V = "lnrr.vi", cluster = "sharedcontrolcluster",
    obs = "infoid")
# is.positive.definite(V.d2.shared.lnRR_Plant.Family_NOx) #
# check the VCV is made correctly

Q1B.null.Plant.Family_NOx <- rma.mv(yi = lnrr.yi, V = V.d2.shared.lnRR_Plant.Family_NOx,
    random = list(~1 | studyid, ~1 | infoid, ~1 | multipleoutcomecluster),
    data = metadat_Plant.Family_NOx, method = "ML")
Q1B.Plant.Family_NOx <- rma.mv(yi = lnrr.yi, V = V.d2.shared.lnRR_Plant.Family_NOx,
    mods = ~Plant.Family - 1, random = list(~1 | studyid, ~1 |
        infoid, ~1 | multipleoutcomecluster), data = metadat_Plant.Family_NOx,
    method = "ML")
anova(Q1B.null.Plant.Family_NOx, Q1B.Plant.Family_NOx)
```

```
##
##         df     AIC     BIC    AICc   logLik     LRT   pval       QE
## Full    11 70.9012 95.7907 75.3758 -24.4506               515.9221
## Reduced  4 69.5089 78.5597 70.1150 -30.7545 12.6077 0.0823 670.6418
```

```
Q1B.Plant.Family_NOx.reml <- rma.mv(yi = lnrr.yi, V = V.d2.shared.lnRR_Plant.Family_NOx,
    mods = ~Plant.Family - 1, random = list(~1 | studyid, ~1 |
        infoid, ~1 | multipleoutcomecluster), data = metadat_Plant.Family_NOx,
    method = "REML")
summary(Q1B.Plant.Family_NOx.reml)
```

```
##
## Multivariate Meta-Analysis Model (k = 71; method: REML)
##
##   logLik  Deviance       AIC       BIC      AICc
## -20.7938   41.5875   63.5875   87.1620   68.7640
##
## Variance Components:
##
##              estim    sqrt  nlvls  fixed                  factor
## sigma^2.1   0.1638  0.4048     15     no                 studyid
## sigma^2.2   0.0042  0.0649     71     no                  infoid
## sigma^2.3   0.0179  0.1338     53     no  multipleoutcomecluster
##
## Test for Residual Heterogeneity:
## QE(df = 63) = 515.9221, p-val < .0001
##
## Test of Moderators (coefficients 1:8):
## QM(df = 8) = 13.1753, p-val = 0.1060
##
## Model Results:
##
##                               estimate      se     zval    pval    ci.lb
## Plant.FamilyAdoxaceae          -0.3685  0.5377  -0.6853  0.4931  -1.4224
## Plant.FamilyBrassicaceae       -0.7941  0.2663  -2.9822  0.0029  -1.3159
## Plant.FamilyDennstaedtiaceae    0.3481  0.2672   1.3028  0.1926  -0.1756
## Plant.FamilyFabaceae            0.0175  0.2303   0.0762  0.9393  -0.4337
## Plant.FamilyFagaceae            0.3284  0.2641   1.2435  0.2137  -0.1892
## Plant.FamilyPinaceae            0.1694  0.2238   0.7571  0.4490  -0.2692
## Plant.FamilyPoaceae            -0.1383  0.2894  -0.4778  0.6328  -0.7055
## Plant.FamilySolanaceae         -0.6549  0.5310  -1.2335  0.2174  -1.6956
##                                 ci.ub
## Plant.FamilyAdoxaceae          0.6854
## Plant.FamilyBrassicaceae      -0.2722  **
## Plant.FamilyDennstaedtiaceae   0.8717
## Plant.FamilyFabaceae           0.4688
## Plant.FamilyFagaceae           0.8461
## Plant.FamilyPinaceae           0.6081
## Plant.FamilyPoaceae            0.4290
## Plant.FamilySolanaceae         0.3857
##
## ---
## Signif. codes:  0 '***' 0.001 '**' 0.01 '*' 0.05 '.' 0.1 ' ' 1
```

```
r2_ml(Q1B.Plant.Family_NOx.reml)
```

```
##    R2_marginal R2_conditional
##      0.4406448      0.9873164
```

PLANT FAMILY - SO2

```
# summary(metadat_SO2$Plant.Family)
metadat_SO2_Plant.Family <- droplevels(subset(metadat_SO2, Plant.Family !=
    "multiple"))
metadat_Plant.Family_SO2 <- droplevels(subset(metadat_SO2_Plant.Family,
    !(is.na(Plant.Family))))
# rerun VCV matrix
V.d2.shared.lnRR_Plant.Family_SO2 <- VCV.shared(data = metadat_Plant.Family_SO2,
    cV = "cV.lnRR", V = "lnrr.vi", cluster = "sharedcontrolcluster",
    obs = "infoid")
# is.positive.definite(V.d2.shared.lnRR_Plant.Family_SO2) #
# check the VCV is made correctly

Q1B.null.Plant.Family_SO2 <- rma.mv(yi = lnrr.yi, V = V.d2.shared.lnRR_Plant.Family_SO2,
    random = list(~1 | studyid, ~1 | infoid, ~1 | multipleoutcomecluster),
    data = metadat_Plant.Family_SO2, method = "ML")
Q1B.Plant.Family_SO2 <- rma.mv(yi = lnrr.yi, V = V.d2.shared.lnRR_Plant.Family_SO2,
    mods = ~Plant.Family - 1, random = list(~1 | studyid, ~1 |
        infoid, ~1 | multipleoutcomecluster), data = metadat_Plant.Family_SO2,
    method = "ML")
anova(Q1B.null.Plant.Family_SO2, Q1B.Plant.Family_SO2)
```

```
##
##         df      AIC      BIC     AICc    logLik    LRT   pval        QE
## Full     9 165.7009 193.5997 166.8698 -73.8505               1809.2499
## Reduced  4 161.6876 174.0871 161.9392 -76.8438 5.9867 0.3075 1936.1633
```

```
Q1B.Plant.Family_SO2.reml <- rma.mv(yi = lnrr.yi, V = V.d2.shared.lnRR_Plant.Family_SO2,
    mods = ~Plant.Family - 1, random = list(~1 | studyid, ~1 |
        infoid, ~1 | multipleoutcomecluster), data = metadat_Plant.Family_SO2,
    method = "REML")
summary(Q1B.Plant.Family_SO2.reml)
```

```
##
## Multivariate Meta-Analysis Model (k = 164; method: REML)
##
##   logLik  Deviance       AIC       BIC      AICc
## -69.1356  138.2712  156.2712  183.8345  157.4874
##
## Variance Components:
##
##              estim    sqrt  nlvls  fixed                  factor
## sigma^2.1  0.0572  0.2392     21     no                 studyid
## sigma^2.2  0.0101  0.1003    164     no                  infoid
```

```
## sigma^2.3  0.1746  0.4178     96     no  multipleoutcomecluster
##
## Test for Residual Heterogeneity:
## QE(df = 158) = 1809.2499, p-val < .0001
##
## Test of Moderators (coefficients 1:6):
## QM(df = 6) = 5.5124, p-val = 0.4800
##
## Model Results:
##
##                            estimate      se     zval    pval    ci.lb   ci.ub
## Plant.FamilyBetulaceae       0.2183  0.2156   1.0124  0.3113  -0.2043  0.6410
## Plant.FamilyBrassicaceae     0.1092  0.2473   0.4415  0.6589  -0.3755  0.5939
## Plant.FamilyFabaceae        -0.0769  0.1513  -0.5078  0.6116  -0.3735  0.2198
## Plant.FamilyPinaceae         0.2230  0.1551   1.4384  0.1503  -0.0809  0.5270
## Plant.FamilyPoaceae          0.4567  0.3745   1.2196  0.2226  -0.2772  1.1906
## Plant.FamilySalicaceae      -0.1132  0.1636  -0.6916  0.4892  -0.4339  0.2075
##
## ---
## Signif. codes:  0 '***' 0.001 '**' 0.01 '*' 0.05 '.' 0.1 ' ' 1
```

```r
r2_ml(Q1B.Plant.Family_SO2.reml)
```

```
##    R2_marginal R2_conditional
##     0.09956096     0.96256124
```

PLANT FAMILY - PM

```r
# summary(metadat_PM$Plant.Family)
metadat_Plant.Family_PM <- droplevels(subset(metadat_PM, !(is.na(Plant.Family))))
# rerun VCV matrix
V.d2.shared.lnRR_Plant.Family_PM <- VCV.shared(data = metadat_Plant.Family_PM,
    cV = "cV.lnRR", V = "lnrr.vi", cluster = "sharedcontrolcluster",
    obs = "infoid")
# is.positive.definite(V.d2.shared.lnRR_Plant.Family_PM) #
# check the VCV is made correctly

Q1B.null.Plant.Family_PM <- rma.mv(yi = lnrr.yi, V = V.d2.shared.lnRR_Plant.Family_PM,
    random = list(~1 | studyid, ~1 | infoid, ~1 | multipleoutcomecluster),
    data = metadat_Plant.Family_PM, method = "ML")
Q1B.Plant.Family_PM <- rma.mv(yi = lnrr.yi, V = V.d2.shared.lnRR_Plant.Family_PM,
    mods = ~Plant.Family - 1, random = list(~1 | studyid, ~1 |
        infoid, ~1 | multipleoutcomecluster), data = metadat_Plant.Family_PM,
    method = "ML")
anova(Q1B.null.Plant.Family_PM, Q1B.Plant.Family_PM)
```

```
##
##         df     AIC     BIC    AICc   logLik    LRT   pval       QE
## Full     6 36.9057 47.7457 39.1163 -12.4529               303.8234
## Reduced  4 41.9213 49.1480 42.9213 -16.9607 9.0156 0.0110 327.6182
```

```r
Q1B.Plant.Family_PM.reml <- rma.mv(yi = lnrr.yi, V = V.d2.shared.lnRR_Plant.Family_PM,
    mods = ~Plant.Family - 1, random = list(~1 | studyid, ~1 |
        infoid, ~1 | multipleoutcomecluster), data = metadat_Plant.Family_PM,
    method = "REML")
summary(Q1B.Plant.Family_PM.reml)
```

```
##
## Multivariate Meta-Analysis Model (k = 45; method: REML)
##
##    logLik   Deviance        AIC        BIC       AICc
## -12.4859    24.9718    36.9718    47.3979    39.3718
##
## Variance Components:
##
##             estim    sqrt  nlvls  fixed                  factor
## sigma^2.1  0.0236  0.1535      3     no                 studyid
## sigma^2.2  0.0578  0.2404     45     no                  infoid
## sigma^2.3  0.0000  0.0000      9     no  multipleoutcomecluster
##
## Test for Residual Heterogeneity:
## QE(df = 42) = 303.8234, p-val < .0001
##
## Test of Moderators (coefficients 1:3):
## QM(df = 3) = 11.4155, p-val = 0.0097
##
## Model Results:
##
##                         estimate      se     zval    pval    ci.lb    ci.ub
## Plant.FamilyLamiaceae    -0.8544  0.3731  -2.2900  0.0220  -1.5856  -0.1231  *
## Plant.FamilyRosaceae      0.0280  0.1606   0.1741  0.8618  -0.2868   0.3427
## Plant.FamilySolanaceae   -0.5406  0.2181  -2.4782  0.0132  -0.9681  -0.1130  *
##
## ---
## Signif. codes:  0 '***' 0.001 '**' 0.01 '*' 0.05 '.' 0.1 ' ' 1
```

```r
r2_ml(Q1B.Plant.Family_PM.reml)
```

```
##    R2_marginal R2_conditional
##      0.4914578      0.6388314
```

Annuality

ANNUALITY - O3

```r
metadat_Annuality_O3 <- droplevels(subset(metadat_O3, !(is.na(Annuality))))
# rerun VCV matrix
V.d2.shared.lnRR_Annuality_O3 <- VCV.shared(data = metadat_Annuality_O3,
    cV = "cV.lnRR", V = "lnrr.vi", cluster = "sharedcontrolcluster",
    obs = "infoid")
# is.positive.definite(V.d2.shared.lnRR_Annuality_O3) #
```

```r
# check the VCV is made correctly

Q1B.null.Annuality_03 <- rma.mv(yi = lnrr.yi, V = V.d2.shared.lnRR_Annuality_03,
    random = list(~1 | studyid, ~1 | infoid, ~1 | multipleoutcomecluster),
    data = metadat_Annuality_03, method = "ML")
Q1B.Annuality_03 <- rma.mv(yi = lnrr.yi, V = V.d2.shared.lnRR_Annuality_03,
    mods = ~Annuality - 1, random = list(~1 | studyid, ~1 | infoid,
        ~1 | multipleoutcomecluster), data = metadat_Annuality_03,
    method = "ML")
anova(Q1B.null.Annuality_03, Q1B.Annuality_03)
```

```
##
##         df      AIC      BIC     AICc    logLik    LRT   pval        QE
## Full     6 503.6194 527.6575 503.8299 -245.8097               3454.1806
## Reduced  4 500.5309 516.5564 500.6307 -246.2655 0.9115 0.6340 3517.3699
```

```r
Q1B.Annuality_03.reml <- rma.mv(yi = lnrr.yi, V = V.d2.shared.lnRR_Annuality_03,
    mods = ~Annuality - 1, random = list(~1 | studyid, ~1 | infoid,
        ~1 | multipleoutcomecluster), data = metadat_Annuality_03,
    method = "REML")
summary(Q1B.Annuality_03.reml)
```

```
##
## Multivariate Meta-Analysis Model (k = 406; method: REML)
##
##    logLik   Deviance        AIC        BIC       AICc
## -243.5371   487.0741   499.0741   523.0677   499.2862
##
## Variance Components:
##
##             estim    sqrt  nlvls  fixed                  factor
## sigma^2.1  0.1042  0.3228     65     no                 studyid
## sigma^2.2  0.0610  0.2469    405     no                  infoid
## sigma^2.3  0.0000  0.0000    259     no  multipleoutcomecluster
##
## Test for Residual Heterogeneity:
## QE(df = 403) = 3454.1806, p-val < .0001
##
## Test of Moderators (coefficients 1:3):
## QM(df = 3) = 6.3010, p-val = 0.0978
##
## Model Results:
##
##                      estimate      se     zval    pval    ci.lb   ci.ub
## Annualityannual       -0.1162  0.0732  -1.5887  0.1121  -0.2596  0.0272
## Annualitybiennial     -0.2902  0.2131  -1.3615  0.1733  -0.7080  0.1276
## Annualityperennial    -0.0891  0.0615  -1.4488  0.1474  -0.2096  0.0314
##
## ---
## Signif. codes:  0 '***' 0.001 '**' 0.01 '*' 0.05 '.' 0.1 ' ' 1
```

```r
r2_ml(Q1B.Annuality_O3.reml)
```

```
##    R2_marginal R2_conditional
##    0.007810635    0.633709531
```

ANNUALITY - NOx

```r
metadat_Annuality_NOx <- droplevels(subset(metadat_NOx, !(is.na(Annuality))))
# rerun VCV matrix
V.d2.shared.lnRR_Annuality_NOx <- VCV.shared(data = metadat_Annuality_NOx,
    cV = "cV.lnRR", V = "lnrr.vi", cluster = "sharedcontrolcluster",
    obs = "infoid")
# is.positive.definite(V.d2.shared.lnRR_Annuality_NOx) #
# check the VCV is made correctly

Q1B.null.Annuality_NOx <- rma.mv(yi = lnrr.yi, V = V.d2.shared.lnRR_Annuality_NOx,
    random = list(~1 | studyid, ~1 | infoid, ~1 | multipleoutcomecluster),
    data = metadat_Annuality_NOx, method = "ML")
Q1B.Annuality_NOx <- rma.mv(yi = lnrr.yi, V = V.d2.shared.lnRR_Annuality_NOx,
    mods = ~Annuality - 1, random = list(~1 | studyid, ~1 | infoid,
        ~1 | multipleoutcomecluster), data = metadat_Annuality_NOx,
    method = "ML")
anova(Q1B.null.Annuality_NOx, Q1B.Annuality_NOx)
```

```
##
##         df     AIC     BIC    AICc   logLik    LRT   pval       QE
## Full     5 69.3344 80.6478 70.2575 -29.6672               664.4897
## Reduced  4 69.5089 78.5597 70.1150 -30.7545 2.1745 0.1403 670.6418
```

```r
Q1B.Annuality_NOx.reml <- rma.mv(yi = lnrr.yi, V = V.d2.shared.lnRR_Annuality_NOx,
    mods = ~Annuality - 1, random = list(~1 | studyid, ~1 | infoid,
        ~1 | multipleoutcomecluster), data = metadat_Annuality_NOx,
    method = "REML")
summary(Q1B.Annuality_NOx.reml)
```

```
##
## Multivariate Meta-Analysis Model (k = 71; method: REML)
##
##   logLik  Deviance       AIC       BIC      AICc
## -27.5878   55.1755   65.1755   76.3461   66.1279
##
## Variance Components:
##
##             estim    sqrt  nlvls  fixed                  factor
## sigma^2.1  0.2307  0.4803     15     no                 studyid
## sigma^2.2  0.0042  0.0648     71     no                  infoid
## sigma^2.3  0.0171  0.1307     53     no  multipleoutcomecluster
##
## Test for Residual Heterogeneity:
## QE(df = 69) = 664.4897, p-val < .0001
```

```
##
## Test of Moderators (coefficients 1:2):
## QM(df = 2) = 3.3993, p-val = 0.1827
##
## Model Results:
##
##                       estimate      se     zval    pval    ci.lb   ci.ub
## Annualityannual        -0.3051  0.1707  -1.7870  0.0739  -0.6396  0.0295  .
## Annualityperennial      0.1050  0.2314   0.4537  0.6501  -0.3486  0.5585
##
## ---
## Signif. codes:  0 '***' 0.001 '**' 0.01 '*' 0.05 '.' 0.1 ' ' 1
```

```
r2_ml(Q1B.Annuality_NOx.reml)
```

```
##    R2_marginal R2_conditional
##      0.1427239      0.9856996
```

ANNUALITY - SO2

```
metadat_Annuality_SO2 <- droplevels(subset(metadat_SO2, !(is.na(Annuality))))
# rerun VCV matrix
V.d2.shared.lnRR_Annuality_SO2 <- VCV.shared(data = metadat_Annuality_SO2,
    cV = "cV.lnRR", V = "lnrr.vi", cluster = "sharedcontrolcluster",
    obs = "infoid")
# is.positive.definite(V.d2.shared.lnRR_Annuality_SO2) #
# check the VCV is made correctly

Q1B.null.Annuality_SO2 <- rma.mv(yi = lnrr.yi, V = V.d2.shared.lnRR_Annuality_SO2,
    random = list(~1 | studyid, ~1 | infoid, ~1 | multipleoutcomecluster),
    data = metadat_Annuality_SO2, method = "ML")
Q1B.Annuality_SO2 <- rma.mv(yi = lnrr.yi, V = V.d2.shared.lnRR_Annuality_SO2,
    mods = ~Annuality - 1, random = list(~1 | studyid, ~1 | infoid,
        ~1 | multipleoutcomecluster), data = metadat_Annuality_SO2,
    method = "ML")
anova(Q1B.null.Annuality_SO2, Q1B.Annuality_SO2)
```

```
##
##         df      AIC      BIC     AICc    logLik    LRT   pval        QE
## Full     5 169.3068 184.9858 169.6727 -79.6534               1898.2115
## Reduced  4 167.4069 179.9501 167.6493 -79.7034 0.1000 0.7518 1941.5044
```

```
Q1B.Annuality_SO2.reml <- rma.mv(yi = lnrr.yi, V = V.d2.shared.lnRR_Annuality_SO2,
    mods = ~Annuality - 1, random = list(~1 | studyid, ~1 | infoid,
        ~1 | multipleoutcomecluster), data = metadat_Annuality_SO2,
    method = "REML")
summary(Q1B.Annuality_SO2.reml)
```

```
##
## Multivariate Meta-Analysis Model (k = 170; method: REML)
```

```
##
##   logLik  Deviance       AIC       BIC      AICc
## -77.8984  155.7968  165.7968  181.4166  166.1671
##
## Variance Components:
##
##             estim    sqrt  nlvls  fixed                  factor
## sigma^2.1  0.0616  0.2483     22     no                 studyid
## sigma^2.2  0.0101  0.1004    170     no                  infoid
## sigma^2.3  0.1616  0.4020    102     no  multipleoutcomecluster
##
## Test for Residual Heterogeneity:
## QE(df = 168) = 1898.2115, p-val < .0001
##
## Test of Moderators (coefficients 1:2):
## QM(df = 2) = 0.5219, p-val = 0.7703
##
## Model Results:
##
##                      estimate      se    zval    pval    ci.lb   ci.ub
## Annualityannual        0.0278  0.1228  0.2266  0.8207  -0.2129  0.2685
## Annualityperennial     0.0663  0.0966  0.6860  0.4927  -0.1231  0.2557
##
## ---
## Signif. codes:  0 '***' 0.001 '**' 0.01 '*' 0.05 '.' 0.1 ' ' 1
```

```
r2_ml(Q1B.Annuality_SO2.reml)
```

```
##    R2_marginal R2_conditional
##    0.001529291    0.956871117
```

ANNUALITY - PM

```
metadat_Annuality_PM <- droplevels(subset(metadat_PM, !(is.na(Annuality))))
# rerun VCV matrix
V.d2.shared.lnRR_Annuality_PM <- VCV.shared(data = metadat_Annuality_PM,
    cV = "cV.lnRR", V = "lnrr.vi", cluster = "sharedcontrolcluster",
    obs = "infoid")
# is.positive.definite(V.d2.shared.lnRR_Annuality_PM) #
# check the VCV is made correctly

Q1B.null.Annuality_PM <- rma.mv(yi = lnrr.yi, V = V.d2.shared.lnRR_Annuality_PM,
    random = list(~1 | studyid, ~1 | infoid, ~1 | multipleoutcomecluster),
    data = metadat_Annuality_PM, method = "ML")
Q1B.Annuality_PM <- rma.mv(yi = lnrr.yi, V = V.d2.shared.lnRR_Annuality_PM,
    mods = ~Annuality - 1, random = list(~1 | studyid, ~1 | infoid,
        ~1 | multipleoutcomecluster), data = metadat_Annuality_PM,
    method = "ML")
anova(Q1B.null.Annuality_PM, Q1B.Annuality_PM)
```

```
##
```

```
##         df     AIC     BIC    AICc   logLik    LRT   pval       QE
## Full     5 41.5611 50.5944 43.0996 -15.7806               311.0574
## Reduced  4 41.9213 49.1480 42.9213 -16.9607 2.3602 0.1245 327.6182
```

```
Q1B.Annuality_PM.reml <- rma.mv(yi = lnrr.yi, V = V.d2.shared.lnRR_Annuality_PM,
    mods = ~Annuality - 1, random = list(~1 | studyid, ~1 | infoid,
        ~1 | multipleoutcomecluster), data = metadat_Annuality_PM,
    method = "REML")
summary(Q1B.Annuality_PM.reml)
```

```
##
## Multivariate Meta-Analysis Model (k = 45; method: REML)
##
##   logLik  Deviance       AIC       BIC      AICc
## -14.2889   28.5779   38.5779   47.3839   40.1995
##
## Variance Components:
##
##             estim    sqrt  nlvls  fixed                  factor
## sigma^2.1  0.3303  0.5747      3     no                 studyid
## sigma^2.2  0.0577  0.2401     45     no                  infoid
## sigma^2.3  0.0000  0.0000      9     no  multipleoutcomecluster
##
## Test for Residual Heterogeneity:
## QE(df = 43) = 311.0574, p-val < .0001
##
## Test of Moderators (coefficients 1:2):
## QM(df = 2) = 1.4637, p-val = 0.4810
##
## Model Results:
##
##                      estimate      se     zval    pval    ci.lb   ci.ub
## Annualityannual       -0.5405  0.5952  -0.9080  0.3639  -1.7071  0.6262
## Annualityperennial    -0.3490  0.4364  -0.7996  0.4240  -1.2044  0.5064
##
## ---
## Signif. codes:  0 '***' 0.001 '**' 0.01 '*' 0.05 '.' 0.1 ' ' 1
```

```
r2_ml(Q1B.Annuality_PM.reml)
```

```
##    R2_marginal R2_conditional
##      0.0110453      0.8530047
```

Plant type

MONOCOT_DICOT - O3

```
metadat_Monocot_Dicot_O3 <- droplevels(subset(metadat_O3, !(is.na(Monocot_Dicot))))
# rerun VCV matrix
V.d2.shared.lnRR_Monocot_Dicot_O3 <- VCV.shared(data = metadat_Monocot_Dicot_O3,
```

```r
    cV = "cV.lnRR", V = "lnrr.vi", cluster = "sharedcontrolcluster",
    obs = "infoid")
# is.positive.definite(V.d2.shared.lnRR_Monocot_Dicot_03) #
# check the VCV is made correctly

Q1B.null.Plant.type_03 <- rma.mv(yi = lnrr.yi, V = V.d2.shared.lnRR_Monocot_Dicot_03,
    random = list(~1 | studyid, ~1 | infoid, ~1 | multipleoutcomecluster),
    data = metadat_Monocot_Dicot_03, method = "ML")
Q1B.Plant.type_03 <- rma.mv(yi = lnrr.yi, V = V.d2.shared.lnRR_Monocot_Dicot_03,
    mods = ~Monocot_Dicot - 1, random = list(~1 | studyid, ~1 |
        infoid, ~1 | multipleoutcomecluster), data = metadat_Monocot_Dicot_03,
    method = "ML")
anova(Q1B.null.Plant.type_03, Q1B.Plant.type_03)
```

```
##
##         df      AIC      BIC     AICc    logLik    LRT   pval        QE
## Full     6 556.5188 580.9709 556.7151 -272.2594               3863.1371
## Reduced  4 552.5760 568.8774 552.6690 -272.2880 0.0572 0.9718 3888.1282
```

```r
Q1B.Plant.type_03.reml <- rma.mv(yi = lnrr.yi, V = V.d2.shared.lnRR_Monocot_Dicot_03,
    mods = ~Monocot_Dicot - 1, random = list(~1 | studyid, ~1 |
        infoid, ~1 | multipleoutcomecluster), data = metadat_Monocot_Dicot_03,
    method = "REML")
summary(Q1B.Plant.type_03.reml)
```

```
##
## Multivariate Meta-Analysis Model (k = 435; method: REML)
##
##    logLik   Deviance        AIC        BIC       AICc
## -269.9138   539.8276   551.8276   576.2381   552.0252
##
## Variance Components:
##
##               estim    sqrt  nlvls  fixed                  factor
## sigma^2.1    0.0904  0.3006     68     no                 studyid
## sigma^2.2    0.0531  0.2304    434     no                  infoid
## sigma^2.3    0.0167  0.1292    276     no  multipleoutcomecluster
##
## Test for Residual Heterogeneity:
## QE(df = 432) = 3863.1371, p-val < .0001
##
## Test of Moderators (coefficients 1:3):
## QM(df = 3) = 6.4371, p-val = 0.0922
##
## Model Results:
##
##                            estimate      se     zval    pval    ci.lb    ci.ub
## Monocot_Dicotdicot          -0.1148  0.0501  -2.2934  0.0218  -0.2129  -0.0167  *
## Monocot_Dicotgymnosperm     -0.0819  0.1344  -0.6092  0.5424  -0.3453   0.1816
## Monocot_Dicotmonocot        -0.1105  0.1231  -0.8978  0.3693  -0.3517   0.1307
##
## ---
## Signif. codes:  0 '***' 0.001 '**' 0.01 '*' 0.05 '.' 0.1 ' ' 1
```

```r
r2_ml(Q1B.Plant.type_O3.reml)
```

```
##    R2_marginal R2_conditional
##   0.0005831786   0.6687536357
```

MONOCOT_DICOT - NOx

```r
metadat_Monocot_Dicot_NOx <- droplevels(subset(metadat_NOx, !(is.na(Monocot_Dicot))))
# rerun VCV matrix
V.d2.shared.lnRR_Monocot_Dicot_NOx <- VCV.shared(data = metadat_Monocot_Dicot_NOx,
    cV = "cV.lnRR", V = "lnrr.vi", cluster = "sharedcontrolcluster",
    obs = "infoid")
# is.positive.definite(V.d2.shared.lnRR_Monocot_Dicot_NOx)
# # check the VCV is made correctly

Q1B.null.Plant.type_NOx <- rma.mv(yi = lnrr.yi, V = V.d2.shared.lnRR_Monocot_Dicot_NOx,
    random = list(~1 | studyid, ~1 | infoid, ~1 | multipleoutcomecluster),
    data = metadat_Monocot_Dicot_NOx, method = "ML")
Q1B.Plant.type_NOx <- rma.mv(yi = lnrr.yi, V = V.d2.shared.lnRR_Monocot_Dicot_NOx,
    mods = ~Monocot_Dicot - 1, random = list(~1 | studyid, ~1 |
        infoid, ~1 | multipleoutcomecluster), data = metadat_Monocot_Dicot_NOx,
    method = "ML")
anova(Q1B.null.Plant.type_NOx, Q1B.Plant.type_NOx)
```

```
##
##         df     AIC     BIC    AICc   logLik    LRT   pval       QE
## Full     6 73.2783 86.8543 74.5908 -30.6391               595.5639
## Reduced  4 69.5089 78.5597 70.1150 -30.7545 0.2307 0.8911 670.6418
```

```r
Q1B.Plant.type_NOx.reml <- rma.mv(yi = lnrr.yi, V = V.d2.shared.lnRR_Monocot_Dicot_NOx,
    mods = ~Monocot_Dicot - 1, random = list(~1 | studyid, ~1 |
        infoid, ~1 | multipleoutcomecluster), data = metadat_Monocot_Dicot_NOx,
    method = "REML")
summary(Q1B.Plant.type_NOx.reml)
```

```
##
## Multivariate Meta-Analysis Model (k = 71; method: REML)
##
##   logLik  Deviance       AIC       BIC      AICc
## -28.0663   56.1326   68.1326   81.4497   69.5097
##
## Variance Components:
##
##              estim    sqrt  nlvls  fixed                  factor
## sigma^2.1   0.2554  0.5054     15     no                 studyid
## sigma^2.2   0.0039  0.0627     71     no                  infoid
## sigma^2.3   0.0185  0.1358     53     no  multipleoutcomecluster
##
## Test for Residual Heterogeneity:
## QE(df = 68) = 595.5639, p-val < .0001
```

```
##
## Test of Moderators (coefficients 1:3):
## QM(df = 3) = 1.3896, p-val = 0.7080
##
## Model Results:
##
##                              estimate      se     zval    pval    ci.lb   ci.ub
## Monocot_Dicotdicot            -0.1808  0.1627  -1.1112  0.2665  -0.4997  0.1381
## Monocot_Dicotgymnosperm       -0.1242  0.1897  -0.6544  0.5129  -0.4960  0.2477
## Monocot_Dicotmonocot          -0.1385  0.3599  -0.3848  0.7004  -0.8440  0.5670
##
## ---
## Signif. codes:  0 '***' 0.001 '**' 0.01 '*' 0.05 '.' 0.1 ' ' 1
```

```r
r2_ml(Q1B.Plant.type_NOx.reml)
```

```
##    R2_marginal R2_conditional
##     0.00232903     0.98590136
```

MONOCOT_DICOT - SO2

```r
metadat_Monocot_Dicot_SO2 <- droplevels(subset(metadat_SO2, !(is.na(Monocot_Dicot))))
# rerun VCV matrix
V.d2.shared.lnRR_Monocot_Dicot_SO2 <- VCV.shared(data = metadat_Monocot_Dicot_SO2,
    cV = "cV.lnRR", V = "lnrr.vi", cluster = "sharedcontrolcluster",
    obs = "infoid")
# is.positive.definite(V.d2.shared.lnRR_Monocot_Dicot_SO2)
# # check the VCV is made correctly

Q1B.null.Plant.type_SO2 <- rma.mv(yi = lnrr.yi, V = V.d2.shared.lnRR_Monocot_Dicot_SO2,
    random = list(~1 | studyid, ~1 | infoid, ~1 | multipleoutcomecluster),
    data = metadat_Monocot_Dicot_SO2, method = "ML")
Q1B.Plant.type_SO2 <- rma.mv(yi = lnrr.yi, V = V.d2.shared.lnRR_Monocot_Dicot_SO2,
    mods = ~Monocot_Dicot - 1, random = list(~1 | studyid, ~1 |
        infoid, ~1 | multipleoutcomecluster), data = metadat_Monocot_Dicot_SO2,
    method = "ML")
anova(Q1B.null.Plant.type_SO2, Q1B.Plant.type_SO2)
```

```
##
##         df      AIC      BIC     AICc   logLik    LRT   pval        QE
## Full     6 167.2498 186.0646 167.7652 -77.6249               1853.2089
## Reduced  4 167.4069 179.9501 167.6493 -79.7034 4.1570 0.1251 1941.5044
```

```r
Q1B.Plant.type_SO2.reml <- rma.mv(yi = lnrr.yi, V = V.d2.shared.lnRR_Monocot_Dicot_SO2,
    mods = ~Monocot_Dicot - 1, random = list(~1 | studyid, ~1 |
        infoid, ~1 | multipleoutcomecluster), data = metadat_Monocot_Dicot_SO2,
    method = "REML")
summary(Q1B.Plant.type_SO2.reml)
```

```
##
```

```
## Multivariate Meta-Analysis Model (k = 170; method: REML)
##
##   logLik  Deviance       AIC       BIC      AICc
## -75.4361  150.8722  162.8722  181.5801  163.3972
##
## Variance Components:
##
##             estim    sqrt  nlvls  fixed                  factor
## sigma^2.1  0.0401  0.2003     22     no                 studyid
## sigma^2.2  0.0100  0.1002    170     no                  infoid
## sigma^2.3  0.1650  0.4062    102     no  multipleoutcomecluster
##
## Test for Residual Heterogeneity:
## QE(df = 167) = 1853.2089, p-val < .0001
##
## Test of Moderators (coefficients 1:3):
## QM(df = 3) = 4.5297, p-val = 0.2097
##
## Model Results:
##
##                           estimate      se     zval    pval    ci.lb   ci.ub
## Monocot_Dicotdicot         -0.0283  0.0801  -0.3535  0.7237  -0.1854  0.1287
## Monocot_Dicotgymnosperm     0.2313  0.1413   1.6377  0.1015  -0.0455  0.5082
## Monocot_Dicotmonocot        0.4560  0.3474   1.3125  0.1894  -0.2249  1.1368
##
## ---
## Signif. codes:  0 '***' 0.001 '**' 0.01 '*' 0.05 '.' 0.1 ' ' 1
```

```
r2_ml(Q1B.Plant.type_SO2.reml)
```

```
##    R2_marginal R2_conditional
##     0.07436941     0.95680528
```

MONOCOT_DICOT - PM

```
metadat_Monocot_Dicot_PM <- droplevels(subset(metadat_PM, !(is.na(Monocot_Dicot))))
# rerun VCV matrix
V.d2.shared.lnRR_Monocot_Dicot_PM <- VCV.shared(data = metadat_Monocot_Dicot_PM,
    cV = "cV.lnRR", V = "lnrr.vi", cluster = "sharedcontrolcluster",
    obs = "infoid")
# is.positive.definite(V.d2.shared.lnRR_Monocot_Dicot_PM) #
# check the VCV is made correctly

Q1B.null.Plant.type_PM <- rma.mv(yi = lnrr.yi, V = V.d2.shared.lnRR_Monocot_Dicot_PM,
    random = list(~1 | studyid, ~1 | infoid, ~1 | multipleoutcomecluster),
    data = metadat_Monocot_Dicot_PM, method = "ML")
Q1B.Plant.type_PM <- rma.mv(yi = lnrr.yi, V = V.d2.shared.lnRR_Monocot_Dicot_PM,
    mods = ~Monocot_Dicot - 1, random = list(~1 | studyid, ~1 |
        infoid, ~1 | multipleoutcomecluster), data = metadat_Monocot_Dicot_PM,
    method = "ML")
anova(Q1B.null.Plant.type_PM, Q1B.Plant.type_PM)
```

```
##
##         df     AIC     BIC    AICc   logLik    LRT   pval       QE
## Full     5 42.1528 51.1861 43.6913 -16.0764               320.5651
## Reduced  4 41.9213 49.1480 42.9213 -16.9607 1.7685 0.1836 327.6182
```

```
Q1B.Plant.type_PM.reml <- rma.mv(yi = lnrr.yi, V = V.d2.shared.lnRR_Monocot_Dicot_PM,
    mods = ~Monocot_Dicot - 1, random = list(~1 | studyid, ~1 |
        infoid, ~1 | multipleoutcomecluster), data = metadat_Monocot_Dicot_PM,
    method = "REML")
summary(Q1B.Plant.type_PM.reml)
```

```
##
## Multivariate Meta-Analysis Model (k = 45; method: REML)
##
##   logLik  Deviance       AIC       BIC      AICc
## -14.1582   28.3164   38.3164   47.1224   39.9380
##
## Variance Components:
##
##             estim    sqrt  nlvls  fixed                  factor
## sigma^2.1  0.1481  0.3848      3     no                 studyid
## sigma^2.2  0.0571  0.2389     45     no                  infoid
## sigma^2.3  0.0000  0.0000      9     no  multipleoutcomecluster
##
## Test for Residual Heterogeneity:
## QE(df = 43) = 320.5651, p-val < .0001
##
## Test of Moderators (coefficients 1:2):
## QM(df = 2) = 3.4704, p-val = 0.1764
##
## Model Results:
##
##                          estimate      se     zval    pval    ci.lb   ci.ub
## Monocot_Dicotdicot        -0.2370  0.2832  -0.8367  0.4027  -0.7920  0.3181
## Monocot_Dicotmonocot      -0.8543  0.5133  -1.6644  0.0960  -1.8604  0.1517   .
##
## ---
## Signif. codes:  0 '***' 0.001 '**' 0.01 '*' 0.05 '.' 0.1 ' ' 1
```

```
r2_ml(Q1B.Plant.type_PM.reml)
```

```
##    R2_marginal R2_conditional
##      0.1057157      0.7512056
```

4.1. Pest status: are pests or beneficial invertebrates more affected by air pollution?

Status - O3

```r
#summary(metadat_03$Status)
metadat_03_status <- droplevels(subset(metadat_03,!(is.na(Status))))
# rerun VCV matrix
V.d2.shared.lnRR_03_status <- VCV.shared(data=metadat_03_status,
                                         cV="cV.lnRR", #covariance
                                         V = "lnrr.vi", #variance
                                         cluster="sharedcontrolcluster", #clustering factor
                                         obs="infoid") # each data row has a unique infoid
#is.positive.definite(V.d2.shared.lnRR_03_status) # check the VCV is made correctly

Q2.03.null <- rma.mv(yi = lnrr.yi,
                     V = V.d2.shared.lnRR_03_status,
                     random = list(~1 |studyid, ~1 | infoid, ~1 | multipleoutcomecluster),
                     data = metadat_03_status, method = "ML")
#summary(Q2.03.null)
Q2.03 <- rma.mv(yi = lnrr.yi,
                V = V.d2.shared.lnRR_03_status,
                mods = ~ Status -1,
                random = list(~1 |studyid, ~1 | infoid, ~1 | multipleoutcomecluster),
                data = metadat_03_status, method = "ML")

# re-run with reml
Q2.03.reml <- rma.mv(yi = lnrr.yi,
                V = V.d2.shared.lnRR_03_status,
                mods = ~ Status -1,
                random = list(~1 |studyid, ~1 | infoid, ~1 | multipleoutcomecluster),
                data = metadat_03_status, method = "REML")

anova(Q2.03.null,Q2.03)
```

```
##
##         df      AIC      BIC     AICc    logLik     LRT   pval        QE
## Full     6 606.9751 631.9042 607.1561 -297.4875               4017.1887
## Reduced  4 621.9917 638.6111 622.0775 -306.9958 19.0166 <.0001 4100.9231
```

```r
summary(Q2.03.reml)
```

```
##
## Multivariate Meta-Analysis Model (k = 471; method: REML)
##
##    logLik   Deviance        AIC        BIC       AICc
## -295.5229   591.0457   603.0457   627.9365   603.2279
##
## Variance Components:
##
##             estim    sqrt  nlvls  fixed                  factor
## sigma^2.1  0.0834  0.2887     75     no                 studyid
## sigma^2.2  0.0525  0.2291    470     no                  infoid
## sigma^2.3  0.0193  0.1390    303     no  multipleoutcomecluster
##
## Test for Residual Heterogeneity:
## QE(df = 468) = 4017.1887, p-val < .0001
```

```
##
## Test of Moderators (coefficients 1:3):
## QM(df = 3) = 26.6220, p-val < .0001
##
## Model Results:
##
##                          estimate      se     zval    pval    ci.lb    ci.ub
## StatusBeneficial          -0.4312  0.0837  -5.1515  <.0001  -0.5953  -0.2672
## StatusOther herbivore     -0.0262  0.0791  -0.3317  0.7401  -0.1812   0.1288
## StatusSignificant pest    -0.0521  0.0494  -1.0548  0.2915  -0.1488   0.0447
##
## StatusBeneficial         ***
## StatusOther herbivore
## StatusSignificant pest
##
## ---
## Signif. codes:  0 '***' 0.001 '**' 0.01 '*' 0.05 '.' 0.1 ' ' 1
```

```
r2_ml(Q2.03.reml)
```

```
##    R2_marginal R2_conditional
##      0.1132225      0.7000522
```

Status - NOx

```
metadat_NOx_status <- droplevels(subset(metadat_NOx,!(is.na(Status))))
# rerun VCV MATRIX
V.d2.shared.lnRR_NOx_status <- VCV.shared(data=metadat_NOx_status,
                                          cV="cV.lnRR", #covariance
                                          V = "lnrr.vi", #variance
                                          cluster="sharedcontrolcluster", #clustering factor
                                          obs="infoid") # each data row has a unique infoid
#is.positive.definite(V.d2.shared.lnRR_NOx_status) # check the VCV is made correctly

Q2.NOx.null <- rma.mv(yi = lnrr.yi,
                      V = V.d2.shared.lnRR_NOx_status,
                      random = list(~1 |studyid, ~1 | infoid, ~1 | multipleoutcomecluster),
                      data = metadat_NOx_status, method = "ML")
#summary(Q2.NOx.null)
Q2.NOx <- rma.mv(yi = lnrr.yi,
                 V = V.d2.shared.lnRR_NOx_status,
                 mods = ~ Status -1,
                 random = list(~1 |studyid, ~1 | infoid, ~1 | multipleoutcomecluster),
                 data = metadat_NOx_status, method = "ML")

# fit with reml
Q2.NOx.reml <- rma.mv(yi = lnrr.yi,
                 V = V.d2.shared.lnRR_NOx_status,
                 mods = ~ Status -1,
                 random = list(~1 |studyid, ~1 | infoid, ~1 | multipleoutcomecluster),
                 data = metadat_NOx_status, method = "REML")
```

```r
anova(Q2.NOx.null,Q2.NOx)
```

```
##
##         df      AIC      BIC     AICc   logLik    LRT   pval       QE
## Full     6 131.9080 146.8398 132.9324 -59.9540              671.2432
## Reduced  4 136.1610 146.1155 136.6371 -64.0805 8.2529 0.0161 757.5474
```

```r
summary(Q2.NOx.reml)
```

```
##
## Multivariate Meta-Analysis Model (k = 89; method: REML)
##
##   logLik  Deviance       AIC       BIC      AICc
## -59.4599  118.9197  130.9197  145.6458  131.9830
##
## Variance Components:
##
##             estim    sqrt  nlvls  fixed                  factor
## sigma^2.1  0.0000  0.0000     19     no                 studyid
## sigma^2.2  0.0000  0.0001     89     no                  infoid
## sigma^2.3  0.1256  0.3544     67     no  multipleoutcomecluster
##
## Test for Residual Heterogeneity:
## QE(df = 86) = 671.2432, p-val < .0001
##
## Test of Moderators (coefficients 1:3):
## QM(df = 3) = 9.3142, p-val = 0.0254
##
## Model Results:
##
##                           estimate      se     zval    pval    ci.lb    ci.ub
## StatusBeneficial           -0.2713  0.0929  -2.9193  0.0035  -0.4535  -0.0892  **
## StatusOther herbivore       0.0066  0.0951   0.0696  0.9445  -0.1799   0.1931
## StatusSignificant pest      0.0685  0.0773   0.8870  0.3751  -0.0829   0.2200
##
## ---
## Signif. codes:  0 '***' 0.001 '**' 0.01 '*' 0.05 '.' 0.1 ' ' 1
```

```r
r2_ml(Q2.NOx.reml)
```

```
##    R2_marginal R2_conditional
##      0.1396936      0.9999999
```

```r
summary(Q2.NOx.reml)$b
```

```
##                                [,1]
## StatusBeneficial       -0.271305497
## StatusOther herbivore   0.006620252
## StatusSignificant pest  0.068533865
```

```r
1-exp(-0.271305497) # 23.7% reduction
```

```
## [1] 0.2376164
```

Status - SO2

```r
metadat_SO2_status <- droplevels(subset(metadat_SO2,!(is.na(Status))))
# rerun VCV MATRIX
V.d2.shared.lnRR_SO2_status <- VCV.shared(data=metadat_SO2_status,
                                          cV="cV.lnRR", #covariance
                                          V = "lnrr.vi", #variance
                                          cluster="sharedcontrolcluster", #clustering factor
                                          obs="infoid") # each data row has a unique infoid
#is.positive.definite(V.d2.shared.lnRR_SO2_status) # check the VCV is made correctly

Q2.SO2.null <- rma.mv(yi = lnrr.yi,
                      V = V.d2.shared.lnRR_SO2_status,
                      random = list(~1 |studyid, ~1 | infoid, ~1 | multipleoutcomecluster),
                      data = metadat_SO2_status, method = "ML")
Q2.SO2 <- rma.mv(yi = lnrr.yi,
                 V = V.d2.shared.lnRR_SO2_status,
                 mods = ~ Status -1,
                 random = list(~1 |studyid, ~1 | infoid, ~1 | multipleoutcomecluster),
                 data = metadat_SO2_status, method = "ML")

# reml
Q2.SO2.reml <- rma.mv(yi = lnrr.yi,
                 V = V.d2.shared.lnRR_SO2_status,
                 mods = ~ Status -1,
                 random = list(~1 |studyid, ~1 | infoid, ~1 | multipleoutcomecluster),
                 data = metadat_SO2_status, method = "REML")

anova(Q2.SO2.null,Q2.SO2)
```

```
##
##         df      AIC      BIC     AICc    logLik    LRT   pval        QE
## Full     6 296.8970 317.9046 297.2500 -142.4485              2559.5803
## Reduced  4 299.3097 313.3147 299.4764 -145.6549 6.4127 0.0405 3118.9168
```

```r
summary(Q2.SO2.reml)
```

```
##
## Multivariate Meta-Analysis Model (k = 245; method: REML)
##
##    logLik   Deviance        AIC        BIC       AICc
## -140.2602   280.5204   292.5204   313.4540   292.8778
##
## Variance Components:
##
##             estim    sqrt  nlvls  fixed                  factor
```

```
## sigma^2.1  0.0378  0.1944     32     no                 studyid
## sigma^2.2  0.0184  0.1356    245     no                  infoid
## sigma^2.3  0.1558  0.3947    160     no  multipleoutcomecluster
##
## Test for Residual Heterogeneity:
## QE(df = 242) = 2559.5803, p-val < .0001
##
## Test of Moderators (coefficients 1:3):
## QM(df = 3) = 6.3994, p-val = 0.0937
##
## Model Results:
##
##                          estimate      se     zval    pval    ci.lb    ci.ub
## StatusBeneficial          -0.2292  0.1148  -1.9977  0.0457  -0.4542  -0.0043  *
## StatusOther herbivore     -0.0305  0.0832  -0.3664  0.7141  -0.1935   0.1325
## StatusSignificant pest     0.1407  0.0930   1.5134  0.1302  -0.0415   0.3230
##
## ---
## Signif. codes:  0 '***' 0.001 '**' 0.01 '*' 0.05 '.' 0.1 ' ' 1
```

```r
r2_ml(Q2.SO2.reml)
```

```
##    R2_marginal R2_conditional
##     0.07910104     0.92014471
```

Status - PM

```r
metadat_PM_status <- droplevels(subset(metadat_PM,!(is.na(Status))))
# rerun VCV MATRIX
V.d2.shared.lnRR_PM_status <- VCV.shared(data=metadat_PM_status,
                                          cV="cV.lnRR", #covariance
                                          V = "lnrr.vi", #variance
                                          cluster="sharedcontrolcluster", #clustering factor
                                          obs="infoid") # each data row has a unique infoid
#is.positive.definite(V.d2.shared.lnRR_PM_status) # check the VCV is made correctly

Q2.PM.null <- rma.mv(yi = lnrr.yi,
                     V = V.d2.shared.lnRR_PM_status,
                     random = list(~1 |studyid, ~1 | infoid, ~1 | multipleoutcomecluster),
                     data = metadat_PM_status, method = "ML")
Q2.PM <- rma.mv(yi = lnrr.yi,
                V = V.d2.shared.lnRR_PM_status,
                mods = ~ Status -1,
                random = list(~1 |studyid, ~1 | infoid, ~1 | multipleoutcomecluster),
                data = metadat_PM_status, method = "ML")

Q2.PM.reml <- rma.mv(yi = lnrr.yi,
                V = V.d2.shared.lnRR_PM_status,
                mods = ~ Status -1,
                random = list(~1 |studyid, ~1 | infoid, ~1 | multipleoutcomecluster),
                data = metadat_PM_status, method = "REML")
```

```r
anova(Q2.PM.null,Q2.PM)
```

```
##
##         df     AIC     BIC    AICc   logLik    LRT   pval       QE
## Full     6 67.4164 79.7791 69.0635 -27.7082                403.4048
## Reduced  4 68.2091 76.4509 68.9638 -30.1046 4.7927 0.0910 501.0757
```

```r
summary(Q2.PM.reml)
```

```
##
## Multivariate Meta-Analysis Model (k = 58; method: REML)
##
##   logLik  Deviance       AIC       BIC      AICc
## -24.1378   48.2756   60.2756   72.3196   62.0256
##
## Variance Components:
##
##              estim    sqrt  nlvls  fixed                 factor
## sigma^2.1  0.4774  0.6909      8     no                studyid
## sigma^2.2  0.0536  0.2314     57     no                 infoid
## sigma^2.3  0.0000  0.0000     20     no  multipleoutcomecluster
##
## Test for Residual Heterogeneity:
## QE(df = 55) = 403.4048, p-val < .0001
##
## Test of Moderators (coefficients 1:3):
## QM(df = 3) = 7.6068, p-val = 0.0549
##
## Model Results:
##
##                           estimate      se     zval    pval    ci.lb   ci.ub
## StatusBeneficial           -0.3007  0.3710  -0.8106  0.4176  -1.0277  0.4264
## StatusOther herbivore      -0.5015  0.3920  -1.2793  0.2008  -1.2699  0.2668
## StatusSignificant pest      0.4871  0.4288   1.1359  0.2560  -0.3534  1.3276
##
## ---
## Signif. codes:  0 '***' 0.001 '**' 0.01 '*' 0.05 '.' 0.1 ' ' 1
```

```r
r2_ml(Q2.PM.reml)
```

```
##    R2_marginal R2_conditional
##      0.1619523      0.9154620
```

Figure 2

See main text for Figure 2

4.2. Feeding guilds: which feeding guilds are most affected by air pollution?

```
metadat_4P.FG <- droplevels(subset(metadat_4P,!(is.na(feeding.guild))))
metadat_4P_FG <- droplevels(subset(metadat_4P.FG,feeding.guild!="multiple")) #817 levels

# make VCV matrix
V.d2.shared.lnRR_FG <- VCV.shared(data=metadat_4P_FG,
                                  cV="cV.lnRR", #covariance
                                  V = "lnrr.vi", #variance
                                  cluster="sharedcontrolcluster", #clustering factor
                                  obs="infoid") # each data row has a unique infoid
#is.positive.definite(V.d2.shared.lnRR_FG) # check the VCV is made correctly

# run overall model
Q3.null.4P <- rma.mv(yi = lnrr.yi,
                       V = V.d2.shared.lnRR_FG,
                       random = list(~1 |studyid, ~1 | infoid, ~1 | multipleoutcomecluster),
                       data = metadat_4P_FG, method = "ML")
Q3.4P <- rma.mv(yi = lnrr.yi,
                   V = V.d2.shared.lnRR_FG,
                   mods = ~ feeding.guild -1,
                   random = list(~1 |studyid, ~1 | infoid, ~1 | multipleoutcomecluster),
                   data = metadat_4P_FG, method = "ML")
anova(Q3.null.4P,Q3.4P)
```

```
##
##          df       AIC       BIC      AICc    logLik     LRT   pval        QE
## Full     10 1034.1226 1081.2278 1034.3942 -507.0613               7736.5603
## Reduced   4 1061.2371 1080.0792 1061.2861 -526.6185 39.1145 <.0001 8411.9944
```

```
# run as reml
Q3.4P.reml <- rma.mv(yi = lnrr.yi,
                   V = V.d2.shared.lnRR_FG,
                   mods = ~ feeding.guild -1,
                   random = list(~1 |studyid, ~1 | infoid, ~1 | multipleoutcomecluster),
                   data = metadat_4P_FG, method = "REML")
summary(Q3.4P.reml)
```

```
##
## Multivariate Meta-Analysis Model (k = 821; method: REML)
##
##    logLik   Deviance        AIC        BIC       AICc
## -502.4177  1004.8354  1024.8354  1071.8550  1025.1094
##
## Variance Components:
##
##              estim    sqrt  nlvls  fixed                  factor
## sigma^2.1  0.0742  0.2724    116     no                 studyid
## sigma^2.2  0.0381  0.1951    819     no                  infoid
## sigma^2.3  0.0638  0.2526    522     no  multipleoutcomecluster
##
## Test for Residual Heterogeneity:
## QE(df = 814) = 7736.5603, p-val < .0001
##
```

```
## Test of Moderators (coefficients 1:7):
## QM(df = 7) = 47.9460, p-val < .0001
##
## Model Results:
##
##                                      estimate      se     zval    pval    ci.lb
## feeding.guildborer/miner               0.4345  0.1566   2.7743  0.0055   0.1276
## feeding.guildcell-feeder               0.0217  0.0587   0.3694  0.7118  -0.0933
## feeding.guildchewer                   -0.0441  0.0481  -0.9162  0.3595  -0.1383
## feeding.guilddetritivore              -0.3556  0.1628  -2.1848  0.0289  -0.6746
## feeding.guildnectar/pollen-feeder     -0.4896  0.0909  -5.3863  <.0001  -0.6677
## feeding.guildparasitoid               -0.2044  0.1192  -1.7154  0.0863  -0.4380
## feeding.guildpredator                 -0.1725  0.1864  -0.9253  0.3548  -0.5379
##                                        ci.ub
## feeding.guildborer/miner              0.7415   **
## feeding.guildcell-feeder              0.1367
## feeding.guildchewer                   0.0502
## feeding.guilddetritivore             -0.0366    *
## feeding.guildnectar/pollen-feeder    -0.3114  ***
## feeding.guildparasitoid               0.0291    .
## feeding.guildpredator                 0.1929
##
## ---
## Signif. codes:  0 '***' 0.001 '**' 0.01 '*' 0.05 '.' 0.1 ' ' 1
```

```
# NB, if positive we need a different transformation
(1-exp(Q3.4P.reml$b))*100
```

```
##                                          [,1]
## feeding.guildborer/miner           -54.426522
## feeding.guildcell-feeder            -2.191817
## feeding.guildchewer                  4.311040
## feeding.guilddetritivore            29.925226
## feeding.guildnectar/pollen-feeder   38.710257
## feeding.guildparasitoid             18.488700
## feeding.guildpredator               15.845056
```

Feeding guild - O3

```
#summary(metadat_O3$feeding.guild)
metadat_O3_FG <- droplevels(subset(metadat_O3,feeding.guild!="multiple"))
# rerun VCV MATRIX
V.d2.shared.lnRR_O3_FG <- VCV.shared(data=metadat_O3_FG,
                                     cV="cV.lnRR", #covariance
                                     V = "lnrr.vi", #variance
                                     cluster="sharedcontrolcluster", #clustering factor
                                     obs="infoid") # each data row has a unique infoid
#is.positive.definite(V.d2.shared.lnRR_O3_FG) # check the VCV is made correctly

Q3.null.O3 <- rma.mv(yi = lnrr.yi,
                     V = V.d2.shared.lnRR_O3_FG,
                     random = list(~1 |studyid, ~1 | infoid, ~1 | multipleoutcomecluster),
```

```r
                          data = metadat_03_FG, method = "ML")
Q3.03 <- rma.mv(yi = lnrr.yi,
                    V = V.d2.shared.lnRR_03_FG,
                    mods = ~ feeding.guild -1,
                    random = list(~1 |studyid, ~1 | infoid, ~1 | multipleoutcomecluster),
                    data = metadat_03_FG, method = "ML")

# reml
Q3.03.reml <- rma.mv(yi = lnrr.yi,
                    V = V.d2.shared.lnRR_03_FG,
                    mods = ~ feeding.guild -1,
                    random = list(~1 |studyid, ~1 | infoid, ~1 | multipleoutcomecluster),
                    data = metadat_03_FG, method = "REML")

anova(Q3.03,Q3.null.03)
```

```
##
##         df      AIC      BIC     AICc    logLik     LRT   pval        QE
## Full    10 567.3604 608.5193 567.8581 -273.6802               3932.9379
## Reduced  4 586.5375 603.0011 586.6268 -289.2688 31.1771 <.0001 4022.5518
```

```r
summary(Q3.03.reml)
```

```
##
## Multivariate Meta-Analysis Model (k = 453; method: REML)
##
##    logLik   Deviance        AIC        BIC       AICc
## -269.9041   539.8082   559.8082   600.8114   560.3140
##
## Variance Components:
##
##              estim    sqrt  nlvls  fixed                  factor
## sigma^2.1   0.0926  0.3043     73     no                 studyid
## sigma^2.2   0.0492  0.2218    452     no                  infoid
## sigma^2.3   0.0207  0.1438    285     no  multipleoutcomecluster
##
## Test for Residual Heterogeneity:
## QE(df = 446) = 3932.9379, p-val < .0001
##
## Test of Moderators (coefficients 1:7):
## QM(df = 7) = 37.6508, p-val < .0001
##
## Model Results:
##
##                                    estimate      se     zval    pval    ci.lb
## feeding.guildborer/miner             0.4360  0.1860   2.3433  0.0191   0.0713
## feeding.guildcell-feeder            -0.0274  0.0749  -0.3660  0.7144  -0.1742
## feeding.guildchewer                 -0.0512  0.0595  -0.8605  0.3895  -0.1680
## feeding.guilddetritivore            -0.6024  0.2696  -2.2345  0.0254  -1.1308
## feeding.guildnectar/pollen-feeder   -0.5518  0.1334  -4.1357  <.0001  -0.8133
## feeding.guildparasitoid             -0.3282  0.1481  -2.2160  0.0267  -0.6185
## feeding.guildpredator                0.0062  0.2228   0.0279  0.9777  -0.4304
```

```
##                                        ci.ub
## feeding.guildborer/miner             0.8006    *
## feeding.guildcell-feeder             0.1194
## feeding.guildchewer                  0.0655
## feeding.guilddetritivore            -0.0740    *
## feeding.guildnectar/pollen-feeder   -0.2903  ***
## feeding.guildparasitoid             -0.0379    *
## feeding.guildpredator                0.4429
##
## ---
## Signif. codes:  0 '***' 0.001 '**' 0.01 '*' 0.05 '.' 0.1 ' ' 1
```

```
r2_ml(Q3.03.reml)
```

```
##    R2_marginal R2_conditional
##      0.1708107      0.7490129
```

Feeding guild - NOx

```
#summary(metadat_NOx$feeding.guild)
metadat_NOx_FG <- droplevels(subset(metadat_NOx,feeding.guild!="predator"&feeding.guild!="multiple",drop

levels(factor(metadat_NOx$feeding.guild))
```

```
## [1] "cell-feeder"           "chewer"                "multiple"
## [4] "nectar/pollen-feeder" "parasitoid"            "predator"
```

```
levels(factor(metadat_NOx_FG$feeding.guild))
```

```
## [1] "cell-feeder"           "chewer"                "nectar/pollen-feeder"
## [4] "parasitoid"
```

```
levels(factor(metadat_NOx_FG$feeding.guild))
```

```
## [1] "cell-feeder"           "chewer"                "nectar/pollen-feeder"
## [4] "parasitoid"
```

```
levels(metadat_NOx_FG$feeding.guild)
```

```
## [1] "cell-feeder"           "chewer"                "nectar/pollen-feeder"
## [4] "parasitoid"
```

```
# rerun VCV MATRIX
V.d2.shared.lnRR_NOx_FG <- VCV.shared(data=metadat_NOx_FG,
                                     cV="cV.lnRR", #covariance
                                     V = "lnrr.vi", #variance
                                     cluster="sharedcontrolcluster", #clustering factor
                                     obs="infoid") # each data row has a unique infoid
```

```r
#is.positive.definite(V.d2.shared.lnRR_NOx_FG) # check the VCV is made correctly

Q3.null.NOx <- rma.mv(yi = lnrr.yi,
                      V = V.d2.shared.lnRR_NOx_FG,
                      random = list(~1 |studyid, ~1 | infoid, ~1 | multipleoutcomecluster),
                      data = metadat_NOx_FG, method = "ML")
Q3.NOx <- rma.mv(yi = lnrr.yi,
                  V = V.d2.shared.lnRR_NOx_FG,
                  mods = ~ feeding.guild -1,
                  random = list(~1 |studyid, ~1 | infoid, ~1 | multipleoutcomecluster),
                  data = metadat_NOx_FG, method = "ML")

#reml
Q3.NOx.reml <- rma.mv(yi = lnrr.yi,
                  V = V.d2.shared.lnRR_NOx_FG,
                  mods = ~ feeding.guild -1,
                  random = list(~1 |studyid, ~1 | infoid, ~1 | multipleoutcomecluster),
                  data = metadat_NOx_FG, method = "REML")

anova(Q3.NOx,Q3.null.NOx)
```

```
##
##          df      AIC      BIC     AICc   logLik     LRT   pval       QE
## Full      7 101.3419 116.9806 103.1780 -43.6710                588.0402
## Reduced   4 111.7465 120.6829 112.3715 -51.8733 16.4046 0.0009 674.0319
```

```r
summary(Q3.NOx.reml)
```

```
##
## Multivariate Meta-Analysis Model (k = 69; method: REML)
##
##   logLik  Deviance       AIC       BIC      AICc
## -43.0806   86.1611  100.1611  115.3818  102.1261
##
## Variance Components:
##
##             estim    sqrt  nlvls  fixed                  factor
## sigma^2.1  0.0000  0.0000     18     no                 studyid
## sigma^2.2  0.0238  0.1544     69     no                  infoid
## sigma^2.3  0.1046  0.3234     59     no  multipleoutcomecluster
##
## Test for Residual Heterogeneity:
## QE(df = 65) = 588.0402, p-val < .0001
##
## Test of Moderators (coefficients 1:4):
## QM(df = 4) = 18.1300, p-val = 0.0012
##
## Model Results:
##
##                                    estimate      se     zval    pval    ci.lb
## feeding.guildcell-feeder             0.0743  0.0757   0.9819  0.3262  -0.0741
## feeding.guildchewer                  0.0555  0.1374   0.4039  0.6863  -0.2138
```

```
## feeding.guildnectar/pollen-feeder   -0.6307  0.1535  -4.1096  <.0001  -0.9315
## feeding.guildparasitoid             -0.0418  0.1237  -0.3375  0.7357  -0.2843
##                                      ci.ub
## feeding.guildcell-feeder            0.2227
## feeding.guildchewer                 0.3248
## feeding.guildnectar/pollen-feeder  -0.3299  ***
## feeding.guildparasitoid             0.2008
##
## ---
## Signif. codes:  0 '***' 0.001 '**' 0.01 '*' 0.05 '.' 0.1 ' ' 1
```

```r
r2_ml(Q3.NOx.reml)
```

```
##    R2_marginal R2_conditional
##      0.3737247      0.8837949
```

Feeding guild - SO2

```r
#summary(metadat_SO2$feeding.guild)
#only include feeding guilds if they are represented in 3 or more studies:
metadat_SO2_FG <- droplevels(subset(metadat_SO2,feeding.guild!="multiple"&
feeding.guild!="borer/miner"&feeding.guild!="detritivore"))
# rerun VCV MATRIX
V.d2.shared.lnRR_SO2_FG <- VCV.shared(data=metadat_SO2_FG,
                                     cV="cV.lnRR", #covariance
                                     V = "lnrr.vi", #variance
                                     cluster="sharedcontrolcluster", #clustering factor
                                     obs="infoid") # each data row has a unique infoid
#is.positive.definite(V.d2.shared.lnRR_SO2_FG) # check the VCV is made correctly

Q3.null.SO2 <- rma.mv(yi = lnrr.yi,
                      V = V.d2.shared.lnRR_SO2_FG,
                      random = list(~1 |studyid, ~1 | infoid, ~1 | multipleoutcomecluster),
                      data = metadat_SO2_FG, method = "ML")
Q3.SO2 <- rma.mv(yi = lnrr.yi,
                 V = V.d2.shared.lnRR_SO2_FG,
                 mods = ~ feeding.guild -1,
                 random = list(~1 |studyid, ~1 | infoid, ~1 | multipleoutcomecluster),
                 data = metadat_SO2_FG, method = "ML")
#reml
Q3.SO2.reml <- rma.mv(yi = lnrr.yi,
                 V = V.d2.shared.lnRR_SO2_FG,
                 mods = ~ feeding.guild -1,
                 random = list(~1 |studyid, ~1 | infoid, ~1 | multipleoutcomecluster),
                 data = metadat_SO2_FG, method = "REML")

anova(Q3.SO2,Q3.null.SO2)
```

```
##
##         df      AIC      BIC     AICc    logLik    LRT   pval        QE
## Full     8 242.9186 270.1401 243.5947 -113.4593              2169.2635
## Reduced  4 241.2441 254.8548 241.4284 -116.6220 6.3254 0.1761 2882.6704
```

```r
summary(Q3.SO2.reml)
```

```
##
## Multivariate Meta-Analysis Model (k = 222; method: REML)
##
##    logLik   Deviance        AIC        BIC       AICc
## -109.7518   219.5037   235.5037   262.5429   236.1960
##
## Variance Components:
##
##             estim    sqrt  nlvls  fixed                 factor
## sigma^2.1  0.0425  0.2061     29     no                studyid
## sigma^2.2  0.0100  0.0999    222     no                 infoid
## sigma^2.3  0.1723  0.4151    143     no  multipleoutcomecluster
##
## Test for Residual Heterogeneity:
## QE(df = 217) = 2169.2635, p-val < .0001
##
## Test of Moderators (coefficients 1:5):
## QM(df = 5) = 6.0959, p-val = 0.2970
##
## Model Results:
##
##                                    estimate      se     zval    pval    ci.lb
## feeding.guildcell-feeder             0.1018  0.1003   1.0145  0.3104  -0.0948
## feeding.guildchewer                 -0.0157  0.0936  -0.1672  0.8672  -0.1992
## feeding.guildnectar/pollen-feeder   -0.3915  0.1830  -2.1388  0.0325  -0.7502
## feeding.guildparasitoid             -0.0221  0.1943  -0.1138  0.9094  -0.4030
## feeding.guildpredator               -0.2893  0.4306  -0.6717  0.5018  -1.1333
##                                      ci.ub
## feeding.guildcell-feeder            0.2983
## feeding.guildchewer                 0.1679
## feeding.guildnectar/pollen-feeder  -0.0327  *
## feeding.guildparasitoid             0.3588
## feeding.guildpredator               0.5548
##
## ---
## Signif. codes:  0 '***' 0.001 '**' 0.01 '*' 0.05 '.' 0.1 ' ' 1
```

```r
r2_ml(Q3.SO2.reml)
```

```
##    R2_marginal R2_conditional
##     0.08705884     0.95945324
```

```r
#orchard_plot(Q3.SO2, mod = "feeding.guild",group="studyid", xlab = "Invertebrate
#performance (lnRR)",data=metadat_SO2_FG,angle=0)+ylim(-2.5,3.5)
```

Feeding guild - PM

```r
#summary(metadat_PM$feeding.guild)
metadat_PM_FG <- droplevels(subset(metadat_PM,feeding.guild!="detritivore" & feeding.guild!="multiple"))
# rerun VCV MATRIX
V.d2.shared.lnRR_PM_FG <- VCV.shared(data=metadat_PM_FG,
                                     cV="cV.lnRR", #covariance
                                     V = "lnrr.vi", #variance
                                     cluster="sharedcontrolcluster", #clustering factor
                                     obs="infoid") # each data row has a unique infoid
#is.positive.definite(V.d2.shared.lnRR_PM_FG) # check the VCV is made correctly
### ##
Q3.null.PM <- rma.mv(yi = lnrr.yi,
                     V = V.d2.shared.lnRR_PM_FG,
                     random = list(~1 |studyid, ~1 | infoid, ~1 | multipleoutcomecluster),
                     data = metadat_PM_FG, method = "ML")
Q3.PM <- rma.mv(yi = lnrr.yi,
                V = V.d2.shared.lnRR_PM_FG,
                mods = ~ feeding.guild -1,
                random = list(~1 |studyid, ~1 | infoid, ~1 | multipleoutcomecluster),
                data = metadat_PM_FG, method = "ML")

# reml
Q3.PM.reml <- rma.mv(yi = lnrr.yi,
                V = V.d2.shared.lnRR_PM_FG,
                mods = ~ feeding.guild -1,
                random = list(~1 |studyid, ~1 | infoid, ~1 | multipleoutcomecluster),
                data = metadat_PM_FG, method = "REML")

anova(Q3.PM,Q3.null.PM)
```

```
##
##         df     AIC     BIC    AICc   logLik    LRT   pval       QE
## Full     5 65.9658 75.7220 67.2701 -27.9829                396.6801
## Reduced  4 64.2111 72.0160 65.0621 -28.1055 0.2453 0.6204 398.6756
```

```r
summary(Q3.PM.reml)
```

```
##
## Multivariate Meta-Analysis Model (k = 52; method: REML)
##
##   logLik  Deviance       AIC       BIC      AICc
## -25.4447   50.8894   60.8894   70.4495   62.2530
##
## Variance Components:
##
##             estim    sqrt  nlvls  fixed                  factor
## sigma^2.1  0.3759  0.6131      6     no                 studyid
## sigma^2.2  0.0733  0.2707     51     no                  infoid
## sigma^2.3  0.0000  0.0000     16     no  multipleoutcomecluster
##
## Test for Residual Heterogeneity:
## QE(df = 50) = 396.6801, p-val < .0001
##
```

```
## Test of Moderators (coefficients 1:2):
## QM(df = 2) = 0.4470, p-val = 0.7997
##
## Model Results:
##
##                                    estimate      se     zval    pval    ci.lb
## feeding.guildchewer                 -0.0507  0.3661  -0.1385  0.8899  -0.7683
## feeding.guildnectar/pollen-feeder   -0.2618  0.4002  -0.6541  0.5131  -1.0462
##                                      ci.ub
## feeding.guildchewer                 0.6669
## feeding.guildnectar/pollen-feeder   0.5227
##
## ---
## Signif. codes:  0 '***' 0.001 '**' 0.01 '*' 0.05 '.' 0.1 ' ' 1
```

```
r2_ml(Q3.PM.reml)
```

```
##    R2_marginal R2_conditional
##     0.01164663     0.83878657
```

```
#orchard_plot(Q3.PM, mod = "feeding.guild",group="studyid", xlab = "Invertebrate performance (lnRR)",da
```

Figure 3

See main text for Figure 3

Supplementary Note 5: Air pollutant concentrations

Next, we wanted to assess whether the concentration of elevated air pollution affects the change in invertebrate performance that is measured between control and elevated treatments. Concentration information was missing from many effect sizes, so we first had to subset the dataset. Then we log transform the concentration information, and scale them *within* each pollutant, to make concentrations comparable between pollutants.

```
conc_dat <- droplevels(subset(metadat_4P,!(is.na(eP_conc)))) # 792 rows

conc_dat$zln_eP_conc <- ave(log(conc_dat$eP_conc), list(conc_dat$Pollutant), FUN=scale)

# make VCV matrix for shared control comparisons
all_VCV <- VCV.shared(data=conc_dat,
                                cV="cV.lnRR", #covariance
                                V = "lnrr.vi", #variance
                                cluster="sharedcontrolcluster", #clustering factor
                                obs="infoid") # each data row has a unique infoid
#is.positive.definite(all_VCV) # check the VCV is made correctly

# make null model for this concentration dataset
overall_mod_null.ml <- rma.mv(yi = lnrr.yi,
                              V = all_VCV,
                              random = list(~1|studyid, ~1|infoid, ~1|multipleoutcomecluster),
```

```r
                                 data = conc_dat, method = "ML")

# add concentration to the model - is there an overall effect of elevated air pollutant concentration?
overall_mod.ml <- rma.mv(yi = lnrr.yi,
                                 V = all_VCV,
                                 mod = ~zln_eP_conc,
                                 random = list(~1|studyid, ~1|infoid, ~1|multipleoutcomecluster),
                                 data = conc_dat, method = "ML")
summary(overall_mod.ml)
```

```
##
## Multivariate Meta-Analysis Model (k = 792; method: ML)
##
##    logLik   Deviance        AIC        BIC       AICc
## -508.3883  2392.6054  1026.7765  1050.1493  1026.8528
##
## Variance Components:
##
##              estim    sqrt  nlvls  fixed                  factor
## sigma^2.1  0.0933  0.3055    113     no                 studyid
## sigma^2.2  0.0283  0.1682    790     no                  infoid
## sigma^2.3  0.0727  0.2697    534     no  multipleoutcomecluster
##
## Test for Residual Heterogeneity:
## QE(df = 790) = 7501.2752, p-val < .0001
##
## Test of Moderators (coefficient 2):
## QM(df = 1) = 3.6535, p-val = 0.0560
##
## Model Results:
##
##              estimate      se     zval    pval    ci.lb    ci.ub
## intrcpt       -0.0883  0.0358  -2.4665  0.0136  -0.1585  -0.0181  *
## zln_eP_conc   -0.0527  0.0276  -1.9114  0.0560  -0.1067   0.0013  .
##
## ---
## Signif. codes:  0 '***' 0.001 '**' 0.01 '*' 0.05 '.' 0.1 ' ' 1
```

```r
# do LRT for there being any effect of concentration
anova(overall_mod_null.ml,overall_mod.ml) # p=0.0567 note this is very close to significant
```

```
##
##          df       AIC       BIC      AICc    logLik    LRT   pval        QE
## Full      5 1026.7765 1050.1493 1026.8528 -508.3883               7501.2752
## Reduced   4 1028.4075 1047.1057 1028.4583 -510.2037 3.6310 0.0567 7575.4934
```

```r
# We also test whether there is an effect of control concentration
# To simplify this, we separate control pollutant concentration into 'zero'
#(e.g. filtered air) and 'other' (e.g. ambient concentration of air pollution)
conc_dat$cP_conc_is_zero <- ifelse(conc_dat$cP_conc==0, 0, 1) # if 1, it is not zero
conc_dat$cP_conc_is_zero <- as.factor(conc_dat$cP_conc_is_zero)
```

```r
# cP mod - does the presence of (ambient) pollution in the control change our estimates?
overall_mod.ml_cP <- rma.mv(yi = lnrr.yi,
                            V = all_VCV,
                            mod = ~zln_eP_conc*cP_conc_is_zero,
                            random = list(~1|studyid, ~1|infoid, ~1|multipleoutcomecluster),
                            data = conc_dat, method = "ML")
summary(overall_mod.ml_cP) # no there isn't a change in the slope (no interaction)
```

```
##
## Multivariate Meta-Analysis Model (k = 764; method: ML)
##
##    logLik   Deviance        AIC        BIC       AICc
## -452.5388  2213.8039   919.0775   951.5475   919.2257
##
## Variance Components:
##
##             estim    sqrt  nlvls  fixed                  factor
## sigma^2.1  0.1040  0.3224    111     no                 studyid
## sigma^2.2  0.0288  0.1697    762     no                  infoid
## sigma^2.3  0.0473  0.2176    506     no  multipleoutcomecluster
##
## Test for Residual Heterogeneity:
## QE(df = 760) = 7088.6144, p-val < .0001
##
## Test of Moderators (coefficients 2:4):
## QM(df = 3) = 6.6380, p-val = 0.0844
##
## Model Results:
##
##                               estimate      se     zval    pval    ci.lb
## intrcpt                        -0.0434  0.0602  -0.7205  0.4712  -0.1614
## zln_eP_conc                    -0.0945  0.0385  -2.4554  0.0141  -0.1699
## cP_conc_is_zero1               -0.0619  0.0746  -0.8296  0.4068  -0.2082
## zln_eP_conc:cP_conc_is_zero1    0.0639  0.0550   1.1616  0.2454  -0.0439
##                                 ci.ub
## intrcpt                        0.0746
## zln_eP_conc                   -0.0191  *
## cP_conc_is_zero1               0.0844
## zln_eP_conc:cP_conc_is_zero1   0.1717
##
## ---
## Signif. codes:  0 '***' 0.001 '**' 0.01 '*' 0.05 '.' 0.1 ' ' 1
```

```r
# if we split between studies with zero control and studies with ambient control

# what about cP mod by itself, do we get different responses
# to air pollution if we look at studies with zero control or ambient control?
# the effect sizes are very similar and are not significantly
# different between these two levels
cP_zero_mod.ml <- rma.mv(yi = lnrr.yi,
                         V = all_VCV,
                         mod = ~cP_conc_is_zero,
                         random = list(~1|studyid, ~1|infoid, ~1|multipleoutcomecluster),
```

```r
                                data = conc_dat, method = "ML")
summary(cP_zero_mod.ml)
```

```
##
## Multivariate Meta-Analysis Model (k = 764; method: ML)
##
##    logLik   Deviance        AIC        BIC       AICc
## -455.8265  2220.3794   921.6530   944.8458   921.7322
##
## Variance Components:
##
##             estim    sqrt  nlvls  fixed                  factor
## sigma^2.1  0.1023  0.3199    111     no                 studyid
## sigma^2.2  0.0286  0.1693    762     no                  infoid
## sigma^2.3  0.0491  0.2215    506     no  multipleoutcomecluster
##
## Test for Residual Heterogeneity:
## QE(df = 762) = 7112.9841, p-val < .0001
##
## Test of Moderators (coefficient 2):
## QM(df = 1) = 0.0188, p-val = 0.8908
##
## Model Results:
##
##                   estimate      se     zval    pval    ci.lb   ci.ub
## intrcpt            -0.0884  0.0574  -1.5411  0.1233  -0.2008  0.0240
## cP_conc_is_zero1   -0.0098  0.0715  -0.1373  0.8908  -0.1500  0.1304
##
## ---
## Signif. codes:  0 '***' 0.001 '**' 0.01 '*' 0.05 '.' 0.1 ' ' 1
```

Next we wanted to test whether this varied between significant pests, other herbivores and beneficial insects; 14 data rows in the concentration dataset did not contain information about invertebrate pest status.

```r
conc_dat_status <- droplevels(subset(conc_dat, !(is.na(Status))))  # 782 rows
# rerun VCV matrix
V.conc_dat_status.shared <- VCV.shared(data = conc_dat_status,
    cV = "cV.lnRR", V = "lnrr.vi", cluster = "sharedcontrolcluster",
    obs = "infoid")
# is.positive.definite(V.conc_dat_status.shared) # check
# the VCV is made correctly

# null model for this dataset
conc_dat_status_null <- rma.mv(yi = lnrr.yi, V = V.conc_dat_status.shared,
    random = list(~1 | studyid, ~1 | infoid, ~1 | multipleoutcomecluster),
    data = conc_dat_status, method = "ML")

# add concentration
conc_dat_status_conc <- rma.mv(yi = lnrr.yi, V = V.conc_dat_status.shared,
    mod = ~zln_eP_conc, random = list(~1 | studyid, ~1 | infoid,
        ~1 | multipleoutcomecluster), data = conc_dat_status,
    method = "ML")
anova(conc_dat_status_null, conc_dat_status_conc)  # NS - same as model above
```

```
##
##         df       AIC       BIC      AICc    logLik    LRT   pval        QE
## Full     5 1029.1226 1052.4062 1029.2003 -509.5613              7388.1274
## Reduced  4 1030.1370 1048.7639 1030.1887 -511.0685 3.0144 0.0825 7474.9038
```

```
# add concentration and separate intercepts for status
conc_dat_status_conc_status <- rma.mv(yi = lnrr.yi, V = V.conc_dat_status.shared,
    mod = ~zln_eP_conc + Status, random = list(~1 | studyid,
        ~1 | infoid, ~1 | multipleoutcomecluster), data = conc_dat_status,
    method = "ML")
summary(conc_dat_status_conc_status)
```

```
##
## Multivariate Meta-Analysis Model (k = 778; method: ML)
##
##    logLik   Deviance        AIC        BIC       AICc
## -498.4120  2320.7651  1010.8241  1043.4212  1010.9696
##
## Variance Components:
##
##              estim    sqrt  nlvls  fixed                  factor
## sigma^2.1   0.0727  0.2696    113     no                 studyid
## sigma^2.2   0.0288  0.1698    776     no                  infoid
## sigma^2.3   0.0750  0.2739    522     no  multipleoutcomecluster
##
## Test for Residual Heterogeneity:
## QE(df = 774) = 7124.6450, p-val < .0001
##
## Test of Moderators (coefficients 2:4):
## QM(df = 3) = 26.2459, p-val < .0001
##
## Model Results:
##
##                          estimate      se     zval    pval    ci.lb    ci.ub
## intrcpt                   -0.3696  0.0687  -5.3806  <.0001  -0.5042  -0.2350
## zln_eP_conc               -0.0432  0.0271  -1.5933  0.1111  -0.0964   0.0099
## StatusOther herbivore      0.3019  0.0883   3.4179  0.0006   0.1288   0.4750
## StatusSignificant pest     0.3819  0.0793   4.8142  <.0001   0.2264   0.5374
##
## intrcpt                  ***
## zln_eP_conc
## StatusOther herbivore    ***
## StatusSignificant pest   ***
##
## ---
## Signif. codes:  0 '***' 0.001 '**' 0.01 '*' 0.05 '.' 0.1 ' ' 1
```

```
anova(conc_dat_status_conc, conc_dat_status_conc_status)  # p<0.001 for separate intercepts for status
```

```
##
##         df       AIC       BIC      AICc    logLik     LRT   pval        QE
## Full     7 1010.8241 1043.4212 1010.9696 -498.4120               7124.6450
## Reduced  5 1029.1226 1052.4062 1029.2003 -509.5613 22.2985 <.0001 7388.1274
```

```
# add concentration to the model including with an
# interaction term between pest status and concentration of
# elevated air pollution
conc_dat_status_int <- rma.mv(yi = lnrr.yi, V = V.conc_dat_status.shared,
    mod = ~zln_eP_conc * Status, random = list(~1 | studyid,
        ~1 | infoid, ~1 | multipleoutcomecluster), data = conc_dat_status,
    method = "ML")
anova(conc_dat_status_conc_status, conc_dat_status_int)
```

```
##
##          df       AIC       BIC      AICc    logLik    LRT   pval        QE
## Full      9 1014.5735 1056.4841 1014.8079 -498.2868               7075.7552
## Reduced   7 1010.8241 1043.4212 1010.9696 -498.4120 0.2506 0.8822 7124.6450
```

```
# no difference in slopes for concentration between pest
# status'; p value is 0.8822
```

Figure 4

See main text for Figure 4

Supplementary Note 6: Publication bias and sensitivity analysis

We check for publication bias by making a funnel plot from a model including the two most significant moderators (pest status and feeding guild) and using Egger's regression ([3]Egger et al. 1997; https://doi.org/10.1136/bmj.315.7109.629). A significant slope for standard error (sqrt(vi)) would indicate statistically significant funnel asymmetry after controlling for all other variables in the model.

Supplementary Fig. 3

```
png(file = "fig/figS5_funnel_R1.png", width = 150, height = 200,
    res = 600, units = "mm")
par(mfrow = c(2, 1))
# pest status
f1 <- funnel(Q1B.Status, yaxis = "seinv", level = c(90, 95, 99),
    ylim = c(0.2, 2.4), shade = c("white", "gray55", "gray75"),
    refline = 0, legend = TRUE)
mtext("A", side = 3, line = 0, adj = -0.13, cex = 2)
# feeding guild
f2 <- funnel(Q1B.Guild, yaxis = "seinv", level = c(90, 95, 99),
    ylim = c(0.2, 2.4), shade = c("white", "gray55", "gray75"),
    refline = 0, legend = TRUE)
mtext("B", side = 3, line = 0, adj = -0.13, cex = 2)
dev.off()
```

```
## pdf
##   2
```

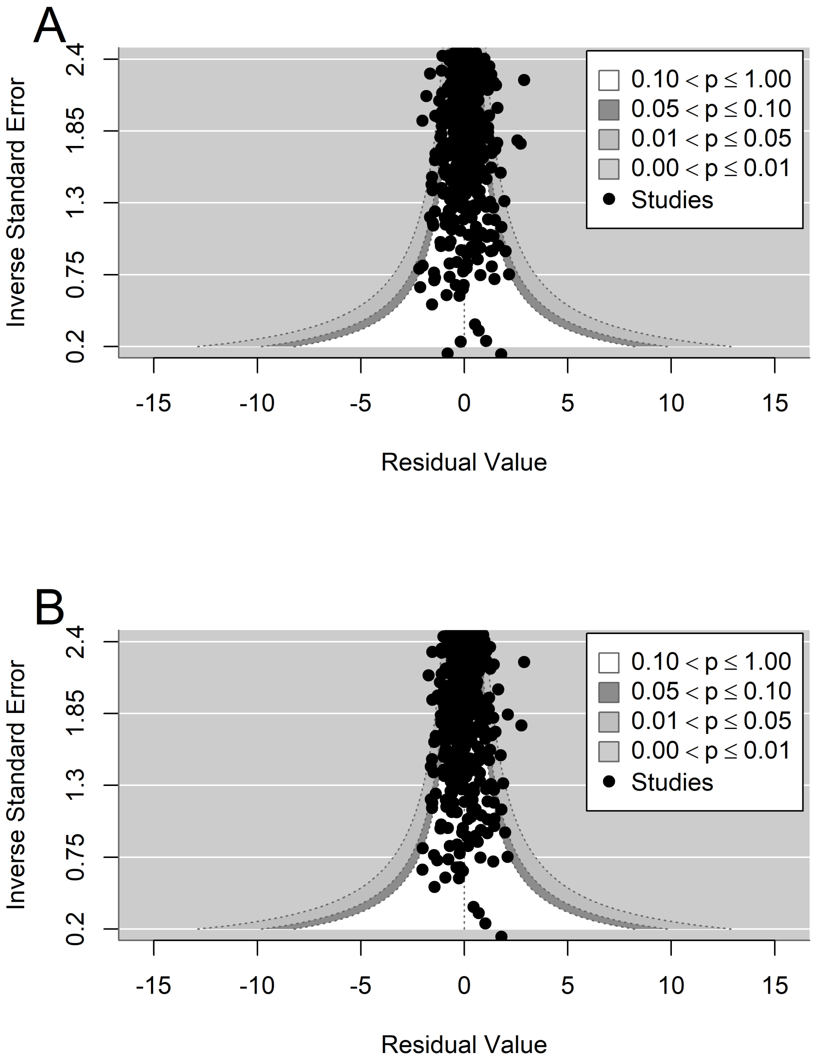

Supplementary Fig. 3. Funnel plots for pest status (A) and feeding guild models (B) showing the relationship between overall effect size and inverse standard error. Symmetry around the mean effect size and no significant correlation between effect size and variance indicated a lack of publication bias.

Eggers regression:

```
egger <- rma.mv(yi = lnrr.yi, V = V.d2.shared.lnRR_4P, mods = sqrt(lnrr.vi),
    random = list(~1 | studyid, ~1 | infoid, ~1 | multipleoutcomecluster),
    data = metadat_4P, method = "ML")
summary(egger)
```

```
##
## Multivariate Meta-Analysis Model (k = 877; method: ML)
##
##    logLik   Deviance        AIC        BIC       AICc
## -557.4385  2643.9073  1124.8771  1148.7596  1124.9460
##
## Variance Components:
##
##              estim    sqrt  nlvls  fixed                 factor
## sigma^2.1  0.0922  0.3037    120     no                studyid
## sigma^2.2  0.0380  0.1949    875     no                 infoid
## sigma^2.3  0.0594  0.2437    562     no  multipleoutcomecluster
##
## Test for Residual Heterogeneity:
## QE(df = 875) = 8607.3463, p-val < .0001
##
## Test of Moderators (coefficient 2):
## QM(df = 1) = 2.2329, p-val = 0.1351
##
## Model Results:
##
##          estimate      se     zval    pval    ci.lb   ci.ub
## intrcpt   -0.0622  0.0406  -1.5305  0.1259  -0.1418  0.0174
## mods      -0.1566  0.1048  -1.4943  0.1351  -0.3620  0.0488
##
## ---
## Signif. codes:  0 '***' 0.001 '**' 0.01 '*' 0.05 '.' 0.1 ' ' 1
```

```
regtest(metadat_4P$lnrr.yi, metadat_4P$lnrr.vi, predictor = "vi")
```

```
##
## Regression Test for Funnel Plot Asymmetry
##
## Model:     mixed-effects meta-regression model
## Predictor: sampling variance
##
## Test for Funnel Plot Asymmetry: z = -0.7637, p = 0.4451
## Limit Estimate (as vi -> 0):    b = -0.0662 (CI: -0.0969, -0.0355)
```

```
# res <- rma(lnrr.yi, lnrr.vi, data=metadat_4P) res reg <-
# regtest(res) reg reg <- regtest(res, predictor='vi') reg
```

We run models (including all pollutants initially) to test for time-lag bias (Koricheva and Kulinskaya 2019). There is a significant effect of Year on invertebrate performance. We don't know why this bias occurs but this trend could be i) a real change in the negative effects of pollutants over time, which could be associated

with interactive effects of other pollutants, or ii) a publication bias effect where there is some bias for the number of negative impacts to be published increasing over time (this could be associated with a larger focus on pollinators in more recent years). We include a two-way interactive effect of air pollution and Year to see if invertebrate performance varies over time between different pollutants. We are able to test the interaction here because year is a continuous variable so we don't encounter the problem of missing factor combinations.

Supplementary Fig. 4

```
test.year.null = rma.mv(yi = lnrr.yi, V = V.d2.shared.lnRR_4P,
    random = list(~1 | studyid, ~1 | infoid, ~1 | multipleoutcomecluster),
    data = metadat_4P, method = "ML")

test.year = rma.mv(yi = lnrr.yi, V = V.d2.shared.lnRR_4P, mods = ~Year,
    random = list(~1 | studyid, ~1 | infoid, ~1 | multipleoutcomecluster),
    data = metadat_4P, method = "ML")
test.year
```

```
##
## Multivariate Meta-Analysis Model (k = 877; method: ML)
##
## Variance Components:
##
##              estim    sqrt  nlvls  fixed                  factor
## sigma^2.1  0.0725  0.2692    120     no                 studyid
## sigma^2.2  0.0371  0.1926    875     no                  infoid
## sigma^2.3  0.0617  0.2483    562     no  multipleoutcomecluster
##
## Test for Residual Heterogeneity:
## QE(df = 875) = 8519.6074, p-val < .0001
##
## Test of Moderators (coefficient 2):
## QM(df = 1) = 17.7553, p-val < .0001
##
## Model Results:
##
##          estimate      se     zval    pval    ci.lb    ci.ub
## intrcpt   21.3750  5.0950   4.1953  <.0001  11.3889  31.3610  ***
## Year      -0.0107  0.0025  -4.2137  <.0001  -0.0157  -0.0057  ***
##
## ---
## Signif. codes:  0 '***' 0.001 '**' 0.01 '*' 0.05 '.' 0.1 ' ' 1
```

```
# Time-lag bias plot (all pollutants):
summary(metadat_4P$Year)
```

```
##    Min. 1st Qu.  Median    Mean 3rd Qu.    Max.
##    1978    1994    2000    2002    2016    2022
```

```
pred_time <- predict.rma(test.year)
```

```
time_lag <- metadat_4P %>%
    mutate(ymin = pred_time$ci.lb, ymax = pred_time$ci.ub, ymin2 = pred_time$cr.lb,
        ymax2 = pred_time$cr.ub, pred = pred_time$pred) %>%
    ggplot(aes(x = Year, y = lnrr.yi, size = sqrt(1/lnrr.vi))) +
    geom_point(shape = 21, fill = "grey90") + geom_smooth(aes(y = ymin2),
    method = "loess", se = FALSE, lty = "dotted", lwd = 0.75,
    colour = "#0072B2") + geom_smooth(aes(y = ymax2), method = "loess",
    se = FALSE, lty = "dotted", lwd = 0.75, colour = "#0072B2") +
    geom_smooth(aes(y = ymin), method = "loess", se = FALSE,
        lty = "dotted", lwd = 0.75, colour = "#D55E00") + geom_smooth(aes(y = ymax),
    method = "loess", se = FALSE, lty = "dotted", lwd = 0.75,
    colour = "#D55E00") + geom_smooth(aes(y = pred), method = "loess",
    se = FALSE, lty = "dashed", lwd = 1, colour = "black") +
    ylim(-3, 3) + xlim(1978, 2022) + labs(x = "year", y = "lnRR (effect size)",
    size = "Precision (1/SE)") + guides(fill = "none", colour = "none") +
    # themes
theme_bw() + theme(legend.position = c(0, 1), legend.justification = c(0,
    1)) + theme(legend.direction = "horizontal") + theme(legend.background = element_blank()) +
    theme(axis.text.y = element_text(size = 10, colour = "black",
        hjust = 0.5, angle = 90))
time_lag
```

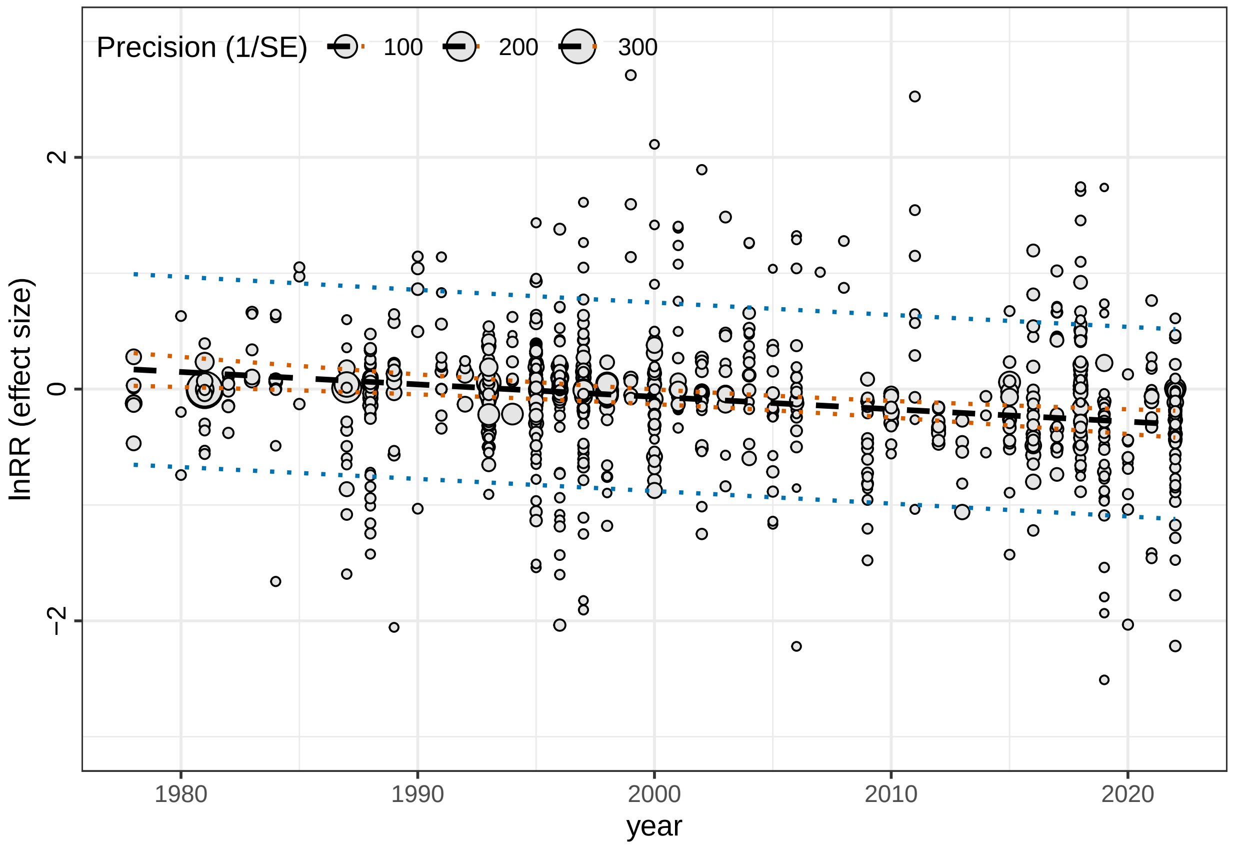

Supplementary Fig. 4. Correlations between year of publication and lnRR for all pollutants. Year of publication was a significant moderator of the overall effect size (z = -4.21, P < 0.001), indicating an overall time-lag bias, which was driven primarily by studies with O3 and NOx.

Time-lag bias for individual pollutants:

```
# Time-lag bias (O3):
test.year.O3.null = rma.mv(yi = lnrr.yi, V = V.d2.shared.lnRR_O3,
    random = list(~1 | studyid, ~1 | infoid, ~1 | multipleoutcomecluster),
    data = metadat_O3, method = "ML")
test.year.O3 = rma.mv(yi = lnrr.yi, V = V.d2.shared.lnRR_O3,
    mods = ~Year, random = list(~1 | studyid, ~1 | infoid, ~1 |
        multipleoutcomecluster), data = metadat_O3, method = "ML")
anova(test.year.O3.null, test.year.O3)
```

```
##
##         df      AIC      BIC     AICc    logLik     LRT   pval        QE
## Full     5 608.2412 629.0893 608.3683 -299.1206                4067.1419
## Reduced  4 619.3467 636.0252 619.4313 -305.6734 13.1055 0.0003 4123.7388
```

```
# plot(metadat_O3$Year,metadat_O3$lnrr.yi)

# Time-lag bias (NOx):
test.year.NOx.null = rma.mv(yi = lnrr.yi, V = V.d2.shared.lnRR_NOx,
    random = list(~1 | studyid, ~1 | infoid, ~1 | multipleoutcomecluster),
    data = metadat_NOx, method = "ML")
test.year.NOx = rma.mv(yi = lnrr.yi, V = V.d2.shared.lnRR_NOx,
    mods = ~Year, random = list(~1 | studyid, ~1 | infoid, ~1 |
        multipleoutcomecluster), data = metadat_NOx, method = "ML")
anova(test.year.NOx.null, test.year.NOx)
```

```
##
##         df      AIC      BIC     AICc   logLik    LRT   pval       QE
## Full     5 130.6605 143.4822 131.3272 -60.3302              748.4781
## Reduced  4 135.2745 145.5319 135.7141 -63.6373 6.6140 0.0101 861.6905
```

```
# Time-lag bias (SO2):
test.year.SO2.null = rma.mv(yi = lnrr.yi, V = V.d2.shared.lnRR_SO2,
    random = list(~1 | studyid, ~1 | infoid, ~1 | multipleoutcomecluster),
    data = metadat_SO2, method = "ML")
test.year.SO2 = rma.mv(yi = lnrr.yi, V = V.d2.shared.lnRR_SO2,
    mods = ~Year, random = list(~1 | studyid, ~1 | infoid, ~1 |
        multipleoutcomecluster), data = metadat_SO2, method = "ML")
anova(test.year.SO2.null, test.year.SO2)
```

```
##
##         df      AIC      BIC     AICc    logLik    LRT   pval        QE
## Full     5 301.1696 318.6759 301.4206 -145.5848               3114.9033
## Reduced  4 299.3097 313.3147 299.4764 -145.6549 0.1401 0.7082 3118.9168
```

```
# Time-lag bias (PM):
test.year.PM.null = rma.mv(yi = lnrr.yi, V = V.d2.shared.lnRR_PM,
    random = list(~1 | studyid, ~1 | infoid, ~1 | multipleoutcomecluster),
    data = metadat_PM, method = "ML")
test.year.PM = rma.mv(yi = lnrr.yi, V = V.d2.shared.lnRR_PM,
```

```r
    mods = ~Year, random = list(~1 | studyid, ~1 | infoid, ~1 |
        multipleoutcomecluster), data = metadat_PM, method = "ML")
anova(test.year.PM.null, test.year.PM)
```

```
##
##         df     AIC     BIC    AICc   logLik    LRT   pval       QE
## Full     5 70.2083 80.5105 71.3621 -30.1041                494.8375
## Reduced  4 68.2091 76.4509 68.9638 -30.1046 0.0009 0.9767 501.0757
```

```r
# plot(metadat_O3$Year,metadat_O3$eP_conc,ylim=c(0,300))
```

Supplementary Fig. 5

```r
# O3
summary(metadat_O3$Year)
```

```
##    Min. 1st Qu.  Median    Mean 3rd Qu.    Max.
##    1984    1995    2002    2005    2016    2022
```

```r
pred_time_O3 <- predict.rma(test.year.O3)
time_lag_O3 <- metadat_O3 %>%
    mutate(ymin = pred_time_O3$ci.lb, ymax = pred_time_O3$ci.ub,
        ymin2 = pred_time_O3$cr.lb, ymax2 = pred_time_O3$cr.ub,
        pred = pred_time_O3$pred) %>%
    ggplot(aes(x = Year, y = lnrr.yi, size = sqrt(1/lnrr.vi))) +
    geom_point(shape = 21, fill = "grey90") + geom_smooth(aes(y = ymin2),
    method = "loess", se = FALSE, lty = "dotted", lwd = 0.75,
    colour = "#0072B2") + geom_smooth(aes(y = ymax2), method = "loess",
    se = FALSE, lty = "dotted", lwd = 0.75, colour = "#0072B2") +
    geom_smooth(aes(y = ymin), method = "loess", se = FALSE,
        lty = "dotted", lwd = 0.75, colour = "#D55E00") + geom_smooth(aes(y = ymax),
    method = "loess", se = FALSE, lty = "dotted", lwd = 0.75,
    colour = "#D55E00") + geom_smooth(aes(y = pred), method = "loess",
    se = FALSE, lty = "dashed", lwd = 1, colour = "black") +
    ylim(-3, 3) + xlim(1984, 2022) + labs(x = "year", y = "lnRR (effect size)",
    size = "Precision (1/SE)") + guides(fill = "none", colour = "none") +
    # themes
theme_bw() + theme(legend.position = c(0, 1), legend.justification = c(0,
    1)) + theme(legend.direction = "horizontal") + theme(legend.background = element_blank()) +
    theme(axis.text.y = element_text(size = 10, colour = "black",
        hjust = 0.5, angle = 90)) + annotate(geom = "text", x = 1984,
    y = -2.4, hjust = 0, label = "paste(O['3'],\" (z = -3.75, P < 0.001)\")",
    size = 4, parse = T)
# time_lag_O3

# NOx
summary(metadat_NOx$Year)
```

```
##    Min. 1st Qu.  Median    Mean 3rd Qu.    Max.
##    1984    1996    2006    2008    2019    2022
```

```r
pred_time_NOx <- predict.rma(test.year.NOx)
time_lag_NOx <- metadat_NOx %>%
    mutate(ymin = pred_time_NOx$ci.lb, ymax = pred_time_NOx$ci.ub,
        ymin2 = pred_time_NOx$cr.lb, ymax2 = pred_time_NOx$cr.ub,
        pred = pred_time_NOx$pred) %>%
    ggplot(aes(x = Year, y = lnrr.yi, size = sqrt(1/lnrr.vi))) +
    geom_point(shape = 21, fill = "grey90") + geom_smooth(aes(y = ymin2),
    method = "loess", se = FALSE, lty = "dotted", lwd = 0.75,
    colour = "#0072B2") + geom_smooth(aes(y = ymax2), method = "loess",
    se = FALSE, lty = "dotted", lwd = 0.75, colour = "#0072B2") +
    geom_smooth(aes(y = ymin), method = "loess", se = FALSE,
        lty = "dotted", lwd = 0.75, colour = "#D55E00") + geom_smooth(aes(y = ymax),
    method = "loess", se = FALSE, lty = "dotted", lwd = 0.75,
    colour = "#D55E00") + geom_smooth(aes(y = pred), method = "loess",
    se = FALSE, lty = "dashed", lwd = 1, colour = "black") +
    ylim(-3, 3) + xlim(1984, 2022) + labs(x = "year", y = "lnRR (effect size)",
    size = "Precision (1/SE)") + guides(fill = "none", colour = "none") +
    # themes
theme_bw() + theme(legend.position = c(0, 1), legend.justification = c(0,
    1)) + theme(legend.direction = "horizontal") + theme(legend.background = element_blank()) +
    theme(axis.text.y = element_text(size = 10, colour = "black",
        hjust = 0.5, angle = 90)) + annotate(geom = "text", x = 1985,
    y = -2.4, hjust = 0, label = "paste(NO['x'],\" (z = -2.87, P = 0.004)\")",
    size = 4, parse = T)
# time_lag_NOx

# SO2
summary(metadat_SO2$Year)
```

```
##    Min. 1st Qu.  Median    Mean 3rd Qu.    Max.
##    1978    1988    1995    1992    1996    2003
```

```r
pred_time_SO2 <- predict.rma(test.year.SO2)
time_lag_SO2 <- metadat_SO2 %>%
    mutate(ymin = pred_time_SO2$ci.lb, ymax = pred_time_SO2$ci.ub,
        ymin2 = pred_time_SO2$cr.lb, ymax2 = pred_time_SO2$cr.ub,
        pred = pred_time_SO2$pred) %>%
    ggplot(aes(x = Year, y = lnrr.yi, size = sqrt(1/lnrr.vi))) +
    geom_point(shape = 21, fill = "grey90") + geom_smooth(aes(y = ymin2),
    method = "loess", se = FALSE, lty = "dotted", lwd = 0.75,
    colour = "#0072B2") + geom_smooth(aes(y = ymax2), method = "loess",
    se = FALSE, lty = "dotted", lwd = 0.75, colour = "#0072B2") +
    geom_smooth(aes(y = ymin), method = "loess", se = FALSE,
        lty = "dotted", lwd = 0.75, colour = "#D55E00") + geom_smooth(aes(y = ymax),
    method = "loess", se = FALSE, lty = "dotted", lwd = 0.75,
    colour = "#D55E00") + geom_smooth(aes(y = pred), method = "loess",
    se = FALSE, lty = "dashed", lwd = 1, colour = "black") +
    ylim(-3, 3) + xlim(1978, 2003) + labs(x = "year", y = "lnRR (effect size)",
    size = "Precision (1/SE)") + guides(fill = "none", colour = "none") +
    # themes
theme_bw() + theme(legend.position = c(0, 1), legend.justification = c(0,
    1)) + theme(legend.direction = "horizontal") + theme(legend.background = element_blank()) +
    theme(axis.text.y = element_text(size = 10, colour = "black",
```

```r
        hjust = 0.5, angle = 90)) + annotate(geom = "text", x = 1978.5,
    y = -2.4, hjust = 0, label = "paste(SO['2'],\" (z = -0.37, P = 0.708)\")",
    size = 4, parse = T)
# time_lag_SO2

# PM
summary(metadat_PM$Year)
```

```
##    Min. 1st Qu.  Median    Mean 3rd Qu.    Max.
##    2017    2018    2018    2018    2018    2021
```

```r
pred_time_PM <- predict.rma(test.year.PM)
time_lag_PM <- metadat_PM %>%
    mutate(ymin = pred_time_PM$ci.lb, ymax = pred_time_PM$ci.ub,
        ymin2 = pred_time_PM$cr.lb, ymax2 = pred_time_PM$cr.ub,
        pred = pred_time_PM$pred) %>%
    ggplot(aes(x = Year, y = lnrr.yi, size = sqrt(1/lnrr.vi))) +
    geom_point(shape = 21, fill = "grey90") + geom_smooth(aes(y = ymin2),
    method = "loess", se = FALSE, lty = "dotted", lwd = 0.75,
    colour = "#0072B2") + geom_smooth(aes(y = ymax2), method = "loess",
    se = FALSE, lty = "dotted", lwd = 0.75, colour = "#0072B2") +
    geom_smooth(aes(y = ymin), method = "loess", se = FALSE,
        lty = "dotted", lwd = 0.75, colour = "#D55E00") + geom_smooth(aes(y = ymax),
    method = "loess", se = FALSE, lty = "dotted", lwd = 0.75,
    colour = "#D55E00") + geom_smooth(aes(y = pred), method = "loess",
    se = FALSE, lty = "dashed", lwd = 1, colour = "black") +
    ylim(-3, 3) + xlim(2017, 2021) + labs(x = "year", y = "lnRR (effect size)",
    size = "Precision (1/SE)") + guides(fill = "none", colour = "none") +
    # themes
theme_bw() + theme(legend.position = c(0, 1), legend.justification = c(0,
    1)) + theme(legend.direction = "horizontal") + theme(legend.background = element_blank()) +
    theme(axis.text.y = element_text(size = 10, colour = "black",
        hjust = 0.5, angle = 90)) + annotate(geom = "text", x = 2017,
    y = -2.4, hjust = 0, label = "paste(PM,\" (z = 0.03, P = 0.976)\")",
    size = 4, parse = T)
# time_lag_PM

library(ggpubr)
Figs5_R1 <- ggarrange(time_lag_O3, time_lag_NOx, time_lag_SO2,
    time_lag_PM, labels = c("A", "B", "C", "D"), ncol = 2, nrow = 2)
ggsave(filename = "fig/FigureS5_R1.tiff", plot = Figs5_R1, width = 10,
    height = 6, dpi = 300, compression = "lzw")
Figs5_R1
```

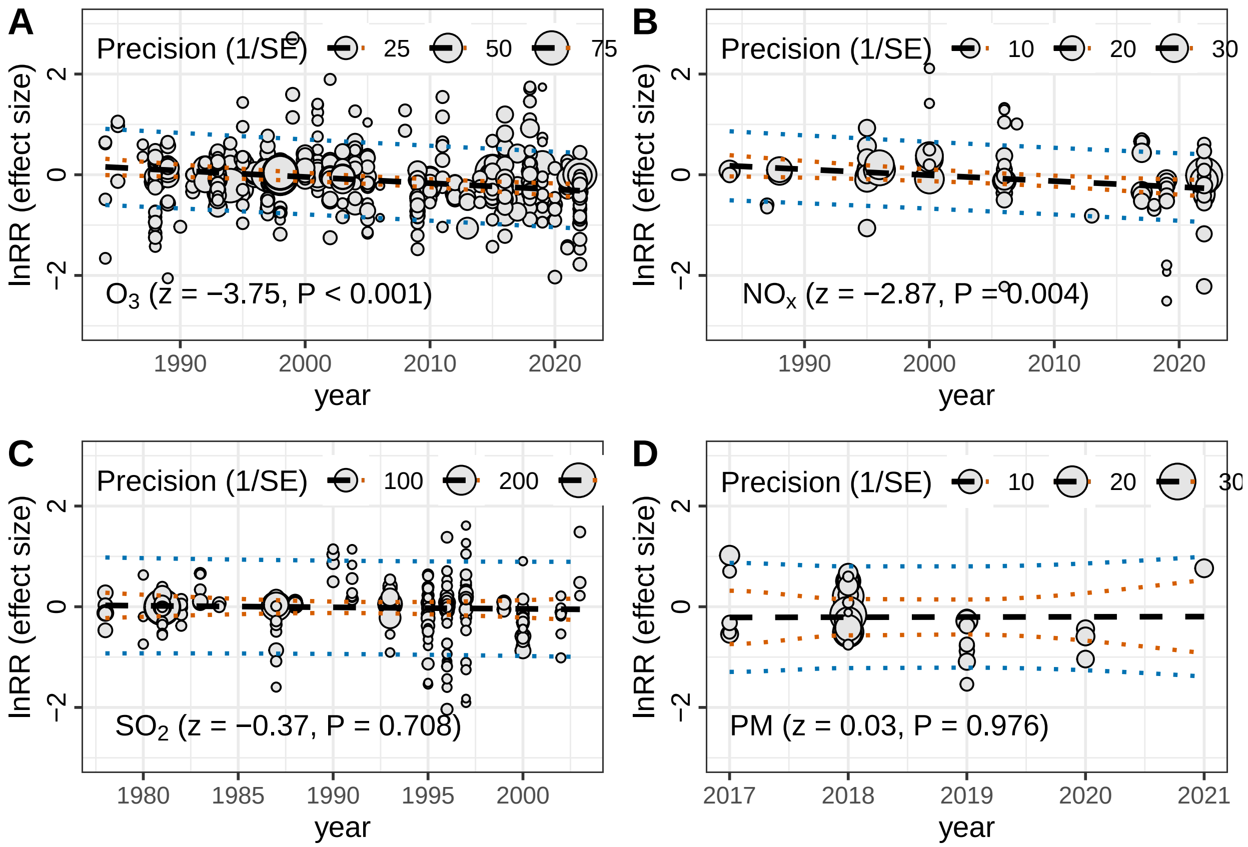

Supplementary Fig. 5. Correlations between year of publication and lnRR for O3 (A), NOx (B), SO2 (C) and PM (D). Year of publication was a significant moderator of the overall effect size, indicating an overall time-lag bias, which was driven primarily by studies with O3 and NOx.

Sensitivity analysis: We use a leave-one-out sensitivity analysis for whole studies to test whether some studies exerted a disproportionate effect on our overall model estimates, e.g. does losing a study change the outcome that beneficial invertebrates are negatively affected by air pollution and invertebrate pests are not?

We run a leave-one-out analysis based upon our main finding that beneficial invertebrates are negatively affected by air pollution and pests/other herbivores are not. Data from a single study in the dataset are removed and the analysis is re-run, this iterates across all studies. We then look at how the model estimates for pest status change.

```
metadat_Status <- readRDS(file = here("R/metadat_Status.rds"))
Q1B.Status <- readRDS(file = here("R/Q1B.Status.rds"))

metadat_Status$studyid <- as.factor(metadat_Status$studyid)
levels(metadat_Status$Status)

LeaveOneOut_effectsize <- list()
for (i in 1:length(levels(metadat_Status$studyid))) {
    # getting VCV ready
    dat <- metadat_Status[metadat_Status$studyid != levels(metadat_Status$studyid)[i],
        ]
    V.d2.shared.lnRR_Status <- VCV.shared(data = dat, cV = "cV.lnRR",
        V = "lnrr.vi", cluster = "sharedcontrolcluster", obs = "infoid")

    # leave one out
```

```
    LeaveOneOut_effectsize[[i]] <- rma.mv(yi = lnrr.yi, V = V.d2.shared.lnRR_Status,
        mods = ~Status - 1, random = list(~1 | studyid, ~1 |
            infoid, ~1 | multipleoutcomecluster), method = "ML",
        data = metadat_Status[metadat_Status$studyid != levels(metadat_Status$studyid)[i],
            ])
}  # not working

# writing function for extracting est, ci.lb, and ci.ub
# from all models
est.func1 <- function(mod_E0) {
    df1 <- data.frame(est = mod_E0$b[[1]], lower = mod_E0$ci.lb[[1]],
        upper = mod_E0$ci.ub[[1]])
    return(df1)
}

est.func2 <- function(mod_E0) {
    df2 <- data.frame(est = mod_E0$b[[2]], lower = mod_E0$ci.lb[[2]],
        upper = mod_E0$ci.ub[[2]])
    return(df2)
}

est.func3 <- function(mod_E0) {
    df3 <- data.frame(est = mod_E0$b[[3]], lower = mod_E0$ci.lb[[3]],
        upper = mod_E0$ci.ub[[3]])
    return(df3)
}

# using dplyr to form data frame
MA_RR_E_bens <- lapply(LeaveOneOut_effectsize, function(x) est.func1(x)) %>%
    bind_rows %>%
    mutate(left_out = levels(metadat_Status$studyid))
MA_RR_E_other <- lapply(LeaveOneOut_effectsize, function(x) est.func2(x)) %>%
    bind_rows %>%
    mutate(left_out = levels(metadat_Status$studyid))
MA_RR_E_pest <- lapply(LeaveOneOut_effectsize, function(x) est.func3(x)) %>%
    bind_rows %>%
    mutate(left_out = levels(metadat_Status$studyid))

saveRDS(MA_RR_E_bens, file = "R/MA_RR_E_bens.rds")
saveRDS(MA_RR_E_other, file = "R/MA_RR_E_other.rds")
saveRDS(MA_RR_E_pest, file = "R/MA_RR_E_pest.rds")
```

Supplementary Fig. 6

```
## pdf
##   2
```

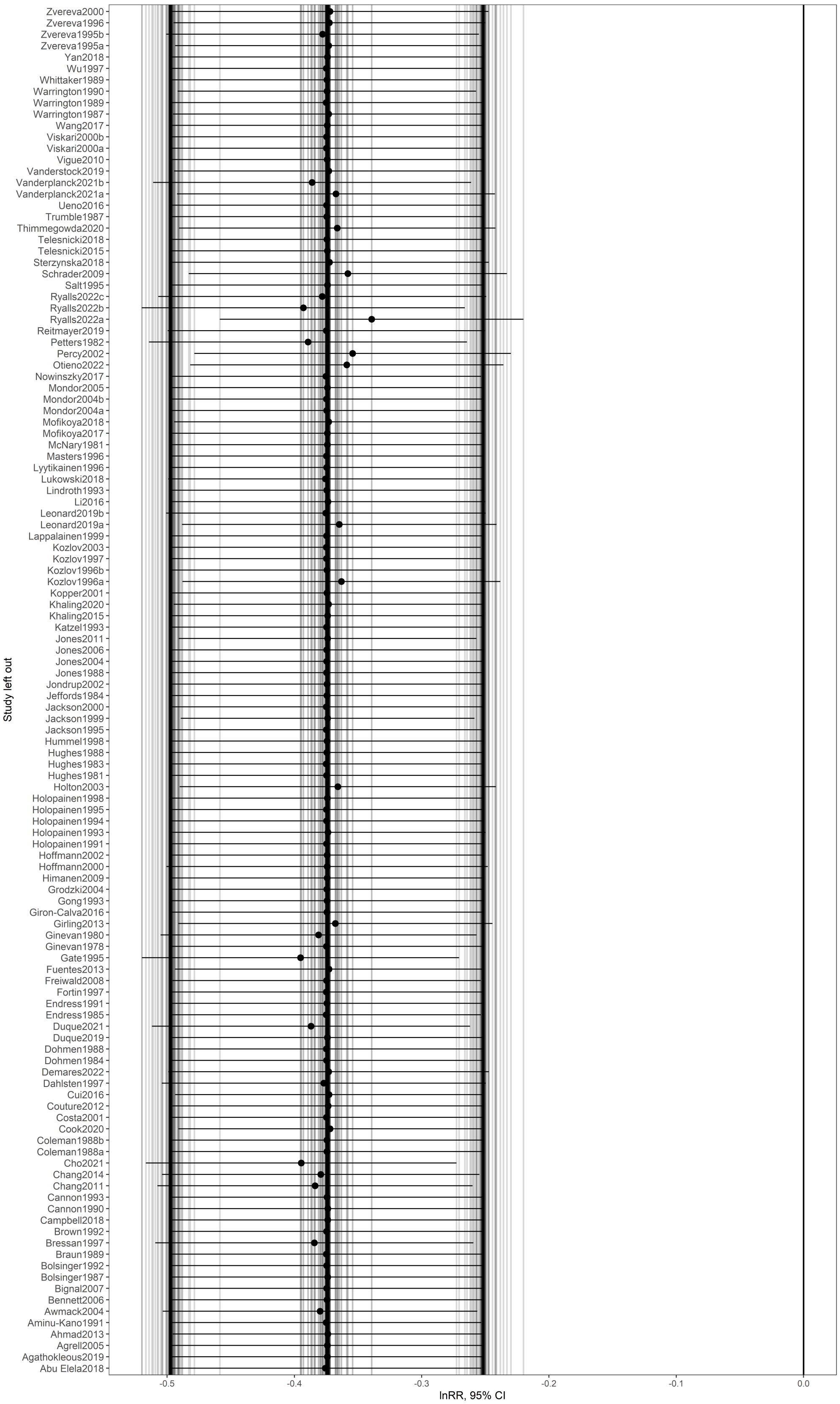

Supplementary Fig. 6. Leave-one-out analysis for beneficial invertebrates

Supplementary Fig. 7

```
## pdf
##   2
```

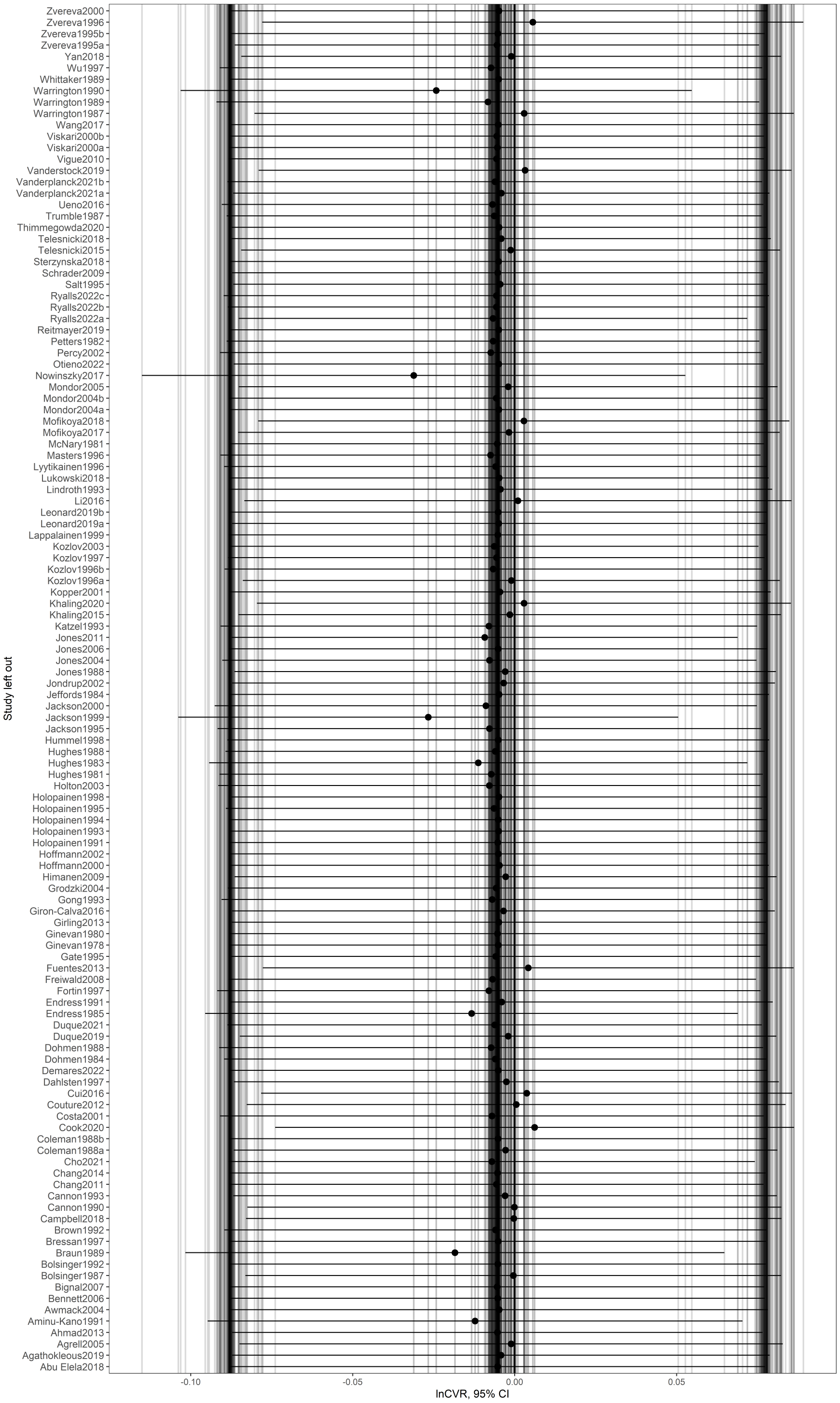

Supplementary Fig. 7. Leave-one-out analysis for significant pest invertebrates

Supplementary Fig. 8

```
## pdf
##   2
```

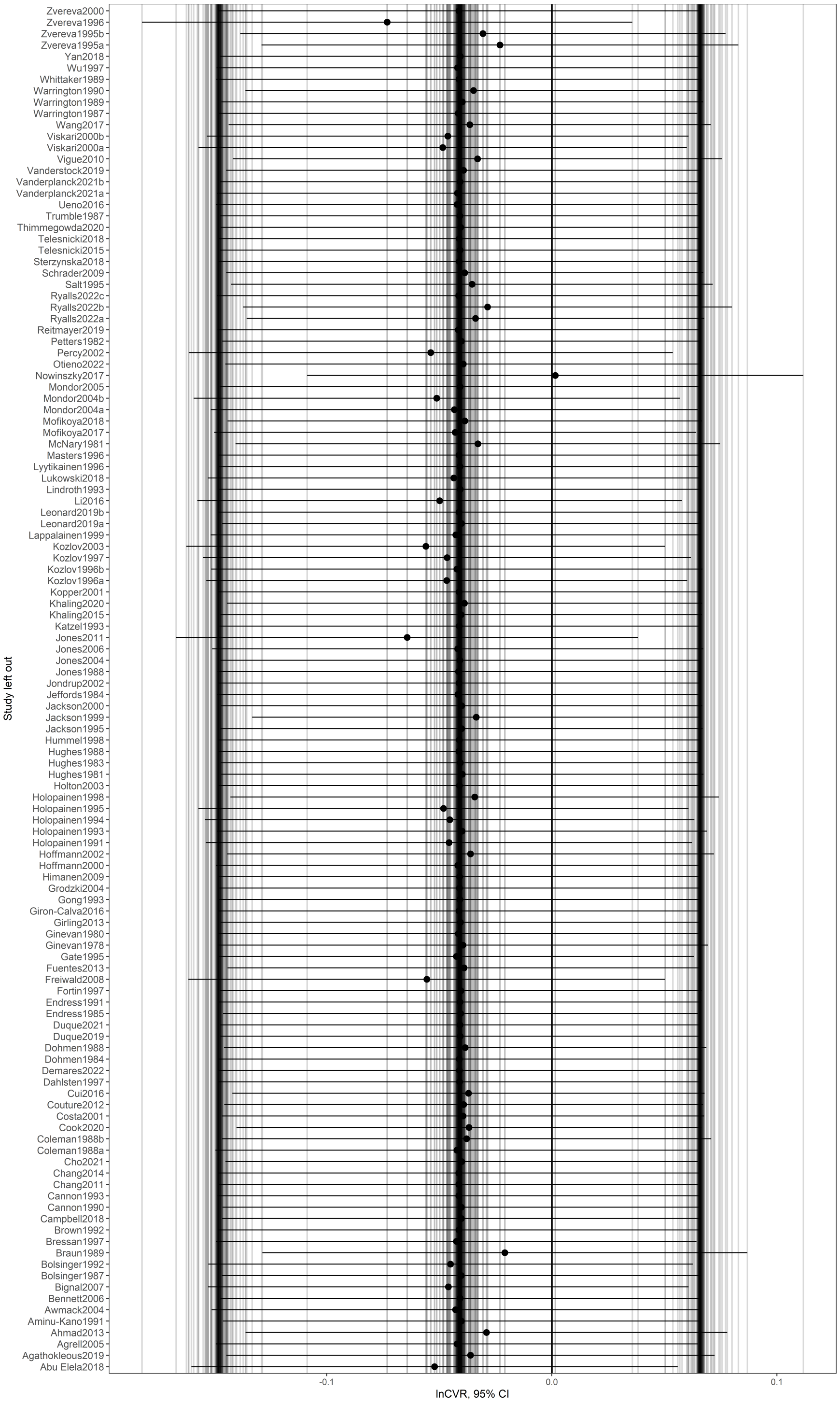

Supplementary Fig. 8. Leave-one-out analysis for other herbivores

We re-run our air pollutant concentration model (manuscript fig 4) excluding the studies where we imputed ambient control concentrations (see methods). This reduced the number of studies by 9 (5 for O3 and 4 for SO2) and marginally increased the significance of our overall model with all pollutants (from p = 0.057 to 0.045).

```
conc_dat_2 <- droplevels(subset(metadat_4P,!(is.na(eP_conc)))) # 792 rows
conc_dat_Imp <- droplevels((subset(conc_dat_2,Control.pollutant.concentration!="ambient"&Control.pollut

conc_dat_Imp$zln_eP_conc <- ave(log(conc_dat_Imp$eP_conc), list(conc_dat_Imp$Pollutant), FUN=scale)

# make VCV matrix for shared control comparisons
all_VCV_Imp <- VCV.shared(data=conc_dat_Imp,
                                 cV="cV.lnRR", #covariance
                                 V = "lnrr.vi", #variance
                                 cluster="sharedcontrolcluster", #clustering factor
                                 obs="infoid") # each data row has a unique infoid
#is.positive.definite(all_VCV) # check the VCV is made correctly

overall_mod_null.ml.Imp <- rma.mv(yi = lnrr.yi,
                               V = all_VCV_Imp,
                               test="t",
                               random = list(~1|studyid, ~1|infoid, ~1|multipleoutcomecluster),
                               data = conc_dat_Imp, method = "ML")

overall_mod.ml.Imp <- rma.mv(yi = lnrr.yi,
                               V = all_VCV_Imp,
                               mod = ~zln_eP_conc,
                          test="t",
                               random = list(~1|studyid, ~1|infoid, ~1|multipleoutcomecluster),
                               data = conc_dat_Imp, method = "ML")

anova(overall_mod_null.ml.Imp,overall_mod.ml.Imp)
```

```
##
##          df      AIC      BIC     AICc    logLik    LRT   pval        QE
## Full      5 965.4027 988.4157 965.4848 -477.7014              7135.3309
## Reduced   4 967.4240 985.8344 967.4787 -479.7120 4.0213 0.0449 7175.9462
```

```
summary(overall_mod.ml.Imp)
```

```
##
## Multivariate Meta-Analysis Model (k = 737; method: ML)
##
##    logLik   Deviance        AIC        BIC       AICc
## -477.7014  2241.4901   965.4027   988.4157   965.4848
##
## Variance Components:
##
##               estim    sqrt  nlvls  fixed                  factor
## sigma^2.1    0.0941  0.3067    104     no                 studyid
## sigma^2.2    0.0315  0.1774    735     no                  infoid
## sigma^2.3    0.0702  0.2650    496     no  multipleoutcomecluster
##
```

```
## Test for Residual Heterogeneity:
## QE(df = 735) = 7135.3309, p-val < .0001
##
## Test of Moderators (coefficient 2):
## F(df1 = 1, df2 = 735) = 4.0412, p-val = 0.0448
##
## Model Results:
##
##              estimate      se     tval   df    pval    ci.lb    ci.ub
## intrcpt       -0.0895  0.0374  -2.3924  735  0.0170  -0.1629  -0.0161  *
## zln_eP_conc   -0.0556  0.0276  -2.0103  735  0.0448  -0.1098  -0.0013  *
##
## ---
## Signif. codes:  0 '***' 0.001 '**' 0.01 '*' 0.05 '.' 0.1 ' ' 1
```

Next we tested whether this varied between significant pests, other herbivores and beneficial insects; excluding these studies did not make any qualitative change to the model findings or confidence intervals for the concentration*pest status interaction.

```
conc_dat_status_Imp <- droplevels(subset(conc_dat_Imp, !(is.na(Status))))  # 723 rows
# rerun VCV matrix
V.conc_dat_status.shared_Imp <- VCV.shared(data = conc_dat_status_Imp,
    cV = "cV.lnRR", V = "lnrr.vi", cluster = "sharedcontrolcluster",
    obs = "infoid")
# is.positive.definite(V.conc_dat_status.shared_Imp) #
# check the VCV is made correctly

# null model for this dataset
conc_dat_status_null_Imp <- rma.mv(yi = lnrr.yi, V = V.conc_dat_status.shared_Imp,
    random = list(~1 | studyid, ~1 | infoid, ~1 | multipleoutcomecluster),
    data = conc_dat_status_Imp, method = "ML")

# add concentration
conc_dat_status_conc_Imp <- rma.mv(yi = lnrr.yi, V = V.conc_dat_status.shared_Imp,
    mod = ~zln_eP_conc, random = list(~1 | studyid, ~1 | infoid,
        ~1 | multipleoutcomecluster), data = conc_dat_status_Imp,
    method = "ML")
anova(conc_dat_status_null_Imp, conc_dat_status_conc_Imp)  # NS
```

```
##
##         df      AIC      BIC     AICc    logLik    LRT   pval        QE
## Full     5 967.5371 990.4541 967.6208 -478.7685               7027.4920
## Reduced  4 968.9077 987.2414 968.9635 -480.4539 3.3707 0.0664 7075.1440
```

```
# add concentration and separate intercepts for status
conc_dat_status_conc_status_Imp <- rma.mv(yi = lnrr.yi, V = V.conc_dat_status.shared_Imp,
    mod = ~zln_eP_conc + Status, random = list(~1 | studyid,
        ~1 | infoid, ~1 | multipleoutcomecluster), data = conc_dat_status_Imp,
    method = "ML")
summary(conc_dat_status_conc_status_Imp)
```

```
##
## Multivariate Meta-Analysis Model (k = 723; method: ML)
```

```
##
##    logLik   Deviance        AIC        BIC       AICc
## -467.6254  2169.4503   949.2508   981.3347   949.4075
##
## Variance Components:
##
##             estim    sqrt  nlvls  fixed                  factor
## sigma^2.1  0.0709  0.2663    104     no                 studyid
## sigma^2.2  0.0321  0.1793    721     no                  infoid
## sigma^2.3  0.0726  0.2695    484     no  multipleoutcomecluster
##
## Test for Residual Heterogeneity:
## QE(df = 719) = 6756.0367, p-val < .0001
##
## Test of Moderators (coefficients 2:4):
## QM(df = 3) = 26.8346, p-val < .0001
##
## Model Results:
##
##                          estimate      se     zval    pval    ci.lb    ci.ub
## intrcpt                   -0.3758  0.0700  -5.3680  <.0001  -0.5129  -0.2386
## zln_eP_conc               -0.0400  0.0273  -1.4631  0.1434  -0.0935   0.0136
## StatusOther herbivore      0.3140  0.0908   3.4578  0.0005   0.1360   0.4920
## StatusSignificant pest     0.3913  0.0811   4.8281  <.0001   0.2325   0.5502
##
## intrcpt                  ***
## zln_eP_conc
## StatusOther herbivore    ***
## StatusSignificant pest   ***
##
## ---
## Signif. codes:  0 '***' 0.001 '**' 0.01 '*' 0.05 '.' 0.1 ' ' 1
```

```
anova(conc_dat_status_conc_Imp, conc_dat_status_conc_status_Imp)  # p<0.001 for separate intercepts for
```

```
##
##         df       AIC       BIC      AICc    logLik     LRT   pval        QE
## Full     7 949.2508 981.3347 949.4075 -467.6254                 6756.0367
## Reduced  5 967.5371 990.4541 967.6208 -478.7685 22.2863 <.0001 7027.4920
```

```
# add concentration to the model including with an
# interaction term between pest status and concentration of
# elevated air pollution
conc_dat_status_int_Imp <- rma.mv(yi = lnrr.yi, V = V.conc_dat_status.shared_Imp,
    mod = ~zln_eP_conc * Status, random = list(~1 | studyid,
        ~1 | infoid, ~1 | multipleoutcomecluster), data = conc_dat_status_Imp,
    method = "ML")
anova(conc_dat_status_conc_status_Imp, conc_dat_status_int_Imp)
```

```
##
##         df       AIC       BIC      AICc    logLik    LRT   pval        QE
## Full     9 952.0084 993.2591 952.2609 -467.0042                6688.1378
## Reduced  7 949.2508 981.3347 949.4075 -467.6254 1.2424 0.5373 6756.0367
```

```r
# no difference in slopes for concentration between pest
# status'; p value is 0.537
```

We re-run our three most important models which link to fig 2-4 in the manuscript, using t-distribution rather than z-distribution - this uses total effect sizes-1 as the degrees of freedom and has been shown to reduce Type I error rates ([4]Nakagawa et al. 2022 https://dx.doi.org/10.1002/ecy.3490). In our case, this approach did not make any qualitative change to the model findings or confidence intervals.

Overall model:

```r
RE3.vcv.t <- rma.mv(yi = lnrr.yi, V = V.d2.shared.lnRR_4P, random = list(~1 |
    studyid, ~1 | infoid, ~1 | multipleoutcomecluster), test = "t",
    data = metadat_4P, method = "ML")
summary(RE3.vcv.t)
```

```
##
## Multivariate Meta-Analysis Model (k = 877; method: ML)
##
##    logLik   Deviance        AIC        BIC       AICc
## -558.5422  2646.1145  1125.0844  1144.1904  1125.1302
##
## Variance Components:
##
##              estim    sqrt  nlvls  fixed                  factor
## sigma^2.1   0.0928  0.3046    120     no                 studyid
## sigma^2.2   0.0373  0.1932    875     no                  infoid
## sigma^2.3   0.0612  0.2473    562     no  multipleoutcomecluster
##
## Test for Heterogeneity:
## Q(df = 876) = 8758.6680, p-val < .0001
##
## Model Results:
##
## estimate      se     tval   df    pval    ci.lb    ci.ub
##  -0.0948  0.0344  -2.7546  876  0.0060  -0.1623  -0.0272  **
##
## ---
## Signif. codes:  0 '***' 0.001 '**' 0.01 '*' 0.05 '.' 0.1 ' ' 1
```

Pest status model:

```r
metadat_Status <- readRDS(file = here("R/metadat_Status.rds"))
Q1B.null.Status.t <- rma.mv(yi = lnrr.yi, V = V.d2.shared.lnRR_Status,
    random = list(~1 | studyid, ~1 | infoid, ~1 | multipleoutcomecluster),
    test = "t", data = metadat_Status, method = "ML")
Q1B.Status.t <- rma.mv(yi = lnrr.yi, V = V.d2.shared.lnRR_Status,
    mods = ~Status - 1, random = list(~1 | studyid, ~1 | infoid,
        ~1 | multipleoutcomecluster), test = "t", data = metadat_Status,
    method = "ML")
anova(Q1B.null.Status.t, Q1B.Status.t)
```

```
##
```

```
##         df       AIC       BIC      AICc    logLik     LRT   pval        QE
## Full     6 1105.0915 1133.6539 1105.1896 -546.5457                8028.0053
## Reduced  4 1126.6916 1145.7333 1126.7382 -559.3458 25.6002 <.0001 8658.7218
```

```
# get R2 for model
r2_ml(Q1B.Status.t)
```

```
##    R2_marginal R2_conditional
##      0.1048394      0.7999669
```

```
# run reml
Q1B.Status.reml.t <- rma.mv(yi = lnrr.yi, V = V.d2.shared.lnRR_Status,
    mods = ~Status - 1, test = "t", random = list(~1 | studyid,
        ~1 | infoid, ~1 | multipleoutcomecluster), data = metadat_Status,
    method = "REML")
summary(Q1B.Status.reml.t)
```

```
##
## Multivariate Meta-Analysis Model (k = 863; method: REML)
##
##    logLik   Deviance        AIC        BIC       AICc
## -544.2349  1088.4699  1100.4699  1129.0115  1100.5683
##
## Variance Components:
##
##             estim    sqrt  nlvls  fixed                  factor
## sigma^2.1  0.0723  0.2689    120     no                 studyid
## sigma^2.2  0.0378  0.1944    861     no                  infoid
## sigma^2.3  0.0631  0.2511    550     no  multipleoutcomecluster
##
## Test for Residual Heterogeneity:
## QE(df = 860) = 8028.0053, p-val < .0001
##
## Test of Moderators (coefficients 1:3):
## F(df1 = 3, df2 = 860) = 11.8117, p-val < .0001
##
## Model Results:
##
##                          estimate      se     tval   df    pval    ci.lb
## StatusBeneficial          -0.3749  0.0634  -5.9146  860  <.0001  -0.4993
## StatusOther herbivore     -0.0431  0.0552  -0.7806  860  0.4352  -0.1514
## StatusSignificant pest    -0.0041  0.0428  -0.0951  860  0.9242  -0.0881
##                            ci.ub
## StatusBeneficial         -0.2505  ***
## StatusOther herbivore     0.0652
## StatusSignificant pest    0.0799
##
## ---
## Signif. codes:  0 '***' 0.001 '**' 0.01 '*' 0.05 '.' 0.1 ' ' 1
```

Feeding guild model:

```r
Q1B.Guild.reml.t <- rma.mv(yi = lnrr.yi, V = V.d2.shared.lnRR_feeding.guild,
    mods = ~feeding.guild - 1, random = list(~1 | studyid, ~1 |
        infoid, ~1 | multipleoutcomecluster), test = "t", data = metadat_feeding.guild,
    method = "REML")
summary(Q1B.Guild.reml.t)
```

```
##
## Multivariate Meta-Analysis Model (k = 821; method: REML)
##
##    logLik   Deviance        AIC        BIC       AICc
## -502.4177  1004.8354  1024.8354  1071.8550  1025.1094
##
## Variance Components:
##
##             estim    sqrt  nlvls  fixed                  factor
## sigma^2.1  0.0742  0.2724    116     no                 studyid
## sigma^2.2  0.0381  0.1951    819     no                  infoid
## sigma^2.3  0.0638  0.2526    522     no  multipleoutcomecluster
##
## Test for Residual Heterogeneity:
## QE(df = 814) = 7736.5603, p-val < .0001
##
## Test of Moderators (coefficients 1:7):
## F(df1 = 7, df2 = 814) = 6.8494, p-val < .0001
##
## Model Results:
##
##                                     estimate      se     tval   df    pval
## feeding.guildborer/miner              0.4345  0.1566   2.7743  814  0.0057
## feeding.guildcell-feeder              0.0217  0.0587   0.3694  814  0.7119
## feeding.guildchewer                  -0.0441  0.0481  -0.9162  814  0.3598
## feeding.guilddetritivore             -0.3556  0.1628  -2.1848  814  0.0292
## feeding.guildnectar/pollen-feeder    -0.4896  0.0909  -5.3863  814  <.0001
## feeding.guildparasitoid              -0.2044  0.1192  -1.7154  814  0.0867
## feeding.guildpredator                -0.1725  0.1864  -0.9253  814  0.3551
##                                      ci.lb    ci.ub
## feeding.guildborer/miner             0.1271   0.7420   **
## feeding.guildcell-feeder            -0.0935   0.1369
## feeding.guildchewer                 -0.1385   0.0503
## feeding.guilddetritivore            -0.6751  -0.0361    *
## feeding.guildnectar/pollen-feeder   -0.6680  -0.3112  ***
## feeding.guildparasitoid             -0.4384   0.0295    .
## feeding.guildpredator               -0.5385   0.1935
##
## ---
## Signif. codes:  0 '***' 0.001 '**' 0.01 '*' 0.05 '.' 0.1 ' ' 1
```

Concentration analysis:

```r
overall_mod_null.ml.t <- rma.mv(yi = lnrr.yi, V = all_VCV, test = "t",
    random = list(~1 | studyid, ~1 | infoid, ~1 | multipleoutcomecluster),
    data = conc_dat, method = "ML")
```

```r
overall_mod.ml.t <- rma.mv(yi = lnrr.yi, V = all_VCV, mod = ~zln_eP_conc,
    test = "t", random = list(~1 | studyid, ~1 | infoid, ~1 |
        multipleoutcomecluster), data = conc_dat, method = "ML")

anova(overall_mod_null.ml.t, overall_mod.ml.t)
```

```
##
##         df       AIC       BIC      AICc    logLik    LRT   pval        QE
## Full     5 1026.7765 1050.1493 1026.8528 -508.3883               7501.2752
## Reduced  4 1028.4075 1047.1057 1028.4583 -510.2037 3.6310 0.0567 7575.4934
```

```r
summary(overall_mod.ml.t)
```

```
##
## Multivariate Meta-Analysis Model (k = 792; method: ML)
##
##    logLik   Deviance        AIC        BIC       AICc
## -508.3883  2392.6054  1026.7765  1050.1493  1026.8528
##
## Variance Components:
##
##             estim    sqrt  nlvls  fixed                 factor
## sigma^2.1  0.0933  0.3055    113     no                studyid
## sigma^2.2  0.0283  0.1682    790     no                 infoid
## sigma^2.3  0.0727  0.2697    534     no  multipleoutcomecluster
##
## Test for Residual Heterogeneity:
## QE(df = 790) = 7501.2752, p-val < .0001
##
## Test of Moderators (coefficient 2):
## F(df1 = 1, df2 = 790) = 3.6535, p-val = 0.0563
##
## Model Results:
##
##              estimate      se     tval   df    pval    ci.lb    ci.ub
## intrcpt       -0.0883  0.0358  -2.4665  790  0.0139  -0.1586  -0.0180  *
## zln_eP_conc   -0.0527  0.0276  -1.9114  790  0.0563  -0.1068   0.0014  .
##
## ---
## Signif. codes:  0 '***' 0.001 '**' 0.01 '*' 0.05 '.' 0.1 ' ' 1
```

Moreover, excluding the performance measure 'Diversity' from the analyses (N = 6 studies), which was included as a population or community performance metric rather than an individual invertebrate metric, did not make any qualitative change to the model findings or confidence intervals.

Pest status model:

```r
# summary(metadat_Status_D$Performance2) with(metadat_4P,
# aggregate(lnrr.yi,by=list(studyid,Performance2),length))
metadat_Status_D <- droplevels(subset(metadat_Status, Performance2 !=
    "diversity"))
# rerun VCV matrix
```

```r
V.d2.shared.lnRR_Status_D <- VCV.shared(data = metadat_Status_D,
    cV = "cV.lnRR", V = "lnrr.vi", cluster = "sharedcontrolcluster",
    obs = "infoid")

Q1B.null.Status_D <- rma.mv(yi = lnrr.yi, V = V.d2.shared.lnRR_Status_D,
    random = list(~1 | studyid, ~1 | infoid, ~1 | multipleoutcomecluster),
    data = metadat_Status_D, method = "ML")
Q1B.Status_D <- rma.mv(yi = lnrr.yi, V = V.d2.shared.lnRR_Status_D,
    mods = ~Status - 1, random = list(~1 | studyid, ~1 | infoid,
        ~1 | multipleoutcomecluster), data = metadat_Status_D,
    method = "ML")
anova(Q1B.null.Status_D, Q1B.Status_D)
```

```
##
##         df       AIC       BIC      AICc    logLik     LRT   pval        QE
## Full     6 1091.9877 1120.3881 1092.0885 -539.9938               7605.6270
## Reduced  4 1112.8342 1131.7678 1112.8821 -552.4171 24.8465 <.0001 7861.6605
```

```r
# get R2 for model


# run reml
Q1B.Status_D.reml <- rma.mv(yi = lnrr.yi, V = V.d2.shared.lnRR_Status_D,
    mods = ~Status - 1, random = list(~1 | studyid, ~1 | infoid,
        ~1 | multipleoutcomecluster), data = metadat_Status_D,
    method = "REML")
summary(Q1B.Status_D.reml)
```

```
##
## Multivariate Meta-Analysis Model (k = 840; method: REML)
##
##    logLik   Deviance        AIC        BIC       AICc
## -537.6813  1075.3627  1087.3627  1115.7416  1087.4639
##
## Variance Components:
##
##             estim    sqrt  nlvls  fixed                  factor
## sigma^2.1  0.0746  0.2731    120     no                 studyid
## sigma^2.2  0.0379  0.1947    838     no                  infoid
## sigma^2.3  0.0640  0.2530    547     no  multipleoutcomecluster
##
## Test for Residual Heterogeneity:
## QE(df = 837) = 7605.6270, p-val < .0001
##
## Test of Moderators (coefficients 1:3):
## QM(df = 3) = 33.4258, p-val < .0001
##
## Model Results:
##
##                           estimate      se     zval    pval    ci.lb    ci.ub
## StatusBeneficial           -0.3735  0.0649  -5.7510  <.0001  -0.5008  -0.2462
## StatusOther herbivore      -0.0371  0.0563  -0.6585  0.5102  -0.1474   0.0733
## StatusSignificant pest     -0.0032  0.0433  -0.0734  0.9415  -0.0880   0.0816
```

```
##
## StatusBeneficial        ***
## StatusOther herbivore
## StatusSignificant pest
##
## ---
## Signif. codes:  0 '***' 0.001 '**' 0.01 '*' 0.05 '.' 0.1 ' ' 1
```

```
r2_ml(Q1B.Status_D.reml)
```

```
##    R2_marginal R2_conditional
##      0.1000305      0.8066583
```

Feeding guild model:

```
# summary(metadat_feeding.guild$Performance2)
metadat_feeding.guild_D <- droplevels(subset(metadat_feeding.guild,
    Performance2 != "diversity"))
# rerun VCV matrix
V.d2.shared.lnRR_feeding.guild_D <- VCV.shared(data = metadat_feeding.guild_D,
    cV = "cV.lnRR", V = "lnrr.vi", cluster = "sharedcontrolcluster",
    obs = "infoid")

Q1B.Guild_D.reml <- rma.mv(yi = lnrr.yi, V = V.d2.shared.lnRR_feeding.guild_D,
    mods = ~feeding.guild - 1, random = list(~1 | studyid, ~1 |
        infoid, ~1 | multipleoutcomecluster), data = metadat_feeding.guild_D,
    method = "REML")
summary(Q1B.Guild_D.reml)
```

```
##
## Multivariate Meta-Analysis Model (k = 815; method: REML)
##
##    logLik   Deviance        AIC        BIC       AICc
## -500.2505  1000.5010  1020.5010  1067.4466  1020.7770
##
## Variance Components:
##
##             estim    sqrt  nlvls  fixed                  factor
## sigma^2.1  0.0755  0.2748    116     no                 studyid
## sigma^2.2  0.0377  0.1941    813     no                  infoid
## sigma^2.3  0.0634  0.2517    522     no  multipleoutcomecluster
##
## Test for Residual Heterogeneity:
## QE(df = 808) = 7555.6454, p-val < .0001
##
## Test of Moderators (coefficients 1:7):
## QM(df = 7) = 46.0566, p-val < .0001
##
## Model Results:
##
##                                     estimate      se     zval    pval    ci.lb
## feeding.guildborer/miner              0.4353  0.1569   2.7752  0.0055   0.1279
```

```
## feeding.guildcell-feeder            0.0219  0.0590   0.3710  0.7106  -0.0937
## feeding.guildchewer                -0.0441  0.0483  -0.9128  0.3614  -0.1389
## feeding.guilddetritivore           -0.3561  0.1635  -2.1782  0.0294  -0.6765
## feeding.guildnectar/pollen-feeder   -0.4774  0.0916  -5.2094  <.0001  -0.6570
## feeding.guildparasitoid            -0.2048  0.1195  -1.7141  0.0865  -0.4389
## feeding.guildpredator              -0.1713  0.1864  -0.9186  0.3583  -0.5367
##                                     ci.ub
## feeding.guildborer/miner            0.7428   **
## feeding.guildcell-feeder            0.1375
## feeding.guildchewer                 0.0506
## feeding.guilddetritivore           -0.0357    *
## feeding.guildnectar/pollen-feeder  -0.2978  ***
## feeding.guildparasitoid             0.0294    .
## feeding.guildpredator               0.1941
##
## ---
## Signif. codes:  0 '***' 0.001 '**' 0.01 '*' 0.05 '.' 0.1 ' ' 1
```

Supplementary Fig. 9

Overlap between moderators: We use alluvial plots to visualize the degree of overlap between categories (i.e. within-moderator levels) for different pairs of moderators.

```
## # A tibble: 32 x 3
## # Groups:   Status [4]
##    Status     Invert.Order       n
##    <fct>      <fct>          <int>
##  1 Beneficial "Acari"            4
##  2 Beneficial "Astigmata"        1
##  3 Beneficial "Chilopoda"        1
##  4 Beneficial "Collembola"      12
##  5 Beneficial "Diplopoda "       1
##  6 Beneficial "Diptera"          6
##  7 Beneficial "Haplotaxida"      4
##  8 Beneficial "Hymenoptera"     95
##  9 Beneficial "Lepidoptera"     10
## 10 Beneficial "Mesostigmata"     4
## # i 22 more rows
```

```
## # A tibble: 15 x 3
## # Groups:   Status [4]
##    Status           feeding.guild            n
##    <fct>            <fct>                <int>
##  1 Beneficial       detritivore             14
##  2 Beneficial       multiple                18
##  3 Beneficial       nectar/pollen-feeder    77
##  4 Beneficial       parasitoid              47
##  5 Beneficial       predator                10
##  6 Other herbivore  borer/miner              9
##  7 Other herbivore  cell-feeder             88
##  8 Other herbivore  chewer                 151
##  9 Other herbivore  detritivore             10
## 10 Other herbivore  multiple                24
```

```
## 11 Significant pest borer/miner             13
## 12 Significant pest cell-feeder            122
## 13 Significant pest chewer                 277
## 14 Significant pest nectar/pollen-feeder     3
## 15 <NA>             multiple                14

## # A tibble: 18 x 3
## # Groups:   Status [4]
##    Status           Lifestage     n
##    <fct>            <fct>     <int>
##  1 Beneficial       adult       111
##  2 Beneficial       multiple     16
##  3 Beneficial       <NA>         39
##  4 Other herbivore  adult        79
##  5 Other herbivore  eggs          1
##  6 Other herbivore  larva        73
##  7 Other herbivore  multiple     46
##  8 Other herbivore  nymph        39
##  9 Other herbivore  pupa         12
## 10 Other herbivore  <NA>         32
## 11 Significant pest adult       102
## 12 Significant pest larva       211
## 13 Significant pest multiple     37
## 14 Significant pest nymph         7
## 15 Significant pest pupa          4
## 16 Significant pest <NA>         54
## 17 <NA>             adult         2
## 18 <NA>             multiple     12

## # A tibble: 15 x 3
## # Groups:   Status [4]
##    Status           Winged     n
##    <fct>            <fct>  <int>
##  1 Beneficial       both       4
##  2 Beneficial       no        38
##  3 Beneficial       yes      103
##  4 Beneficial       <NA>      21
##  5 Other herbivore  both     104
##  6 Other herbivore  no        67
##  7 Other herbivore  yes       25
##  8 Other herbivore  <NA>      86
##  9 Significant pest both      55
## 10 Significant pest no       176
## 11 Significant pest yes      128
## 12 Significant pest <NA>      56
## 13 <NA>             both      10
## 14 <NA>             no         2
## 15 <NA>             yes        2

## # A tibble: 12 x 3
## # Groups:   Status [4]
##    Status           Diet.specialisation     n
##    <fct>            <fct>               <int>
```

```
##  1 Beneficial       generalist            76
##  2 Beneficial       specialist            34
##  3 Beneficial       <NA>                  56
##  4 Other herbivore  generalist           135
##  5 Other herbivore  specialist            61
##  6 Other herbivore  <NA>                  86
##  7 Significant pest generalist           196
##  8 Significant pest specialist           189
##  9 Significant pest <NA>                  30
## 10 <NA>             generalist             2
## 11 <NA>             specialist             2
## 12 <NA>             <NA>                  10
```

```
## # A tibble: 22 x 3
## # Groups:   Status [4]
##    Status          Performance2             n
##    <fct>           <fct>                <int>
##  1 Beneficial      abundance               76
##  2 Beneficial      diversity                9
##  3 Beneficial      feeding efficiency       9
##  4 Beneficial      growth/development       4
##  5 Beneficial      reproduction             4
##  6 Beneficial      searching efficiency    52
##  7 Beneficial      survival                12
##  8 Other herbivore abundance               62
##  9 Other herbivore diversity               14
## 10 Other herbivore feeding efficiency      51
## # i 12 more rows
```

```
## # A tibble: 35 x 3
## # Groups:   Invert.Order [21]
##    Invert.Order feeding.guild            n
##    <fct>        <fct>                <int>
##  1 "Acari"      multiple                 4
##  2 "Astigmata"  detritivore              1
##  3 "Chilopoda"  predator                 1
##  4 "Coleoptera" borer/miner             11
##  5 "Coleoptera" chewer                 193
##  6 "Collembola" detritivore              1
##  7 "Collembola" multiple                11
##  8 "Diplopoda " multiple                 1
##  9 "Diptera"    detritivore             10
## 10 "Diptera"    nectar/pollen-feeder     2
## # i 25 more rows
```

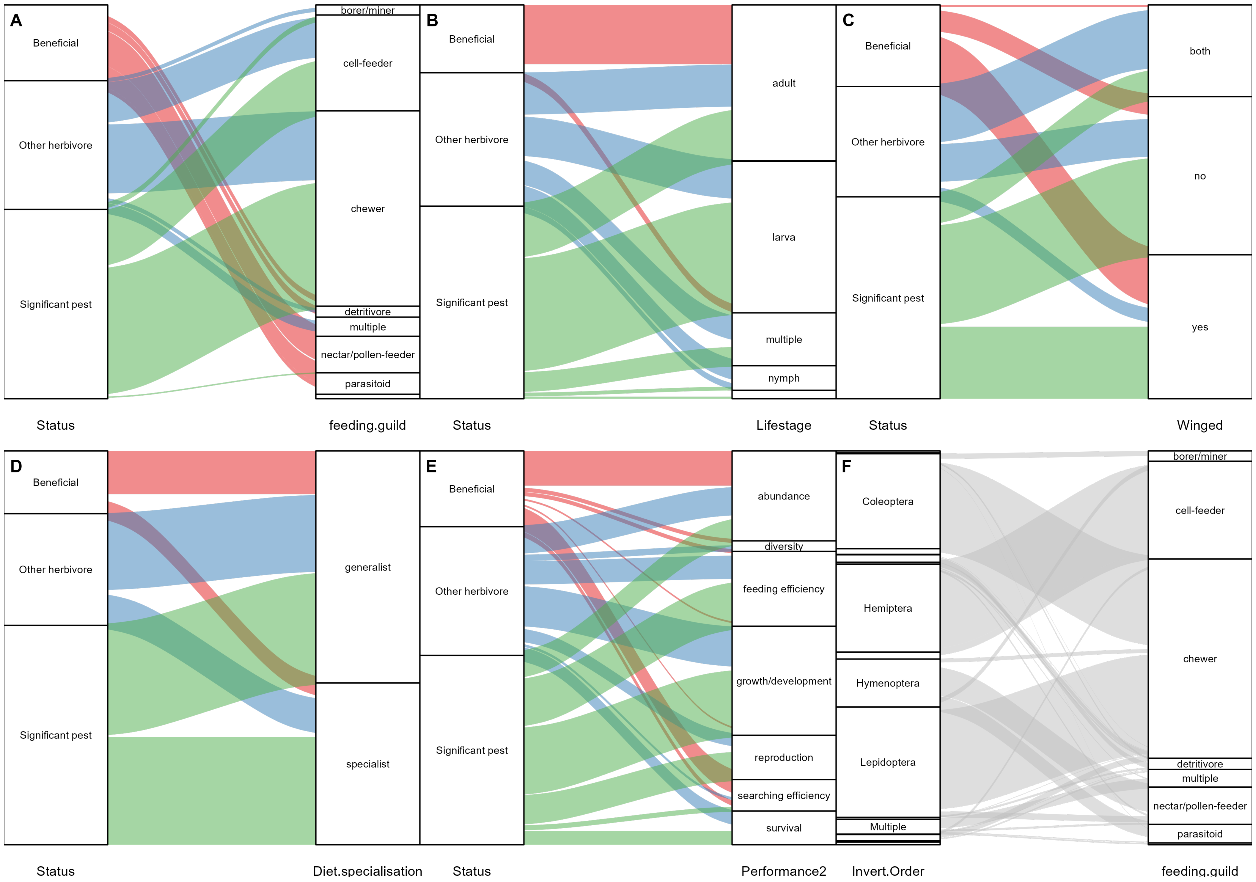

Supplementary Fig. 9. Alluvial plots showing overlap between levels within the moderators (A) pest status and feeding guild, (B) pest status and lifestage, (C) pest status and wing presence, (D) pest status and diet specialization, (E) pest status and invertebrate performance measure, and (F) invertebrate Order and feeding guild.

R Session Information

```r
sessionInfo()
```

```
## R version 4.3.2 (2023-10-31 ucrt)
## Platform: x86_64-w64-mingw32/x64 (64-bit)
## Running under: Windows 11 x64 (build 22621)
##
## Matrix products: default
##
##
## locale:
## [1] LC_COLLATE=English_United Kingdom.utf8
## [2] LC_CTYPE=English_United Kingdom.utf8
## [3] LC_MONETARY=English_United Kingdom.utf8
## [4] LC_NUMERIC=C
## [5] LC_TIME=English_United Kingdom.utf8
```

```
##
## time zone: Europe/London
## tzcode source: internal
##
## attached base packages:
## [1] stats     graphics  grDevices utils     datasets  methods   base
##
## other attached packages:
##  [1] ggpubr_0.6.0        ggalluvial_0.12.5   formatR_1.14
##  [4] rworldmap_1.3-8     sp_2.1-2            MCMCglmm_2.35
##  [7] ape_5.7-1           coda_0.19-4.1       here_1.0.1
## [10] corpcor_1.6.10      cowplot_1.1.2       R.rsp_0.45.0
## [13] patchwork_1.2.0     lubridate_1.9.3     forcats_1.0.0
## [16] stringr_1.5.1       dplyr_1.1.4         purrr_1.0.2
## [19] readr_2.1.5         tidyr_1.3.1         tibble_3.2.1
## [22] ggplot2_3.5.0       tidyverse_2.0.0     devtools_2.4.5
## [25] usethis_2.2.2       metafor_4.4-0       numDeriv_2016.8-1.1
## [28] metadat_1.2-0       Matrix_1.6-1.1      orchaRd_2.0
## [31] metaAidR_0.0.0.9000
##
## loaded via a namespace (and not attached):
##  [1] remotes_2.4.2.1    rlang_1.1.3        magrittr_2.0.3     compiler_4.3.2
##  [5] mgcv_1.9-0         systemfonts_1.0.5  vctrs_0.6.5        maps_3.4.2
##  [9] profvis_0.3.8      pkgconfig_2.0.3    fastmap_1.1.1      backports_1.4.1
## [13] ellipsis_0.3.2     labeling_0.4.3     utf8_1.2.4         promises_1.2.1
## [17] rmarkdown_2.25     sessioninfo_1.2.2  tzdb_0.4.0         ggbeeswarm_0.7.2
## [21] ragg_1.2.7         xfun_0.41          cachem_1.0.8       highr_0.10
## [25] later_1.3.2        terra_1.7-65       broom_1.0.5        parallel_4.3.2
## [29] R6_2.5.1           RColorBrewer_1.1-3 stringi_1.8.3      car_3.1-2
## [33] pkgload_1.3.4      estimability_1.5   Rcpp_1.0.12        knitr_1.45
## [37] fields_15.2        R.utils_2.12.3     splines_4.3.2      httpuv_1.6.13
## [41] R.cache_0.16.0     timechange_0.3.0   tidyselect_1.2.0   abind_1.4-5
## [45] rstudioapi_0.15.0  yaml_2.3.8         codetools_0.2-19   miniUI_0.1.1.1
## [49] pkgbuild_1.4.3     lattice_0.21-9     shiny_1.8.0        withr_3.0.0
## [53] evaluate_0.23      urlchecker_1.0.1   pillar_1.9.0       carData_3.0-5
## [57] tensorA_0.36.2.1   generics_0.1.3     rprojroot_2.0.4    mathjaxr_1.6-0
## [61] hms_1.1.3          munsell_0.5.0      scales_1.3.0       xtable_1.8-4
## [65] glue_1.7.0         cubature_2.1.0     emmeans_1.10.0     tools_4.3.2
## [69] ggsignif_0.6.4     mvtnorm_1.2-4      fs_1.6.3           dotCall64_1.1-1
## [73] grid_4.3.2         colorspace_2.1-0   nlme_3.1-163       raster_3.6-26
## [77] beeswarm_0.4.0     vipor_0.4.7        cli_3.6.2          textshaping_0.3.7
## [81] spam_2.10-0        fansi_1.0.6        viridisLite_0.4.2  gtable_0.3.4
## [85] R.methodsS3_1.8.2  rstatix_0.7.2      digest_0.6.34      farver_2.1.1
## [89] htmlwidgets_1.6.4  memoise_2.0.1      htmltools_0.5.7    R.oo_1.25.0
## [93] lifecycle_1.0.4    mime_0.12
```

Supplementary References

1. Dohmen GP, McNeill S, Bell JNB. Air pollution increases Aphis fabae pest potential. Nature 307, 52-53 (1984).

2. Senior AM, Viechtbauer W, Nakagawa S. Revisiting and expanding the meta-analysis of variation: The log coefficient of variation ratio. Research Synthesis Methods 11, 553-567 (2020).

3. Egger M, Smith GD, Schneider M, Minder C. Bias in meta-analysis detected by a simple, graphical test. BMJ 315, 629-634 (1997).

4. Nakagawa S, Senior AM, Viechtbauer W, Noble DWA. An assessment of statistical methods for non-independent data in ecological meta-analyses: Comment. Ecology 103, e03490 (2022).
