# Supplementary material for: Air pollution disproportionately impairs beneficial invertebrates: a meta-analysis
Source: Nat Commun. 2024 Jul 11;15:5447. doi: 10.1038/s41467-024-49729-5 (PMC11239652; doi:10.1038/s41467-024-49729-5)
Supplement: Supplementary file 3 — Description of Additional Supplementary Files [file 41467_2024_49729_MOESM3_ESM.pdf]

Supplementary Data 1
Meta-analysis dataset (Tab 1; detailed below) and references of studies used in the meta-analysis (Tab 2: Supplementary Table 1).

Meta-analysis dataset description:
These data were extracted from 120 studies (from 1978 to 2022) to conduct a global meta-analysis comparing the performance of different invertebrate groups/categories (e.g. pest status and feeding guild) in unpolluted and polluted atmospheres. The meta-analysis focused on the pollutants ozone (O_3), nitrogen oxides (NO_x), sulfur dioxide (SO_2) and particulate matter (PM).

Authors (Column A; Extractor) extracted relevant data from the publications directly from text and tables, or using WebPlotDigitiser for figures.

Each publication was assigned a unique identifier (Column B; studyid) and each row (or effect size) in the dataset was assigned a unique identifier (Column C; infoid) for modelling residual heterogeneity. The first author (Column D; Author), year (Column E; Year), journal (Column F; Journal), title (Column G; Title) and country of publication (Column H; Country) was recorded.

Three categories of pollutant recorded were included to ease the sorting of pollutants recorded (Columns I,J,K; Pollutant, Pollutant2, Pollutant3). The method used in the study (Column L; Location) was also recorded.

The control or ambient and elevated pollution level and their units of measurement was recorded (Column M; Control pollutant concentration and Column O; Elevated pollutant concentration, respectively) and both were scaled to ppb (for O_3, NO_x and SO_2) or μg/m^3 (for PM); Column N; cP_conc and Column P; eP_conc).

Various invertebrate parameters or groups were recorded, including the pest status (Column Q; Status), feeding guild (Column R; feeding guild), order (Column S; Invert.Order), family (Column T; Invert.Family), subfamily or species (Column U; Invert.species.subfamily), common name of species (Column V; Common name), lifestage (Column W; Lifestage) wing presence (Column X; Winged) and diet specialization (Column Y; Diet specialisation) of invertebrates.

The performance measurements were categorized (Column Z; Performance2) and the precise measurement (Column AA; measure) and associated units of measurement (Column AB; units) were recorded.

Where available, the plant order (Column AC; Plant Order), family (Column AD; Plant Family), species (Column AE; Plant Species) and common name (Column AF; Plant common name) associated with the invertebrate was recorded. The age (Column AG; Plant age), annuality (Column AH; Annuality) and type (Column AI; Monocot_Dicot) of plant was also recorded.

A detailed description of each moderator tested is described in the Supplementary Information published alongside the article.

Any additional and relevant information about the recorded statistic was included (Column AJ; Additional info). Within each study, results from treatment effects being compared to a shared control (e.g. where two different air pollution treatments are applied, and compared to the same control group) were assigned a unique identifier (Column AK; sharedcontrolcluster). A unique identifier was also used per experimental unit to indicate where more than one effect size is reported for the same individual or group (Column AL; multipleoutcomecluster).

The source of the data within each publication (e.g. which table or figure) is provided (Column AM; Source). The number of experimental units per invertebrate performance measurement (i.e. data row) for the control/ambient pollution treatment (Column AN; Nc) and elevated pollution treatment (Column AO; Ne) was recorded. The associated mean value, standard error and standard deviation was recorded for the control/ambient pollution treatment (Column AP, AR and AT; Xc, Error_c and Dev_C, respectively) and elevated pollution treatment (Column AQ, AS and AU; Xe, Error_e and Dev_e, respectively). Column AV (Pest Database) refers to whether invertebrate species' recorded were included in EPPO and/or CABI plant pest databases (see associated publication for further details).
