# Supplementary material for: Air pollution disproportionately impairs beneficial invertebrates: a meta-analysis
Source: Nat Commun. 2024 Jul 11;15:5447. doi: 10.1038/s41467-024-49729-5 (PMC11239652; doi:10.1038/s41467-024-49729-5)
Supplement: Supplementary file 5 — Reporting Summary [file 41467_2024_49729_MOESM5_ESM.pdf]

Corresponding author(s): James Ryalls (j.ryalls@reading.ac.uk)

Last updated by author(s): Jun 12, 2024

Reporting Summary

Nature Portfolio wishes to improve the reproducibility of the work that we publish. This form provides structure for consistency and transparency in reporting. For further information on Nature Portfolio policies, see our Editorial Policies and the Editorial Policy Checklist.

Statistics

For all statistical analyses, confirm that the following items are present in the figure legend, table legend, main text, or Methods section.

n/a	Confirmed	
☐	☒	The exact sample size (n) for each experimental group/condition, given as a discrete number and unit of measurement
☐	☒	A statement on whether measurements were taken from distinct samples or whether the same sample was measured repeatedly
☐	☒	The statistical test(s) used AND whether they are one- or two-sided<br>*Only common tests should be described solely by name; describe more complex techniques in the Methods section.*
☐	☒	A description of all covariates tested
☐	☒	A description of any assumptions or corrections, such as tests of normality and adjustment for multiple comparisons
☐	☒	A full description of the statistical parameters including central tendency (e.g. means) or other basic estimates (e.g. regression coefficient) AND variation (e.g. standard deviation) or associated estimates of uncertainty (e.g. confidence intervals)
☐	☒	For null hypothesis testing, the test statistic (e.g. F, t, r) with confidence intervals, effect sizes, degrees of freedom and P value noted<br>*Give P values as exact values whenever suitable.*
☒	☐	For Bayesian analysis, information on the choice of priors and Markov chain Monte Carlo settings
☐	☒	For hierarchical and complex designs, identification of the appropriate level for tests and full reporting of outcomes
☐	☒	Estimates of effect sizes (e.g. Cohen's d, Pearson's r), indicating how they were calculated

Our web collection on statistics for biologists contains articles on many of the points above.

Software and code

Policy information about availability of computer code

Data collection	We extracted numerical data from graphical figures using WebPlotDigitizer, version 4.6
Data analysis	All analyses were conducted in R ver 4.3.2. Annotated code for data analysis that support the findings of this study are available in the Supplementary Information including all packages and their versions. The full code (.rmd) is openly available via 10.17605/OSF.IO/UEQP7.

For manuscripts utilizing custom algorithms or software that are central to the research but not yet described in published literature, software must be made available to editors and reviewers. We strongly encourage code deposition in a community repository (e.g. GitHub). See the Nature Portfolio guidelines for submitting code & software for further information.

Data

Policy information about availability of data

All manuscripts must include a data availability statement. This statement should provide the following information, where applicable:

- Accession codes, unique identifiers, or web links for publicly available datasets
- A description of any restrictions on data availability
- For clinical datasets or third party data, please ensure that the statement adheres to our policy

The data that support the findings of this study are available in Supplementary Data 1. Source data for figures generated in this study can be found in the Source Data file.

Research involving human participants, their data, or biological material

Policy information about studies with human participants or human data. See also policy information about sex, gender (identity/presentation), and sexual orientation and race, ethnicity and racism.

Reporting on sex and gender	*Use the terms sex (biological attribute) and gender (shaped by social and cultural circumstances) carefully in order to avoid confusing both terms. Indicate if findings apply to only one sex or gender; describe whether sex and gender were considered in study design; whether sex and/or gender was determined based on self-reporting or assigned and methods used. Provide in the source data disaggregated sex and gender data, where this information has been collected, and if consent has been obtained for sharing of individual-level data; provide overall numbers in this Reporting Summary. Please state if this information has not been collected. Report sex- and gender-based analyses where performed, justify reasons for lack of sex- and gender-based analysis.*
Reporting on race, ethnicity, or other socially relevant groupings	*Please specify the socially constructed or socially relevant categorization variable(s) used in your manuscript and explain why they were used. Please note that such variables should not be used as proxies for other socially constructed/relevant variables (for example, race or ethnicity should not be used as a proxy for socioeconomic status). Provide clear definitions of the relevant terms used, how they were provided (by the participants/respondents, the researchers, or third parties), and the method(s) used to classify people into the different categories (e.g. self-report, census or administrative data, social media data, etc.) Please provide details about how you controlled for confounding variables in your analyses.*
Population characteristics	*Describe the covariate-relevant population characteristics of the human research participants (e.g. age, genotypic information, past and current diagnosis and treatment categories). If you filled out the behavioural & social sciences study design questions and have nothing to add here, write "See above."*
Recruitment	*Describe how participants were recruited. Outline any potential self-selection bias or other biases that may be present and how these are likely to impact results.*
Ethics oversight	*Identify the organization(s) that approved the study protocol.*

Note that full information on the approval of the study protocol must also be provided in the manuscript.

Field-specific reporting

Please select the one below that is the best fit for your research. If you are not sure, read the appropriate sections before making your selection.

☐ Life sciences ☐ Behavioural & social sciences ☒ Ecological, evolutionary & environmental sciences

For a reference copy of the document with all sections, see nature.com/documents/nr-reporting-summary-flat.pdf

Ecological, evolutionary & environmental sciences study design

All studies must disclose on these points even when the disclosure is negative.

Study description	This study conducted a global meta-analysis of 120 publications comparing the performance of different invertebrate groups (e.g. pest status and feeding guild) in unpolluted and polluted atmospheres. We focused on the pollutants ozone, nitrogen oxides, sulfur dioxide and particulate matter, applied in isolation (e.g. we removed a small number of effect sizes where combinations of pollutants were applied as there were too few for meaningful analysis). We used multi-level meta-analysis models to explain heterogeneity in responses of invertebrates to pollution, testing the explanatory power of a range of moderators (fixed effects). Where different (treatment) concentrations of air pollution were compared to a common control group, we modeled correlation of sampling errors using a variance covariance matrix. We also modeled the correlation of effect sizes where multiple measures of performance (e.g. abudance, growth) were reported on the same experimental unit using a random effect which was included in all models. Annotated code for all models is included in the supplementary information file.
Research sample	We used a systematic review, the process of which is fully outlined in the manuscript and supplementary information (via PRISMA diagram), searching Web of Science to identify all publications that reported measures of invertebrate performance in elevated air pollution (NOx, O3, SO2 and PM) and control conditions. A total of 120 publications met our inclusion criteria, these are listed in full in supplementary table S1.
Sampling strategy	We did not conduct any sample size calculations for this meta-analysis. The data used are from existing datasets.
Data collection	Authors J.M.W.R., L.M.B. and A.M. extracted relevant data from the publications, directly from text and tables, or using WebPlotDigitiser for figures. We list the source of the data within each publication (e.g. which table or figure) in Supplementary Data 1. The attribution of effect sizes to different categories (moderators and random effects) is explained in the manuscript (methods section 'defining categories') and supplementary information.
Timing and spatial scale	Our search of Web of Science was conducted on 10 November 2022. The year of studies included in the meta-analysis ranged from 1978 to 2022.
Data exclusions	Some publications were excluded during our systematic review process. 1215 articles were excluded following a title/abstract screen,

Data exclusions	and 231 were then full-text screened for eligibility, we excluded review articles (12), articles that did not include a comparison between elevated pollution and control (25), articles that were not accessible (5) and articles that had an unclear experimental design or no clear metric relating to invertebrate performance (69). Relevant details are presented in a PRISMA diagram in the supplementary information.
Reproducibility	We conducted a number of sensitivity analyses including leave-one-out analysis, where all effect sizes from each of the 120 publications is retracted from the analysis and the analyses re-run.
Randomization	During the systematic review process we checked that experimental units in publications were randomly assigned. Our meta-analysis focuses on controlled experimental comparisons of invertebrate performance in elevated pollutant concentrations vs control conditions.
Blinding	We did not use blinding during acquisition or analysis of data.

Did the study involve field work? ☐ Yes ☒ No

Reporting for specific materials, systems and methods

We require information from authors about some types of materials, experimental systems and methods used in many studies. Here, indicate whether each material, system or method listed is relevant to your study. If you are not sure if a list item applies to your research, read the appropriate section before selecting a response.

Materials & experimental systems

n/a	Involved in the study
☒	☐ Antibodies
☒	☐ Eukaryotic cell lines
☒	☐ Palaeontology and archaeology
☒	☐ Animals and other organisms
☒	☐ Clinical data
☒	☐ Dual use research of concern
☒	☐ Plants

Methods

n/a	Involved in the study
☒	☐ ChIP-seq
☒	☐ Flow cytometry
☒	☐ MRI-based neuroimaging

Plants

Seed stocks	*Report on the source of all seed stocks or other plant material used. If applicable, state the seed stock centre and catalogue number. If plant specimens were collected from the field, describe the collection location, date and sampling procedures.*
Novel plant genotypes	*Describe the methods by which all novel plant genotypes were produced. This includes those generated by transgenic approaches, gene editing, chemical/radiation-based mutagenesis and hybridization. For transgenic lines, describe the transformation method, the number of independent lines analyzed and the generation upon which experiments were performed. For gene-edited lines, describe the editor used, the endogenous sequence targeted for editing, the targeting guide RNA sequence (if applicable) and how the editor was applied.*
Authentication	*Describe any authentication procedures for each seed stock used or novel genotype generated. Describe any experiments used to assess the effect of a mutation and, where applicable, how potential secondary effects (e.g. second site T-DNA insertions, mosiacism, off-target gene editing) were examined.*
