# Supplementary material for: Air pollution disproportionately impairs beneficial invertebrates: a meta-analysis
Source: Nat Commun. 2024 Jul 11;15:5447. doi: 10.1038/s41467-024-49729-5 (PMC11239652; doi:10.1038/s41467-024-49729-5)
Supplement: Supplementary file 2 — Peer Review File [file 41467_2024_49729_MOESM2_ESM.pdf]

nature portfolio

Peer Review File

Air pollution disproportionately impairs beneficial invertebrates: a meta-analysis

Open Access This file is licensed under a Creative Commons Attribution 4.0 International License, which permits use, sharing, adaptation, distribution and reproduction in any medium or format, as long as you give appropriate credit to the original author(s) and the source, provide a link to the Creative Commons license, and indicate if changes were made. In the cases where the authors are anonymous, such as is the case for the reports of anonymous peer reviewers, author attribution should be to 'Anonymous Referee' followed by a clear attribution to the source work. The images or other third party material in this file are included in the article's Creative Commons license, unless indicated otherwise in a credit line to the material. If material is not included in the article's Creative Commons license and your intended use is not permitted by statutory regulation or exceeds the permitted use, you will need to obtain permission directly from the copyright holder. To view a copy of this license, visit http://creativecommons.org/licenses/by/4.0/.

Reviewers' Comments:

Reviewer #1:
Remarks to the Author:
This meta-analysis is interesting in evaluating the effects of air pollution on invertebrates. Although similar meta-analyses have been performed before (see my comment #2 below), this meta-analysis is novel in testing multiple invertebrate guilds and levels of pollution, and having a greater number of studies than previous meta-analyses. In particular, I found the results of beneficial invertebrates being more negatively affected by pollution than pests (but see my comment below), and even low concentrations having large effects, being noteworthy. This topic is extremely important and worth of attention, and the methods seem correct, although more information on how the authors classify the categories used is needed. Below, I present a few comments that need to be addressed.

Comment #1 – Classification of beneficial and pest invertebrates: I could not find the information on how the authors define this classification, but I deducted that beneficial invertebrates include pollinators, detritivores, and predators/parasitoids, whereas 'pests' are all herbivores (it's not clear if it's also including disease vectors). First, the authors must clearly state how they define these two groups. Second, and most importantly, if my deduction is correct, I find it problematic to classify all invertebrate herbivores as pests. Pests are usually defined as invertebrates that negatively impact humans, but not all herbivores will have these impacts. Importantly, insect herbivores are extremely necessary food sources for higher trophic levels (e.g., almost all birds feed on caterpillars), or can be pollinators as adults (as the authors briefly mention in the discussion), and hence will have ecological importance and help maintain ecosystem function. Classifying all invertebrate herbivores as pests only increases the problematic view of these taxa as a nuisance for humans, which in turn contributes to the lack of conservation efforts for these taxa. This classification needs to be changed in this manuscript before publication. If the 'pests' category only includes herbivores, I would suggest calling it 'herbivores' and then discussing that some herbivores can be considered as pests. Alternatively, if all herbivores are included in pests, maybe only herbivores in this category that are known to damage crops, and other herbivores could be classified as 'beneficial' (again, I'm not sure if this is what the authors have done already, but it seems unlikely to have so many data points of 'pests' that only includes known agricultural pests, unless it's biased to a certain study system).

Comment #2 – Consideration of other similar meta-analysis. The authors mention in the introduction that the importance of their meta-analysis is because "there has been no synthesis of this evidence to assess the broader impacts of air pollution on invertebrate communities." There have been at least 5 other meta-analyses that consider either ozone or multiple air pollutants effects on insects. I see that the authors cite a couple in the discussion. However, the existence of these meta-analyses would contradict their affirmation, and the novelty of their synthesis should be made clearer.
Here are a few meta-analyses about O3 and air pollution in general that I encountered recently:
Massad and Dyer 2010 -https://link.springer.com/article/10.1007/s11829-010-9102-7
Barker et al. 2018 (they measured O3) -
https://besjournals.onlinelibrary.wiley.com/doi/10.1111/1365-2435.13249
Zvereva and Koslov 2010 (already cited)
Valkama et al. 2006 (also considered O3, and its percent change)-
https://onlinelibrary.wiley.com/doi/10.1111/j.1365-2486.2006.01284.x
Mundim and Bruna 2016 (also considered O3) -
https://www.journals.uchicago.edu/doi/pdf/10.1086/687530?casa_token=KG-jymiaePMAAAAA:Dvr65mOGwUTaX-qUsoblka_YVKHkjf7JwybkjIx3aer0x-DWpwLrxzEIP0avwZu5MKCwRyP6slU

Comment #3 – Definition of performance measures: only in line 257 the authors allude to the fact that they are using a very broad definition of performance. I believe the reasoning for their choice of performance measures should be made early on. But even if their reasoning is to use a broad definition of performance, some of the choices seem odd. Diversity is not clearly connected to

individual performance and is rather a community metric. Additionally, the authors seem not to include an important performance measure: mass (either body mass, mass gain, etc). Also, a figure summarizing the results of pollutants on performance measures (similar to figure 3) would be helpful. I had a hard time going back to the text to find the results when reading the discussion. Similarly, most of the results about the moderators are only in text; it might be helpful to have figures for these results.

Comment #4 – More information overall about the moderators: I missed some explanation, especially in the introduction, of why the authors were testing some of the moderators. In particular, why would you expect to see differences in performance across functional groups? I can reason it myself after working for many years with insects, but for a general audience, it might not be as clear, and it would be helpful to get some background on how these functional groups would be expected to respond or be affected differently by pollution. Also, it would be helpful to include an overview of the moderators' data. In particular, I missed more taxa information within each feeding guild. For instance, how many pollinators were bees? The beneficial vs pests are composed of which percentage of functional groups/taxa? Or who are the predators (hymenopterans, spiders, etc)? Are you considering predators of detritivores in the 'predator' category or in the 'detritivore' category? If the former, would these predators still be considered 'beneficial'?

Minor comments:

Line 338: Please add more information on the first exclusion criteria that led from 1446 to 231 papers.
Line 348 and line 369 (averaging across studies to input missing data): would it be the case to also perform a sensitivity analysis removing these input data? Or at least some information on how variable the data is across studies to make the averaging feasible or not.
Line 362: Similar to comment #3, I was missing the reasoning for using these performance metrics (and even some that I would not consider as performance, such as diversity and what the authors consider as diversity).
Line 401: I would also appreciate more information on how the authors classify the moderators (e.g., what are considered as 'beneficial' invertebrates, as I mentioned in my previous comments).
Line 409: why use a model comparison instead of QM to assess the importance of each moderator?

Reviewer #2:
Remarks to the Author:
This manuscript provides a valuable meta-analysis of air pollution effects on invertebrates. The analysis scripts and details are well-documented and seem rigorous and appropriate from what I can determine, though it is often difficult to assess this fully.
The key result of this analysis is that air pollution disproportionately impairs beneficial invertebrates. How were beneficials differentiated from pests? To what degree does this reflect a trophic level separation between herbivores and higher trophic levels, vs something about the other characteristics of pest vs beneficial species? Are there species that were neither pest nor beneficial? Is there a difference between pest and non-pest herbivores, for example? For metrics like searching efficiency, how was this defined across trophic levels? It seems like the beneficials vs. pest comparison might be correlated with differences in trophic position, and also with differences in feeding guild. Could those different results all stem from a common cause?
The effects of O3 and NOx's were striking, compared with the weaker or less consistent effects of PMs and SOx's. It would be great to have more context on prior expectations for these differences. It seemed like the authors took pains to point out the lack of data on PMs in particular, though some sections of this paper seem to suggest that a greater emphasis on O3 and NOx management was warranted. Is that the intended message?
The variation in the NOx effect with plant taxonomy was interesting – was it different for N-fixers?

More generally, to what extent do the authors attribute the observed effects to direct effects on the inverts vs. indirect effects mediated via plant hosts or other members of the community?
The lack of a concentration effect was surprising, and it seemed like few studies provided much data on concentrations overall. This seems like a key gap to point out for future studies. To what degree was this due to a lack of power or leverage in available concentration data? One of the key results is that “even moderate levels of O3 and NOx adversely affect beneficial invertebrates”, but does this current study actually provide strong evidence for this? Do we know that the concentrations used in the current analysis were moderate, or within realistic ranges?
The effect of experimental method was also interesting – why? Potential interactions with temperature would be great to study in future.
L263: Interesting idea re: VOC dependence, but is there strong evidence for this? Seems like we don't often know this.

Reviewer comments (black), Author responses (red)

Reviewer #1 (Remarks to the Author):
This meta-analysis is interesting in evaluating the effects of air pollution on invertebrates. Although similar meta-analyses have been performed before (see my comment #2 below), this meta-analysis is novel in testing multiple invertebrate guilds and levels of pollution, and having a greater number of studies than previous meta-analyses. In particular, I found the results of beneficial invertebrates being more negatively affected by pollution than pests (but see my comment below), and even low concentrations having large effects, being noteworthy. This topic is extremely important and worth of attention, and the methods seem correct, although more information on how the authors classify the categories used is needed. Below, I present a few comments that need to be addressed.

Comment #1 – Classification of beneficial and pest invertebrates: I could not find the information on how the authors define this classification, but I deducted that beneficial invertebrates include pollinators, detritivores, and predators/parasitoids, whereas 'pests' are all herbivores (it's not clear if it's also including disease vectors). First, the authors must clearly state how they define these two groups. Second, and most importantly, if my deduction is correct, I find it problematic to classify all invertebrate herbivores as pests. Pests are usually defined as invertebrates that negatively impact humans, but not all herbivores will have these impacts. Importantly, insect herbivores are extremely necessary food sources for higher trophic levels (e.g., almost all birds feed on caterpillars), or can be pollinators as adults (as the authors briefly mention in the discussion), and hence will have ecological importance and help maintain ecosystem function. Classifying all invertebrate herbivores as pests only increases the problematic view of these taxa as a nuisance for humans, which in turn contributes to the lack of conservation efforts for these taxa. This classification needs to be changed in this manuscript before publication. If the 'pests' category only includes herbivores, I would suggest calling it 'herbivores' and then discussing that some herbivores can be considered as pests. Alternatively, if all herbivores are included in pests, maybe only herbivores in this category that are known to damage crops, and other herbivores could be classified as 'beneficial' (again, I'm not sure if this is what the authors have done already, but it seems unlikely to have so many data points of 'pests' that only includes known agricultural pests, unless it's biased to a certain study system).

RESPONSE:
Thank you for your excellent feedback and suggestions for improvement. As a result, we have redefined our grouping of invertebrates into three categories; significant pests, other herbivores and beneficial invertebrates. We have updated our analyses to investigate these three categories and incorporate definitions of each in the introduction and methods sections of the manuscript (lines 92-97 [L90-95 without tracked changes (TT)] and lines 409-427 [L393-411 without TT]).

Following the FAO, we had defined pest invertebrates as 'any species injurious to plants or plant products'. However, as you correctly indicate, some pests are considered more damaging to humans than others, so we have now improved the manuscript by differentiating between significant pests of economic importance and other herbivores (which may be either minor pests or non-pests). We have included clear definitions in the text in the introduction and methods sections of the manuscript. To summarise, we used the CABI Distribution Maps of Plant Pests (DMPP) and EPPO's Alert lists/Pest Risk Analyses (PRA) databases to search for individual species (where available from the source publication) to determine whether herbivore species were considered as economically-significant

pests. Pest species present in the DMPP/EPPO databases were defined as 'significant pests' and those species that were not included in DMPP/EPPO databases were defined as 'other herbivores'. Where source publications did not define pest invertebrates to species level we classified these as 'other herbivores'. We have updated the results and figures to reflect this change.

Note: The majority of pest invertebrates were herbivores, but there were some exceptions; adult stages of some DMPP-associated butterfly species (e.g. large cabbage white), for example, can pollinate plants, although they are often regarded as poor pollinators, with no significant ecological/economic benefit to humans. Any species that undergoes outbreaks that are known to result in significant damage of economic importance at some stage in their lifecycle were considered a pest for the purposes of this study (now described in the methods, lines 421-427 [L400-411 without TT]). In other words, where these species were defined in CABI's DMPP or EPPO's PRA/A1 list, both the adult and larval form were regarded as 'significant pests'. The full list of invertebrates defined as significant pests, other herbivores and beneficial invertebrates are shown in the supplementary data file provided.

After re-running the analyses, these changes did not alter our main findings (beneficial invertebrates remain adversely impacted by air pollution, while significant pests and other herbivores did not deviate significantly from zero), but we feel this change has improved the manuscript by clearly separating the plant pests of economic importance from other herbivores.

Comment #2 – Consideration of other similar meta-analysis. The authors mention in the introduction that the importance of their meta-analysis is because "there has been no synthesis of this evidence to assess the broader impacts of air pollution on invertebrate communities." There have been at least 5 other meta-analyses that consider either ozone or multiple air pollutants effects on insects. I see that the authors cite a couple in the discussion. However, the existence of these meta-analyses would contradict their affirmation, and the novelty of their synthesis should be made clearer.

Here are a few meta-analyses about O3 and air pollution in general that I encountered recently:
Massad and Dyer 2010 -https://link.springer.com/article/10.1007/s11829-010-9102-7
Barker et al. 2018 (they measured O3) -
https://besjournals.onlinelibrary.wiley.com/doi/10.1111/1365-2435.13249
Zvereva and Koslov 2010 (already cited)
Valkama et al. 2006 (also considered O3, and its percent change)-
https://onlinelibrary.wiley.com/doi/10.1111/j.1365-2486.2006.01284.x
Mundim and Bruna 2016 (also considered O3) -
https://www.journals.uchicago.edu/doi/pdf/10.1086/687530?casa_token=KG-jymiaePMAAAAA:Dvr65mOGwUTaX-qUsoblka_YVKHkjf7JwybkjIx3aer0x-DWpwLrxzEIP0avwZu5MKCwRyP6slU

RESPONSE:
We agree that there have been a range of meta-analyses that have investigated air-pollution; however, the context and aim of our analysis is very different to these previous analyses, and we admit that perhaps we did not articulate this clearly enough in our original manuscript. Therefore, we have now altered this paragraph in the introduction and included additional references of previous meta-analyses in the text (Introduction and Discussion). While previous meta-analyses have synthesized the effects

of some air pollutants on invertebrates, no studies have identified how we could effectively predict the broad impacts of air pollution across invertebrate communities.

Comment #3 – Definition of performance measures: only in line 257 the authors allude to the fact that they are using a very broad definition of performance. I believe the reasoning for their choice of performance measures should be made early on. But even if their reasoning is to use a broad definition of performance, some of the choices seem odd. Diversity is not clearly connected to individual performance and is rather a community metric. Additionally, the authors seem not to include an important performance measure: mass (either body mass, mass gain, etc). Also, a figure summarizing the results of pollutants on performance measures (similar to figure 3) would be helpful. I had a hard time going back to the text to find the results when reading the discussion. Similarly, most of the results about the moderators are only in text; it might be helpful to have figures for these results.

RESPONSE:
In our original drafts of the first version of the manuscript we had analysed the effects of air pollution on different performance metrics but omitted this from our final manuscript. We have now included a figure to clarify the effects of air pollution on different performance metrics for all pollutants combined and for individual pollutants (Supplementary Figure 2; Supplementary materials Section 4). We also now provide examples of specific metrics used for each performance parameter in Supplementary materials Section 3 where we list and describe all moderators in detail.

Our measures of invertebrate performance are in part due to our source publications, which referred to both individual species responses and the responses of groups of invertebrates. Diversity was included as a population or community performance metric where reported. This is now clarified in the introduction (lines 81-82 [L79-80 without TT]). We note that only 6 studies reported diversity and its removal had no effect on the main results (supplementary information section 6; line 400 [L384 without TT]).

Mass was already included in our study as a component of the ‘growth/development’ performance metric.

Comment #4 – More information overall about the moderators: I missed some explanation, especially in the introduction, of why the authors were testing some of the moderators. In particular, why would you expect to see differences in performance across functional groups? I can reason it myself after working for many years with insects, but for a general audience, it might not be as clear, and it would be helpful to get some background on how these functional groups would be expected to respond or be affected differently by pollution. Also, it would be helpful to include an overview of the moderators' data. In particular, I missed more taxa information within each feeding guild. For instance, how many pollinators were bees? The beneficial vs pests are composed of which percentage of functional groups/taxa? Or who are the predators (hymenopterans, spiders, etc)? Are you considering predators of detritivores in the ‘predator’ category or in the ‘detritivore’ category? If the former, would these predators still be considered ‘beneficial’?

RESPONSE:
We now include common invertebrate Families of each level for our two most important predictors (i.e. pest status and feeding guild) briefly in the text (methods lines 416-421 [L400-405 without TT) and in more detail in Supplementary Information Section 3, which now includes a detailed overview of each moderator and study/effect size counts under each level. Our feeding guild classifications are also now defined more clearly in the text (methods lines 418-421 [L401-405 without TT). The majority of beneficial invertebrates were nectar or pollen feeders (classified as pollinators for brevity) but also includes the feeding guilds ‘predators’, ‘parasitic wasps (i.e. parasitoids)’ and ‘detritivores’, all of which generally provide positive ecological or practical benefits to their environment. The most common Families of beneficial invertebrates were Apidae and Braconidae.

The introduction provides an overview of why it is important to identify generalizable groupings (moderators) so that we are able to better predict the effects of air pollution on invertebrates. Therefore, we previously avoided focussing on any individual moderators in the introduction prior to the results of the analysis. However, we now include ‘pest status’ as an example in the introduction so that this can be defined up-front for the reader (lines 92-97 [L90-95 without TT]), as suggested. Moreover, within-moderator comparisons are made for the most important groups in the Discussion and are now included in the Supplementary Information as alluvial plots to visualise the overlap between moderators (Supplementary Figure 9).

We have also now included the raw data as a supplementary file – The Order, Family and species (where available) for each feeding guild per study are listed here. This will be assigned its own DOI upon acceptance so that it will be easily accessible to readers interested in specific groups/classifications. In response to your specific question about predators, there were very few predators recorded and all predators included in the meta-analysis were considered beneficial (N = 4 studies and 11 effect sizes only). Further details are now provided in Supplementary Information Section 3 where all moderators are listed and described in detail.

Minor comments:
Line 338: Please add more information on the first exclusion criteria that led from 1446 to 231 papers.

RESPONSE:
This is now included in the Methods (lines 373-374 [L358-359 without TT]).

Line 348 and line 369 (averaging across studies to input missing data): would it be the case to also perform a sensitivity analysis removing these input data? Or at least some information on how variable the data is across studies to make the averaging feasible or not.

RESPONSE:
The sensitivity analysis now incorporates testing main model effects after excluding effect sizes where we imputed the control air pollutant concentration; there was no qualitative difference in the model findings (Supplementary materials Section 6).

Line 362: Similar to comment #3, I was missing the reasoning for using these performance metrics (and even some that I would not consider as performance, such as diversity and what the authors consider as diversity).

RESPONSE:
This is addressed in our response to comment #3 above.

Line 401: I would also appreciate more information on how the authors classify the moderators (e.g., what are considered as 'beneficial' invertebrates, as I mentioned in my previous comments).

RESPONSE:
This is addressed in our response to comment #1 above, the changes for which have greatly improved the manuscript, thank you again for the suggestion.

Line 409: why use a model comparison instead of QM to assess the importance of each moderator?

RESPONSE:
Our objective in this study was to identify simple and generalisable groupings that explain heterogeneity in invertebrate responses to elevated air pollution. We used likelihood ratio tests (LRT) to compare two nested models (a null model without the moderator and a full model including the moderator), to understand whether different moderators improved the model fit and to allow us to identify those moderators that best trade-off explanatory power and simplicity (ability to generalise). In our case, LRTs were less susceptible to bias than QM, especially when sample size was small. We also note that the QM statistic in metafor is by default an omnibus test, which would test the significance of all model coefficients rather than just those relating to the moderator of interest when more complex models are used.

Reviewer #2 (Remarks to the Author):

This manuscript provides a valuable meta-analysis of air pollution effects on invertebrates. The analysis scripts and details are well-documented and seem rigorous and appropriate from what I can determine, though it is often difficult to assess this fully.
The key result of this analysis is that air pollution disproportionately impairs beneficial invertebrates. How were beneficials differentiated from pests? To what degree does this reflect a trophic level separation between herbivores and higher trophic levels, vs something about the other characteristics of pest vs beneficial species? Are there species that were neither pest nor beneficial? Is there a difference between pest and non-pest herbivores, for example? For metrics like searching efficiency, how was this defined across trophic levels? It seems like the beneficials vs. pest comparison might be correlated with differences in trophic position, and also with differences in feeding guild. Could those different results all stem from a common cause?

RESPONSE:
Thank you for your excellent feedback and suggestions for improvement. The definitions and classifications of pest vs beneficial invertebrates was also queried by reviewer 1. As such, we now categorize pests and beneficials into 'significant pests', 'other herbivores' and 'beneficials' and include clear definitions and additional details in the text to clarify (introduction lines 92-97 [L90-95 without tracked changes (TT)] and methods lines 409-427 [L393-411 without TT]). We have updated all analyses based on these new categories. These changes did not alter the study outcome but we feel this change has improved the manuscript by clearly separating the plant pests of economic importance from other herbivores.

Regarding your concerns about the overlap between different moderator levels, we have now produced an additional panel (Supplementary Figure 9) to illustrate this. There was varying degrees of overlap between different moderators but no moderator pairs completely converged.

An example of each performance metric is now listed in Supplementary Information Section 3. Some performance metrics will inevitably be more associated with specific feeding guilds (e.g. feeding efficiency mostly consisted of pollinators or parasitoids, hence why it was negatively impacted by air pollution). We have now included the raw data as a supplementary file for review – this will be assigned its own DOI upon acceptance so that it will be easily accessible to readers interested in specific groups/classifications.

The effects of O3 and NOx's were striking, compared with the weaker or less consistent effects of PMs and SOx's. It would be great to have more context on prior expectations for these differences. It seemed like the authors took pains to point out the lack of data on PMs in particular, though some sections of this paper seem to suggest that a greater emphasis on O3 and NOx management was warranted. Is that the intended message?

RESPONSE:
In the discussion section we gave greater emphasis to O_3 and NO_x management because these were the pollutants where we had the strongest evidence of detrimental impacts from the meta-analysis. The effect of SO2 is marginally significant and, in general, the effects of SO2 and PM were less conclusive, and therefore further commentary on these pollutants would have been more speculative. We felt that it was important for us to emphasise the lack of studies on PM relative to the other pollutants to encourage further work on PM.

The variation in the NOx effect with plant taxonomy was interesting – was it different for N-fixers? More generally, to what extent do the authors attribute the observed effects to direct effects on the inverts vs. indirect effects mediated via plant hosts or other members of the community?

RESPONSE:
The effects of N-fixing Fabaceae were in line with other non-N-fixing plant taxa (Supplementary Information sections 3 and 4). Regarding the direct vs indirect (plant-mediated) effects, this is an interesting point and one that is debated amongst the research community in this field. In the discussion we theorise that the detrimental impacts of O_3, NO_x and SO_2 on beneficial invertebrates

stem from interactions between pollutants and VOCs in the air, impairing searching efficiency. It follows that these impacts would therefore be largely independent of the plants, however direct effects of pollution on the plants can in itself change their VOC emissions profile. This is obviously very challenging to disentangle in experimental studies, and more so in this meta-analysis of experimental evidence and therefore it is not possible to provide an absolute answer to the question of attribution of effect. As we state above, we have theorised the potential mechanism in the discussion and hope that this encourages further investigation into disentangling this network of effects by researchers in the field.

The lack of a concentration effect was surprising, and it seemed like few studies provided much data on concentrations overall. This seems like a key gap to point out for future studies. To what degree was this due to a lack of power or leverage in available concentration data? One of the key results is that “even moderate levels of O3 and NOx adversely affect beneficial invertebrates”, but does this current study actually provide strong evidence for this? Do we know that the concentrations used in the current analysis were moderate, or within realistic ranges?

RESPONSE:
Our results do indicate that pollutant concentration has surprisingly little effect. We explored several different analyses to try and understand this relationship. We would add that while close to significantly different from zero ($p=0.057$), as shown in figure 4A, the slope of lnRR with scaled concentration of the elevated pollutants is shallow, with a change in effect size of only 0.3 across the entire range of pollutant concentrations tested.
With respect to whether we provided evidence to state whether concentrations used in studies were moderate or within realistic ranges, in figure 4 panels B, D and E we included National Ambient Air Quality Standards for each pollutant as set by the United States Environmental Protection Agency to try and illustrate how the range of experimental concentrations compares to external benchmarks. These are national standards set to ensure pollutant levels remain below concentrations harmful to public health and the environment. Many of the data points fall below these levels, ozone being an exception; however, evidence (Air Quality Expert Group, 2021) suggests that local pollution episodes of ozone can see concentrations in excess of 200 ppb, particularly within or downwind of large conurbations and only two of the data points for ozone were over 200 ppb.

Furthermore, as shown in figure 2, the vast majority of effect sizes show negative impacts for beneficial invertebrates exposed to O_3, NO_x and SO_2.

The effect of experimental method was also interesting – why? Potential interactions with temperature would be great to study in future.

RESPONSE:
This finding was only significant for NO_x, with free air-enrichment (FAE) showing the strongest effect. It is difficult to speculate why this might have been the case, which is why we had avoided such speculation in our manuscript. It could be that FAE experiments often involve longer and more realistic durations and levels of exposure and that effects only emerge after increased exposure durations. However, this really is speculation and therefore we would argue that such commentary

should not be included in the manuscript.

L263: Interesting idea re: VOC dependence, but is there strong evidence for this? Seems like we don't often know this.

RESPONSE:
We explore the evidence for this towards the end of the same paragraph (now L295-308).

Reviewers' Comments:

Reviewer #1:
Remarks to the Author:
I believe the authors have addressed my previous comments extremely well, and I really enjoyed reading this new version of the manuscript.

I only have a couple of small comments/suggestions:

1. Suggestions about policies related to air pollution control and ozone (Lines 261-267, Lines 340-343, and last sentence of abstract): I can see where the authors are coming from, but I would suggest dialing down, explaining it more, or even remove this part about policies to reduce pollution. The effects of reducing NOx on O3 concentrations can be complex, and might depend on several factors not only NOx. For example, some studies done during the pandemic (when NOx concentrations were lower) have found contrasting effects on O3 concentrations (e.g., see review from https://www.sciencedirect.com/science/article/pii/S135223102200098X)

But also, NOx had negative effect on beneficial invertebrates as well, even though it had fewer data points and performance metrics. I believe we have to be extremely cautious when suggesting that policies to reduce air pollution could potentially be harmful, especially if you are not showing direct data regarding that.

2. Very minor: L 305-307 when talking about 'adaptation', I believe the authors might not mean adaptation in the evolutionary sense, right? If adaptation by natural selection is not what you mean here, edit this sentence to make it clear.

Reviewer #1:

Remarks to the Author:

I believe the authors have addressed my previous comments extremely well, and I really enjoyed reading this new version of the manuscript.

I only have a couple of small comments/suggestions:

1. Suggestions about policies related to air pollution control and ozone (Lines 261-267, Lines 340-343, and last sentence of abstract): I can see where the authors are coming from, but I would suggest dialing down, explaining it more, or even remove this part about policies to reduce pollution. The effects of reducing NOx on O3 concentrations can be complex, and might depend on several factors not only NOx. For example, some studies done during the pandemic (when NOx concentrations were lower) have found contrasting effects on O3 concentrations (e.g., see review from https://www.sciencedirect.com/science/article/pii/S135223102200098X) But also, NOx had negative effect on beneficial invertebrates as well, even though it had fewer data points and performance metrics. I believe we have to be extremely cautious when suggesting thatpolicies to reduce air pollution could potentially be harmful, especially if you are not showing direct data regarding that.

RESPONSE: We agree and have now adjusted the text within the abstract and discussion to reflect that policies to reduce air pollutant concentrations are recommended. The mention of policy and NOx/O3 interactions within the concluding summary have now been altered.

2. Very minor: L 305-307 when talking about 'adaptation', I believe the authors might not mean adaptation in the evolutionary sense, right? If adaptation by natural selection is not what you mean here, edit this sentence to make it clear.

RESPONSE: We have now adjusted the text here to make it clear that we do not mean adaptation in the evolutionary sense.
